# Investigating trait variability of gene co-expression network architecture in brain by controlling for genomic risk of schizophrenia

**DOI:** 10.1371/journal.pgen.1010989

**Published:** 2023-10-13

**Authors:** Eugenia Radulescu, Qiang Chen, Giulio Pergola, Pasquale Di Carlo, Shizhong Han, Joo Heon Shin, Thomas M. Hyde, Joel E. Kleinman, Daniel R. Weinberger

**Affiliations:** 1 Lieber Institute for Brain Development, Johns Hopkins Medical Campus, Baltimore, Maryland United States of America; 2 Group of Psychiatric Neuroscience, Department of Translational Biomedicine and Neuroscience, University of Bari Aldo Moro, Bari, Italy; 3 Department of Psychiatry and Behavioral Sciences, Johns Hopkins School of Medicine, Baltimore, Maryland, United States of America; 4 Department of Neurology, Johns Hopkins School of Medicine, Baltimore, Maryland, United States of America; 5 Department of Neuroscience, Johns Hopkins School of Medicine, Baltimore, Maryland, United States of America; 6 McKusick-Nathans Department of Genetic Medicine, Johns Hopkins School of Medicine, Baltimore, Maryland, United States of America; University of California Los Angeles, UNITED STATES

## Abstract

The effect of schizophrenia (SCZ) genetic risk on gene expression in brain remains elusive. A popular approach to this problem has been the application of gene co-expression network algorithms (e.g., WGCNA). To improve reliability with this method it is critical to remove unwanted sources of variance while also preserving biological signals of interest. In this WCGNA study of RNA-Seq data from postmortem prefrontal cortex (78 neurotypical donors, EUR ancestry), we tested the effects of SCZ genetic risk on co-expression networks. Specifically, we implemented a novel design in which gene expression was adjusted by linear regression models to preserve or remove variance explained by biological signal of interest (GWAS genomic scores for SCZ risk—(GS-SCZ), and genomic scores- GS of height (GS-Ht) as a negative control), while removing variance explained by covariates of non-interest. We calculated co-expression networks from adjusted expression (GS-SCZ and GS-Ht preserved or removed), and consensus between them (representative of a “background” network free of genomic scores effects). We then tested the overlap between GS-SCZ preserved modules and background networks reasoning that modules with reduced overlap would be most affected by GS-SCZ biology. Additionally, we tested these modules for convergence of SCZ risk (i.e., enrichment in PGC3 SCZ GWAS priority genes, enrichment in SCZ risk heritability and relevant biological ontologies. Our results highlight key aspects of GS-SCZ effects on brain co-expression networks, specifically: 1) preserving/removing SCZ genetic risk alters the co-expression modules; 2) biological pathways enriched in modules affected by GS-SCZ implicate processes of transcription, translation and metabolism that converge to influence synaptic transmission; 3) priority PGC3 SCZ GWAS genes and SCZ risk heritability are enriched in modules associated with GS-SCZ effects. Overall, our results indicate that gene co-expression networks that selectively integrate information about genetic risk can reveal novel combinations of biological pathways involved in schizophrenia.

## Author summary

Genetic risk for schizophrenia (SCZ) is linked to brain connectivity at multiple levels, from molecular interactions to coherent cognitive processing. Biological mechanisms by which SCZ genetic risk affects brain connectivity are generally unknown. In this study we sought to uncover some of these mechanisms by focusing on gene co-expression networks calculated from RNA-Seq data extracted from postmortem DLPFC. We created co-expression networks from gene expression adjusted to account for, or to remove the likely effects of genomic (polygenic) scores of SCZ risk. We minimized potential confounding effects of illness state or treatment on gene expression by using only brains from donors without a history of mental disorder. We used as a control, co-expression networks from gene expression adjusted to account for effects of genomic scores of height- a normative trait.

Our results show subtle and widespread effects of SCZ genomic scores on DLPFC co-expression networks. Gene sets from co-expression modules adjusted for SCZ genetic risk harbor biological significance represented by neuronal functions, but also by interacting pathways of general cellular processes like transcription, translation and metabolism. Our findings highlight possible avenues for identifying regulatory targets of gene expression in schizophrenia.

## Introduction

Crystalized in the name of the most severe psychotic illness—schizophrenia (SCZ)—is the definitory feature of this condition, a failure of connectedness with profound consequences for a person’s individual and social life. The fundamental notion of dysconnectivity- first proffered through clinical observation by Eugen Bleuler in his use of the term ‘schizophrenia’ [1]- has been also abstracted at various biological levels, including in neuroimaging connectivity studies [2–3] and in transcriptomic studies on post-mortem brain which have shown abnormal relationships between gene expression across brain regions [4–5]. Gene co-expression studies in the past decade have uncovered evidence for dysconnectivity at a molecular level, suggesting that how genes covary in expression is altered in schizophrenia, plausibly implicating alterations of gene regulatory networks with genetic and epigenetic origins [6–14]. These various studies have offered heuristic clues to the complex biology of complex psychiatric illness, but the fundamental mechanisms of how genetic risk translates into biological risk is largely unknown.

In this general context, the purpose of the present study was to explore how genomic risk for schizophrenia influences the architecture of gene co-expression in brain, and more specifically:

I. To estimate the contribution of genomic scores of SCZ risk (the trait of interest in our study) and height (the normative control)- to DLPFC gene expression and co-expression architecture.

II. To isolate differential functional patterns dependent on specific effects of genomic signatures of SCZ risk and height on co-expression network architecture, and further to explore their biological relevance. The current state of the art in large scale genetic studies of schizophrenia has identified over 280 genomic loci that are associated with increased risk of schizophrenia and using computation approaches based on fine mapping and brain transcriptomics, likely causative genes in excess of several hundred have been implicated as accounting for many of these GWAS significant associations [15]. Each of these putative causative genes accounts for literally a tiny increment in individual risk, and even the sum of risk alleles computed as a polygenic risk score predicts only about 7% of liability [15]. These humbling statistics have challenged investigators to explore various approaches to systems biology in speculation that these various risk genes converge in gene networks that show greater biological effects at the systems level [16].

Large-scale transcriptomic studies have been a popular strategy to this end, the standard approach being the use of various statistical tools to summarize the genomic signal of SCZ (i.e., polygenic risk scores- PRS) and link it to parameters of gene expression or co-expression measured from postmortem brain [6, 10–11]. While these studies have revealed plausible relations between polygenic risk for SCZ and gene expression/ co-expression, the magnitude of effects has also been small. The generally accepted explanation for such modest findings is the multitude of biological and technical factors that simultaneously contribute in different proportions to the gene expression variation in the brain and obscure the readout of polygenic risk effects.

Notably, gene expression and especially co-expression networks are sensitive to effects of demographic and biological factors like age, sex [17–18], cell composition [19–20], treatment [21] and technical artifacts (e.g., tissue degradation) [22]. While these factors are probably confounders that need to be controlled, many of them overlap also with the biological signal of interest that needs to be preserved. Furthermore, as an additional layer of complexity, effects of pathological traits of interest on expression/ co-expression likely intertwine with effects of other normative or pathological complex traits, since some genetic signal is shared between multiple conditions [23–25].

**Fig 1:**
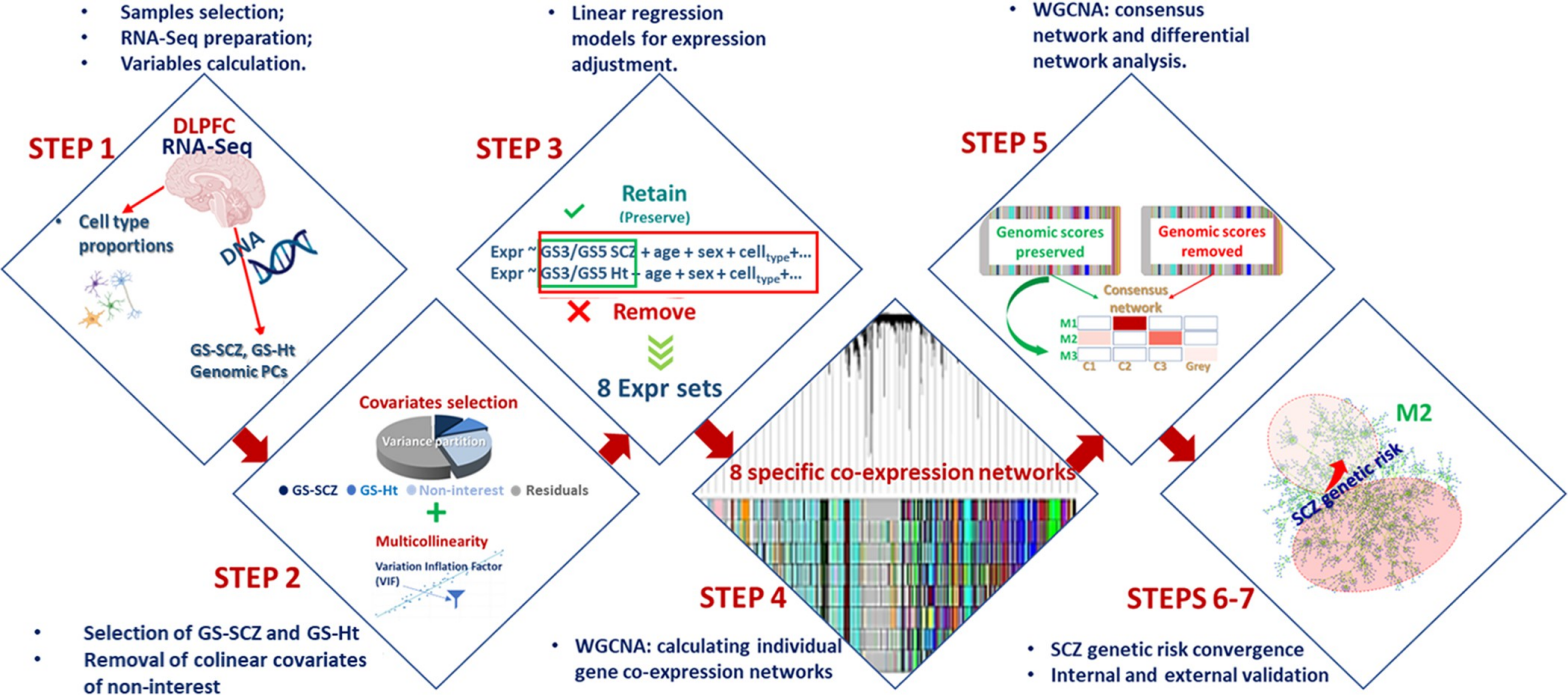
Analytical pipeline. *Step 1*: Preliminary processing of RNA-Seq data and calculation of variables for gene expression adjustment (genomic score of schizophrenia risk- GS-SCZ, genomic score of height- GS-Ht, cell type proportions by bulk RNA-Seq deconvolution, ancestry principal components- genomic PCs, observed and hidden technical artifacts) (part of the Step1 panel was created with templates from bioRender (https://app.biorender.com/). *Step 2*: Selection of covariates of interest and of non-interest for gene expression adjustment by using explained variance in gene expression and multicollinearity criteria. *Step 3*: Calculating eight linear regression models with gene expression as dependent variable, GS-SCZ and GS-Ht as covariates of interest alternatively preserved or removed, and other covariates of non-interest (age, sex, cell type proportions, genomic PCs and technical artifacts). *Step 4*: Calculation of gene co-expression networks with Weighted Gene Co-expression Analysis (WGCNA) routines from eight expression inputs (residuals extracted from step 3 linear models). *Step 5*: Calculation of background co-expression networks (networks neutral to genomic scores effects; schematically represented as C1-C3); identification of differential GS-SCZ and GS-Ht *preserved* modules (modules with weak or no correspondence in background, annotated as M1-M3); *legend*: M1 = example of a module with strong correspondence in background, M2 = module with weak correspondence and M3 = module with no correspondence in background. *Steps 6–7*: Further evidence of biological significance and specificity of modules that *preserve* GS-SCZ effects by convergence of SCZ genetic risk by internal and external validation.

Consequently, in the current study we sought to resolve some of this uncertainty by applying a novel design (**Fig 1**) in which gene expression input used for gene co-expression network construction and analysis is systematically adjusted to highlight or de-emphasize the biological signal of interest (here, polygenic profile of SCZ), while controlling for effects of other concurrent biological and technical confounders.

The biological signal (i.e., covariates of interest) was represented by genomic scores of SCZ risk and of height (as a control normative trait), while the covariates of non-interest were age, sex, cell type proportions, ancestry related genomic principal components, hidden and observed RNA quality artifacts). To obviate potential confounders related to the state of illness and associated treatment and other variables linked with chronic illness, we performed the study on the DLPFC from neurotypical adult brain.

By adopting this strategy, we created condition-specific co-expression networks from the same postmortem dataset that became its own comparator to study differential effects of complex traits genomic profiles on co-expression architecture.

Finally, we examined the internal and external validity of our co-expression networks by using various strategies, including replication in an independent data set and biological significance in principal represented by the convergence of genetic risk for SCZ in modules of co-expression.

## Results

### Gene expression variation with biological variables and technical confounders

Overall, known and hidden technical confounders, genomic PCs and other unknown factors, accounted for almost 70% of variance of gene expression. The rest of the variance was collectively explained by demographic (age, sex) and biological variables (cell type proportions, and genomic scores of SCZ and height), from which the largest part was attributable to neuronal cell type proportion- neurons (4.23%, max = 65.6%) followed by astrocytes- (1.15%, max≈50%) and GS3-SCZ (1.14%, max ≈24%). GS5-Ht, GS5-SCZ and GS3-Ht explained in order ≈1% (max≈20.46%), 0.72% (max = 22.4%), 0.7% (max≈16.6%) of gene expression variance (**S1 Fig**). Because GS3-SCZ, GS3-Ht, GS5-SCZ and GS5-Ht explained the greatest variance, these sets were selected as genomic profiles for adjustment of the expression input and estimating the effect on the co-expression architecture.

Notably, although biological variables in general and genomic scores in particular, explained little expression variation genome-wide, a number of genes showed significant deviation from the genome-wide trend (**S2 Table** and **S1 Fig**).

Results of cell-type decomposition with CIBERSORT showed almost perfect collinearity, but an inverse relationship between neuronal type and oligodendrocytes; we, therefore, excluded oligodendrocytes proportion from the analysis. Likewise, the algorithm failed to detect microglia both from the bulk RNA-seq data, and from an independent analysis of pseudobulk data created using a snRNA-Seq data [26]. While these results were of concern, we kept cell type proportions calculated with CIBERSORT for two principal reasons: our main interest in estimating cell-type proportions was to avoid identifying modules of co-expression mainly driven by cell type composition that could obscure more subtle differences potentially attributable to the genomic profiles’ effects on co-expression network architecture, not for statistical inference; furthermore, all co-expression networks were treated in the same way with respect to removal of unwanted covariance explained by cell type proportions; therefore we did not expect major differences directly related to these covariates.

Further, in order to evaluate the possible contribution of microglia to co-expression network architecture, we tested modules from the GS-SCZ *preserved* networks for the enrichment in microglial markers represented by a set of 881 genes predominantly expressed in human microglia [27]. In each co-expression network from *preserved* genomic scores, at least one module was significantly enriched in microglial markers with an overlap of >260 genes (**S3 Table**). Interestingly though, across the four networks, this putatively “microglial” module was depleted of SCZ risk heritability (**S19–S22 Figs**), further supporting the observation of reduced contribution of microglia to the co-expression networks of interest in our study.

### Gene co-expression network variability when adjusting the expression input to account for genomic signatures of SCZ risk and height (WGCNA results)

Co-expression networks and modules based on expression inputs adjusted to preserve or remove the effects of GS-SCZ and GS-Ht scores are summarized in the cluster dendrogram- heatmap plot in **S2 Fig**. Notably, the eight networks (i.e., two *preserved* and two *removed* for each GS level) had a comparable number of merged modules, varying between 40–47, and unmerged modules (52–65). By visual inspection, the co-expression network architecture indicates a good consistency across the eight networks with interspersed subtle variations potentially attributable to the adjustment of the expression input to account for genomic profiles effects (**S2 Fig**).

**Fig 2:**
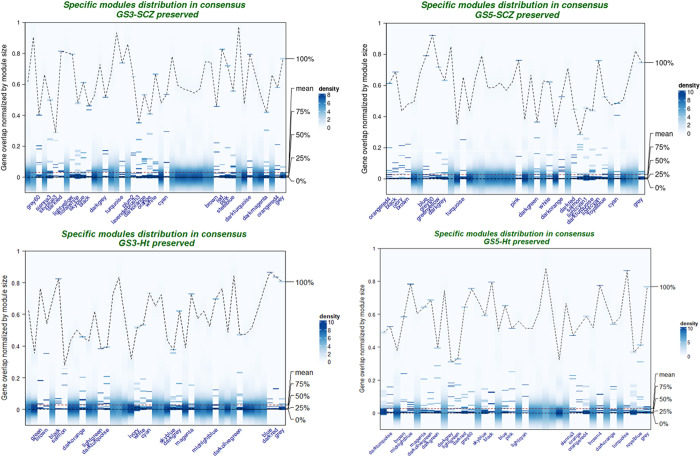
Kernel density maps- the distribution of overlap between GS-SCZ, GS-Ht preserved modules and background modules. Annotated modules on the x axis represent fragmented modules with weaker conservation in their background modules.

For differential co-expression network analysis, we used pairwise consensus between GS-SCZ *preserved* and *removed* and GS-Ht *preserved* and *removed* networks to generate the background networks for each GS group, followed by assessing the distribution of the *preserved* modules in the respective background modules. Relative to the three patterns described in the **Methods** section, this analysis showed a clear prevalence of weak conservation of individual modules in background modules, respectively; thus, while each module from the GS-SCZ/ GS-Ht *preserved* networks had correspondents in the background network, some modules were discretely segregated in two or more background modules (**Figs 2** and **S3–S6**). The density heatmaps in **Fig 2** show that, in fact, within each GS-SCZ or GS-Ht *preserved* network, approximately half of the merged modules were distinctly split in two or more background counterparts. Surprisingly, a similar, consistent and non-negligible pattern of distribution of individual GS-SCZ or GS-Ht merged *preserved* modules in background modules manifested across all background networks, suggestive of subtle and non-specific reconfigurations of co-expression architecture, allegedly owed to genomic profiles’ effects on gene expression (**Fig 2**).

In addition, we performed an exploratory analysis based on overlaying genes’ module memberships (i.e., kME) distributions of *preserved* and *removed* GS-SCZ and GS-Ht networks. The purpose of this analysis was to see if gene module affiliation shows a similar pattern of subtle shifts in co-expression network configuration that might be potentially explained by the adjustment of expression input. It is worth mentioning that in WGCNA, each gene of a co-expression network is assigned to only one module based on the correlations between its expression and with module eigengenes (MEs) of all modules. The final module affiliation is dictated by the highest correlation coefficient. In this case, examining the distributions of genes’ module memberships (i.e., the vector of kME for each gene across all modules) could be a more sensitive approach to explore the effects of expression input adjustments on network co-expression architecture. Our analysis of kME distribution revealed a high degree of overlap between histograms, with no major shifts; however, small but noticeable differences in histogram shapes were characteristic for all networks (**S7–S14 Figs**). This pattern was concordant with the WGCNA consensus analysis suggestive of small but widespread effects possibly due to the adjustments in the expression input to account for genomics scores effects.

### Evidence of genetic risk convergence in modules of *preserved* genomics scores

To further explore the strength of relationship between GS-SCZ and GS-Ht *preserved* modules and polygenic signatures effects on co-expression, we calculated the correlations between their module eigengenes (MEs) and genomic scores and the enrichment in priority PGC3 genes (step 6 of the analytical pipeline; **Fig 1**). The expectation here was that trait specific polygenic burden would be mainly concentrated in GS-SCZ and GS height preserved modules that were most deviant from their corresponding background modules. In other words, significant correlations between MEs and polygenic scores would be expected to characterize differential genomic scores *preserved* modules (i.e., modules with weak correspondence in background). Furthermore, we expected that PGC3 priority genes will be mainly enriched in differential GS-SCZ *preserved* modules.

The results of MEs- genomic scores correlations summarized in **Table 1** and **S15–S18 Figs** show that our expectations were only partially met:

In co-expression networks that preserve SCZ-risk genomic profile effects, significant MEs correlations with GS-SCZ were present for modules with both weak and strong correspondence in background modules. Notably though, in co-expression networks that preserve SCZ risk genomic profiles, no ME was correlated with GS-Ht. (**Table 1** and **S15 and S16 Figs**).

**Table 1:** GS-SCZ, GS-Ht *preserved* modules correlated with genomic scores and significant enrichment in PGC3 priority genes in LBD and PITT samples.

Genomic scores preserved networks	Positive correlations with GS-SCZ		Negative correlations with GS-SCZ		Positive correlations with GS-Ht		Negative correlations with GS-Ht	
	Strong corresp. A/B	Weak corresp. C/D	Strong corresp. A/B	Weak corresp. C/D	Strong corresp. A/B	Weak corresp. C/D	Strong corresp. A/B	Weak corresp. C/D
GS3-SCZ-SCZ LIBD (N = 47) **PITT (N = 47)**	3/24 **4/20**	4/23 **12/27**	1/24 (p = .001); 3/24 **1/20**	1/23 (p = .01); 1/23 **1/27**	NA **NA**	NA **1/27 (p = 0.0247)** **1/27 (p = 0.02)**	NA **2/20**	NA **4/27**
GS5-SCZ-SCZ LIBD (N = 42) **PITT (N = 45)**	5/22 **4/19**	1/20 (p = .02); 4/20 **4/26**	1/22 (p = 4e-04) **2/19**	1/20 (p = .003); 1/20 **2/26**	NA **NA**	NA **1/26 (p = 0.01)** **1/26**	NA **3/19**	NA **2/26**
GS3-Ht LIBD (N = 43) **PITT (N = 44)**	4/26 **1/19**	2/17 **4/25**	NA **NA**	1/17 (p = .03); 1/17 **NA**	3/26 **3/19**	2/17 **1/25 (p = 0.0004)** **1/25 (p = 0.03)** **4/25**	* **1/26** **5/19**	1/17 (p = .006); **7/25**
GS5-Ht LIBD (N = 40) **PITT (N = 51)**	2/18 **2/19**	1/ (22) **4/32**	1/18 (p = 4e-04) **NA**	2/22 **1/32**	2/18 **1/19**	2/22 **1/32 (p = 0.006)** **4/32**	NA **5/19**	1/22 (p = .02); **11/32**

*Legend*: N = total number of modules; A/B = number of GS-SCZ, GS-Ht *preserved* modules with strong correspondence in background modules and ME correlated with genomic scores, over the total number of GS-SCZ, GS-Ht *preserved* modules with strong correspondence in background modules; C/D = number of GS-SCZ, GS-Ht *preserved* modules with weak correspondence in background modules and ME correlated with genomic scores, over the total number of GS-SCZ, GS-Ht *preserved* modules with weak correspondence in background modules; A/B and C/D followed by p values are also significantly enriched for PGC3 prioritized genes (p = permutation p value). Strong, Weak corresp. = GS-SCZ, GS-Ht *preserved* modules with strong, respectively weak correspondence in background modules; one module in bold font marked with *- the only module with ME significantly correlated with GS-SCZ and GS-Ht; NA = not available; results with PITT data set in bold.

Likewise, in co-expression networks that preserve height genomic profile effects, MEs correlations with genomic scores were also present for modules weakly or strongly conserved in the background modules. However, in contrast with GS-SCZ *preserved* networks, in the Gs-Ht *preserved* networks MEs-genomic scores correlated with both GS-SCZ risk and GS-Ht. Of note, except for one module in the GS3-Ht *preserved* network, different modules correlated with GS-SCZ in comparison with GS-Ht (**S17–S18 Figs**).

We then assessed the distribution of PGC3 priority genes in modules that preserve effects of genomic scores on co-expression. Overall, PGC3 priority genes were significantly enriched in modules from all co-expression networks that preserve GS-SCZ or GS-Ht, with a trend of preponderance in modules negatively correlated with genomic scores. Nevertheless, the enrichment in PGC3 priority genes was not concentrated only in modules with weak correspondence in background modules (**Tables 1** and **S4**). Gene content for modules with a significant overrepresentation of PGC3 loci genes are in **S5 Table**.

We also assessed the distribution of trait heritability in the modules from networks of preserved GS-SCZ SCZ and GS height scores by performing stratified LD-score regression in our original sample of 78 DLPFC RNA-Seq data (LIBD sample).

**Fig 3:**
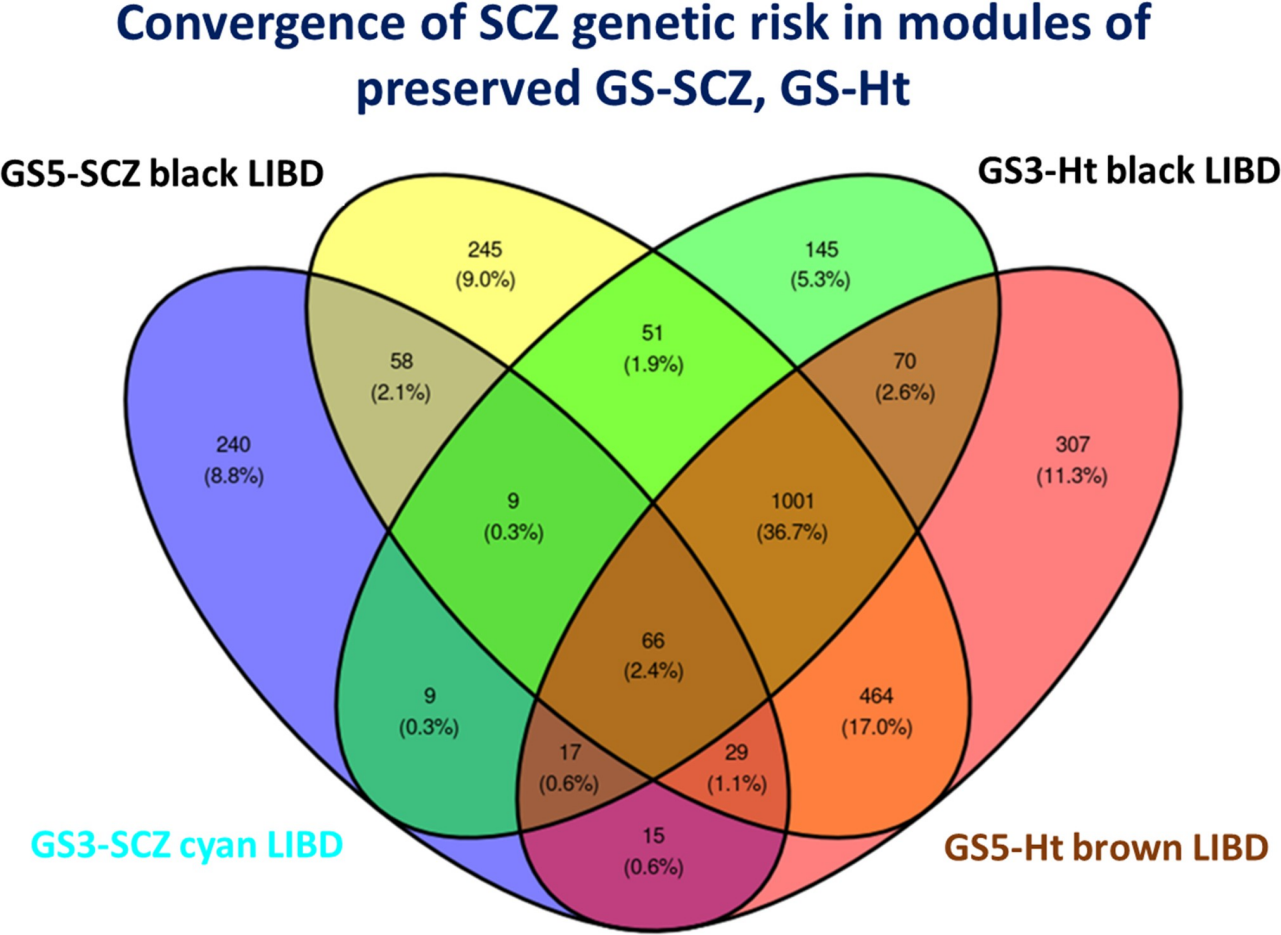
Gene overlap of modules that concentrate genetic risk of SCZ risk in all co-expression networks of *preserved* GS3-SCZ-GS5-SCZ and GS3-GS5 height.

The main results of this analysis showed:

1) A general trend for dissociation in trait heritability, with modules enriched for one trait and “depleted” for the other one. The largest concentration of this pattern was in the SCZ GS3-SCZ “preserved” network with 9 modules enriched for SCZ heritability and depleted for height.

2) Between 2–3 modules per network showed an overlap of enrichment for the two traits, consistent with the idea of a continuum between normal and pathological traits potentially suggestive of pleiotropy.

3) Between 12–17 modules per network were enriched or depleted for heritability of only one trait. In this scenario, height heritability was predominant irrespective of the “preserved” genomic score, GS-SCZ SCZ risk or GS height, which seems consistent with a more pervasive relation between a normative trait and gene co-expression in general.

4) Importantly, across all networks, a cluster of 66 genes was shared by all network specific modules that concentrate genetic risk for SCZ (i.e., MEs correlated with GS-SCZ SCZ risk, enriched for PGC3 genes and for SCZ risk heritability) (**Fig 3**), suggesting a convergence of genomic risk for schizophrenia irrespective of expression adjustment for genomic profile.

In summary, enrichment in trait heritability added a new layer of evidence for co-expression networks association with genomic risk of SCZ, the most interesting being the trend toward the relative divergence between SCZ risk and height heritability.

Detailed results of this analysis are in **S19–S22 Figs**.

### Results of internal and external validation

a. As an internal validation approach, we first created *artifact* co-expression networks and an *artifact* background generated through consensus analysis applied to average adjacencies from expression input adjusted to *preserve* or *remove* effects of re-sampled (shuffled) genomic scores GS3-SCZ SCZ risk and GS3-Ht, as explained in the methods section.

Then we examined the fragmentation pattern of unmerged modules from the *preserved* networks in the *artifact* background from shuffled GS3-SCZ, GS3-Ht scores.

Specifically, we tested the null hypothesis that the fragmentation pattern of modules from *preserved* shuffled scores in the *artifact* background is similar with the fragmentation pattern observed by overlapping modules from the *preserved* original scores with the real background. Rejecting the null hypothesis would indicate that effects of GS3-SCZ or GS3-Ht on the co-expression architecture are not just a consequence of the statistical adjustment of the expression input.

Results of this analysis summarized in the **S7–S9** Tables and **S1 Appendix** showed several important differences between the background and modules from *preserved* original scores and those from the *preserved* shuffled (artifact) scores.

First, the *artifact* background from shuffled scores had **no grey genes**, in contrast to the backgrounds based on original scores that had approx. 4,000 genes in grey (**S7 Table**). While not all the 50 networks respected the scale invariance property of the co-expression structure, this feature could be interpreted as association with a noisier graph structure, when using the exact same parameters for network identification. This is not surprising, considering this model derives from scrambled scores, but it is noteworthy that the scale invariance that is thought to be a hallmark of many biological networks is compromised when a trait relevant to the tissue at hand is reshuffled across subjects.

Second, the original GS3-SCZ preserved and GS3-Ht preserved networks had **more modules than networks from shuffled scores**: 47 merged and 60 unmerged (**S8 Table**), respectively 43 merged and 56 unmerged (**S9 Table**). In contrast, each of the 50 shuffled scores networks had around 40 unmerged modules (**S7 Table).**

Third, the original GS3-SCZ preserved and GS3-Ht preserved networks had a **smaller proportion of fragmented unmerged modules** (12/60- **S8 Table** for GS3-SCZ, respectively 14/56- **S9 Table** for GS3-Ht preserved), compared with shuffled artifact scores networks.

Most importantly, the null hypothesis that proportion of fragmented *preserved* original GS-SCZ is similar with the proportion of fragmented modules from the *preserved* shuffled scores was rejected for the GS3-SCZ (X-squared = 234.6, df = 50, p-value < 2.2e-16), however, not for GS3-Ht (X-squared = 49.047, df = 50, p-value = 0.5116). We interpret this result as evidence that, indeed, GS-SCZ effects carry more biological signal for brain tissue, such that altering the trait attribution to subjects confounds the co-expression architecture—whereas the GS from height, a trait less specific from brain tissue, does not share this feature.

Overall, these results further support the observation that polygenic background associated with complex traits, pathological or normal, can have detectable effects on the gene co-expression architecture in CNS.

b. External validation by biological significance.

**Fig 4:**
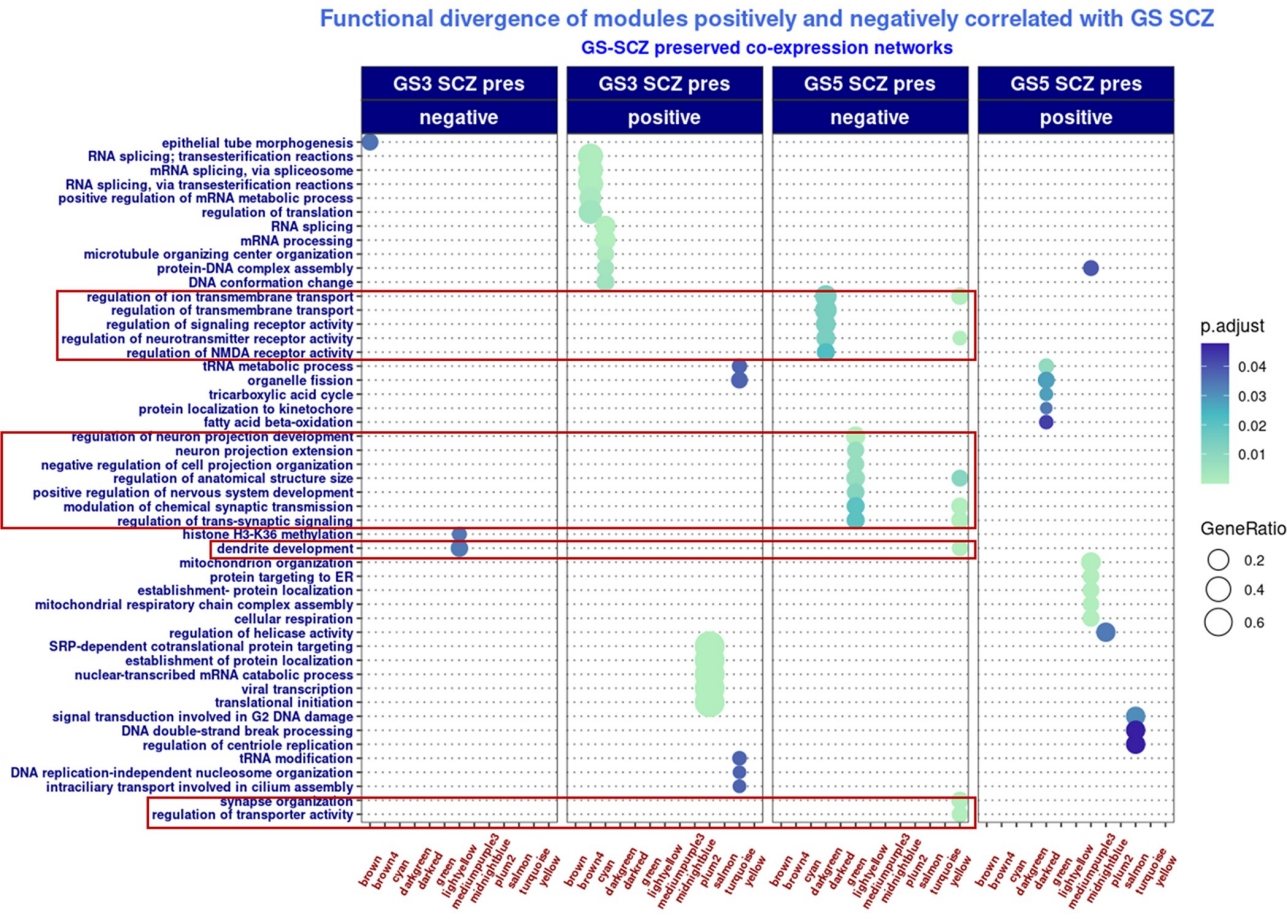
Functional profiling of GS-SCZ *preserved* modules with cumulative evidence for genomic risk effect on co-expression. Transcription, translation and metabolic ontologies are enriched in gene sets originated from modules with MEs positively correlated with GS-SCZs SCZ; nervous system development and functionality ontologies are enriched in gene sets originated from modules with MEs negatively correlated with GS-SCZs SCZ (highlighted by red rectangles). *Legend*: BG_GS3-SCZ_ / BG_GS5-SCZ_x = fragments from SCZ risk GS3-SCZ or GS5-SCZ preserved modules overlapped with background (BG) modules.

**Fig 5:**
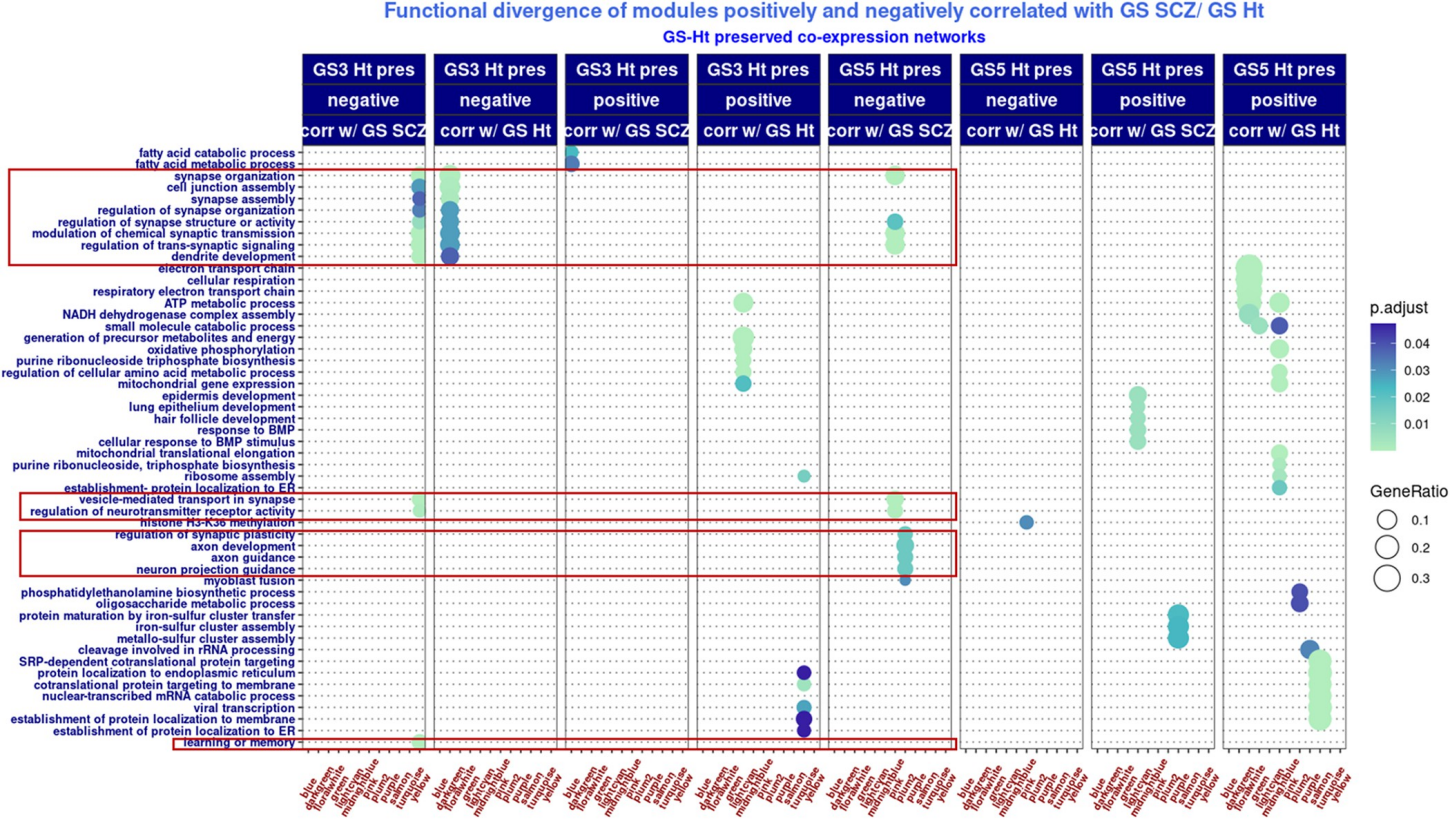
Functional profiling of GS height *preserved* modules with cumulative evidence for genomic risk effect on co-expression. Similar functional divergence in biological processes enrichment like GS-SCZ preserved modules: transcription, translation and metabolic ontologies are enriched in gene sets originated from modules with MEs positively correlated with GS-SCZs SCZ; nervous system development and functionality ontologies are enriched in gene sets originated from modules with MEs negatively correlated with GS-SCZs SCZ (highlighted by red rectangles). In addition, one set from GS3-Ht *preserved* lightgreen module negatively correlated with genomic scores of height was enriched in synapse related GO:BP. *Legend*: BG_GS3_ / BG_GS5_x = fragments from height GS3 or GS5 *preserved* modules overlapped with background (BG) modules.

To further assess the biological significance of co-expression modules with cumulative evidence for genomic profile effects on co-expression network architecture, we functionally profiled GS-SCZ risk and GS-Ht *preserved* modules with weak correspondence in their background modules and whose MEs were correlated with genomic scores of the two traits. From the respective modules, we isolated the fragments (i.e., gene sets with > 20 genes) dispersed in more than one background module and tested their enrichment in biological processes (GO:BP analysis). The functional profiling showed a divergent pattern, respectively: fragments derived from modules whose MEs negatively correlated with GS-SCZ were enriched for nervous system specific ontologies, whereas fragments from modules whose MEs positively correlated with GS-SCZ were enriched for ontologies related to general processes of transcription, translation and/ or metabolism. This divergent pattern was consistent for all modules correlated with GS-SCZ scores, irrespective of membership to GS-SCZ *preserved* or GS-Ht *preserved* networks (**Figs 4 and 5**). Of note, this was to be expected, as during the *preservation* step the effect of the other trait was not removed. To the extent the co-expression networks mirror the regulatory effects of genomic signatures of complex traits, this pattern is suggestive for a role in functional segregation between complex traits.

c. External validation by testing replication in an independent data set.

To assess the replicability of results with our approach, we used a data set from the NIH NeuroBioBank Brain and Tissue Repository (source- University of Pittsburgh) [28], which we refer to here as the PITT sample.

Our preliminary screening to evaluate the comparability of two data sets and potential sources for variation and reduced replicability, showed several differences between LIBD and PITT RNA-Seq samples (**S23 Fig**). The two samples differed also by demographic characteristics (i.e., age of death) and technical parameters of the expression data (**S24 Fig**).

Importantly for the replication effort, genomic scores of SCZ risk and height had a relatively similar pattern and distribution in the two samples (**S25 Fig**) and there were no differences in genomic scores between the two groups.

To test whether the impact of GS on gene expression is itself reproducible, we applied the same pipeline in PITT data set, by using only expressed genes common with LIBD (N = 17,767).

As expected, this analysis showed a relative concordance between the two data sets, but also some differences.

**Fig 6:**
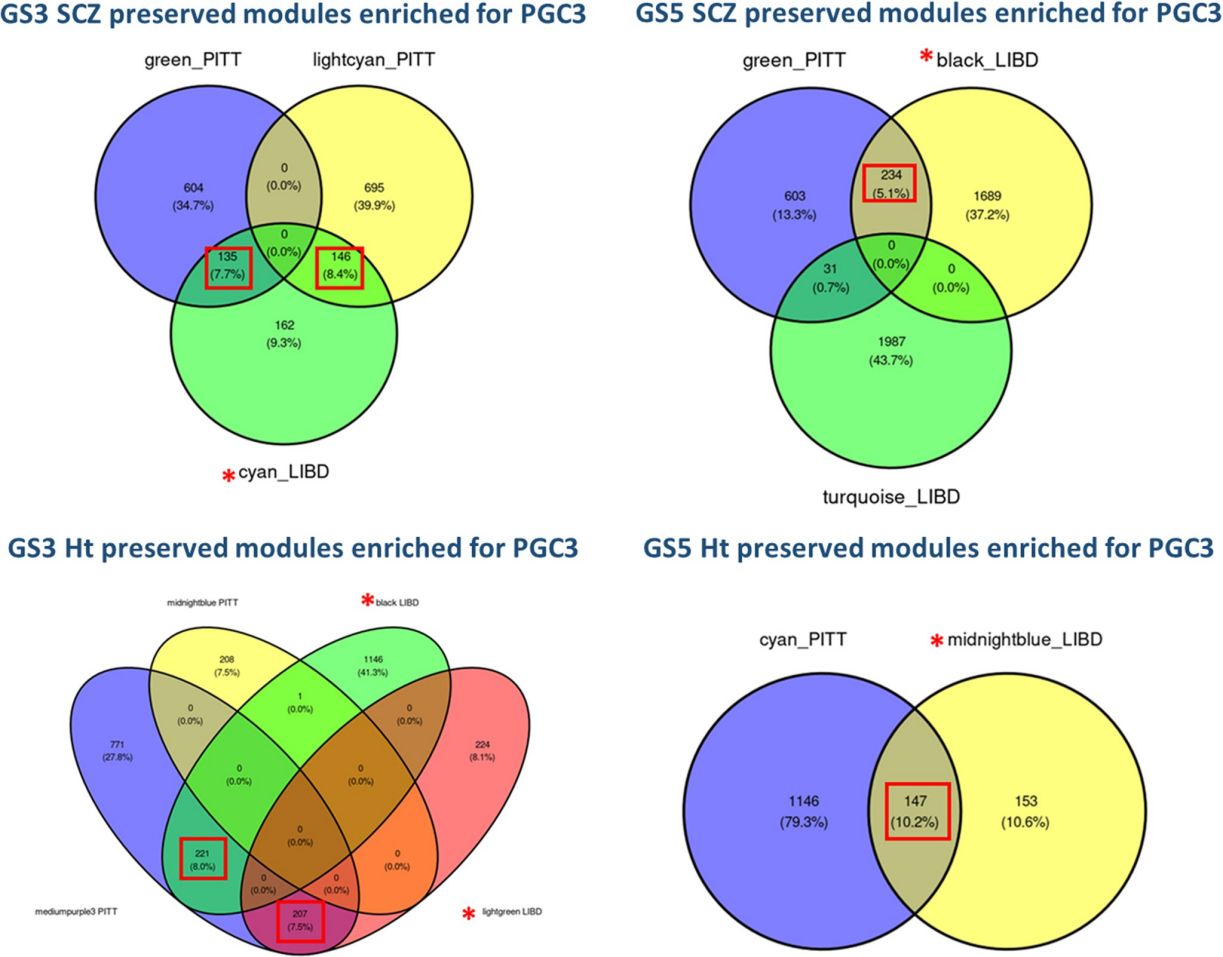
Gene overlap of modules that concentrate genetic risk of SCZ risk in co-expression networks of *preserved* GS3-SCZ-GS5-SCZ and GS3-GS5 height from LIBD dataset and the independent replication sample (PITT sample).

Concordance between LIBD and PITT samples:

1. A similar number of modules for the PITT eight co-expression networks (**S26 Fig)**.

2. A similar pattern of fragmentation of GS-SCZ and GS height preserved modules in background modules for PITT co-expression networks (**S27 Fig**).

3. Significant MEs- genomic scores correlations (SCZ risk GS-SCZ and height GS) for modules with both weak and strong correspondence in background modules (**Table 1** and **S28–S31 Figs**).

4. MEs-genomic scores correlations were present in co-expression networks of preserved scores for both traits, SCZ risk and height, generally with the same divergent pattern, respectively: MEs significantly correlated with GS-SCZ (genomic scores for SCZ risk) were not correlated with GS-Ht (genomic scores for height) or the directionality of correlation was opposite (positive for one trait, negative for the other and vice-versa) (**S28–S31 Figs**).

5. Overall, PGC3 prioritized genes were also enriched in modules from all co-expression networks irrespective of preservation for SCZ risk or height.

6. Most notably, there was a significant degree of overlap between the LIBD “cyan” module with the most evidence for enrichment in SCZ polygenic risk and PITT modules also harboring increased GWAS signal for SCZ risk (**Fig 6**). This result indicates the presence of a consistent and credible polygenic profile for SCZ risk with a role in co-expression network architecture.

B. Differences between LIBD and PITT samples:

1. In contrast to LIBD, although the modules with MEs correlated with GS-SCZ showed the same separation between neuronal functions and general cellular processes, the directionality of correlations was similar only for GS5-SCZ preserved network (neuronal functions in modules negatively correlated with GS-SCZ and more general cellular functions in modules positively correlated with GS-SCZ) (**S32 Fig**). Particularly in the GS-Ht *preserved* networks, correlations with GS-SCZ were scarce. However, the functional divergence was clear, and GO:BP related to neuronal functions were present only in modules positively correlated with GS Ht (**S33 Fig**).

2. Related to the above results and accounting that PGC3 loci genes are enriched for neuronal processes, it was not surprising that PGC3 gene enrichment, at least in GS height *preserved* networks, was in modules with MEs positively correlated with GS-Ht (**Table 1**).

In summary, notwithstanding several apparent discrepancies, PITT and LIBD modules of interest shared similar biological themes related to neuronal processes and general development (**S34 Fig**). Likewise, there was a non-negligible gene overlap between PITT and LIBD modules enriched for SCZ risk GWAS signal and related to nervous system functionality.

## Discussion

In this study, we explored the effects of SCZ risk polygenic burden on the architecture of co-expression networks from postmortem brain by applying a systematic adjustment of gene expression input to account for genomic scores calculated from the PGC3 SCZ GWAS. To better highlight potential GS-SCZ influences on co-expression networks, we controlled for known interfering effects on gene expression like those exerted by age, sex, race, cell type content, technical artifacts and illness epiphenomena. We used height- a normative complex trait- for qualitative comparisons with GS-SCZ effects on co-expression networks. Finally, we also applied methods of external validation to test the generalizability of our results.

The main findings of our study are:

I. Overall small variations in gene-wise expression based on the effects of genomic scores of SCZ risk and of height.

II. While the modular structure of gene co-expression architecture is generally conserved when modifying expression input to *preserve* or to *remove* the effects of SCZ risk and height scores, comparisons of networks based on preserved genetic risk with background networks, (i.e., those independent of genetic risk effects) reveal that many of the modules in the preserved networks are not conserved *in toto* in specific background modules but are fragmented. This suggests that genetic risk has a moderate impact on some aspects of co-expression architecture. A similar pattern of fragmentation was observed in an independent data set used for replication.

II. The subtle re-configurations reflected in the fragmentation of some *preserved* modules into more than one background module may reflect changes in gene expression correlations due to the accumulation of GS-SCZ and GS-Ht small effects on gene expression variability.

III. Surprisingly, modules of *preserved* GS-SCZ and GS height co-expression networks do not specifically represent genomic signatures of the two traits on co-expression architecture. If that would have been the case, the MEs correlations with genomic scores would have been predominant in *preserved* modules with weak correspondence in their background modules or in modules enriched for trait heritability. Likewise, significant enrichment in PGC3 priority genes would have been only in modules from GS-SCZ *preserved* networks, also preponderant in those with weak correspondence in their background modules or in those enriched for trait heritability. The more dispersed evidence across modules with strong and weak correspondence in the background modules suggests either subjacent *pleiotropy* [29] or reduced capacity of our measurements to capture specific effects of traits on co-expression networks (e.g., the lack of discriminative capacity of genomic scores, limitations of WGCNA in module detection, the lack of more complex statistical models for gene co-expression networks, etc.). Another explanation could also be a degree of polygenicity overlap between SCZ risk and height, a finding that was recently reported in a cross-trait LD score regression study [30].

IV. Notably, the functional profiles of co-expression modules with higher evidence for GS-SCZ and GS-Ht effects (i.e., *preserved* modules with weak correspondence in background and MEs correlated with genomic scores) implicate multiple biological pathways that belong to three generic biological processes: transcription, translation, and metabolism. The divergence of functional profiles associated with GS-SCZ and GS-Ht effects on co-expression networks is arguably driven by different combinations of pathways from these three major biological processes. A similar complex functional pattern was also observed in the co-expression modules from our replication sample.

V. Finally, the combinations of transcriptional, translational, and metabolic pathways are also intertwined with trait specific pathways and functions. For example, the functional profiles of some *preserved* GS-SCZ modules with lower correspondence in the background modules are linked to brain development and functionality (i.e., dendrites and axon projection development, regulation of transmembrane transport, synapse organization, etc.), while *preserved* GS-Ht modules with weaker representation in the background modules suggest a predominance of metabolic processes and signaling pathways related to organism growth (i.e., cellular respiration, response to BMP, epidermis development, myoblast fusion, etc.). Of note, the functional separation between *preserved* SCZ risk modules and *preserved* height modules, although present in the replication sample, was less consistent. However, despite such inconsistencies, we found a substantial overlap between modules from both samples that concentrate SCZ risk.

From a conceptual perspective, our study is best fitting a model of “network dyshomeostasis” that was initially proposed in inflammatory bowel disease (IBD) [31]. Essentially, this model suggests that a complex and heterogeneous disease (i.e., IBD or SCZ in our case) could be the result of dysfunctional cross-talk between multiple biological pathways and the potential perturbations can be triggered by genetic and environmental interactions, which affect homeostasis at the systems level.

In some respects, our results support also the recently proposed “omnigenic” model of common illness [32–33] especially through the multiple small variations of co-expression architecture, depending on the preservation or removal of GS-SCZ and GS-Ht effects. However, the main tenet of the omnigenic model, respectively the existence of core genes with direct effects on a trait and peripheral genes that act as master regulators of core genes, is not represented into our current study; the main reason is primarily the design in which we placed emphasis on potential functional perturbations inferred from co-expression networks, rather than focusing on finding specific genes.

Importantly, variations in transcription, translation and metabolic processes, some of which are highlighted in our study, have been previously amply documented as risk pathways in schizophrenia: transcription regulation largely through alternative splicing [34], translation dysregulation [35] and metabolic dysfunctions via bioenergetic decoupling at synaptic level [36–37].

Nonetheless, in our view, perhaps more important is the equilibrium between these processes sustained by an attuned and efficient cross-talk between pathways related to transcriptional regulation, translation and metabolism. Previous studies supporting this view showed complex interactions between the three fundamental processes in various tissues and systems [38–40]. At nervous system level, effects on the cross-talk between transcription, translation and metabolism putatively converge to produce a diverse palette of synaptic dysfunctions dependent on the cell types or compartments. From this angle, our findings from co-expression networks in the brain support the view that biological roles of genes in brain development and functions are mostly context dependent, with the polygenic background acting as a plausible modulator of the genes associated with SCZ vulnerability [16].

### Limitations

The relatively small sample size of the postmortem brain data sets in our co-expression network re-construction in comparison with studies based on larger consortium data (e.g., PsychENCODE) [10,13] is one important limitation of our study. However, we used more homogeneous samples including only neurotypical subjects of European ancestry to avoid population stratification confounders and chronic illness and treatment epiphenomena when creating the co-expression networks. Another possible limitation is related to the selection of the genomic profile of height as a representative complex trait for comparison with genomic risk for SCZ in terms of effects on co-expression network architecture. However, in the context of demonstrated shared genetic signal between complex traits, selection of control normative trait is not straightforward. We believe that our strategy to ensure that genetic scores of the SCZ risk and height are not highly correlated helped in avoiding a big functional overlap between genomic effects of the two traits on co-expression network architecture.

The main limitation related to reduced portability of genomic scores in prediction of heritable common pathological traits is the LD structure in a population [41]. While this was the main factor for preferring a smaller sample of Eur ancestry and although we controlled for genomic PCs, we cannot exclude that some effects of GS-SCZ and GS height effects on co-expression modules could be attributable to local ancestry and further studies are warranted to elucidate this issue.

Likewise, it worth reiterating that GWAS derived genomic scores are crude measures representing mixtures of genetic signal not specific to only one complex trait of interest. As suggested in [42–43], parsing genomic scores by biological pathways may provide a solution to increase their portability, especially for new therapies development.

While we were able to confirm a number of our observations in an independent data set, there were inconsistencies, perhaps to be expected considering the inherent differences between the samples. It is worth emphasizing that finding a replication data set in postmortem human brain with identical characteristics as the original data is particularly challenging and this is a major source for the lack of convincing replicability in the postmortem RNA-Seq studies, as it has been repeatedly highlighted in various studies and review articles [44].

We also mention important limitations related to cell-type decomposition algorithms suitable to resolve the cellular heterogeneity of bulk RNA-Seq data and to estimate with minimal bias cell type proportions necessary for data processing prior to co-expression network construction. While we did not specifically relate these measures to our modules of co-expression, we found at least a module substantially enriched for microglial markers in our networks, and thus we cannot exclude confounding effects of residual variance from incompletely removed cell types.

Finally, a note of caution should be made about potential misspecification of statistical models used in gene co-expression analysis. While little is known about regulation of gene co-expression in general, current models mainly relying on classical linear regression model are probably too simplistic for this kind of approach.

While our study explored a promising approach to integrate genomic regulatory effects with gene co-expression, future studies to refine the methodology of gene co-expression analysis, including complex statistical modeling, better module detection algorithms and expanding the repertoire of network parametrization, are warranted.

In conclusion:

1. Our results show that genetic signatures of complex traits (pathological or normal) reflected in genomic risk scores, have an important contribution to gene expression, plausibly through small, but cumulative effects from many individual genes. These effects, in turn, influence the architecture of gene co-expression networks.

2. On a more general note, our approach to specifically adjust gene expression to integrate the genetic risk for SCZ has the potential to identify clusters of genes that concentrate a large portion of genetic risk and can constitute biomarkers, useful for preventive or novel therapeutic approaches.

## Methods and materials

Step 1: Postmortem brain samples, RNA-Seq preparation and calculation of variables to enter the selection for gene expression adjustment

Data was acquired from assays of postmortem human brain tissue from the LIBD Human Brain Repository, collected under a protocol of standardized brain acquisition, processing, and curation (location, legal authorizations, informed consent, clinical review/ diagnosis) described elsewhere [4, 45] (further details about RNA-Seq data pre-processing and quality check are presented in **S1 Text** (**SM1**).

After pre-processing, a final gene expression (N = 18,980 genes) dataset of 78 DLPFC samples (age of death: 43 ± 15.8; M/F: 64/14) from neurotypical adults of European ancestry was retained for further analysis. Of note, 50 samples from the current study overlap with the cohort used in a previous study [6]. RNA sequencing data from the 50 samples accounts as new data, while it was re-sequenced by using libraries constructed with the TruSeq Stranded Total RNA Library Preparation kit with Ribo-Zero Gold ribosomal RNA depletion.

Variables targeted for gene expression adjustment were: demographic variables (age, sex), 10 sets of genomic scores associated with SCZ risk (GS1-10 SCZ) (also called polygenic risk scores- PRS) calculated from the latest PGC3 SCZ GWAS [15], 10 sets of genomic scores associated with a normative complex trait, height (GS1-10 Ht), calculated from a GWAS study of height [46] (for method of genomic scores calculations and definition of each set, see **S1 Text-SM 4** and **Fig 1**), and ancestry represented by genomic principal components.

Additionally, to avoid identifying modules of co-expression mainly driven by cell type composition that could obscure more subtle differences potentially attributable to the genomic profiles’ effects on co-expression network architecture, we used as covariates of non-interest cell type proportions estimated through an algorithm of partial cell-type deconvolution from bulk RNA-Seq data (CIBERSORT [47–48]). Of note, finding the appropriate algorithm for cell-type deconvolution is a matter of debate [49]. We selected CIBERSORT as presented in [48] based on the availability of several brain signatures and a webtool that calculates a goodness-of-fit measure to indicate what signature is the most appropriate for a specific data set [50]. We also evaluated the algorithm’s performance by analyzing pseudobulk data from a human snRNA-seq study on postmortem DLPFC [26] (S36 Fig). Details about cell-type decomposition are in supplementary methods (**S1 Text- SM3**). In addition, if the algorithm failed to compute the proportion of a cell type, we performed enrichment analyses by using permutations tests (**SM**) to determine if there are modules significantly enriched for markers of the respective cell type.

Finally, observed and hidden technical confounders were also used as covariates of non-interest for gene expression adjustment (more details are presented in supplementary information (**S1 Text- SM2**). The set of samples’ characteristics (demographic, ancestry principal components, genomic scores, cell type proportions) and RNA quality variables are in **S1 Table.**

Step 2: Variance partition analysis and selection of covariates of interest and non-interest for gene expression adjustment

The purpose of this screening step was twofold:

a. Selection of covariates of interest (i.e., sets of genomic scores of SCZ risk and height) by contribution to the variability in gene expression.

b. Estimating the collinearity of covariates of non-interest and removing colinear variables.

a. For the first part of the screening we used routines from the Bioconductor R package *variancePartition* [51].

Contribution of selected variables to the variation in expression of each gene from the DLPFC (gene expression matrix- 18,980 genes x 78 samples) was quantified by fitting linear regression models in two steps as suggested in [51–52]: initially, a regression model was fitted on gene expression to remove the variance explained by technical sources of variation and ancestry genomic PCs. Residuals extracted from this model were then used as input in subsequent regression models to calculate the contribution of biological covariates to the variation of each gene’s expression. Details about the model fitting and variance calculation are provided in **S1 Text- SM5**.

b. We tested for collinearity between all covariates of interest and non-interest, by using functions from the R packages *variancePartition* and *car* (**S1 Text- SM5**).

The final selection of genomic scores was based on gene-wise explained variance. Collinearity measures served as criteria for the final selection of the covariates of non-interest used for gene expression adjustment before co-expression networks calculation. We adopted the variables selection by criteria of explained variance and collinearity diagnostics to ensure that regressors used for expression data adjustment were the most informative and the linear regression models used for adjustment do not overcorrect the expression data. Furthermore, to maximize the probability of constructing relatively distinct co-expression networks from gene expression adjusted for GS-SCZ and GS-Ht, we also calculated the correlations between genomic scores of the two traits. If GS-SCZ and GS-Ht are not correlated, it is an indication that co-expression networks are reasonably trait specific in the context of an expected biological overlap.

Based on explained variance, we selected as covariates of interest 2 of the 10 sets of SCZ risk genomic scores (GS3-SCZ/ GS5-SCZ), and 2 of the 10 height genomic scores (GS3/ GS5-Ht). Of note, the GS3 set of genomic scores for SCZ risk and height is calculated from all SNPs with a GWAS significance of p<1e-04, while the GS5 set is calculated from all SNPs with a GWAS significance of p<0.01. For each trait these were the genomic scores that explained a higher variance of gene expression in our dataset (**S1 Fig**). Notably, GS-SCZ and GS-Ht sets were not significantly correlated (**S1 Fig**).

A note of caution is in order about the selection of two sets of genomic scores: while GS-SCZ SCZ-risk and GS-Ht were not correlated, GS3-SCZ-GS5-SCZ and particularly GS3-Ht-GS5-Ht showed strong correlations (**S35 Fig**). In this case, a legitimate question is why we selected these two sets of scores. Our reasons for this decision were:

Even if two variables are strongly correlated, they still could have different associations with the response variable and other variables in the model, which could induce different changes in the network architecture. Notably, two highly correlated variables could have different marginal distributions, which in turn will influence the correlations with other variables (**S35 Fig**). Finally, using 2 sets of scores accounted also as a control that allowed us to test the stability of co-expression networks to variations in genomic scores while identically controlling for other covariates of non-interest.

Based on collinearity diagnostics we discarded colinear regressors and selected as covariates of non-interest: age, sex, three cell-type proportions (neurons, astrocytes, endothelia), three observed RNA-quality measures, 10 ancestry genomic PCs and 5 RNA-quality surrogate variables (qSVs).

Step 3: Fitting multiple linear regression models for adjusting the expression input to account for effects of covariates of interest and non-interest.

For gene expression adjustment, we used linear regression models with expression data as dependent variables and variables selected in step 2 as independent regressors. For this purpose, we implemented a customized R function- *cleaningY* [53]- that removes the variance associated with variables of non-interest while retaining (“preserving”) the intercept and variables of interest, i.e., variables carrying the biological signal of interest (S37 and S38 Figs).

To this end we defined eight regression models that were fitted to alternatively retain or remove the variance explained by our covariates of interest selected in step 2- GS3-SCZ/ GS5-SCZ, respectively GS3/ GS5-Ht, while the variance explained by covariates of non-interest (age, sex, cell-type proportions, ancestry genomic PCs- snpPCs and technical confounders- i.e., observed technical parameters and quality surrogate variables qSVs) was consistently removed across all eight models.

The general equation for the eight linear regression models was:

***Expression*** *~ β*_*0*_ *+* β1*Genomic Score *+* β_2_*Age *+ β*_3_*Sex + β_4_*neurons + β_5_*astrocytes + β_8_*endothelia + β_10_*RIN + β_11_*totalAssignedGene + β_12_*mitoRate + *Σβ*_i_*snpPC_*i*_+ *Σβ*_j_*qSV_*j*_ where Genomic Score is represented by GS3-SCZ, GS5-SCZ SCZ, respectively GS3, GS5 height. Each of these score sets was alternatively *preserved* and *removed* with the *cleaningY* function (details about all variables, equations and implementation in **Fig 1** and **S1 Text- SM6**).

Residuals calculated from the eight regression models were used as expression input sets in weighted gene co-expression network analysis (WGCNA).

Linear models and principles of gene expression cleaning while preserving the biological signal of interest are explained in more detail in supplementary material (**S1 Text- SM6**).

Step 4: Co-expression network analysis with WGCNA

The eight new expression input datasets generated in step 3, representing the adjustments to *preserve* or *remove* the effects of GS3-SCZ or GS5-SCZ, respectively GS3 or GS5-Ht on gene expression, were used for calculating co-expression networks with WGCNA package [54] as previously described [6] (details in **S1 Text- SM7**).

Two patterns of the co-expression networks were generated for each trait and score: 1) Networks of retained *or preserved* genomic risk score (in statistical modelling jargon), in short, GS3-SCZ/ GS5-SCZ and GS3/ GS5-Ht *preserved* networks and 2) Networks of “regressed out”/ i.e., *removed* genomic risk score (again, in statistical modelling jargon), in short, GS3-SCZ/ GS5-SCZ, and GS3/ GS5-Ht *removed* networks.

The total of these eight co-expression networks (**Figs 1** and **S2**) were subsequently analyzed to identify patterns suggestive for distinct genetic signatures of complex traits that contribute to co-expression network architecture.

Step 5: “Background” network analysis and evaluating patterns of individual networks’ distribution in the background networks

To highlight differences in network architecture by variations in expression input, we performed pairwise consensus network analyses between the GS-SCZ *preserved* and *removed* and GS-Ht *preserved* and *removed* co-expression networks by using specific functions implemented in the WGCNA package (analytical flow for calculation of consensus network and detection of these consensus modules is schematically represented in **Fig 1**; details about method and parameters used for calculating the consensus are provided in **S1 Text- SM7B**.

Notably, consensus networks based on co-expression networks from input adjusted to *preserve* or *remove* genomic scores effects are representative of a background co-expression architecture free of the influence of trait specific genetic scores. In other words, we refer herein to a background network as co-expression of genes that are independent of influence from our genomic scores of interest, GS-SCZ or GS-Ht. In total, there are two background networks and sets of modules based on GS3-SCZ/ GS5-SCZ networks and two background networks and sets of modules based on GS3/ GS5-Ht networks. As a preferred approach to identify differential co-expression networks, we used the contribution of *preserved* networks for GS-SCZ and GS-Ht to their corresponding background co-expression networks (**Fig 1**). The rationale was that *preserved* networks likely carry the most relevant information about the effects of schizophrenia genomic risk on co-expression networks, i.e., the illness risk element on the biological systems. Therefore, we focused our analysis on the deviation of GS-SCZ, GS-Ht *preserved* networks from their corresponding background networks (**Fig 1**).

Correspondence of individual networks to their respective background networks is quantified as the gene overlap between their modules and the background modules. A Fisher’s exact test is used to determine the statistical significance of the overlap and heatmaps are generated to visualize the agreement between individual and background modules [55].

Relating individual GS-SCZ and GS-Ht relevant modules to background modules can show various degrees of correspondence with the respective background [55] as follows:

1. Strong preservation of module’s genes content in the background, that is a module significantly overlaps with only one correspondent background module, suggests little influence of GS-SCZ or GS-Ht on co-expression.

2. Relatively weak preservation of a module’s genes content in the background, respectively, a module significantly overlaps with 2 or more background modules; on the heatmap representing the overlap’s statistics this will appear as a discrete segregation of the specific module in several background modules. This pattern implicates lower influence of GS-SCZ or GS-Ht on co-expression.

3. A GS-SCZ or GS-Ht *preserved* module lacks a background counterpart and majority of its genes are in background “grey”, or the module appears fragmented in multiple background modules without any significant overlap. This pattern implicates GS-SCZ/ GS-Ht as a predominant factor in co-expression architecture.

The lack of background overlaps in pattern 3 would identify the most differential GS-SCZ risk/ GS-Ht *preserved* modules relative to their corresponding background modules, whereas the second pattern is suggestive of more subtle differences possibly related to variations in genes connectivity that lead to the “fragmentation” of the individual module in several background modules.

In addition, we also evaluated potential differences between GS-SCZ-GS *preserved* and *removed* modules of co-expression by inspection of module membership distribution (i.e., kME defined as the correlation between each gene expression and module eigengenes- MEs). The purpose of this qualitative analysis was to assess if large shifts in kME distributions are produced by adjusting the gene expression input to preserve or remove genomic scores effects (details are provided in the S1 Text- **SM11b**).

Step 6: Evidence of genetic risk convergence in modules of *preserved* genomic scores

Individual GS-SCZ/ GS-Ht *preserved* modules that showed a deviant pattern relative to the background modules were further investigated for enrichment in trait specific genetic signal or relevant functional profiles.

The purpose was to determine by additional lines of evidence if the differential GS-SCZ SCZ and GS height *preserved* modules concentrate the trait specific polygenic signature on co-expression architecture.

Specifically, we tested for correlations between our covariates of interest (GS-SCZ SCZ risk and GS height) and module eigengenes (MEs) of modules from networks that *preserve* GS3-SCZ-GS5-SCZ and GS3-GS5 height (**S1 Text- SM7D**). Of note, MEs are first principal components that summarize the gene expression profile in a module.

Then, we tested for module enrichments of the 120 priority genes highlighted in the latest SCZ GWAS (PGC3) [15], in short PGC3 genes. For this step, we used permutations tests (N = 100,000 iterations) to test hypotheses that differential modules are significantly enriched in PGC3 genes (**S1 Text- SM10**). Of note, although our main interest was finding additional evidence of polygenic profile effects on differential modules, the enrichment in PGC3 loci genes and correlations between MEs and genomic scores were applied to the entire set of genomic scores *preserved* modules of co-expression, irrespective of the strength of correspondence in the background modules.

We also tested for enrichment in trait heritability by using stratified linkage disequilibrium (LD) regression scores (S-LDSC) [56]. We used GWAS summary statistics of European ancestry for each trait that are publicly available (**PMID: 30124842,** PMID**: 35396580**). Following recommendations from the LDSC resource website (https://alkesgroup.broadinstitute.org/LDSCORE/), we ran S-LDSC for each list of genes in the *preserved* modules of co-expression. We used the baseline LD model v2.2 that included 97 annotations to control for the LD between variants with other functional annotations in the genome. To capture the regulatory regions of each gene, we defined gene intervals as a region spanning 500 kb upstream and downstream of the gene’s start and end positions. We used HapMap Project Phase 3 SNPs as regression SNPs and 1000 Genomes SNPs of European ancestry samples as reference SNPs. We downloaded all SNPs from the LDSC resource website.

Step 7: Internal and external validation of individual GS-SCZ/ GS-Ht *preserved* co-expression networks.

We tested the internal validity of co-expression networks that preserve the genomic scores effects by using permutations approaches to generate a null hypothesis. Specifically, we examined the pattern of modules’ fragmentation in the background for multiple co-expression networks that preserve the effects of re-sampled (shuffled) genomic scores.

The null hypothesis was that if the pattern of overlap between the original networks and the background is an artifact of gene expression adjustment and not a GS-SCZ effect, it would be recapitulated for the *preserved* networks from the permuted scores.

To this end, we applied the analysis for GS3-SCZ and GS3 height scores according to the pipeline represented in the **S39 Fig** and detailed in the supplementary material **(S1 Text- SM6).** Briefly, for each of the 78 DLPFC samples used in our study, we permuted/ shuffled each genomic score 50 times by using the *sample()* function in R. We then “cleaned”/ adjusted the expression data while *preserving* and *removing* the permuted GS3-SCZ and GS3 scores. The adjusted expression sets were used to create random co-expression networks and the corresponding background by applying the same WGCNA pipeline as for the original data. Specifically, we computed 100 *preserved* co-expression networks (50 for each score GS3-SCZ and GS3-Ht). For the background we calculated adjacency matrices from *preserved* GS3-SCZ, respectively GS3-Ht expression input (50 per score and adjustment method, in total 200 matrices of adjacency). We then calculated the average of adjacency matrices, one per score and adjustment method. From the mean adjacencies we computed the consensus network (background) and identified the background modules using the same procedure applied to define the background from the actual scores. The output was an average background and corresponding modules from the shuffled scores, GS3-SCZ, respectively GS3-Ht.

In summary, we generated the *preserved* shuffled GS3-SCZ and GS3-Ht networks and backgrounds to test the following hypothesis:

H_0_: Proportion of fragmented *preserved* original GS-SCZ and GS-Ht is similar to the proportion of fragmented modules from the *preserved* shuffled scores.

To test the H_0_ null hypothesis we used functions implemented in R (the *prop*.*test()*; details in supplementary material, S1 Text- SM11).

The results were summarized through heatmaps and density maps detailed in **S1 Text- SM12a**.

For external validation, we used two approaches: a. examining the biological significance of individual GS-SCZ, GS-Ht *preserved* modules deviant from the background modules and b. testing for replication in an independent data set.

a. To identify relevant and potentially trait distinct biological mechanisms likely affected by the influence of genomic scores on co-expression, we analyzed the differential modules by performing gene ontology enrichment analysis with functions implemented in the *clusterProfiler* R package [57] (**Fig 1** and **S1 Text- SM8**).

b. As an independent replication dataset we used a sample selected from the NIH NeuroBioBank Brain and Tissue Repository, collected at University of Pittsburgh [28] (henceforth named PITT sample). This data set was selected because it best approximated our original dataset, i.e., it included DLPFC RNA-Seq from 72 donors of European ancestry.

As a preliminary step, we performed a screening of our two sets, the original data (LIBD) and the replication set (PITT); the purpose was to identify potential sources for variation and reduced replicability. We started by examining the differences in demographic characteristics, genomic scores, and technical characteristics of the expression data.

After data screening for comparability, we applied the same pipeline in PITT data set, by using only genes common with LIBD (N = 17,767). In short, we performed expression input processing using the same model, and then we created co-expression networks that preserved or removed GS-SCZ and GS-Ht effects by using the same set of scores (i.e., GS3-SCZ-GS5-SCZ and GS3-Ht-GS5-Ht); finally, we computed the consensus (background) networks, and tested the overlap between GS-SCZ, GS-Ht *preserved* modules and the background.

More details and particularities of PITT data analysis are in the **S1 Text- SM9**.

## Supporting information

S1 Text Supplementary methods (SM). (DOCX)

S1 Table Demographics and technical characteristics of postmortem brain samples. (CSV)

S2 Table Partition of gene expression variance explained by biological and technical covariates. (XLSX)

S3 Table Modules enriched in microglial markers. (XLSX)

S4 Table PGC3 prioritized genes enrichment in modules of preserved SCZ GS-SCZ and GS-Ht; LIBD sample. (XLSX)

S5 Table PGC3 prioritized genes overlap in modules of preserved SCZ GS-SCZ and GS-Ht; LIBD sample. (XLSX)

S6 Table PGC3 prioritized genes enrichment in modules of preserved SCZ GS-SCZ and GS-Ht; PITT sample. (XLSX)

S7 Table Number of modules and grey genes in original vs. shuffled preserved GS3-SCZ and GS3-Ht networks and background. (CSV)

S8 Table Proportions of fragmented original vs. shuffled preserved GS3-SCZ in the background. (XLSX)

S9 Table Proportions of fragmented original vs. shuffled preserved GS3 height in the background. (XLSX)

S1 Fig Variance partition analysis for variables selection. **A:** Variance of gene expression explained by biological variables from highest to lowest; SCZ risk GS3-SCZ (p_GWAS_ = p<1e-04), GS5-SCZ (p_GWAS_ = p < .01) and height GS3-Ht (p_GWAS_ = p<1e-04), GS5-Ht (p_GWAS_ = p < .01) are score sets with higher contribution to gene expression variability; **B:** Heatmap showing highest correlations between height GSs, subsets of SCZ GS-SCZs and neurons-oligodendrocytes; correlations between GS-SCZ and GS-Ht are not significant (correlation coefficients: R_GS3-SCZ-GS3-Ht_ = .098, **R**_**GS3-SCZ-GS5-SCZ**_ **= .72**, R_GS3-SCZ-GS5-Ht_ = .034, **R**_**GS3-Ht-GS5-Ht**_ **= .94,** R_GS3-Ht-GS5-SCZ_ = .079, R_GS5-SCZ-GS5-Ht_ = .01).
(TIF)

S2 Fig Cluster dendrogram. Correspondence between co-expression networks calculated after adjusting the expression data to protect/ preserve, or remove variance explained by effects of genomic scores. *Legend*: N = number of modules from each network, grey = genes not assigned to modules; prot = protected or preserved; rm = removed.
(TIF)

S3 Fig Gene overlap between SCZ risk GS3-SCZ *preserved* modules and background modules. GS3-SCZ preserved modules on y axis; background modules calculated by consensus between GS3-SCZ *preserved* and GS3-SCZ *removed* networks on x axis. Numbers after modules color annotations = module size (total number of genes in the module). The color bar shows the significance of overlap (more intense red- more significant overlap).
(TIF)

S4 Fig Gene overlap between SCZ risk GS5-SCZ *preserved* modules and background modules. GS5-SCZ preserved modules on y axis; background modules calculated by consensus between GS5-SCZ *preserved* and GS5-SCZ *removed* networks on x axis. Numbers after modules color annotations = module size (total number of genes in the module). The color bar shows the significance of overlap (more intense red- more significant overlap).
(TIF)

S5 Fig Gene overlap between height GS3-Ht *preserved* modules and background modules. GS3-Ht preserved modules on y axis; background modules calculated by consensus between GS3-Ht *preserved* and GS3-Ht *removed* networks on x axis. Numbers after modules color annotations = module size (total number of genes in the module). The color bar shows the significance of overlap (more intense red- more significant overlap).
(TIF)

S6 Fig Gene overlap between height GS5-Ht *preserved* modules and background modules. GS5-Ht preserved modules on y axis; background modules calculated by consensus between GS5-Ht *preserved* and GS5 *removed* networks on x axis. Numbers after modules color annotations = module size (total number of genes in the module). The color bar shows the significance of overlap (more intense red- more significant overlap).
(TIF)

S7 Fig Overlaid histograms of gene module membership (kME) of *preserved* vs. *removed* genomic scores effects (GS3 SCZ risk) co-expression network (modules with less than 200 genes). For visualization purposes, kME histograms from modules with size less than 200 genes are plotted separately from kME from modules with size more than 200 genes.
(TIF)

S8 Fig Overlaid histograms of gene module membership (kME) of *preserved* vs. *removed* genomic scores effects (GS3 SCZ risk) co-expression network (modules with more than 200 genes). (TIF)

S9 Fig Overlaid histograms of gene module membership (kME) of *preserved* vs. *removed* genomic scores effects (GS5 SCZ risk) co-expression network (modules with less than 200 genes). (TIF)

S10 Fig Overlaid histograms of gene module membership (kME) of *preserved* vs. *removed* genomic scores effects (GS5 SCZ risk) co-expression network (modules with more than 200 genes). (TIF)

S11 Fig Overlaid histograms of gene module membership (kME) of *preserved* vs. *removed* genomic scores effects (GS3 height) co-expression network (modules with less than 200 genes). (TIF)

S12 Fig Overlaid histograms of gene module membership (kME) of *preserved* vs. *removed* genomic scores effects (GS3 height) co-expression network (modules with more than 200 genes). (TIF)

S13 Fig Overlaid histograms of gene module membership (kME) of *preserved* vs. *removed* genomic scores effects (GS5 height) co-expression network (modules with less than 200 genes). (TIF)

S14 Fig Overlaid histograms of gene module membership (kME) of *preserved* vs. *removed* genomic scores effects (GS5 height) co-expression network (modules with more than 200 genes). (TIF)

S15 Fig Heatmaps of module eigengene (MEs) correlations with traits of interest from LIBD sample (GS3 SCZ risk preserved co-expression network). SCZ risk genomic scores GS1-SCZ (p_GWAS_<5e-08)-GS6-SCZ (p_GWAS_ < .05), and height genomic scores GS1-Ht-GS6-Ht (same p_GWAS_ thresholds). Last four columns: correlations of MEs and cell type proportions to quality check the removal of variance explained by cell type proportion. Virtually no ME had correlations with cell type proportions, which confirms the efficient cell type deconvolution for neurons, astrocytes and endothelia.
(TIF)

S16 Fig Heatmaps of module eigengene (MEs) correlations with traits of interest from LIBD sample (GS5 SCZ risk preserved co-expression network). SCZ risk genomic scores GS1-SCZ (p_GWAS_<5e-08)-GS6-SCZ (p_GWAS_ < .05), and height genomic scores GS1-Ht-GS6-Ht (same p_GWAS_ thresholds). Last four columns: correlations of MEs and cell type proportions to quality check the removal of variance explained by cell type proportion. Virtually no ME had correlations with cell type proportions, which confirms the efficient cell type deconvolution for neurons, astrocytes and endothelia.
(TIF)

S17 Fig Heatmaps of module eigengene (MEs) correlations with traits of interest from LIBD sample (GS3 height preserved co-expression network). SCZ risk genomic scores GS1-SCZ (p_GWAS_<5e-08)-GS6-SCZ (p_GWAS_ < .05), and height genomic scores GS1-Ht-GS6-Ht (same p_GWAS_ thresholds). Last four columns: correlations of MEs and cell type proportions to quality check the removal of variance explained by cell type proportion. Virtually no ME had correlations with cell type proportions, which confirms the efficient cell type deconvolution for neurons, astrocytes and endothelia.
(TIF)

S18 Fig Heatmaps of module eigengene (MEs) correlations with traits of interest from LIBD sample (GS5 height preserved co-expression network). SCZ risk genomic scores GS1-SCZ (p_GWAS_<5e-08)-GS6-SCZ (p_GWAS_ < .05), and height genomic scores GS1-Ht-GS6-Ht (same p_GWAS_ thresholds). Last four columns: correlations of MEs and cell type proportions to quality check the removal of variance explained by cell type proportion. Virtually no ME had correlations with cell type proportions, which confirms the efficient cell type deconvolution for neurons, astrocytes and endothelia.
(TIF)

S19 Fig Distribution of SCZ genetic risk and height heritability in modules of preserved genomic scores effects (GS3 SCZ risk). Columns are organized by enrichment of heritability for two traits or just one; directionality of enrichment (enrichment: significant LD score >1; depletion: significant LD score<1); significance and directionality of MEs correlations with genomic scores; fragmentation of *preserved* genomics scores modules in the background (yes = fragmented; no = not fragmented); significance of enrichment in biological pathways; significance of enrichment in PGC3 loci genes. Criteria for concordant concentration of genetic burden for SCZ risk (green cells = 1 in the table) represented by significant enrichment for trait heritability, significant MEs correlations with genomic scores, directionality- MEs negatively correlated with GS-SCZ associated with neuronal functionality pathways, MEs positively correlated with GS-SCZ associated with general cellular functions; fragmentation in background; significant enrichment in PGC3 loci genes. *Legend*: modules in green cells annotated with * are fulfilling all criteria and have the highest concentration of genetic risk for SCZ (i.e, cyan for GS3-SCZ preserved network). Modules annotated with ** or in tan colored cells do not fulfill all criteria for SCZ genetic risk convergence.
(TIF)

S20 Fig Distribution of SCZ genetic risk and height heritability in modules of preserved genomic scores effects (GS5 SCZ risk). Columns are organized by enrichment of heritability for two traits or just one; directionality of enrichment (enrichment: significant LD score >1; depletion: significant LD score<1); significance and directionality of MEs correlations with genomic scores; fragmentation of *preserved* genomics scores modules in the background (yes = fragmented; no = not fragmented); significance of enrichment in biological pathways; significance of enrichment in PGC3 loci genes. Criteria for concordant concentration of genetic burden for SCZ risk (green cells = 1 in the table) represented by significant enrichment for trait heritability, significant MEs correlations with genomic scores, directionality- MEs negatively correlated with GS-SCZ associated with neuronal functionality pathways, MEs positively correlated with GS-SCZ associated with general cellular functions; fragmentation in background; significant enrichment in PGC3 loci genes. *Legend*: modules in green cells annotated with * are fulfilling all criteria and have the highest concentration of genetic risk for SCZ (i.e., black for GS5-SCZ preserved network). Modules annotated with ** or in tan colored cells do not fulfill all criteria for SCZ genetic risk convergence.
(TIF)

S21 Fig Distribution of SCZ genetic risk and height heritability in modules of preserved genomic scores effects (GS3 height). Columns are organized by enrichment of heritability for two traits or just one; directionality of enrichment (enrichment: significant LD score >1; depletion: significant LD score<1); significance and directionality of MEs correlations with genomic scores; fragmentation of *preserved* genomics scores modules in the background (yes = fragmented; no = not fragmented); significance of enrichment in biological pathways; significance of enrichment in PGC3 loci genes. Criteria for concordant concentration of genetic burden for SCZ risk (green cells = 1 in the table) represented by significant enrichment for trait heritability, significant MEs correlations with genomic scores, directionality- MEs negatively correlated with GS-SCZ associated with neuronal functionality pathways, MEs positively correlated with GS-SCZ associated with general cellular functions; fragmentation in background; significant enrichment in PGC3 loci genes. *Legend*: modules in green cells annotated with * are fulfilling all criteria and have the highest concentration of genetic risk for SCZ (i.e., black for GS3 height preserved network). Modules annotated with ** or in tan colored cells do not fulfill all criteria for SCZ genetic risk convergence.
(TIF)

S22 Fig Distribution of SCZ genetic risk and height heritability in modules of preserved genomic scores effects (GS5 height). Columns are organized by enrichment of heritability for two traits or just one; directionality of enrichment (enrichment: significant LD score >1; depletion: significant LD score<1); significance and directionality of MEs correlations with genomic scores; fragmentation of *preserved* genomics scores modules in the background (yes = fragmented; no = not fragmented); significance of enrichment in biological pathways; significance of enrichment in PGC3 loci genes. Criteria for concordant concentration of genetic burden for SCZ risk (green cells = 1 in the table) represented by significant enrichment for trait heritability, significant MEs correlations with genomic scores, directionality- MEs negatively correlated with GS-SCZ associated with neuronal functionality pathways, MEs positively correlated with GS-SCZ associated with general cellular functions; fragmentation in background; significant enrichment in PGC3 loci genes. *Legend*: modules in green cells annotated with * are fulfilling all criteria and have the highest concentration of genetic risk for SCZ (i.e., brown for GS5 height preserved network). Modules annotated with ** or in tan colored cells do not fulfill all criteria for SCZ genetic risk convergence.
(TIF)

S23 Fig Principal component analysis from gene expression of LIBD sample and replication sample (PITT); x axis- first principal component (PC1), y axis- second principal component (PC2). (TIF)

S24 Fig LIBD- PITT differences in age and RNA-Seq quality technical parameters, plotted with statistical details of between group comparisons (type “robust”) (ggstatplot package in R). (TIF)

S25 Fig LIBD- PITT differences in genomics scores of SCZ risk and height plotted with statistical details of between group comparisons (type “robust”) (ggstatplot package in R). (TIF)

S26 Fig Cluster dendrogram PITT sample. Correspondence between co-expression networks calculated after adjusting the expression data to protect/ preserve, or remove variance explained by effects of genomic scores. *Legend*: N = number of modules from each network, grey = genes not assigned to modules; prot = protected or preserved; rm = removed.
(TIF)

S27 Fig Kernel density maps- the distribution of overlap between GS-SCZ, GS-Ht preserved modules and background modules; PITT sample. Annotated modules on the x axis represent fragmented modules with weaker conservation in their background modules.
(TIF)

S28 Fig Heatmaps of module eigengene (MEs) correlations with traits of interest from PITT sample (GS3 SCZ risk preserved co-expression network). SCZ risk genomic scores GS1-SCZ (p_GWAS_<5e-08)-GS6-SCZ (p_GWAS_ < .05), and height genomic scores GS1-Ht-GS6-Ht (same p_GWAS_ thresholds). Last four columns: correlations of MEs and cell type proportions to quality check the removal of variance explained by cell type proportion. Virtually no ME had correlations with cell type proportions, which confirms the efficient cell type deconvolution for neurons, astrocytes and endothelia.
(TIF)

S29 Fig Heatmaps of module eigengene (MEs) correlations with traits of interest from PITT sample (GS5 SCZ risk preserved co-expression network). SCZ risk genomic scores GS1-SCZ (p_GWAS_<5e-08)-GS6-SCZ (p_GWAS_ < .05), and height genomic scores GS1-Ht-GS6-Ht (same p_GWAS_ thresholds). Last four columns: correlations of MEs and cell type proportions to quality check the removal of variance explained by cell type proportion. Virtually no ME had correlations with cell type proportions, which confirms the efficient cell type deconvolution for neurons, astrocytes and endothelia.
(TIF)

S30 Fig Heatmaps of module eigengene (MEs) correlations with traits of interest from PITT sample (GS3 height preserved co-expression network). SCZ risk genomic scores GS1-SCZ (p_GWAS_<5e-08)-GS6-SCZ (p_GWAS_ < .05), and height genomic scores GS1-Ht-GS6-Ht (same p_GWAS_ thresholds). Last four columns: correlations of MEs and cell type proportions to quality check the removal of variance explained by cell type proportion. Virtually no ME had correlations with cell type proportions, which confirms the efficient cell type deconvolution for neurons, astrocytes and endothelia.
(TIF)

S31 Fig Heatmaps of module eigengene (MEs) correlations with traits of interest from PITT sample (GS5 height preserved co-expression network). SCZ risk genomic scores GS1-SCZ (p_GWAS_<5e-08)-GS6-SCZ (p_GWAS_ < .05), and height genomic scores GS1-Ht-GS6-Ht (same p_GWAS_ thresholds). Last four columns: correlations of MEs and cell type proportions to quality check the removal of variance explained by cell type proportion. Virtually no ME had correlations with cell type proportions, which confirms the efficient cell type deconvolution for neurons, astrocytes and endothelia.
(TIF)

S32 Fig Functional profiling of GS-SCZ *preserved* modules with cumulative evidence for genomic risk effect on co-expression- PITT sample. Less distinct pattern of functional divergence by MEs correlation directionality, although the functional segregation of modules is conserved. Only in the GS5-SCZ *preserved* modules with negative correlations between MEs and GS-SCZ an enrichment in neuronal pathways is apparent. However, there are also modules with MEs negatively correlated with GS-SCZ and enriched in ontologies related to more general cellular processes *Legend*: BG_GS3-SCZ_ / BG_GS5-SCZ_x = fragments from SCZ risk GS3-SCZ or GS5-SCZ preserved modules overlapped with background (BG) modules.
(TIF)

S33 Fig Functional profiling of GS height *preserved* modules with cumulative evidence for genomic risk effect on co-expression; PITT sample. Relative functional divergence in biological processes enrichment like in LIBD GS preserved modules: cellular general ontologies are enriched in gene sets originated from modules with MEs positively correlated with GS-SCZs SCZ; nervous system development and functionality ontologies are enriched in gene sets originated from modules with MEs positively correlated with GS Ht. *Legend*: BG_GS3-Ht_ / BG_GS5-Ht_x = fragments from height GS3 or GS5 *preserved* modules overlapped with background (BG) modules.
(TIF)

S34 Fig LIBD and PITT samples’ modules with a relative convergence of SCZ genetic risk (on x axis: LIBD preserved GS3-SCZ cyan, GS5-SCZ black, GS3-Ht black and GS5-Ht midnightblue, and PITT preserved GS3-SCZ green, lightcyan, GS5-SCZ green, GS3-Ht mediumpurple and GS5-Ht cyan) share to an important extent biological themes related to neuronal specific functions and general cellular processes. (TIF)

S35 Fig Combined plot of correlations between GS3-SCZ and GS5-SCZ scores and marginal distributions (right panel), and plot of correlations between GS3-Ht and GS5-Ht risk scores and marginal distributions (left panel); both plots display summary statistics *robust* type. (TIF)

S36 Fig Goodness-of-fit measures of CIBERSORT deconvolution of pseudobulk RNA-Seq data (i.e., correlation coefficients and Root Mean Square Error- RMSE) show a better performance for a model based on brain scRNA-Seq signature- Velmeshev 2019 (higher correlation coefficients and lower RMSE). The algorithm fails though to estimate microglia and endothelia.
(TIF)

S37 Fig Example of one of 18,980 linear regression models fitted for MAPT gene expression. a. Summary statistics of the model; b. and c. Diagnostics plots of the model: residuals vs. fitted and normal quantile-quantile plots showing a relatively good fit for the model.
(TIF)

S38 Fig Illustration of gene expression “cleaning” effects with preservation or removal of biological signal of interest (GS3-SCZ) for two PGC3 genes, MAPT and GABBR2 in LIBD sample. Small variations are observed in the expression data in the 3 scenarios (no “cleaning”, preserved GS3-SCZ and removed GS3-SCZ) (on x axis: the 78 LIBD DLPFC samples).
(TIF)

S39 Fig Methodological pipeline to create artifact co-expression networks and background from input adjusted to preserve or remove shuffled GS SCZ and height scores. (TIF)

S1 Appendix Kernel density maps- the distribution of overlap between GS-SCZ, GS-Ht preserved modules and background modules; co-expression networks from preserved shuffled GS-SCZ and GS-Ht scores. (PDF)
