# Supplementary material for: Investigating trait variability of gene co-expression network architecture in brain by controlling for genomic risk of schizophrenia
Source: PLoS Genet. 2023 Oct 13;19(10):e1010989. doi: 10.1371/journal.pgen.1010989 (PMC10599557; doi:10.1371/journal.pgen.1010989)
Supplement: S1 Text — (DOCX) [file pgen.1010989.s001.docx]

**Supplementary Methods (SM):**

**SM 1. Postmortem brain samples and RNA-Seq processing**

*LIBD RNA-Seq*

Postmortem brain tissue was collected, dissected and processed under a protocol described in Jaffe et al (1), and Collado-Torres et al (2). The RNA pre-processing pipeline and tissue quality check also have been detailed previously^-^ (1).

RNA sequencing: total RNA was extracted from DLPFC gray matter (BA9/46) with RNeasy Lipid Tissue Mini Kit (QIAGEN) and sequencing libraries were constructed with the TruSeq Stranded Total RNA Library Preparation kit with Ribo-Zero Gold ribosomal RNA depletion.

Raw sequencing reads were quality checked with FastQC (3) and corrected with Trimmomatic (4) if necessary. The quality checked sequencing reads were mapped to the hg38/GRCh38 human reference genome with HISAT2 (v2.0.4) (5); following alignment, the expression for genes and exons was summarized in counts based on GENCODE v25 (GRCh38.p7) (6) and converted to RPKM (Reads Per Kilobase of transcript per Million mapped reads).

Genes with sufficient abundance (RPKM ≥0.1) in more than 80% of samples (N=18,980 genes) were then normalized by log_2_(x+1) transformation. Lastly, we removed the samples outlying for the standardized connectivity, which was computed by hierarchical clustering of the Euclidean distances measured from the expression data (7).

**SM 2. Calculation of quality surrogate variables for removing unwanted variance from RNA quality**

A prominent source of bias in re-constructing co-expression networks is RNA quality (i.e. technical or biological artifacts). To minimize the unwanted variance associated with this potential confounder, we implemented the quality Surrogate Variable Analysis (qSVA) approach.

Briefly, QSVA consists of identifying transcript features most susceptible to RNA degradation, respectively, estimating the so-called “degradation matrix” quantified as the coverage of the susceptible features in customized sequencing libraries and performing a principal component analysis on the “degradation matrix” that yields a number of k principal components named “quality surrogate variables” (qSVs). Quality surrogate variables (qSVs) were extracted from an RNA degradation matrix calculated as described in Jaffe et al (8).

More in detail, the degradation matrix consisted of expression measures for 1000 chromosomal regions most susceptible to RNA decay when brain tissue from 5 donors was exposed to the room temperature for various intervals of time (8)). Quality-surrogate variables computed from the degradation-matrix (“degradation principal components”- qSVs hence fort) were derived with the sva package in R, which implements a principal component analysis algorithm.

The number of qSVs was pre-determined with the num.sv function that uses the method of Buja and Eyuboglu (option method=”be” in the num.sv() function).

The quality surrogate variables (qSVs) were subsequently used in the downstream analyses for adjusting the input for co-expression network analysis.

**SM 3. Cell-type deconvolution from bulk DLPFC RNA-Seq data**

Cell type composition represents another factor that contributes to construction of co-expression networks from bulk RNA-Seq data, while modules of co-expression are likely to be significantly driven by cellular type (9). Since in this study we sought to identify more subtle changes in gene correlatability associated with the genomic risk for schizophrenia, we opted to remove the variance explained by relative cell type proportion. For this purpose, we used an adapted version of CIBERSORT (**C**ell-Type **I**dentification **b**y **E**stimating **R**elative **S**ubsets **o**f **R**NA **T**ranscripts) algorithm implemented in R (10, 11). CIBERSORT is a partial deconvolution method that uses a linear support vector regression (SVR) algorithm to estimate the cell type proportion from bulk RNA-Seq expression data by using as input a matrix of reference gene expression signatures representing each cell type of interest.

In our study, we performed CIBERSORT on our DLPFC gene expression dataset by using as reference matrix MultiBrain (11), a composite signature generated by quantile normalization of multiple datasets of cortical origin (11). The selection of MB was based on a web tool that facilitates evaluation of various reference matrices relative to the gene expression dataset of interest (in our case bulk RNA-Seq from DLPFC), through measures of goodness-of-fit (12).

Of note, deconvolution algorithms to date are still work in progress and could generate biased cell-type estimates. From this perspective, previous studies reported CIBERSORT’s lower performance in estimating glial cells (i.e., microglia) [ref 49 in main article]. Therefore, we took an additional step and performed a post-hoc evaluation of CIBERSORT performance by using pseudobulk data created from a data set of snRNA-Seq extracted from DLPFC of three donors, reported in (13), and available at <https://github.com/LieberInstitute/10xPilot_snRNAseq-human>.

The steps of this analysis were: a. quality control of the data after removing the cells with too few or too many genes; b. creating the pseudobulk data by summing counts across cells and c. data normalization with *cpm()* function from the edgeR package. We then applied the CIBERSORT algorithm on the pseudobulk data by using 2 reference brain signatures: one that we used for cell-type deconvolution of bulk RNA-Seq data (“multibrain” = MB signature), and one from the Velmeshev single-cell study (VL signature).

Results of cell-type decomposition with this algorithm indicated a similar poor performance in detecting microglia or endothelia like cell type both in the bulk and pseudobulk data (**S36 Fig**).

While these results were of concern, we appreciated that the impact on our study is rather indirect, while we used the cell-type proportion just as covariates of non-interest to adjust the expression data prior to WGCNA. Therefore, because we did not use these values for inferential purpose, we decided to keep them in our study.

**SM 4.** **GRS and genomic PC calculation**

Genomic risk scores (GRS) from two GWAS studies- PGC3 SCZ (14) and height meta-analysis (15) - were calculated as previously described (14, 16). More in detail, we obtained odds ratios of 100,101 index SNPs from meta-analysis of PGC3 and height GWAS including only European Ancestry and excluding samples from the Lieber Institute. These 101K SNPs are LD independent (R^2^<0.1) and span across the whole genome. We then calculated a weighted sum of risk alleles for schizophrenia or alleles associated with height, by summing the imputation probability for the reference allele of the index SNP, weighted by the natural log of the odds ratio of association with schizophrenia or height, at each independent locus across the whole genome, as described elsewhere (14, 16). Consistent with the original approach taken by the Psychiatric Genomics Consortium in the GWAS study (14), ten GRS (GRS1-GRS10) were calculated using sub-sets of the 101K SNPs under different thresholds of the PGC3 GWAS p-values of association with schizophrenia/ height: 5e-08, 1e-06, 1e-04, 0.001, 0.01, 0.05, 0.1, 0.2, 0.5, and 1.

To select genomic scores for further analyses, we used as criteria: 1. Magnitude of effects on gene expression variation explained by the first six sets of scores for each complex trait and 2. Collinearity diagnostics for the six genomic scores within each trait (Variation Inflation Factor- VIF calculated with *vif* function from R package *car* (17), collinearity score calculated with *colinearityScore* function from R package *variancePartition* (18, 19)). We used in this study two sets of scores for each trait, respectively the GRS-SCZ and GS-Ht that explained the highest variance of expression in our dataset and had acceptable VIF scores (≤10).

**SM 5. Variance partition analysis:**

A. For variance analysis we used the *variancePartition* R package (18, 19). The same multivariate linear (fixed effects) regression model was fit for each gene in the gene expression data set and the summary statistics were computed with the function *fitExtractVarPartModel,* including the variance fractions explained by each variable when controlling for all the other variables. As a cautionary note, while the variance explained by each variable is dependent on the other variables in the model, the results can vary considerably between studies.

For an illustration of model fit with the variance partition pipeline, we selected one of the 18,980 models used for the PGC3 prioritized gene, MAPT, and created a supplementary figure that shows: a. the summary statistic for the linear regression model, with regression coefficients and p value for each regressor; b. plot of residuals vs. fitted values and c. The quantile-quantile plot of standardized residuals (**S37 Fig**).

B. While the variables explored in the variance partition analysis were used in linear models to adjust the expression input for gene co-expression network analysis, preliminary to the adjustment step, we also evaluated the potential collinearity between all variables (including genomic scores as explained in the previous section), by using the canonical correlation matrix from *variancePartition* package) (18, 19) and the variance inflation factor (VIF) (calculated with *vif* function; *car* package) (17).

**SM 6. Data cleaning with *cleaningY* function**

Selection of this approach was based on previous studies that showed an efficient removal of batch effects, or variance explained by observed and hidden technical artifacts on gene expression, with positive consequence especially in differential gene expression analysis (ref sva paper).

The procedure was described by Jaffe et al (2015) (20) as a part of sva (surrogate variable analysis) and it was specifically developed to remove variance explained by technical artifacts (e.g., batch effects) while preserving the biological signal of interest. The necessity of applying this method came from the observation that biological signal of interest could correlate with artifacts and therefore would be removed during the step of data cleaning.

This method is implemented in *cleaningY* function available in the jaffelab R package (version 0.99.30) (21) that fits linear regression models with expression data as dependent variables and multiple variables as independent regressors and removes the variance associated with variables of non-interest (for the current study age, sex, cell-type proportion and ethnicity represented by 10 genomic PCs), hidden and observed technical confounders (quality surrogate variables (qSVs, RIN, mitochondrial mapping rate and total assigned gene rate), while retaining (“preserving”) the intercept and variables of interest (in this study, genomic risk score for SCZ or genomic score for height). In other words, retained effects are estimated but not marginalized.

The mathematical formula at the bottom of this function is the calculation of ordinary least squares (OLS) from a linear regression model with equation: y=βX + ε, where β is the vector of regression coefficients.

The (maximum likelihood) estimate of β vector is:

*β*^=(*X*′*X*)^−1^*X*′*y*

where X are the covariates in the design matrix and y is the dependent variable, in our case the matrix of ~20,000 genes x N (number of samples). If the variance explained by all variables except for the intercept, would be removed (parameter P=1 in the *cleaningY()* function), the new expression data would be equivalent to the matrix of residuals.

However, if the biological variable of interest is “preserved” (parameter P=2), only the variance explained by covariates of non-interest would be removed from the expression data.

For our specific study, residuals calculated from eight models were used as expression input sets in weighted gene co-expression network analysis (WGCNA).

Accordingly, the eight fitted models were:

1. Intercept and GRS schizophrenia “retained”;
2. Only intercept “retained” while GRS SCZ is removed with the other variables.

Equation for the above 1-2 SCZ models:

*Expression ~ β_0_ +* ***β_1_GRS_SCZ_*** *+ β_2_Age + β_3_Sex + β_4_neurons + β_5_astrocytes + β_8_endothelia + β_10_RIN + β_11_totalAssignedGene + β_12_mitoRate* + $\sum\eta_{i}snpPCs$ + $\sum\gamma_{j}qSVs$ (**Eq.1**)

1. Intercept and GS height “retained”;
2. Only intercept “protected” while GS height is removed with the other variables.

Analogous equations for the above 3-4 height models:

*Expression ~ β_0_ +* ***β_1_GS_height_*** *+ β_2_Age + β_3_Sex β_4_neurons + β_5_astrocytes + β_8_endothelia + β_10_RIN + β_11_totalAssignedGene + β_12_mitoRate* + $\sum\eta_{i}snpPCs$ + $\sum\gamma_{j}qSVs$ (**Eq.2**)

For both equations: i=10 snpPCs and j=5 qSVs.

To better visualize the results of expression “cleaning”, we select two PGC3 genes with variance more than 5% explained by GRS3 SCZ risk, respectively MAPT and GABBR2, (**S38 Fig**).

From the figure, small variations in the expression data are apparent; however, such variations potentially accumulate at the large scale of ~20,000 genes in a sufficient manner to change the genes’ connectivity and implicitly the co-expression network architecture.

In summary, we performed a required step in RNA-seq data processing, respectively removal of variance explained by covariates of non-interest, with an additional adjustment developed to preserve (“protect”) the biological signal of interest. Notably, similar approaches are described in (2, 22-23).

**SM 7. Co-expression network analysis with WGCNA**

A. For the co-expression network analysis, we used WGCNA package version 1.68 (24). Adjusted expression data were used as input for network construction. Before selecting the beta power, the outliers for standardized connectivity were removed as specified in **section 1**.

The beta power threshold was selected by analysis of scale free topology for multiple soft thresholding powers (*sft* function in WGCNA).

The networks were automatically created in a step-wise manner with functions implemented in WGCNA: 1. Calculation of adjacency matrix: correlation type = bi-weight midcorrelation; type of network = signed; power=16 selected with soft thresholding to correspond to an R^2≥0.8; 2. Calculation of topological overlap matrix (TOM) dissimilarity; 3. Detect modules by hierarchical clustering: deep split parameter=3, minimum module size=30; 4. Merging modules whose eigengenes are highly correlated for a height of cut dendrogram of 0.15.

Detected modules were labeled with pre-specified colors implemented in WGCNA routines.

B. Consensus Network Analysis:

Consensus networks between co-expression networks adjusted to retain or remove genomic scores effects were calculated according to a step-by-step protocol, as described in the online tutorial (25).

The steps of consensus network construction were:

1. Calculation of a common soft-thresholding beta power with the *sft* function in WGCNA. An estimated power of 16 appeared as suitable across all network pairs.

2. Adjacencies for all networks were created by raising the gene expression biweight correlation matrices to the power of 16 (network type: “signed”).

3. Transformation of adjacencies to Topological Overlap Matrices (TOMs) (TOM type: “signed”).

4. Scaling the TOMs to make them comparable by using the 95^th^ percentile of one network and calibrating the other to equal the reference percentile (network calibration option: “single quantile”).

5. Finally, the consensus TOM was calculated by using consensus quantile of 0 (default) of the scaled adjacencies as described in (25).

6. After calculating the consensus network, the usual hierarchical clustering algorithm was used for consensus modules detection (parameters: minimum module size=30, cut height of cluster dendrogram=0.995, merging modules with highly correlated MEs (R>0.8).

The function to calculate the consensus network was *consensusTOM* (24).

C. Cross-tabulation with *matchLabels* function (24) for visualization of co-expression networks:

This function calculates the overlap between genes present in modules from a “source” and a “reference” network by computing a Fisher’s exact test. The modules of the “source” network are then re-labeled by the “reference” modules with which they have the most overlap. The source modules without a significant overlap in the reference network are considered specific for the source network and are given distinct labels. We applied this function by taking each one network as reference and the other seven as “source” networks. Consequently, a pairwise gene overlap was performed across the modules of the eight networks and matched/ non-matched modules were identified for each network relative to the reference.

D. Relating traits to co-expression modules:

To assess the association between co-expression modules and traits of interest (i.e., genomic scores of SCZ risk and height), we first computed module eigengenes (MEs) for GS-SCZ/ GS-Ht “protected” networks and consensus networks, followed by correlations between traits an MEs. MEs are the first principal components extracted from the gene expression matrix of each individual module. They are considered representatives of gene expression profiles in a module. A positive correlation between ME and a trait of interest for a “signed” network-type, indicates that correlation between expression of genes members of the respective module and the trait would be also positive. Conversely, a negative correlation ME-trait suggests also a negative correlation between gene expression and the trait. Specifically, for our case, a positive correlation between MEs and genomic scores means that genes in the respective module follow the same pattern of increasing expression with increasing trait values, whereas a negative correlation indicates a decreasing in expression of genes members of the module with the increase of the trait (i.e., genomic score).

**SM 8. Functional convergence- Gene Ontology analysis:**

Functional enrichment of modules or gene sets of interest was performed with the *clusterProfiler,* v.4.0.5 (26, 27). Bioconductor package in R environment. Two functions were used: *enrichGO* and *compareCluster.* Both implements gene ontology over-representation calculated with a hypergeometric test by which functional terms (i.e., gene ontology sources such as biological process- GO-BP) are tested for statistically significant enrichment in gene lists of interest (26, 27). For multiple comparisons correction the FDR correction is used (26, 27). Likewise, we performed the functional enrichment by using as statistical domain the gene background represented by the total LIBD gene set (N=18,980). In our study we used only enrichment in biological processes- respectively, GO:BP annotations. We performed the functional enrichment analysis for consensus and differential modules (preserved vs. removed) genomic scores effects.

**SM 9: External validation by an independent data set:**

As an independent replication data set we used a sample selected from the NIH NeuroBioBank Brain and Tissue Repository, collected at University of Pittsburgh (henceforth named PITT sample). This data set was selected because it best approximated our original dataset, i.e. it included DLPFC RNA-Seq from 72 donors of European ancestry (28).

As a preliminary step, we performed a screening of the two sets, LIBD and PITT; the purpose was to identify potential sources for variation and reduced replicability. We started by examining the differences in demographic characteristics, genomic scores, and technical characteristics of the expression data. LIBD and PITT samples showed a statistically significant difference in age of death, and technical covariates (**S24 Fig**).

Importantly though, genomic scores of SCZ risk and height had a relatively similar pattern and distribution in the two samples and there were no differences between groups (**S25 Fig**).

Then we performed a principal component analysis on the pooled normalized gene expression from LIBD and PITT that showed a clear separation between the two data (**S23 Fig**).

To test whether the impact of GS-SCZ on gene expression is itself reproducible, we applied the same pipeline in PITT data set, by using only genes common with LIBD (N=17,767).

After testing for collinearity, the final model for gene expression adjustment in the PITT sample was:

***Expression*** *~ β_0_ +* ***β1*Genomic Score*** *+* β_2_*Age *+ β_3_*Sex + β_4_*neurons + β_5_*astrocytes + β_8_*oligodendrocytes + β_10_*RIN + β_11_*totalAssignedGene + β_12_*mitoRate* + *Σβ_i_*snpPC_i_*+ *Σβ_j_*qSV_j_*

After adjusting the expression data, we created the eight GS SCZ risk, GS-Ht *preserved* and *removed* networks by using the same WGCNA pipeline with parameters: number of genes N=17,767, beta power=10, minimum module size- 30, deep split=3. Consensus between *preserved* and *removed* networks was created also in the same manner as for LIBD data.

**SM 10. Permutations tests**

To determine the significance of overlap between gene sets of interests we used permutations tests performed with functions defined with R package *purr* (29, 30). Specifically, we used 100,000 permutations to generate the null distribution for the overlap between two gene sets with the same size as the original gene sets by randomly sampling genes without replacement from the full gene list (i.e., the group specific DLPFC set of genes plus the genes of interest absent in the DLPFC dataset), followed by computing the number of overlapping genes. To assess the statistical significance, we calculated the p values representing the number of simulated values greater than or equal to the observed values (plus one) divided by the number of iterations (plus one).

We used the permutations tests for calculating the overrepresentation of gene sets of interest (PGC3 SCZ genes and “height” GWAS genes) in individual or consensus co-expression modules.

**SM 11. Internal validation by testing the overlap between *preserved* random (shuffled) GRS modules and background.**

The pipeline for creating and testing co-expression modules from random GS3-SCZ and GS3-Ht is summarized in S39 Fig.

In step 1, gene expression input adjusted to *preserve* or *remove* 50 times shuffled GS3, respectively GS3 scores from the LIBD dataset was used to create random adjacency matrices after estimating the beta power according to the standard WGCNA pipeline. The adjacency matrices were packed in arrays of 50 matrices with functions from the *abind* R package (ref).

In step 2, average adjacencies were computed, followed by calculation of consensus (background) from the average of *preserved* and *removed* shuffled GS3 SCZ risk effects, respectively average of *preserved* or *removed* shuffled GS3 height effects. The background (consensus) was calculated with the same parameters applied to the original scores (step 3).

In step 4, full co-expression networks were calculated from the expression adjusted to *preserve* shuffled GS3-SCZ, and GS3-Ht scores effects. From these networks, 50 sets of unmerged modules per score were detected with the standard hierarchical clustering algorithm of WGCNA.

These sets of modules were tested for overlap with the corresponding background by using a Fisher’s exact test as described in **Fig 1**.

In step 5, the fragmentation pattern of *preserved* shuffled GS3-SCZ, respectively GS3-Ht sets of modules in the corresponding background was determined from density heatmaps created as described below (**SM 12 a**.).

Finally, in step 6, tests of proportions comparisons were used to compare the fragmentation pattern of *preserved* shuffled scores with that of *preserved* original scores, as explained in the Methods section.

**SM 12. Visualization of differential co-expression modules**

a. To visualize individual modules distribution in consensus modules, respectively the gene overlap between individual and consensus modules, we used density heatmaps generated with functions in ComplexHeatmap R package, by calculating kernel density estimates (31).

b. To examine module membership affinity (kME) distributions between *preserved* and *removed* modules, we introduced a preliminary condition, respectively we designated the *preserved* modules as reference for both co-expression networks, GS *preserved* and GS *removed*. Subsequently, we calculated kME relative to this reference set of modules, but we used two expression inputs: one that *preserved* the effects of GS and one that *removed* the effects of GS. In principle, this procedure can be regarded as a normalization step with two advantages: 1) ensuring the comparability of the kME and 2) maximizing the potential differences between *preserved* and *removed* kME.

After calculating the kME with the *signedKME()* function from WGCNA, we overlaid histograms of module specific *preserved* and *removed* kMEs by using functions from the *ggplot2* package (**S7-S14 Figs**). Inspection of these histograms indicate a substantial overlap of kMEs with small shifts for all networks.

**References for supplementary methods:**

1. Jaffe AE, Straub RE, Shin JH, Tao R, Gao Y, Collado-Torres L, et al (2018): Developmental and genetic regulation of the human cortex transcriptome illuminate schizophrenia pathogenesis. Nat Neurosci 21:1117-1125.

2. Collado-Torres L, Burke EE, Peterson A, Shin J, Straub RE, Rajpurohit A, et al (2019): Regional Heterogeneity in Gene Expression, Regulation, and Coherence in the Frontal Cortex and Hippocampus across Development and Schizophrenia. Neuron 103:203-216.e8.

3. Babraham Bioinformatics (2016). FastQC (Babraham Institute). <https://www.bioinformatics.babraham.ac.uk/projects/fastqc/>.

4. Bolger AM, Lohse M, Usadel B (2014): Trimmomatic: a flexible trimmer for Illumina sequence data. Bioinformatics 30:2114-2120.

5. Kim D, Paggi JM, Park C, Bennett C, Salzberg SL (2019): Graph-based genome alignment and genotyping with HISAT2 and HISAT-genotype. Nat Biotechnol 37:907-915.

6. Frankish A, Diekhans M, Ferreira AM, Johnson R, Jungreis I, Loveland J, et al (2019): GENCODE reference annotation for the human and mouse genomes. Nucleic Acids Res 47:D766-D773.

7. Horvath, S (2011). Chapter 1, pp.1-34, in Weighted Network Analysis. Applications in Systems Biology. Springer: New York, Dordrecht, Heidelberg, London.

8. Jaffe AE, Tao R, Norris AL, Kealhofer M, Nellore A, Shin JH, et al (2017): qSVA framework for RNA quality correction in differential expression analysis. Proc Natl Acad Sci U S A 114:7130-7135.

9. McKenzie AT, Wang M, Hauberg ME, Fullard JF, Kozlenkov A, Keenan A, et al. Brain cell type specific gene expression and co-expression network architectures. Sci Rep 2018; 8: 8868-5.

10. Newman AM, Liu CL, Green MR, Gentles AJ, Feng W, Xu Y, et al. Robust enumeration of cell subsets from tissue expression profiles. Nat Methods 2015; 12: 453-7.

11. Sutton GJ, Poppe D, Simmons RK, Walsh K, Nawaz U, Lister R, et al. Comprehensive evaluation of deconvolution methods for human brain gene expression. Nat Commun 2022; 13: 1358-4.

12. <https://voineagulab.shinyapps.io/BrainDeconvShiny/>

13. Tran MN, Maynard KR, Spangler A, Huuki LA, Montgomery KD, Sadashivaiah V *et al.* Single-nucleus transcriptome analysis reveals cell-type-specific molecular signatures across reward circuitry in the human brain. 2021; **109**: 3088-3103.e5.

14. Trubetskoy V, Pardinas AF, Qi T, Panagiotaropoulou G, Awasthi S, Bigdeli TB, et al. Mapping genomic loci implicates genes and synaptic biology in schizophrenia. Nature 2022; 604: 502-8.

15. Yengo L, Sidorenko J, Kemper KE, Zheng Z, Wood AR, Weedon MN, et al (2018): Meta-analysis of genome-wide association studies for height and body mass index in approximately 700000 individuals of European ancestry. Hum Mol Genet 27:3641-3649.

16. Schizophrenia Working Group of the Psychiatric Genomics C. Biological insights from 108 schizophrenia-associated genetic loci. Nature 2014; 511(7510): 421-7.

17. Fox J, Weisberg S (2019). An R Companion to Applied Regression, Third edition. Sage, Thousand Oaks CA.

18. Hoffman GE, Schadt EE (2016): variancePartition: interpreting drivers of variation in complex gene expression studies. BMC Bioinformatics 17:483-z.

19.https://www.bioconductor.org/packages/devel/bioc/vignettes/variancePartition/inst/doc/variancePartition.pdf

20. Jaffe AE, Hyde T, Kleinman J, Weinberger DR, Chenoweth JG, McKay RD *et al.* Practical impacts of genomic data ‘cleaning’ on biological discovery using surrogate variable analysis. 2015; **16**: 372-015-0808–5.

21 jaffelab package: <https://github.com/LieberInstitute/jaffelab>

22. Zandi PP, Jaffe AE, Goes FS, Burke EE, Collado-Torres L, Huuki-Myers L *et al.* Amygdala and anterior cingulate transcriptomes from individuals with bipolar disorder reveal downregulated neuroimmune and synaptic pathways. 2022; **25**: 381–389.

23. Pergola G, Parihar M, Sportelli L, Bharadwaj R, Borcuk C, Radulescu E *et al.* Consensus molecular environment of schizophrenia risk genes in coexpression networks shifting across age and brain regions. 2023; **9**: eade2812.

24. WGCNA: <https://cran.r-project.org/web/packages/WGCNA/WGCNA.pdf>

25.https://horvath.genetics.ucla.edu/html/CoexpressionNetwork/Rpackages/WGCNA/Tutorials/Consensus-NetworkConstruction-man.pdf

26. Yu G, Wang LG, Han Y, He QY (2012): clusterProfiler: an R package for comparing biological themes among gene clusters. OMICS 16:284-287.

27.https://bioconductor.org/packages/release/bioc/manuals/clusterProfiler/man/clusterProfiler.pdf

28. Hoffman GE, Bendl J, Voloudakis G, Montgomery KS, Sloofman L, Wang Y-C *et al.* CommonMind Consortium provides transcriptomic and epigenomic data for Schizophrenia and Bipolar Disorder. 2019; **6**: 180-019-0183–6.

29. https://github.com/tidyverse/purrr

30. Tutorial Biostar: <https://www.biostars.org/p/458853/>

31. <https://jokergoo.github.io/ComplexHeatmap-reference/book/>
