# Supplementary figures and images for: Investigating trait variability of gene co-expression network architecture in brain by controlling for genomic risk of schizophrenia
Source: PLoS Genet. 2023 Oct 13;19(10):e1010989. doi: 10.1371/journal.pgen.1010989 (PMC10599557; doi:10.1371/journal.pgen.1010989)

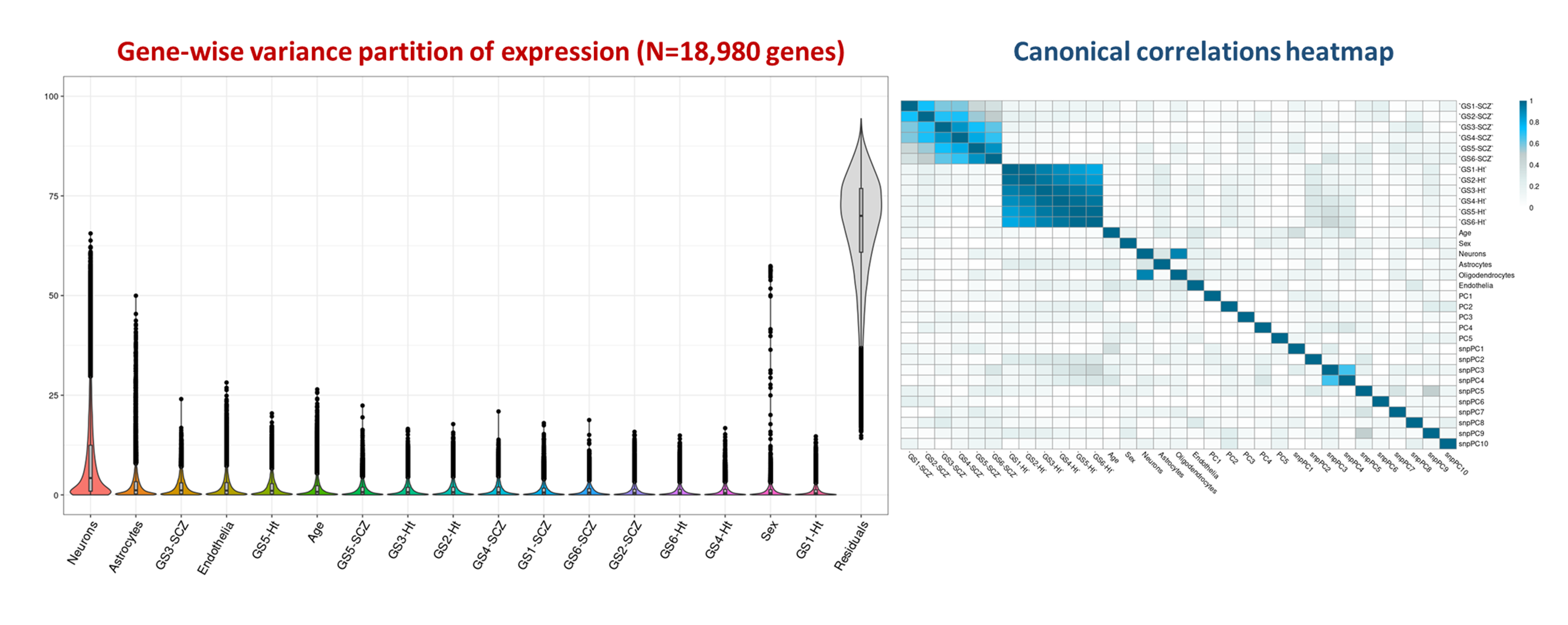

Supplement: S1 Fig — A: Variance of gene expression explained by biological variables from highest to lowest; SCZ risk GS3-SCZ (pGWAS = p<1e-04), GS5-SCZ (pGWAS = p < .01) and height GS3-Ht (pGWAS = p<1e-04), GS5-Ht (pGWAS = p < .01) are score sets with higher contribution to gene expression variability; B: Heatmap showing highest correlations between height GSs, subsets of SCZ GS-SCZs and neurons-oligodendrocytes; correlations between GS-SCZ and GS-Ht are not significant (correlation coefficients: RGS3-SCZ-GS3-Ht = .098, RGS3-SCZ-GS5-SCZ = .72, RGS3-SCZ-GS5-Ht = .034, RGS3-Ht-GS5-Ht = .94, RGS3-Ht-GS5-SCZ = .079, RGS5-SCZ-GS5-Ht = .01). (TIF) [file pgen.1010989.s011.tif]

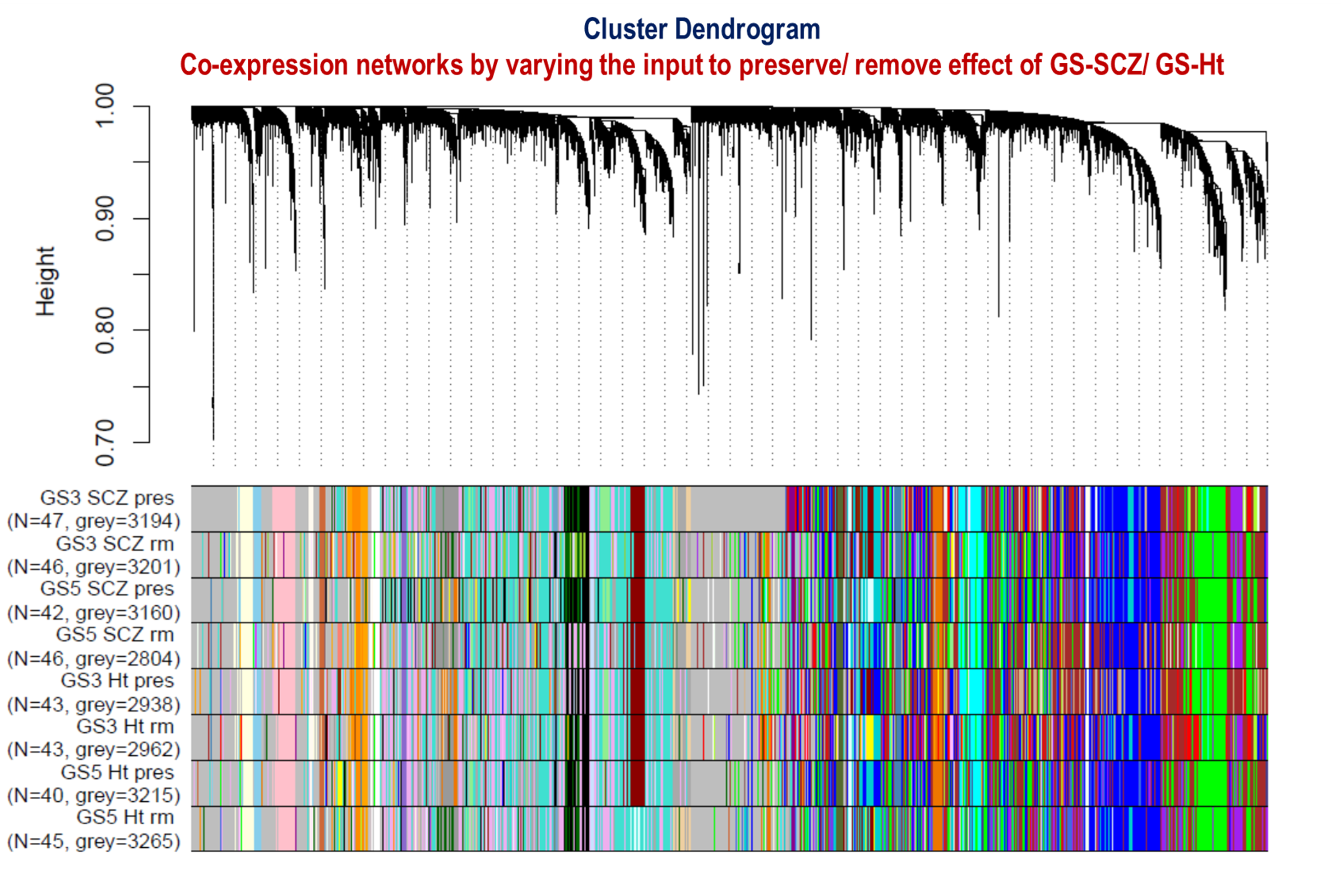

Supplement: S2 Fig — Correspondence between co-expression networks calculated after adjusting the expression data to protect/ preserve, or remove variance explained by effects of genomic scores. Legend: N = number of modules from each network, grey = genes not assigned to modules; prot = protected or preserved; rm = removed. (TIF) [file pgen.1010989.s012.tif]

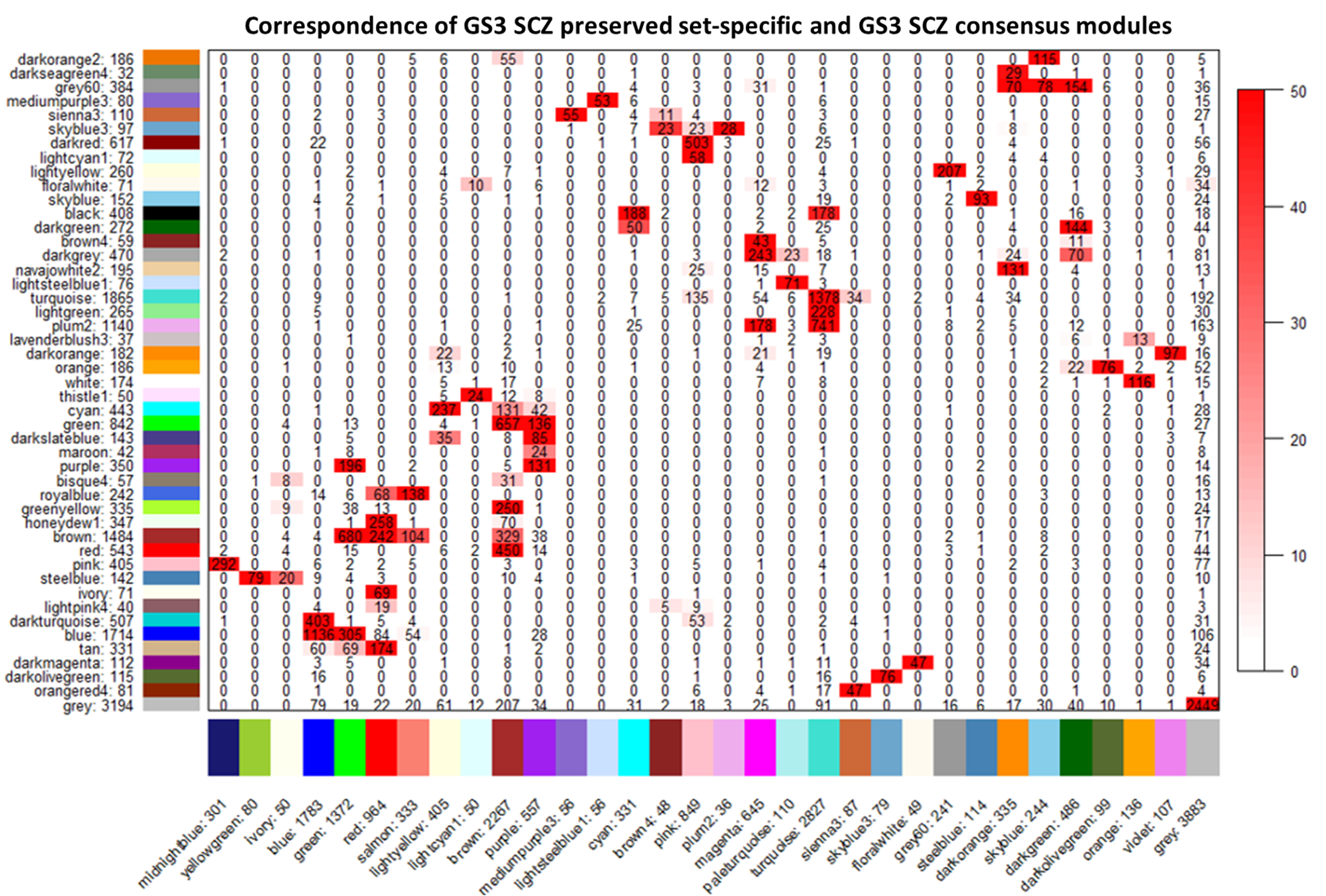

Supplement: S3 Fig — GS3-SCZ preserved modules on y axis; background modules calculated by consensus between GS3-SCZ preserved and GS3-SCZ removed networks on x axis. Numbers after modules color annotations = module size (total number of genes in the module). The color bar shows the significance of overlap (more intense red- more significant overlap). (TIF) [file pgen.1010989.s013.tif]

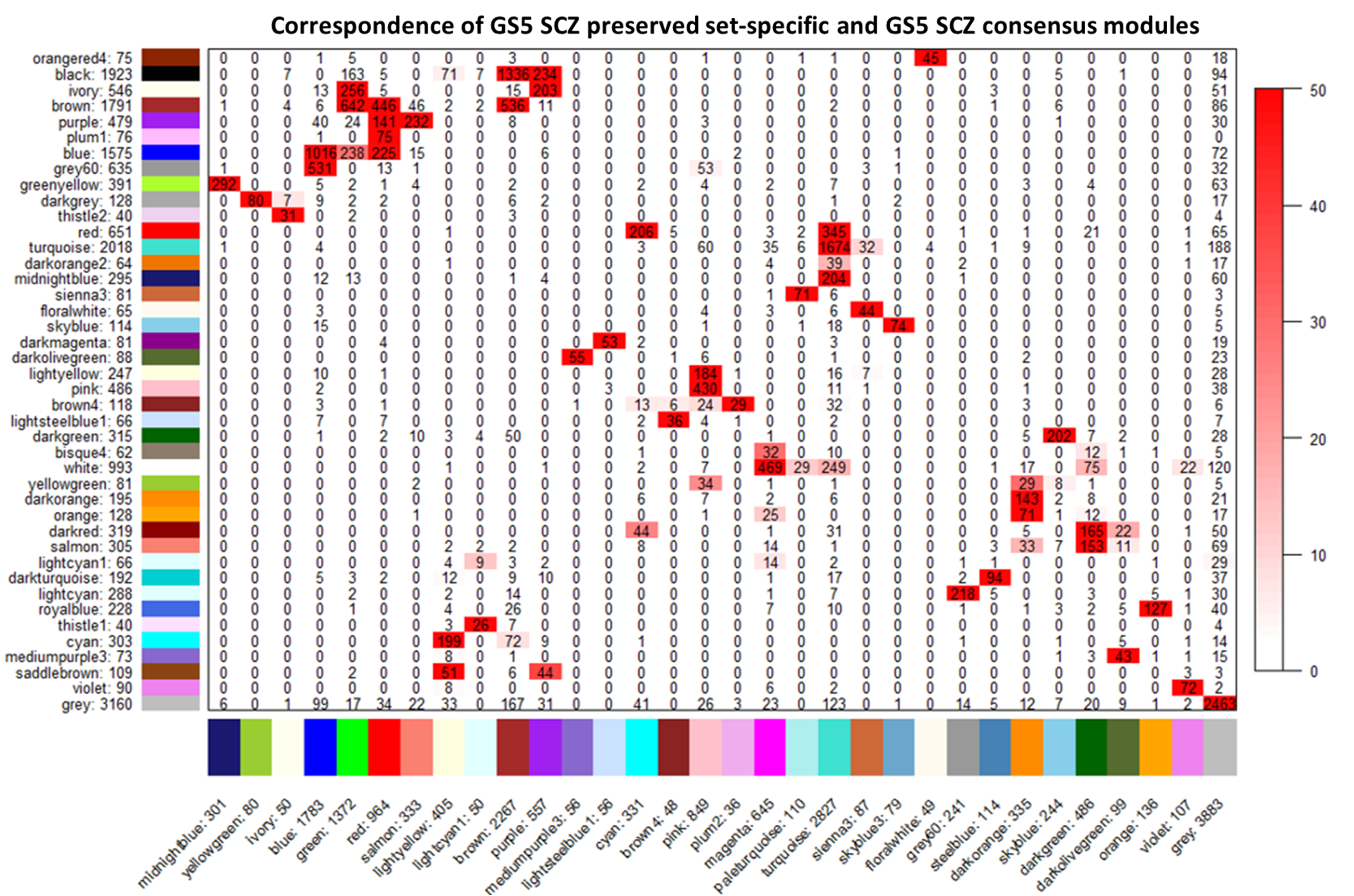

Supplement: S4 Fig — GS5-SCZ preserved modules on y axis; background modules calculated by consensus between GS5-SCZ preserved and GS5-SCZ removed networks on x axis. Numbers after modules color annotations = module size (total number of genes in the module). The color bar shows the significance of overlap (more intense red- more significant overlap). (TIF) [file pgen.1010989.s014.tif]

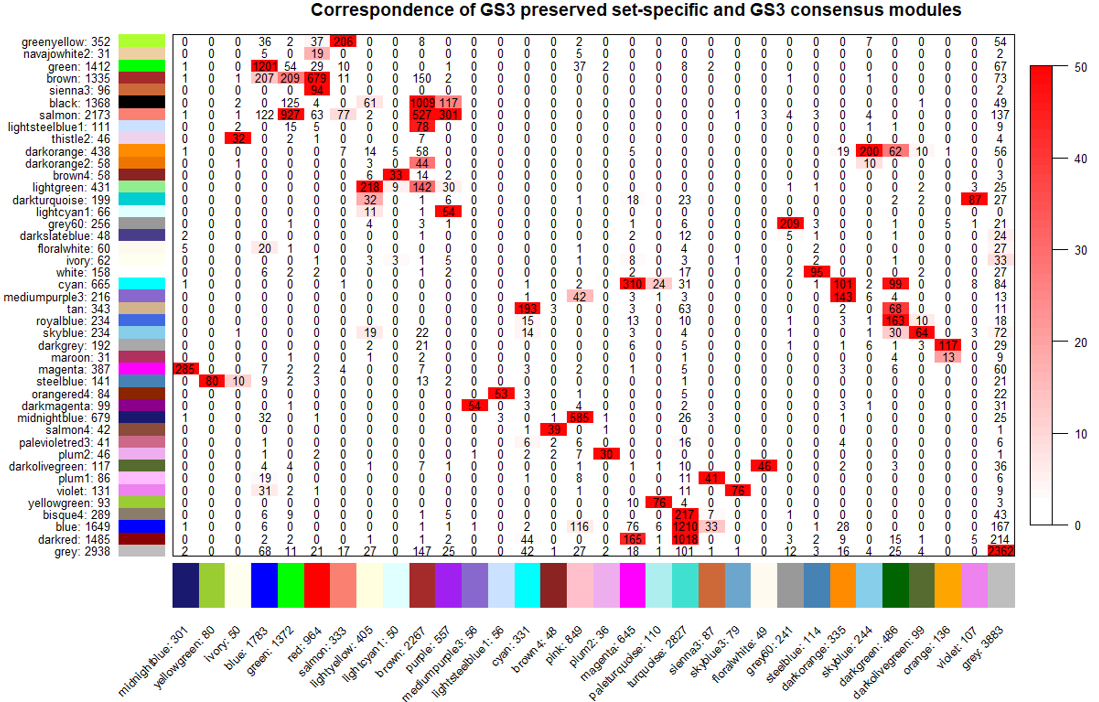

Supplement: S5 Fig — GS3-Ht preserved modules on y axis; background modules calculated by consensus between GS3-Ht preserved and GS3-Ht removed networks on x axis. Numbers after modules color annotations = module size (total number of genes in the module). The color bar shows the significance of overlap (more intense red- more significant overlap). (TIF) [file pgen.1010989.s015.tif]

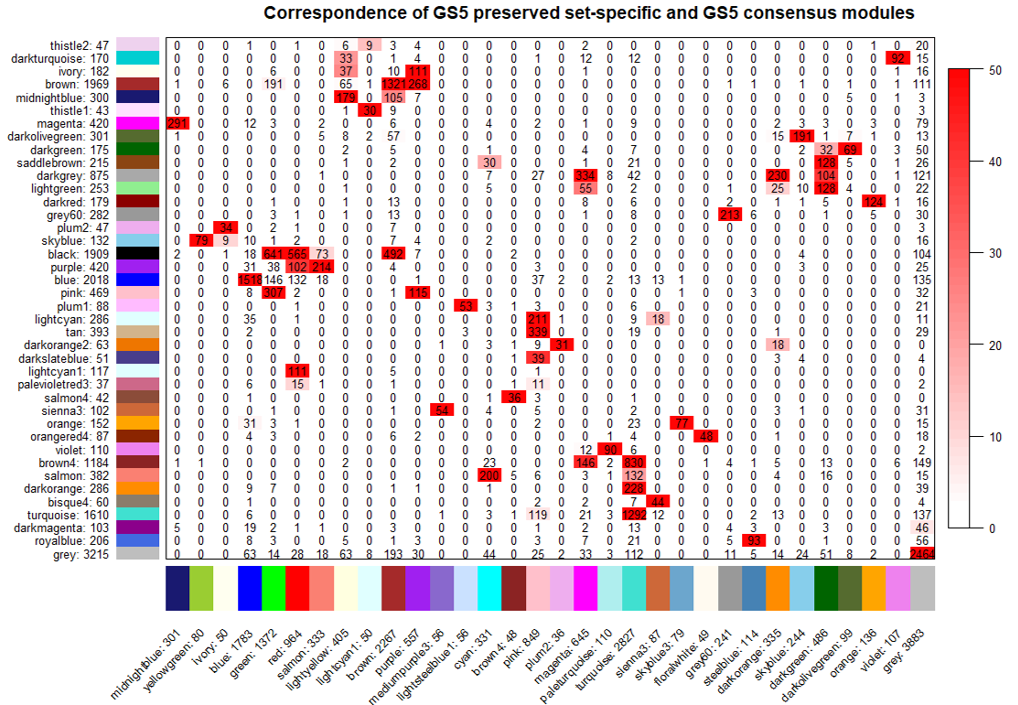

Supplement: S6 Fig — GS5-Ht preserved modules on y axis; background modules calculated by consensus between GS5-Ht preserved and GS5 removed networks on x axis. Numbers after modules color annotations = module size (total number of genes in the module). The color bar shows the significance of overlap (more intense red- more significant overlap). (TIF) [file pgen.1010989.s016.tif]

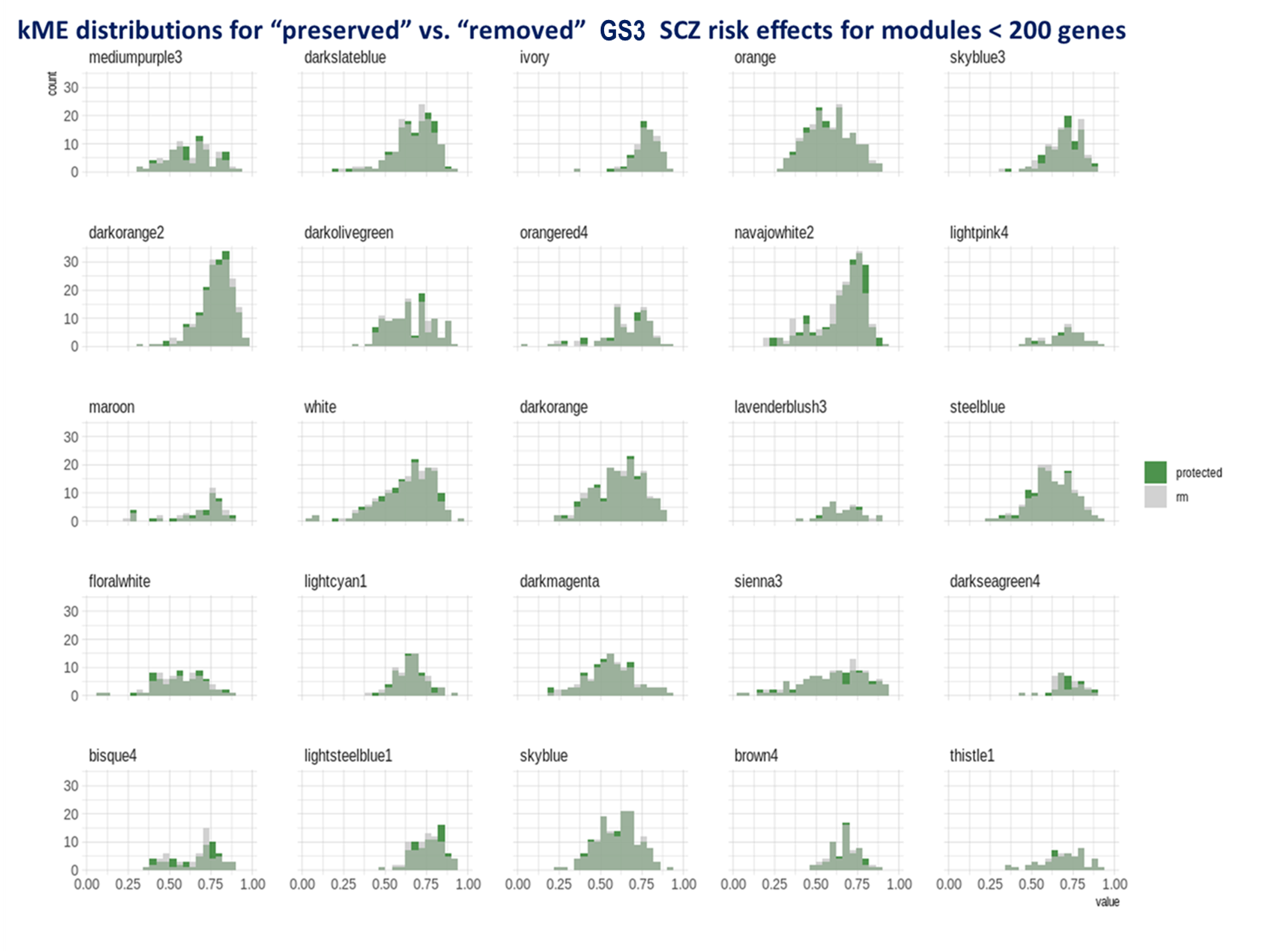

Supplement: S7 Fig — For visualization purposes, kME histograms from modules with size less than 200 genes are plotted separately from kME from modules with size more than 200 genes. (TIF) [file pgen.1010989.s017.tif]

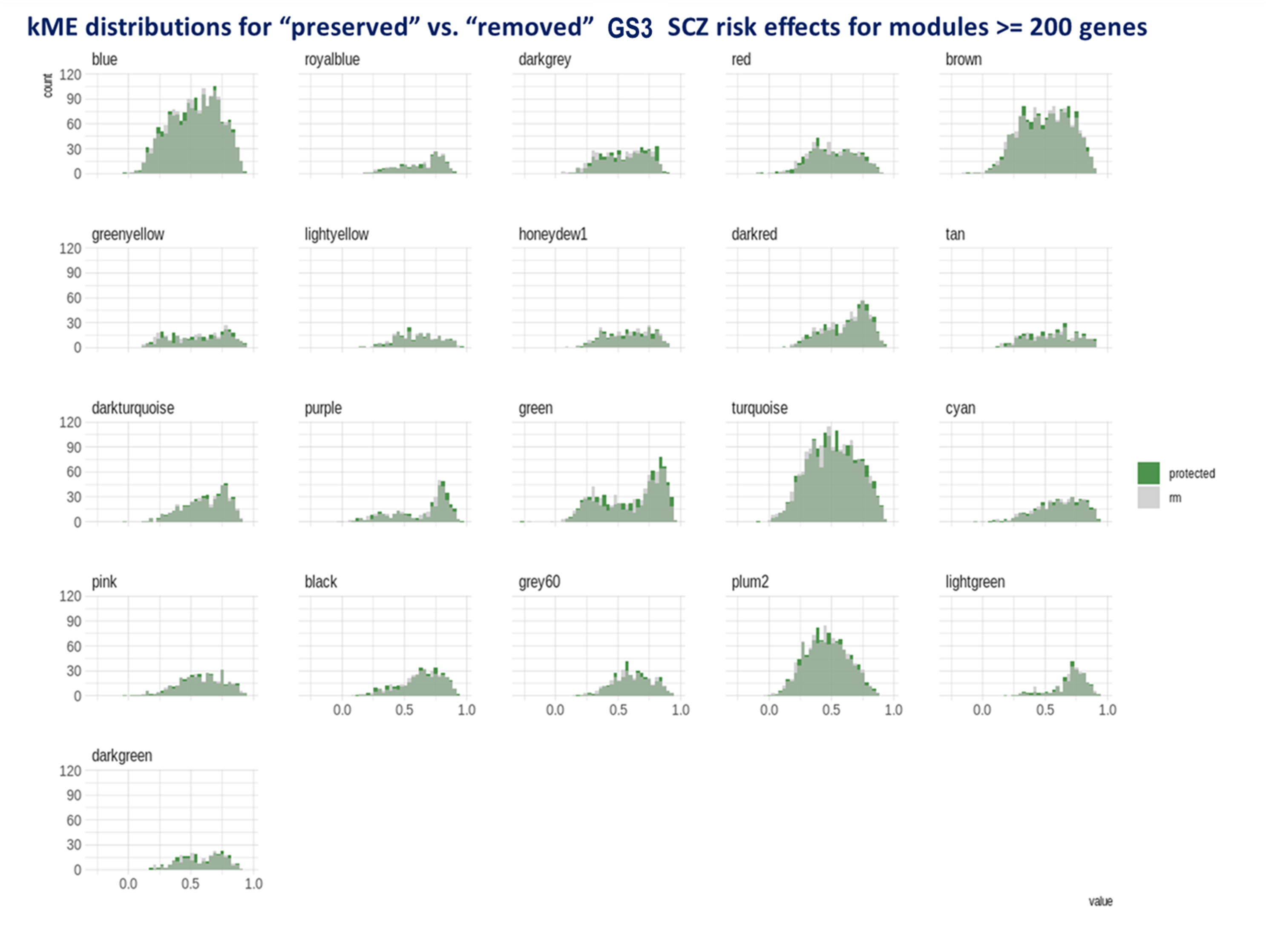

Supplement: S8 Fig — (TIF) [file pgen.1010989.s018.tif]

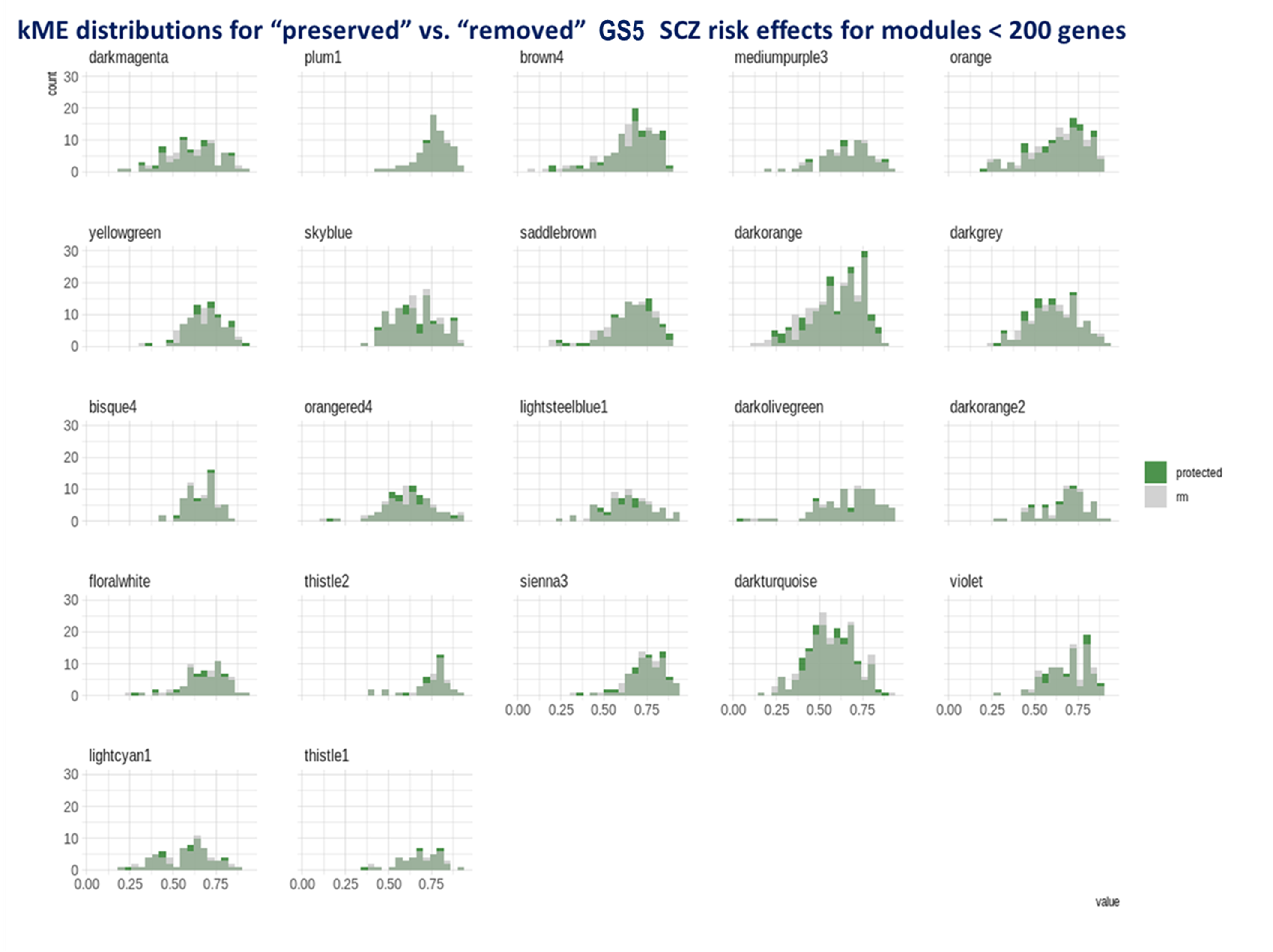

Supplement: S9 Fig — (TIF) [file pgen.1010989.s019.tif]

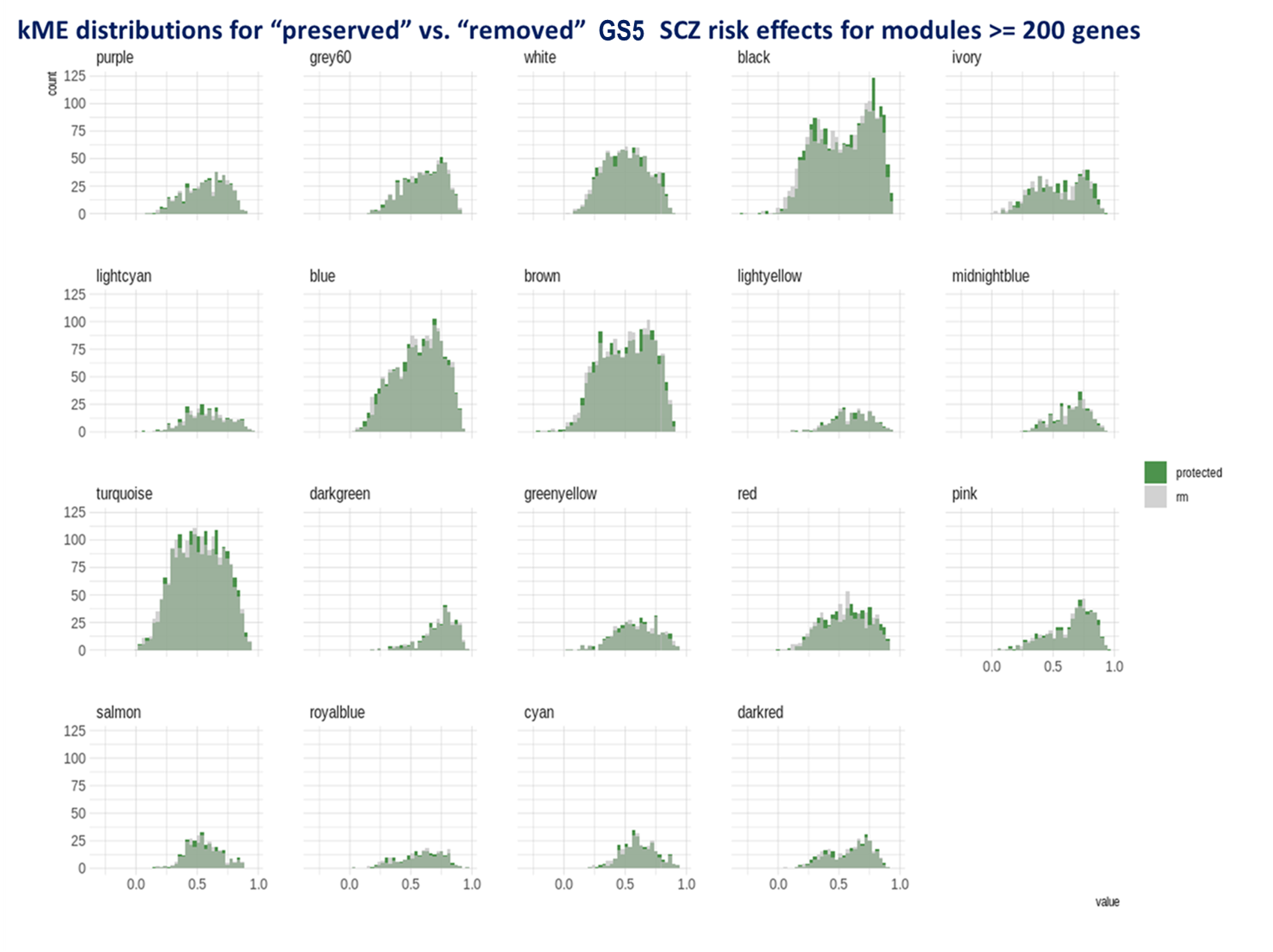

Supplement: S10 Fig — (TIF) [file pgen.1010989.s020.tif]

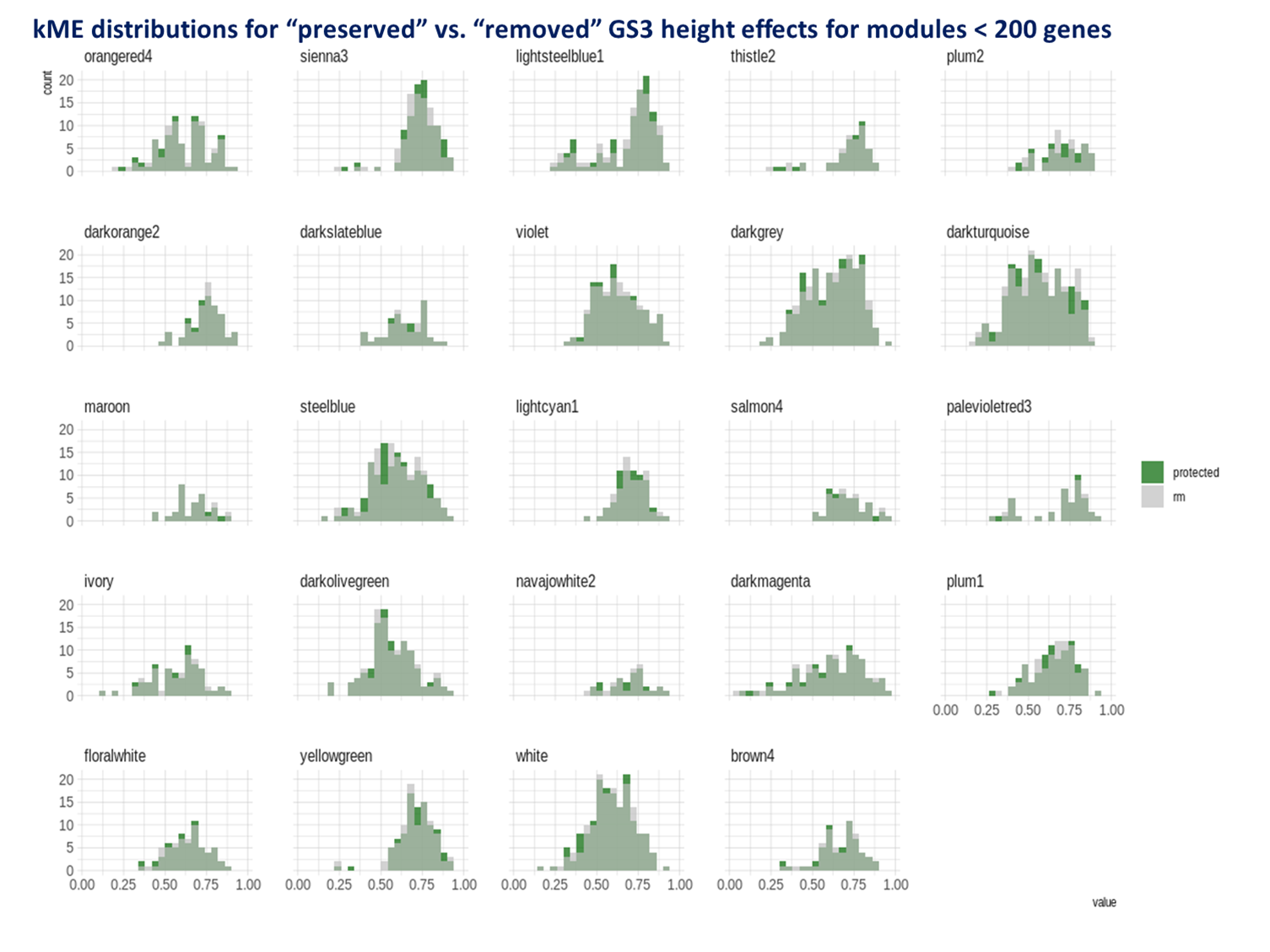

Supplement: S11 Fig — (TIF) [file pgen.1010989.s021.tif]

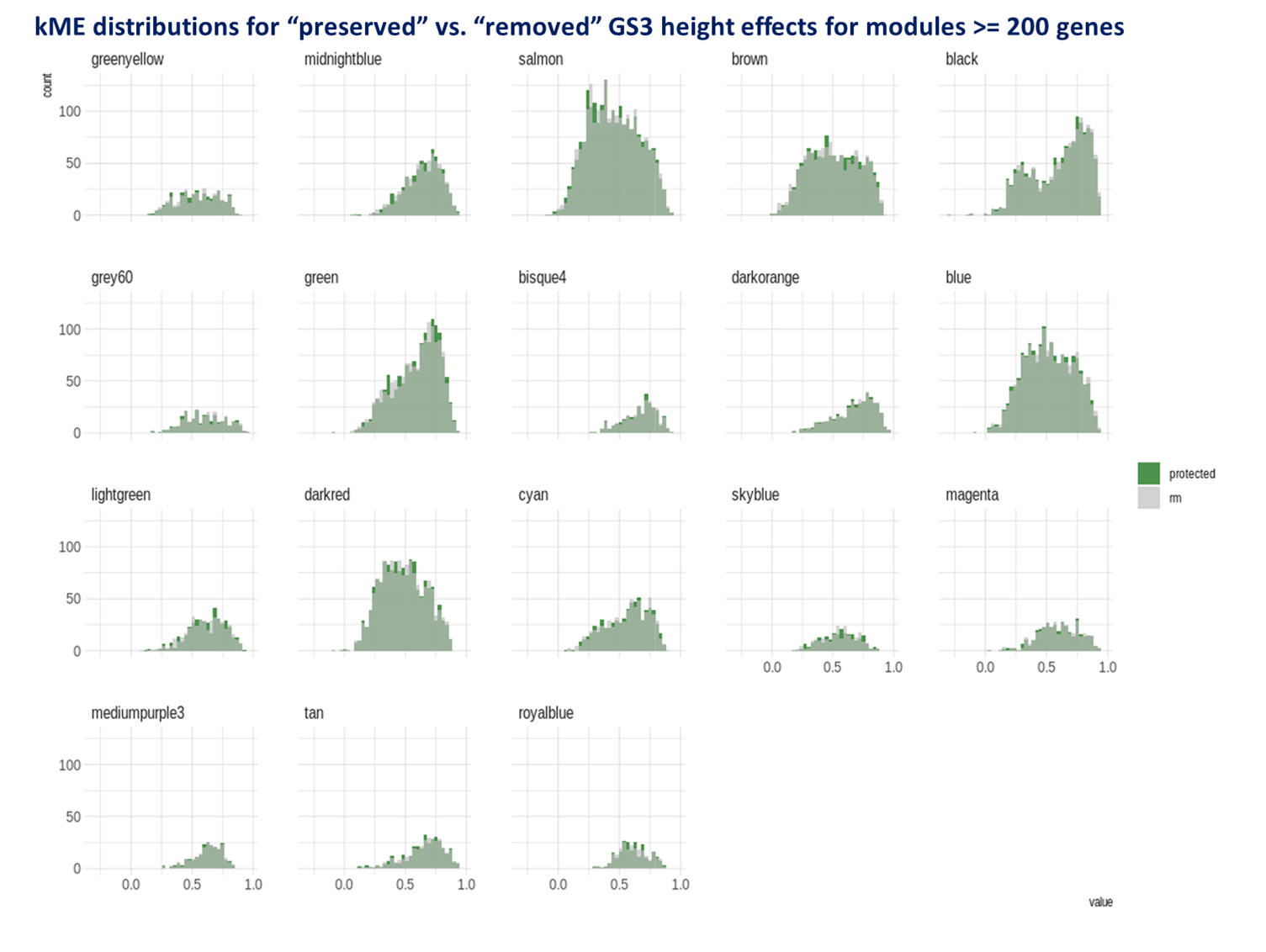

Supplement: S12 Fig — (TIF) [file pgen.1010989.s022.tif]

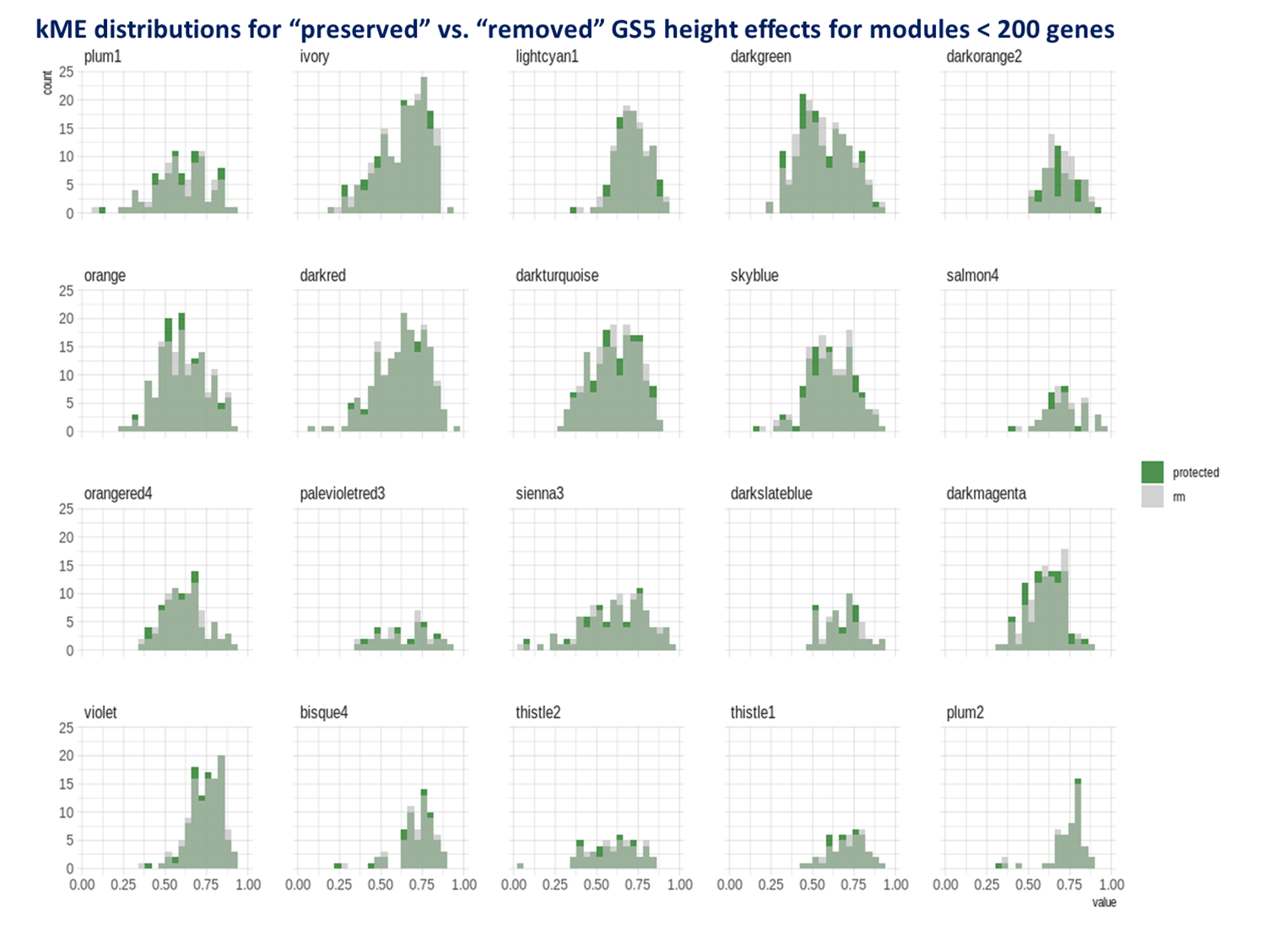

Supplement: S13 Fig — (TIF) [file pgen.1010989.s023.tif]

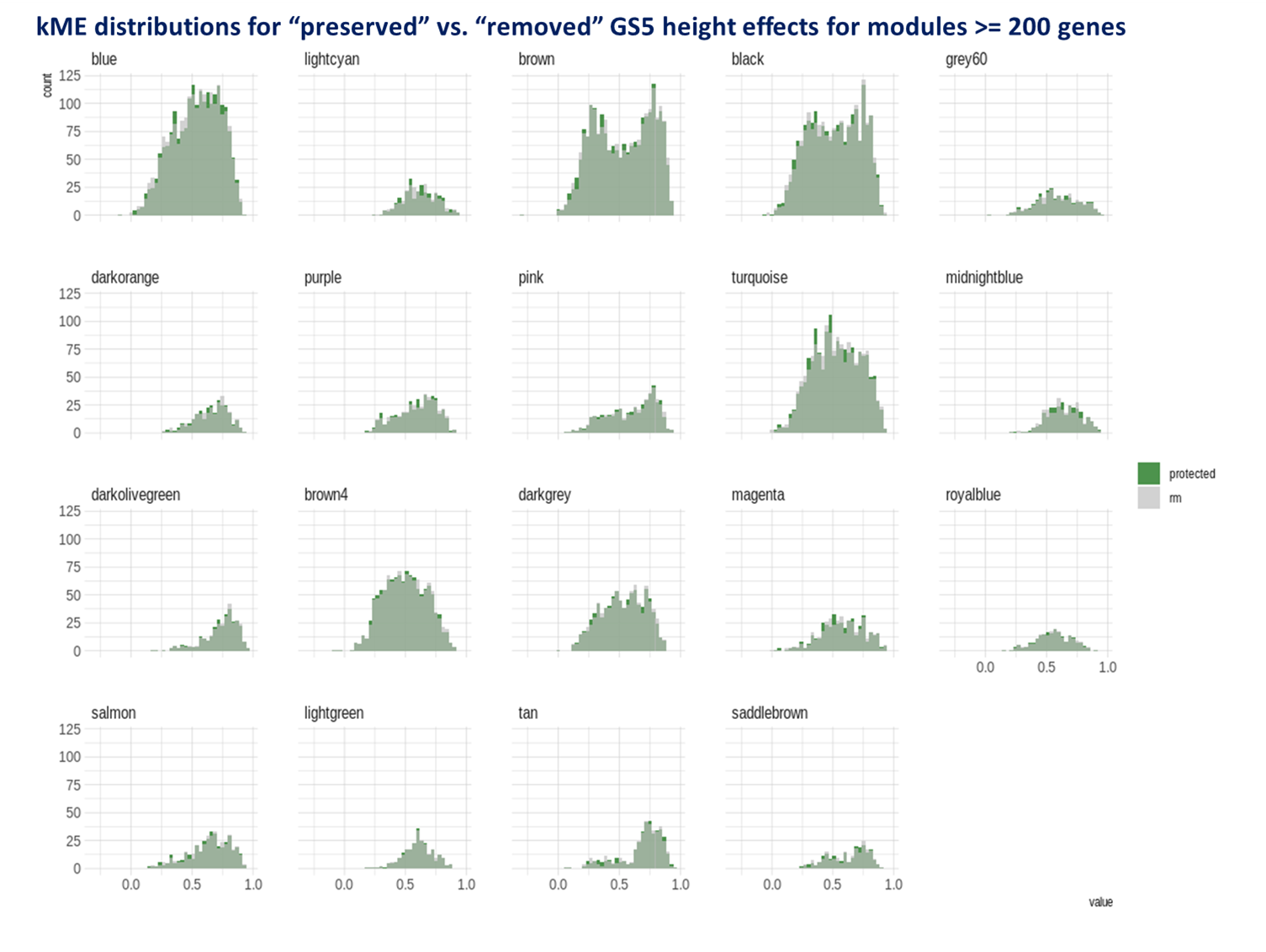

Supplement: S14 Fig — (TIF) [file pgen.1010989.s024.tif]

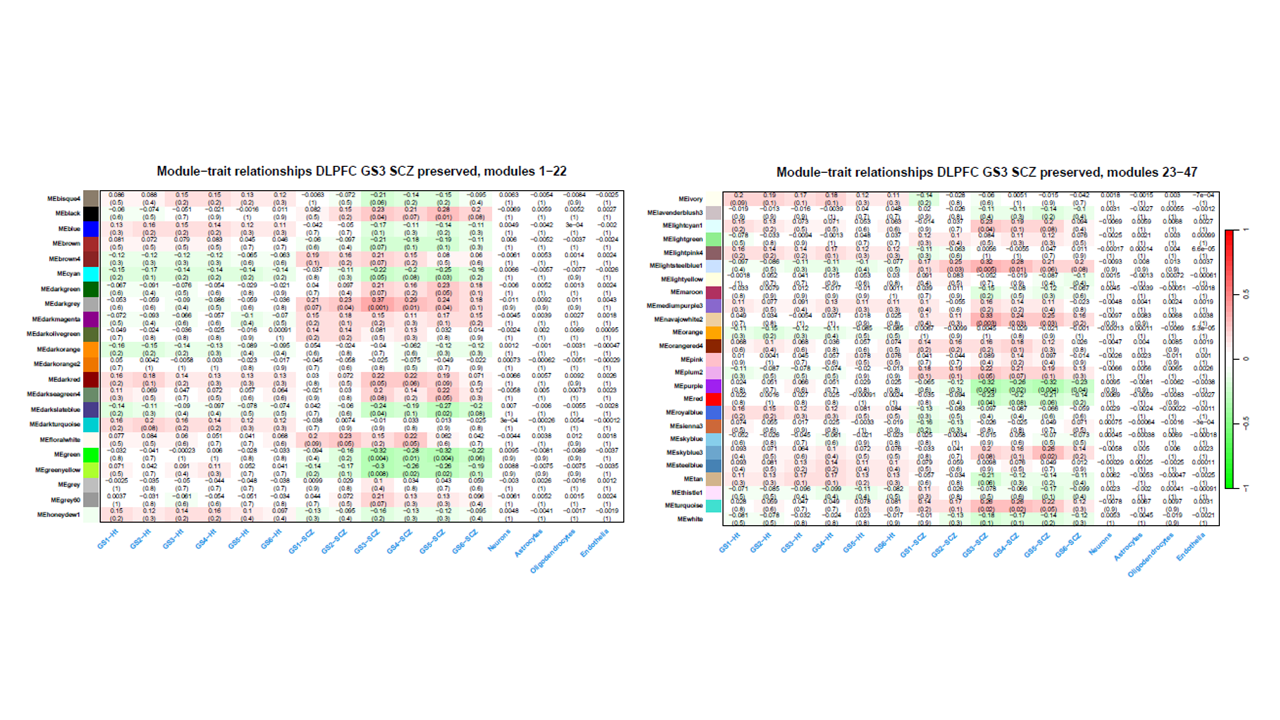

Supplement: S15 Fig — SCZ risk genomic scores GS1-SCZ (pGWAS<5e-08)-GS6-SCZ (pGWAS < .05), and height genomic scores GS1-Ht-GS6-Ht (same pGWAS thresholds). Last four columns: correlations of MEs and cell type proportions to quality check the removal of variance explained by cell type proportion. Virtually no ME had correlations with cell type proportions, which confirms the efficient cell type deconvolution for neurons, astrocytes and endothelia. (TIF) [file pgen.1010989.s025.tif]

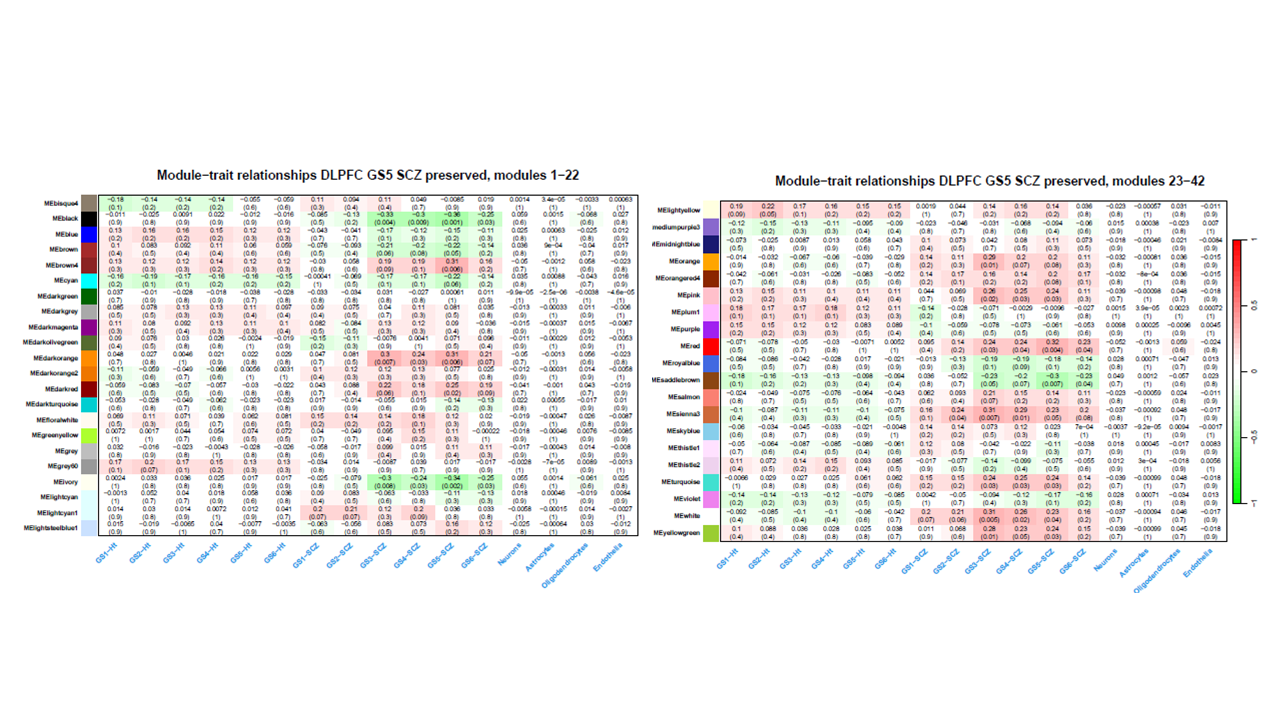

Supplement: S16 Fig — SCZ risk genomic scores GS1-SCZ (pGWAS<5e-08)-GS6-SCZ (pGWAS < .05), and height genomic scores GS1-Ht-GS6-Ht (same pGWAS thresholds). Last four columns: correlations of MEs and cell type proportions to quality check the removal of variance explained by cell type proportion. Virtually no ME had correlations with cell type proportions, which confirms the efficient cell type deconvolution for neurons, astrocytes and endothelia. (TIF) [file pgen.1010989.s026.tif]

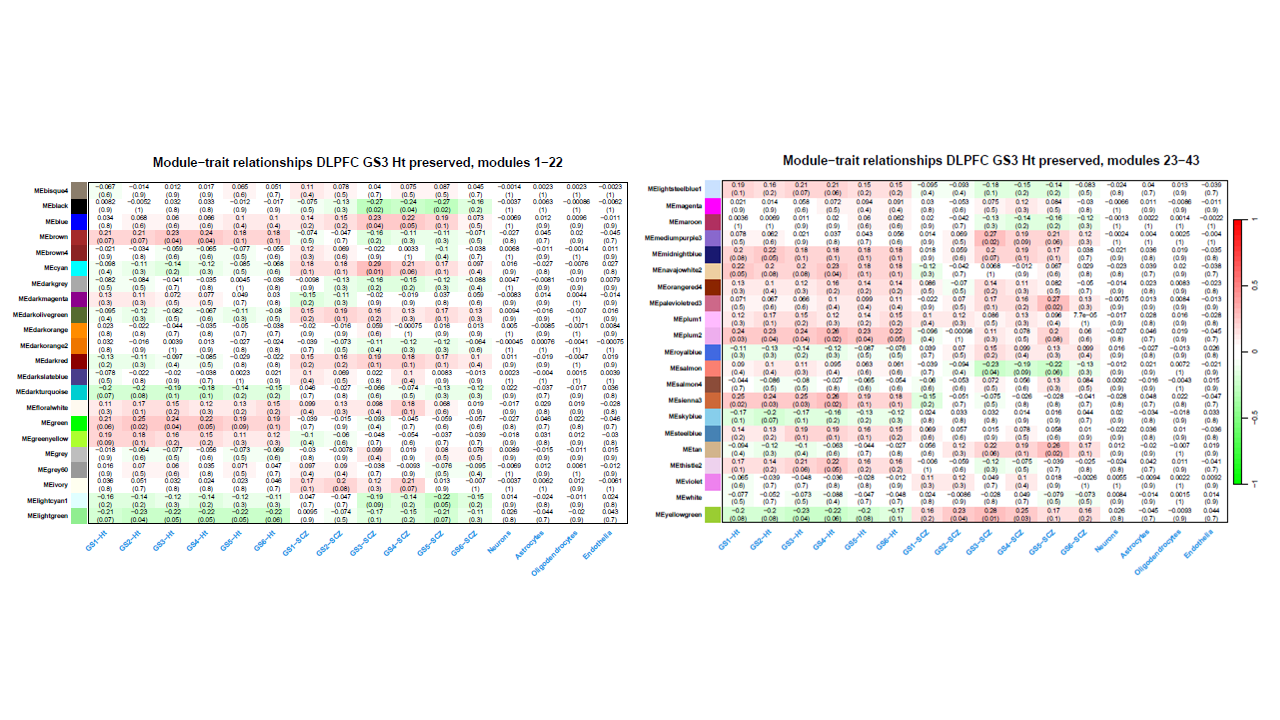

Supplement: S17 Fig — SCZ risk genomic scores GS1-SCZ (pGWAS<5e-08)-GS6-SCZ (pGWAS < .05), and height genomic scores GS1-Ht-GS6-Ht (same pGWAS thresholds). Last four columns: correlations of MEs and cell type proportions to quality check the removal of variance explained by cell type proportion. Virtually no ME had correlations with cell type proportions, which confirms the efficient cell type deconvolution for neurons, astrocytes and endothelia. (TIF) [file pgen.1010989.s027.tif]

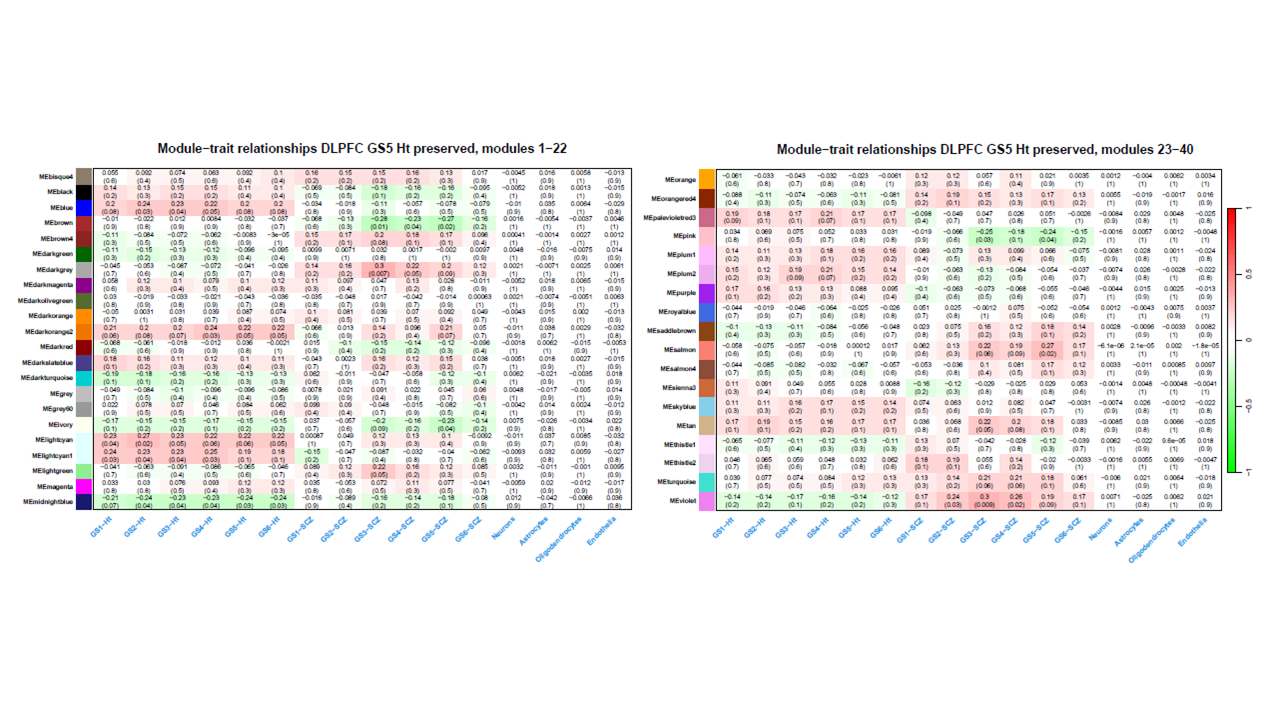

Supplement: S18 Fig — SCZ risk genomic scores GS1-SCZ (pGWAS<5e-08)-GS6-SCZ (pGWAS < .05), and height genomic scores GS1-Ht-GS6-Ht (same pGWAS thresholds). Last four columns: correlations of MEs and cell type proportions to quality check the removal of variance explained by cell type proportion. Virtually no ME had correlations with cell type proportions, which confirms the efficient cell type deconvolution for neurons, astrocytes and endothelia. (TIF) [file pgen.1010989.s028.tif]

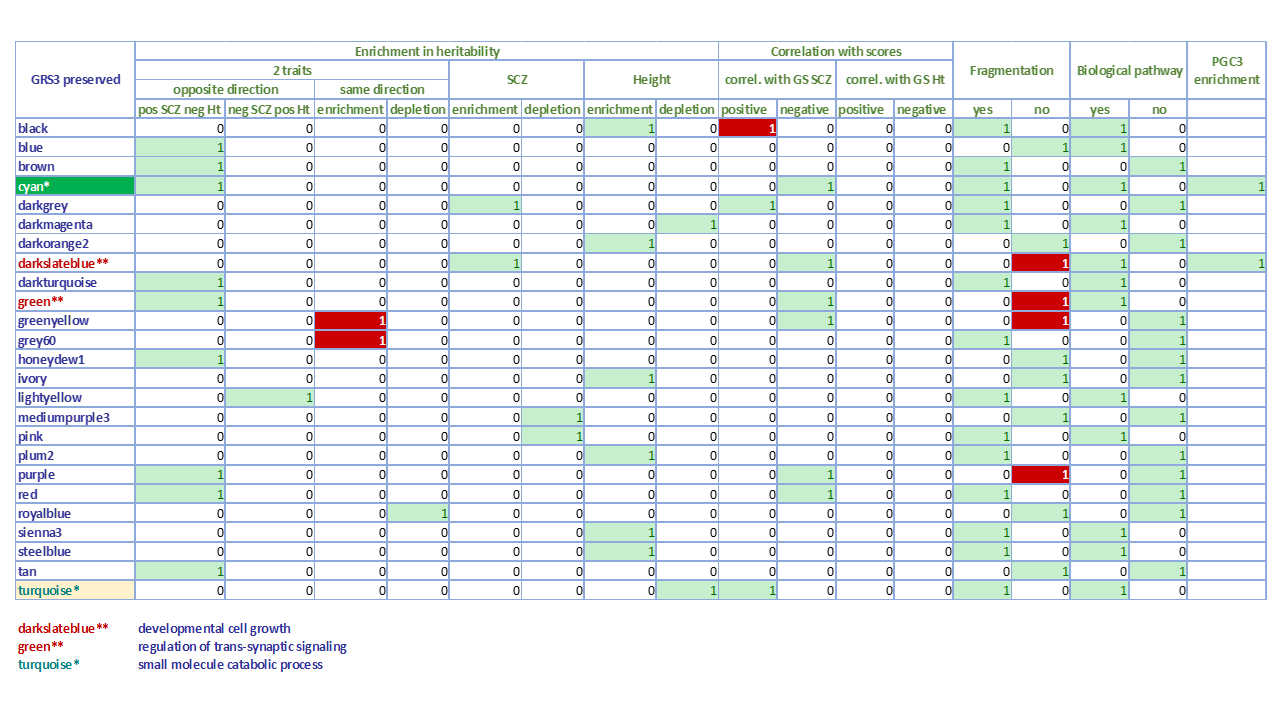

Supplement: S19 Fig — Columns are organized by enrichment of heritability for two traits or just one; directionality of enrichment (enrichment: significant LD score >1; depletion: significant LD score<1); significance and directionality of MEs correlations with genomic scores; fragmentation of preserved genomics scores modules in the background (yes = fragmented; no = not fragmented); significance of enrichment in biological pathways; significance of enrichment in PGC3 loci genes. Criteria for concordant concentration of genetic burden for SCZ risk (green cells = 1 in the table) represented by significant enrichment for trait heritability, significant MEs correlations with genomic scores, directionality- MEs negatively correlated with GS-SCZ associated with neuronal functionality pathways, MEs positively correlated with GS-SCZ associated with general cellular functions; fragmentation in background; significant enrichment in PGC3 loci genes. Legend: modules in green cells annotated with * are fulfilling all criteria and have the highest concentration of genetic risk for SCZ (i.e, cyan for GS3-SCZ preserved network). Modules annotated with ** or in tan colored cells do not fulfill all criteria for SCZ genetic risk convergence. (TIF) [file pgen.1010989.s029.tif]

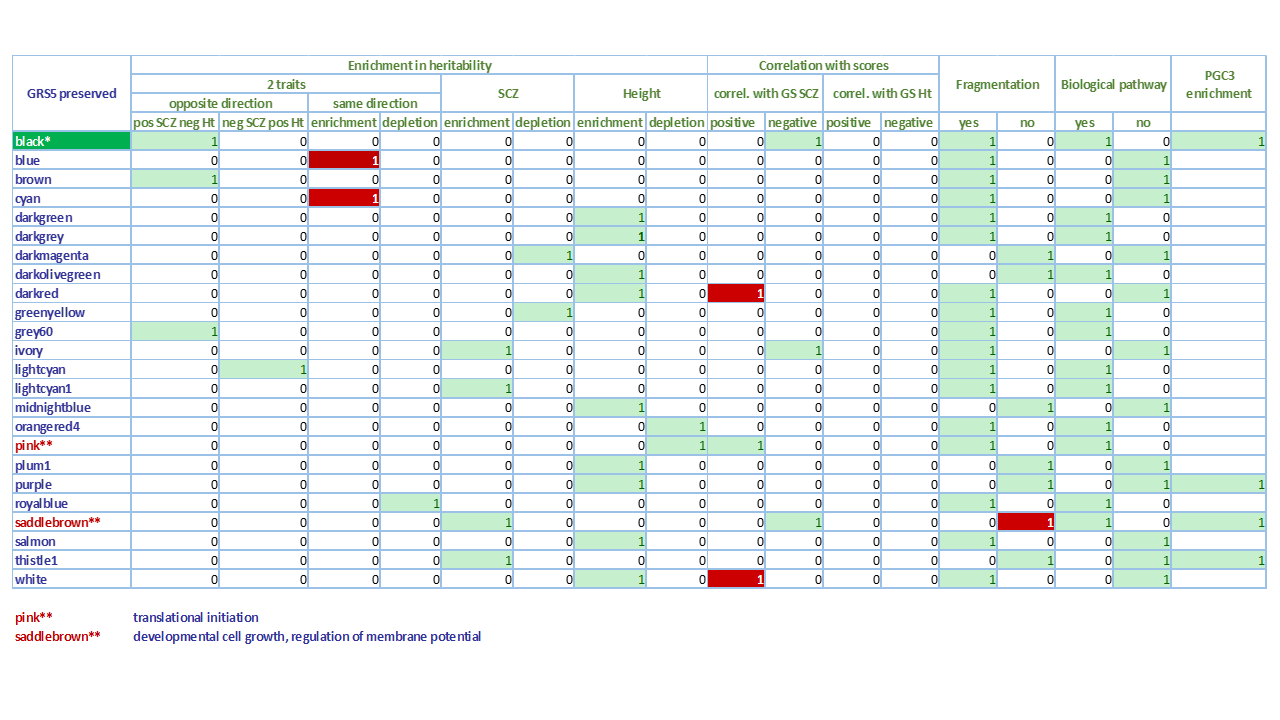

Supplement: S20 Fig — Columns are organized by enrichment of heritability for two traits or just one; directionality of enrichment (enrichment: significant LD score >1; depletion: significant LD score<1); significance and directionality of MEs correlations with genomic scores; fragmentation of preserved genomics scores modules in the background (yes = fragmented; no = not fragmented); significance of enrichment in biological pathways; significance of enrichment in PGC3 loci genes. Criteria for concordant concentration of genetic burden for SCZ risk (green cells = 1 in the table) represented by significant enrichment for trait heritability, significant MEs correlations with genomic scores, directionality- MEs negatively correlated with GS-SCZ associated with neuronal functionality pathways, MEs positively correlated with GS-SCZ associated with general cellular functions; fragmentation in background; significant enrichment in PGC3 loci genes. Legend: modules in green cells annotated with * are fulfilling all criteria and have the highest concentration of genetic risk for SCZ (i.e., black for GS5-SCZ preserved network). Modules annotated with ** or in tan colored cells do not fulfill all criteria for SCZ genetic risk convergence. (TIF) [file pgen.1010989.s030.tif]

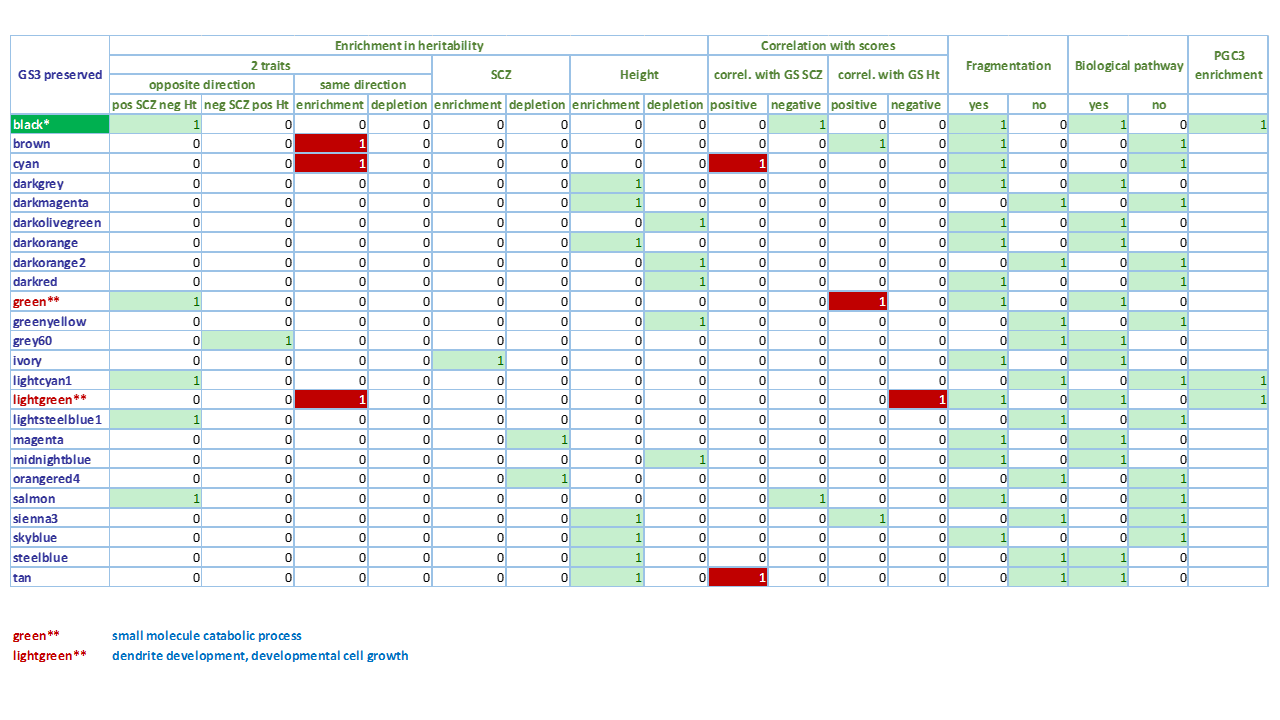

Supplement: S21 Fig — Columns are organized by enrichment of heritability for two traits or just one; directionality of enrichment (enrichment: significant LD score >1; depletion: significant LD score<1); significance and directionality of MEs correlations with genomic scores; fragmentation of preserved genomics scores modules in the background (yes = fragmented; no = not fragmented); significance of enrichment in biological pathways; significance of enrichment in PGC3 loci genes. Criteria for concordant concentration of genetic burden for SCZ risk (green cells = 1 in the table) represented by significant enrichment for trait heritability, significant MEs correlations with genomic scores, directionality- MEs negatively correlated with GS-SCZ associated with neuronal functionality pathways, MEs positively correlated with GS-SCZ associated with general cellular functions; fragmentation in background; significant enrichment in PGC3 loci genes. Legend: modules in green cells annotated with * are fulfilling all criteria and have the highest concentration of genetic risk for SCZ (i.e., black for GS3 height preserved network). Modules annotated with ** or in tan colored cells do not fulfill all criteria for SCZ genetic risk convergence. (TIF) [file pgen.1010989.s031.tif]

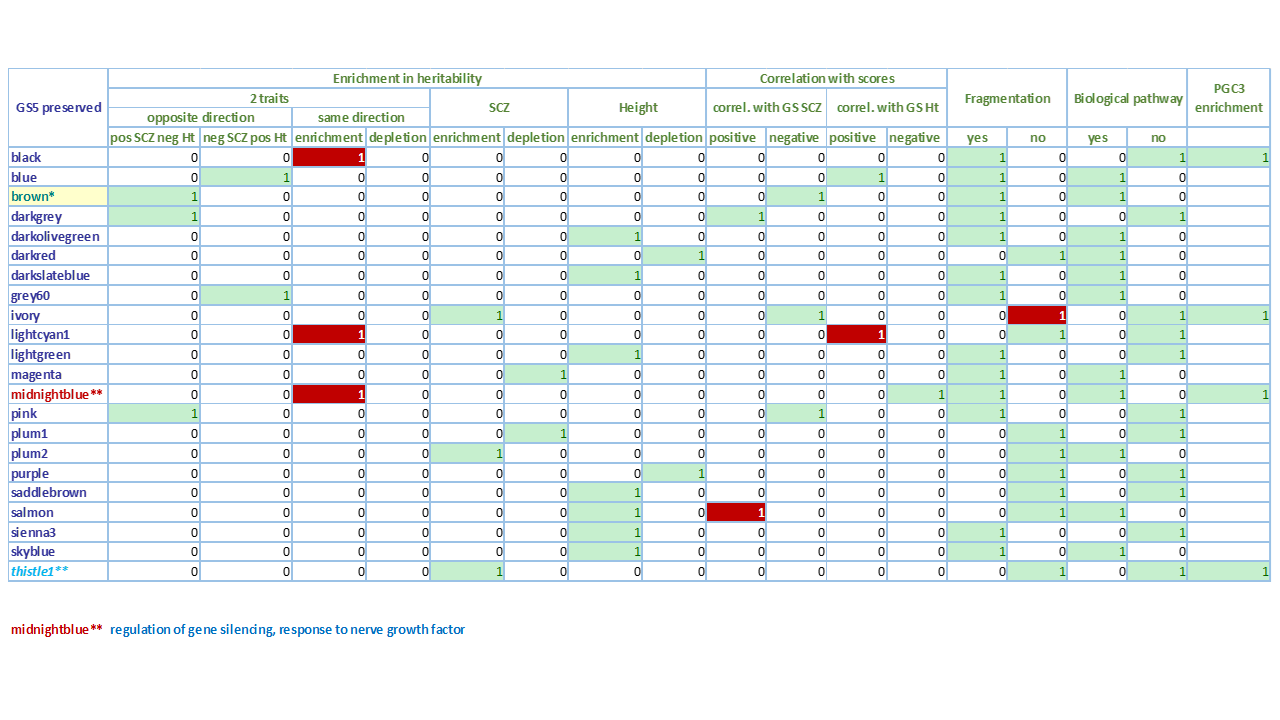

Supplement: S22 Fig — Columns are organized by enrichment of heritability for two traits or just one; directionality of enrichment (enrichment: significant LD score >1; depletion: significant LD score<1); significance and directionality of MEs correlations with genomic scores; fragmentation of preserved genomics scores modules in the background (yes = fragmented; no = not fragmented); significance of enrichment in biological pathways; significance of enrichment in PGC3 loci genes. Criteria for concordant concentration of genetic burden for SCZ risk (green cells = 1 in the table) represented by significant enrichment for trait heritability, significant MEs correlations with genomic scores, directionality- MEs negatively correlated with GS-SCZ associated with neuronal functionality pathways, MEs positively correlated with GS-SCZ associated with general cellular functions; fragmentation in background; significant enrichment in PGC3 loci genes. Legend: modules in green cells annotated with * are fulfilling all criteria and have the highest concentration of genetic risk for SCZ (i.e., brown for GS5 height preserved network). Modules annotated with ** or in tan colored cells do not fulfill all criteria for SCZ genetic risk convergence. (TIF) [file pgen.1010989.s032.tif]

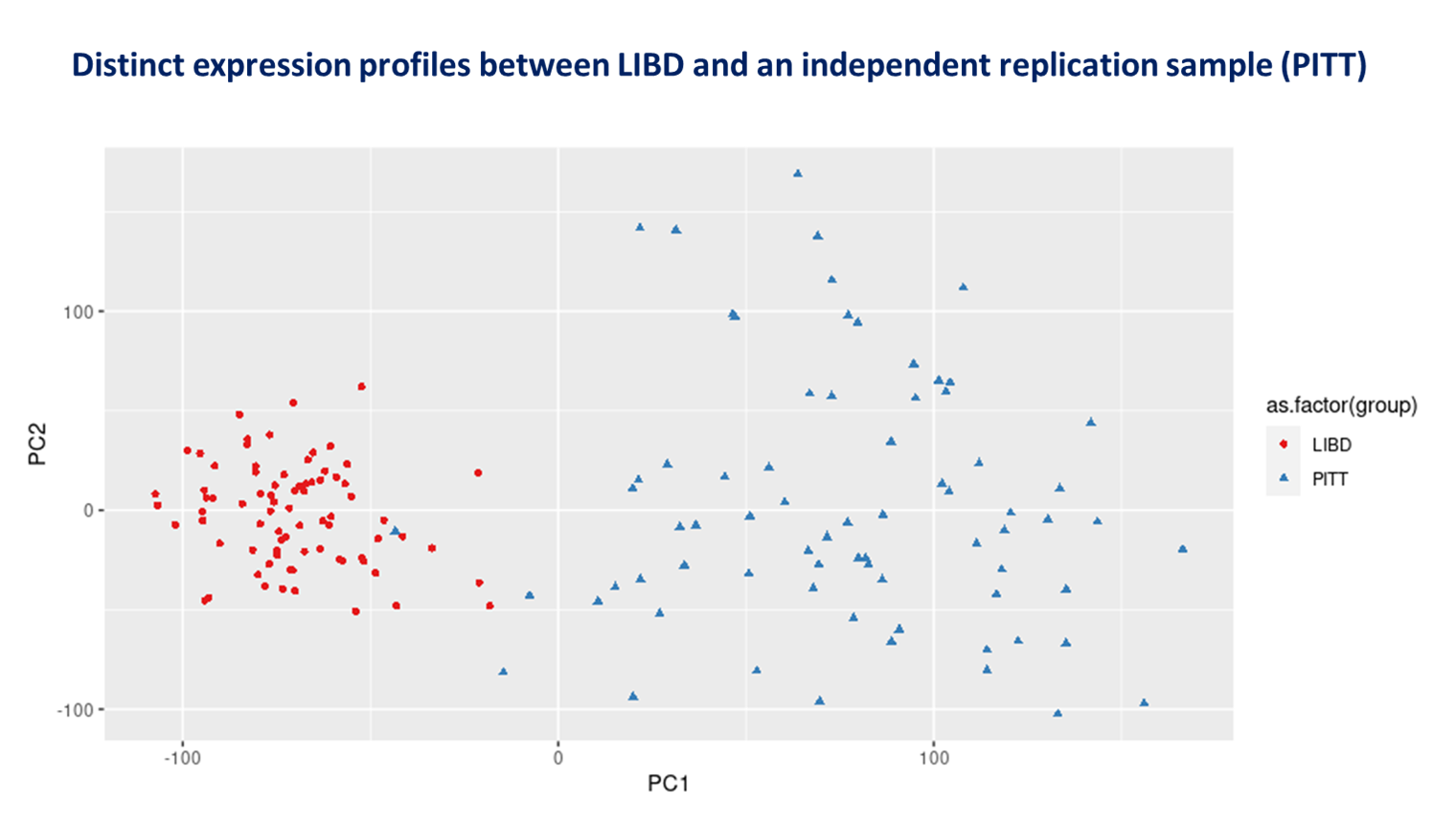

Supplement: S23 Fig — (TIF) [file pgen.1010989.s033.tif]

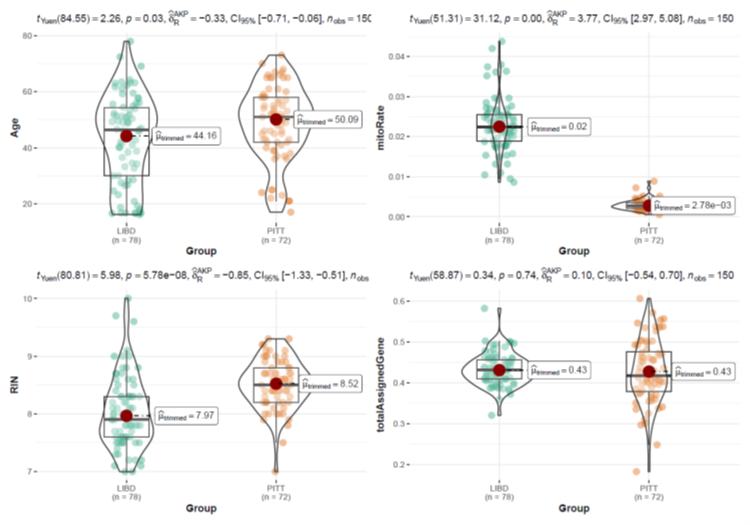

Supplement: S24 Fig — (TIF) [file pgen.1010989.s034.tif]

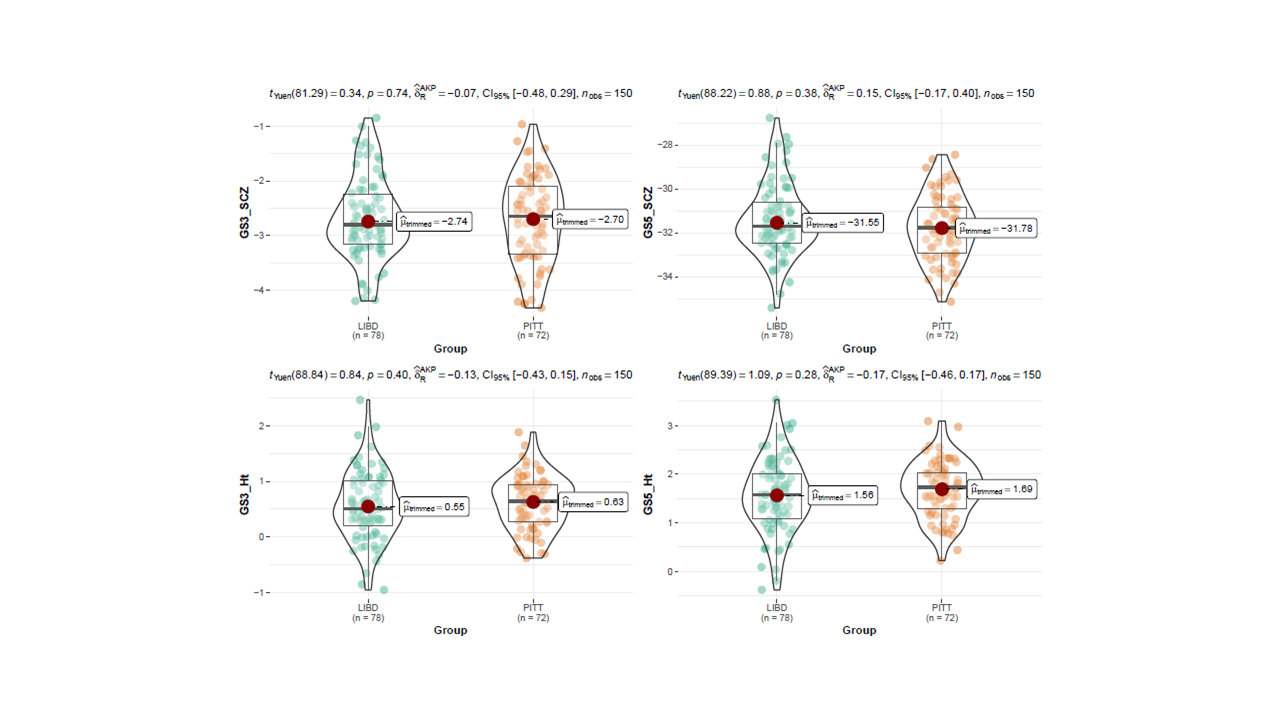

Supplement: S25 Fig — (TIF) [file pgen.1010989.s035.tif]

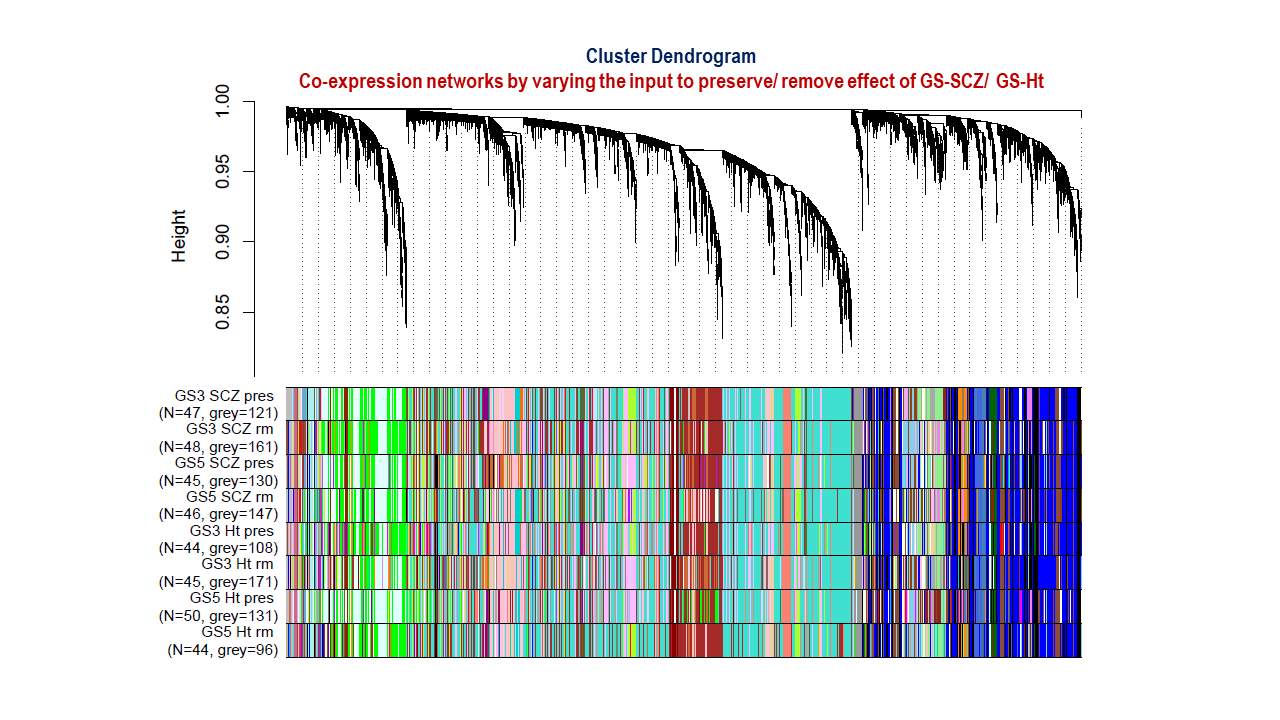

Supplement: S26 Fig — Correspondence between co-expression networks calculated after adjusting the expression data to protect/ preserve, or remove variance explained by effects of genomic scores. Legend: N = number of modules from each network, grey = genes not assigned to modules; prot = protected or preserved; rm = removed. (TIF) [file pgen.1010989.s036.tif]

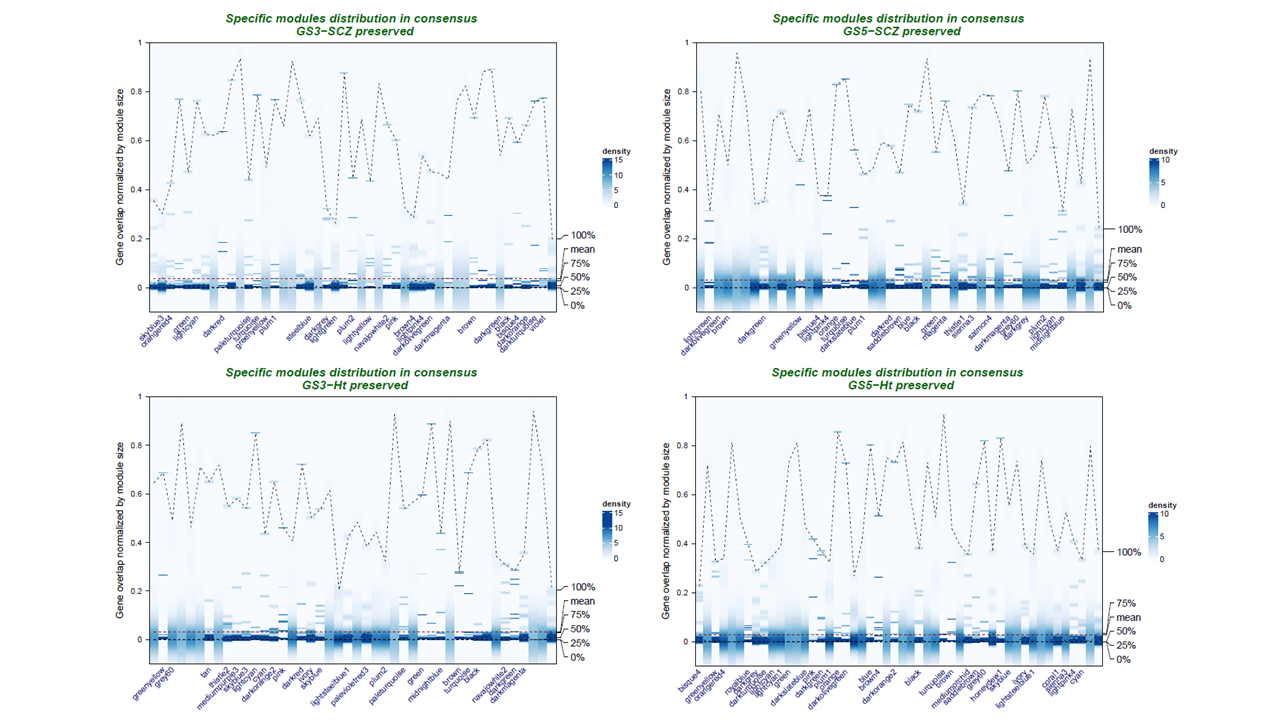

Supplement: S27 Fig — Annotated modules on the x axis represent fragmented modules with weaker conservation in their background modules. (TIF) [file pgen.1010989.s037.tif]

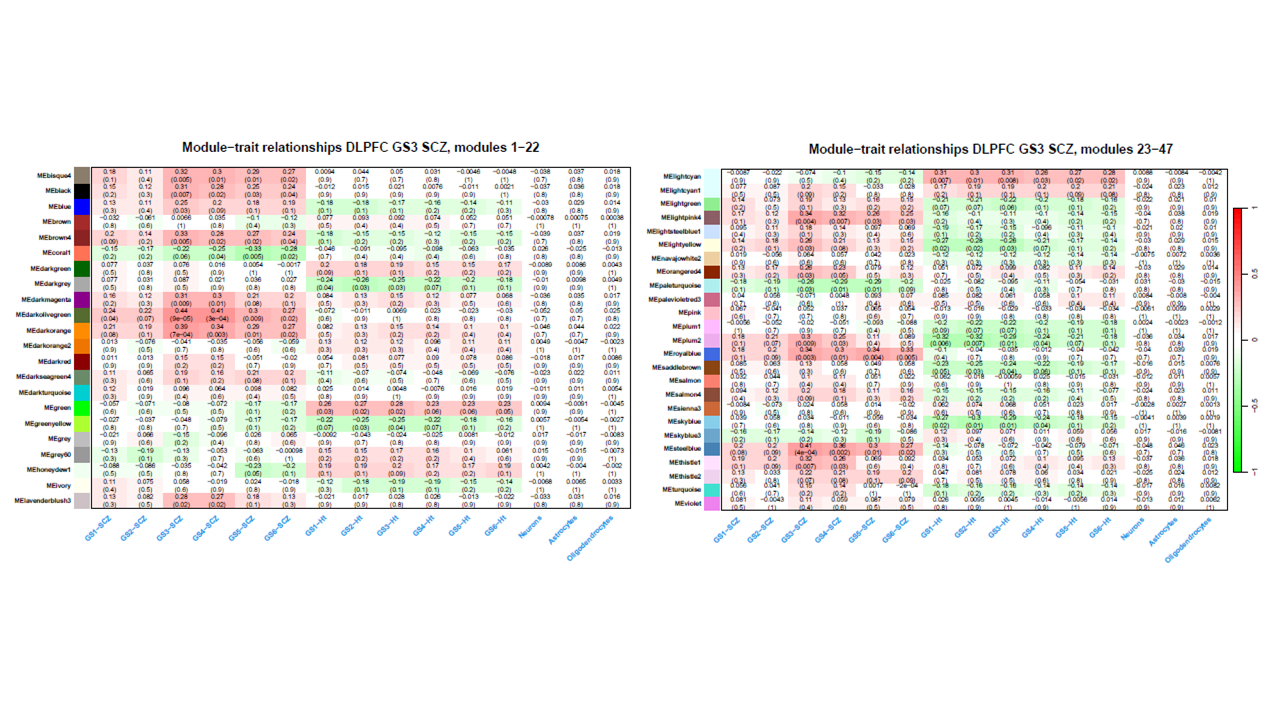

Supplement: S28 Fig — SCZ risk genomic scores GS1-SCZ (pGWAS<5e-08)-GS6-SCZ (pGWAS < .05), and height genomic scores GS1-Ht-GS6-Ht (same pGWAS thresholds). Last four columns: correlations of MEs and cell type proportions to quality check the removal of variance explained by cell type proportion. Virtually no ME had correlations with cell type proportions, which confirms the efficient cell type deconvolution for neurons, astrocytes and endothelia. (TIF) [file pgen.1010989.s038.tif]

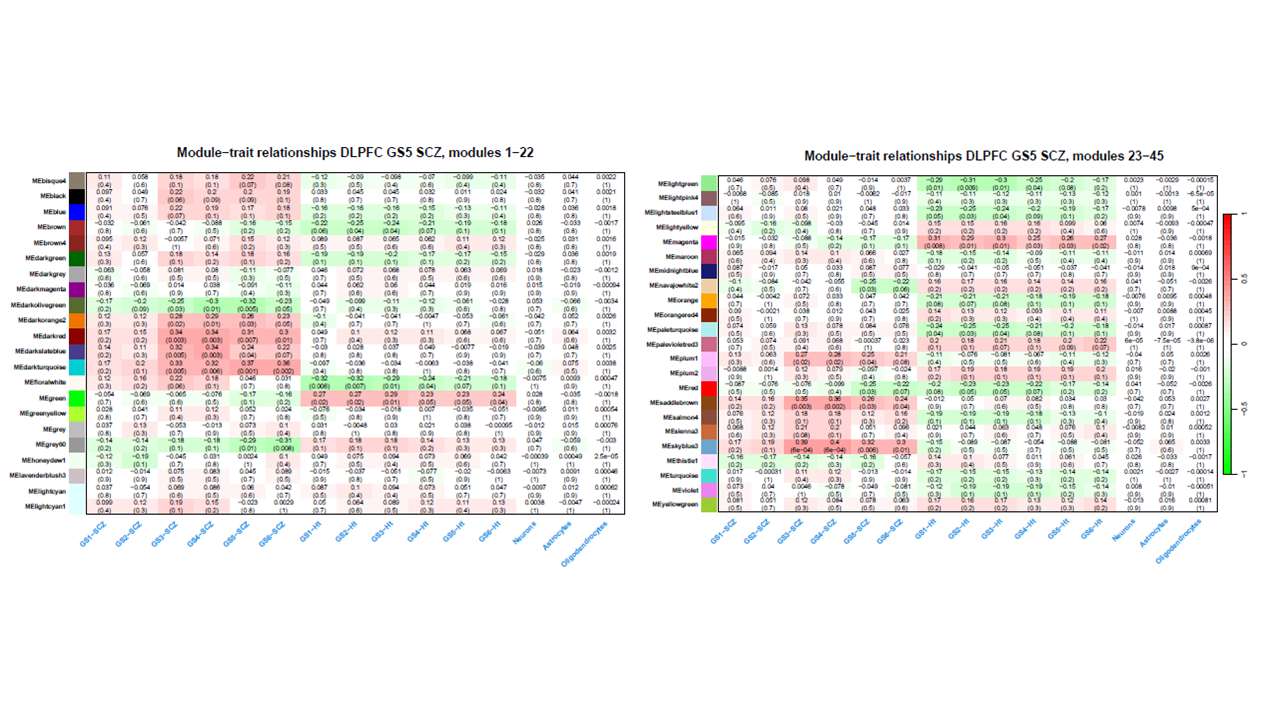

Supplement: S29 Fig — SCZ risk genomic scores GS1-SCZ (pGWAS<5e-08)-GS6-SCZ (pGWAS < .05), and height genomic scores GS1-Ht-GS6-Ht (same pGWAS thresholds). Last four columns: correlations of MEs and cell type proportions to quality check the removal of variance explained by cell type proportion. Virtually no ME had correlations with cell type proportions, which confirms the efficient cell type deconvolution for neurons, astrocytes and endothelia. (TIF) [file pgen.1010989.s039.tif]

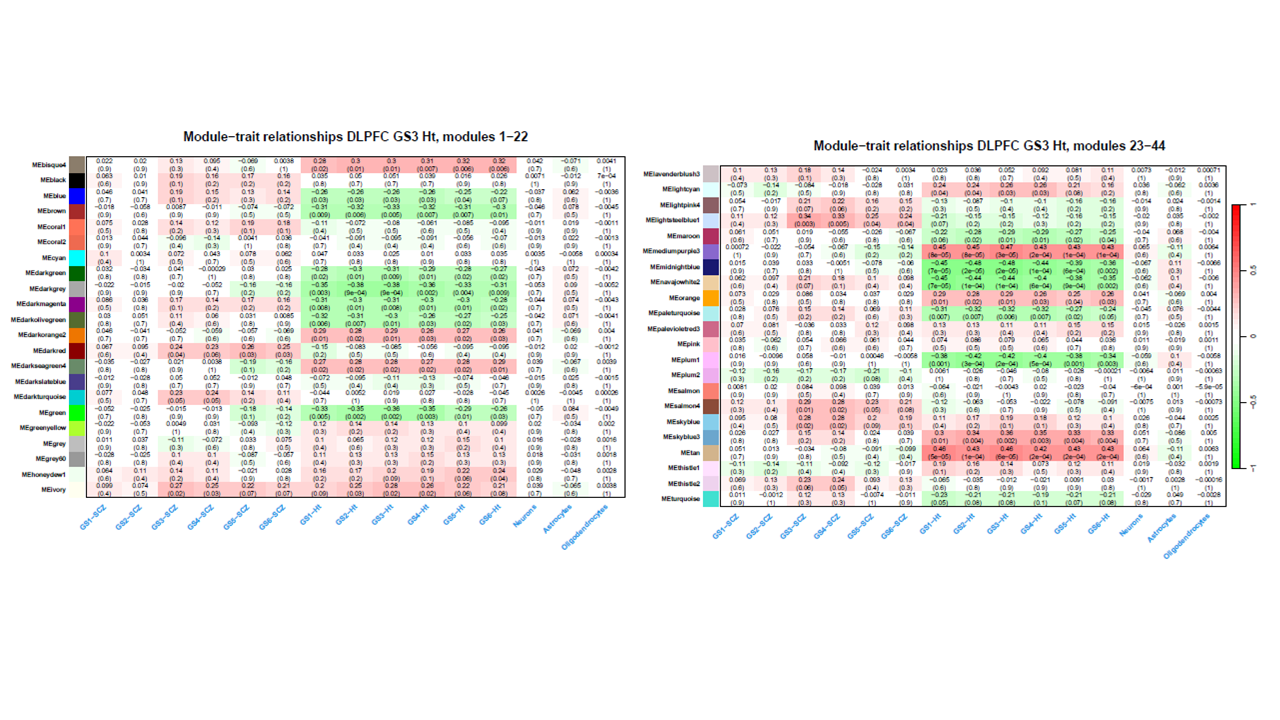

Supplement: S30 Fig — SCZ risk genomic scores GS1-SCZ (pGWAS<5e-08)-GS6-SCZ (pGWAS < .05), and height genomic scores GS1-Ht-GS6-Ht (same pGWAS thresholds). Last four columns: correlations of MEs and cell type proportions to quality check the removal of variance explained by cell type proportion. Virtually no ME had correlations with cell type proportions, which confirms the efficient cell type deconvolution for neurons, astrocytes and endothelia. (TIF) [file pgen.1010989.s040.tif]

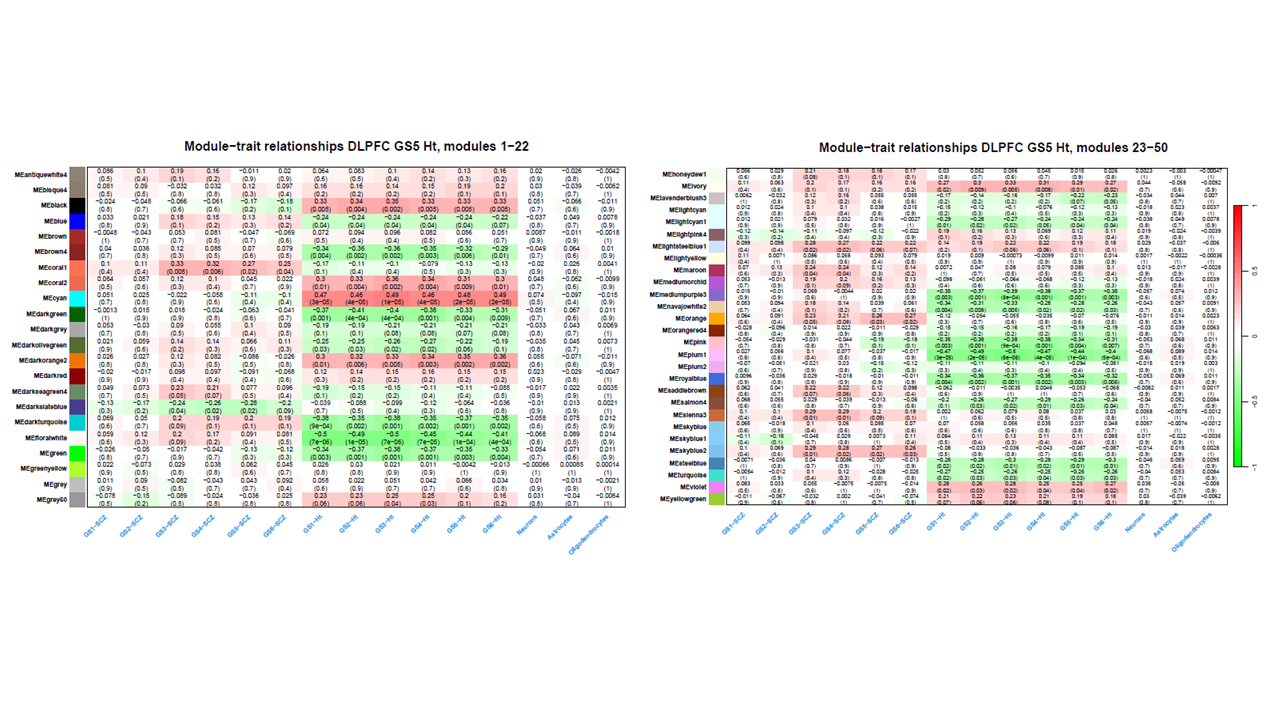

Supplement: S31 Fig — SCZ risk genomic scores GS1-SCZ (pGWAS<5e-08)-GS6-SCZ (pGWAS < .05), and height genomic scores GS1-Ht-GS6-Ht (same pGWAS thresholds). Last four columns: correlations of MEs and cell type proportions to quality check the removal of variance explained by cell type proportion. Virtually no ME had correlations with cell type proportions, which confirms the efficient cell type deconvolution for neurons, astrocytes and endothelia. (TIF) [file pgen.1010989.s041.tif]

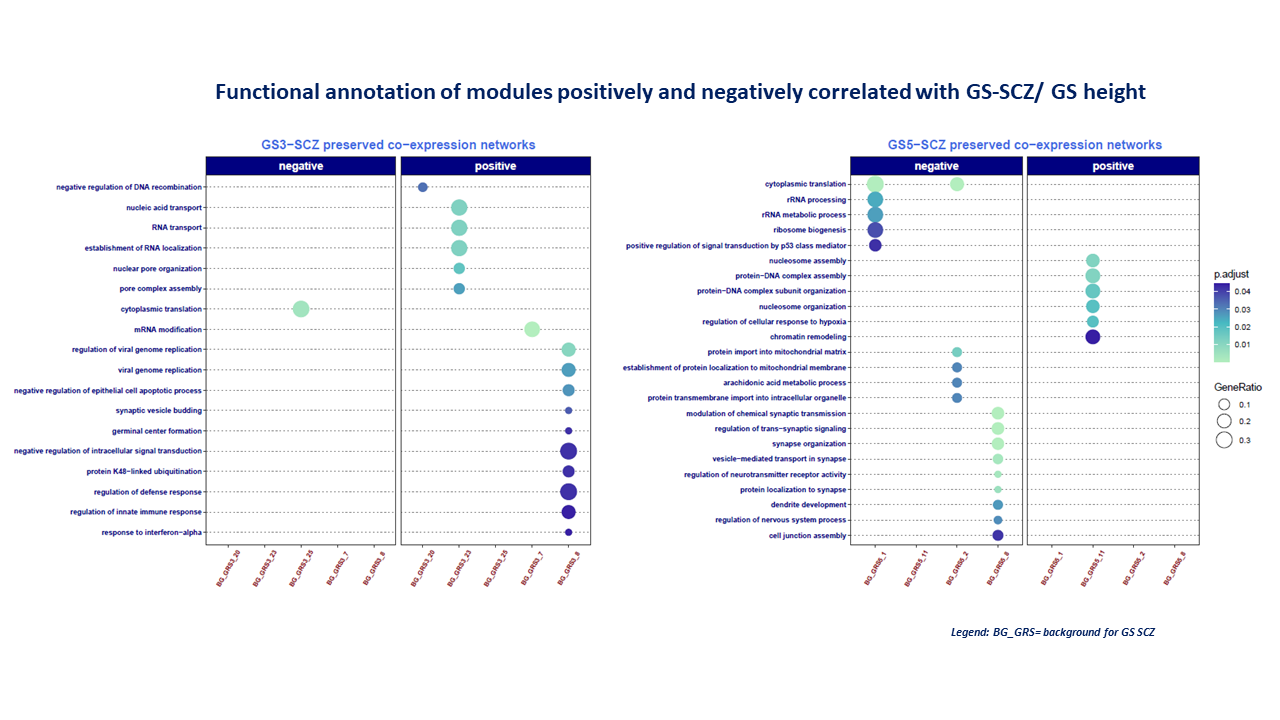

Supplement: S32 Fig — Less distinct pattern of functional divergence by MEs correlation directionality, although the functional segregation of modules is conserved. Only in the GS5-SCZ preserved modules with negative correlations between MEs and GS-SCZ an enrichment in neuronal pathways is apparent. However, there are also modules with MEs negatively correlated with GS-SCZ and enriched in ontologies related to more general cellular processes Legend: BG_GS3-SCZ_ / BG_GS5-SCZ_x = fragments from SCZ risk GS3-SCZ or GS5-SCZ preserved modules overlapped with background (BG) modules. (TIF) [file pgen.1010989.s042.tif]

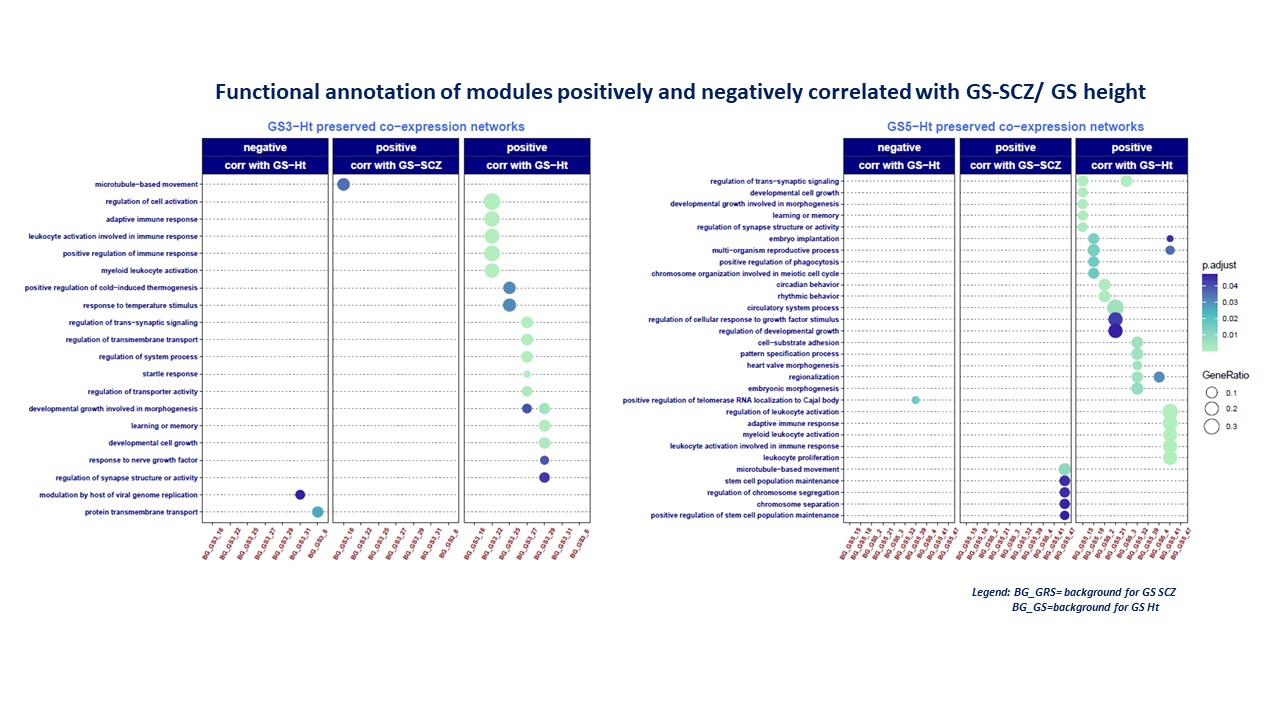

Supplement: S33 Fig — Relative functional divergence in biological processes enrichment like in LIBD GS preserved modules: cellular general ontologies are enriched in gene sets originated from modules with MEs positively correlated with GS-SCZs SCZ; nervous system development and functionality ontologies are enriched in gene sets originated from modules with MEs positively correlated with GS Ht. Legend: BG_GS3-Ht_ / BG_GS5-Ht_x = fragments from height GS3 or GS5 preserved modules overlapped with background (BG) modules. (TIF) [file pgen.1010989.s043.tif]

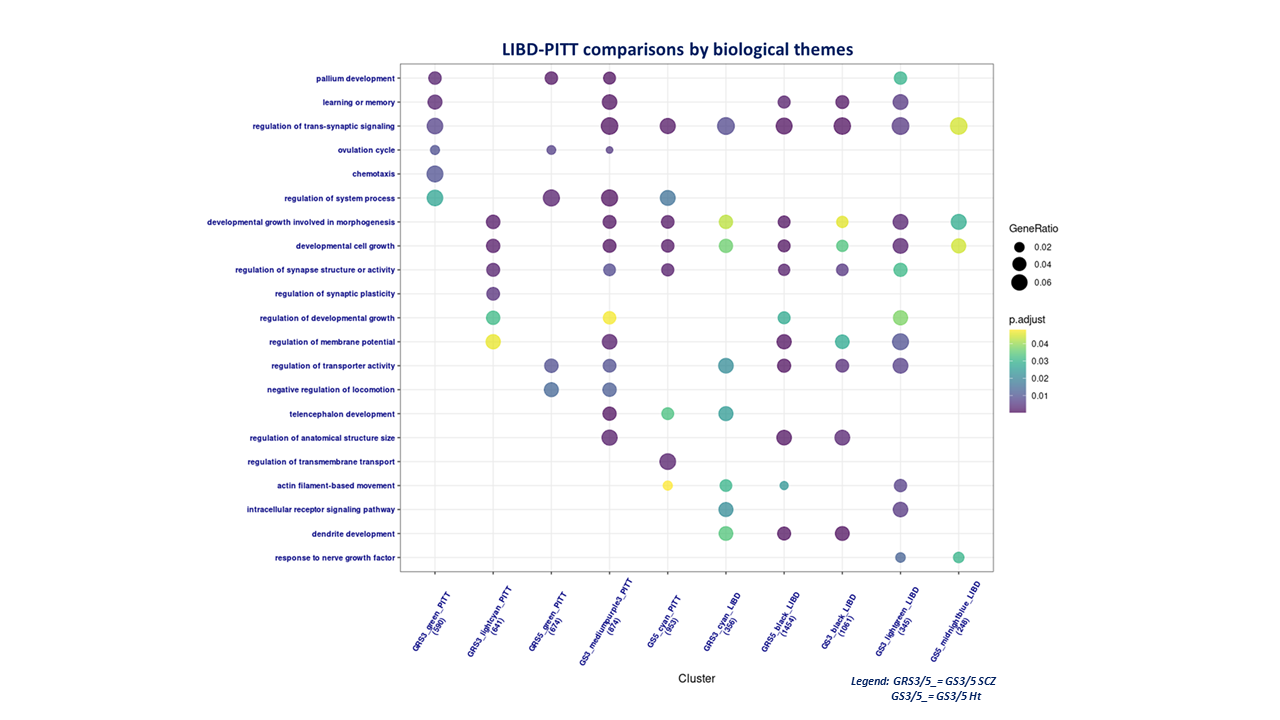

Supplement: S34 Fig — (TIF) [file pgen.1010989.s044.tif]

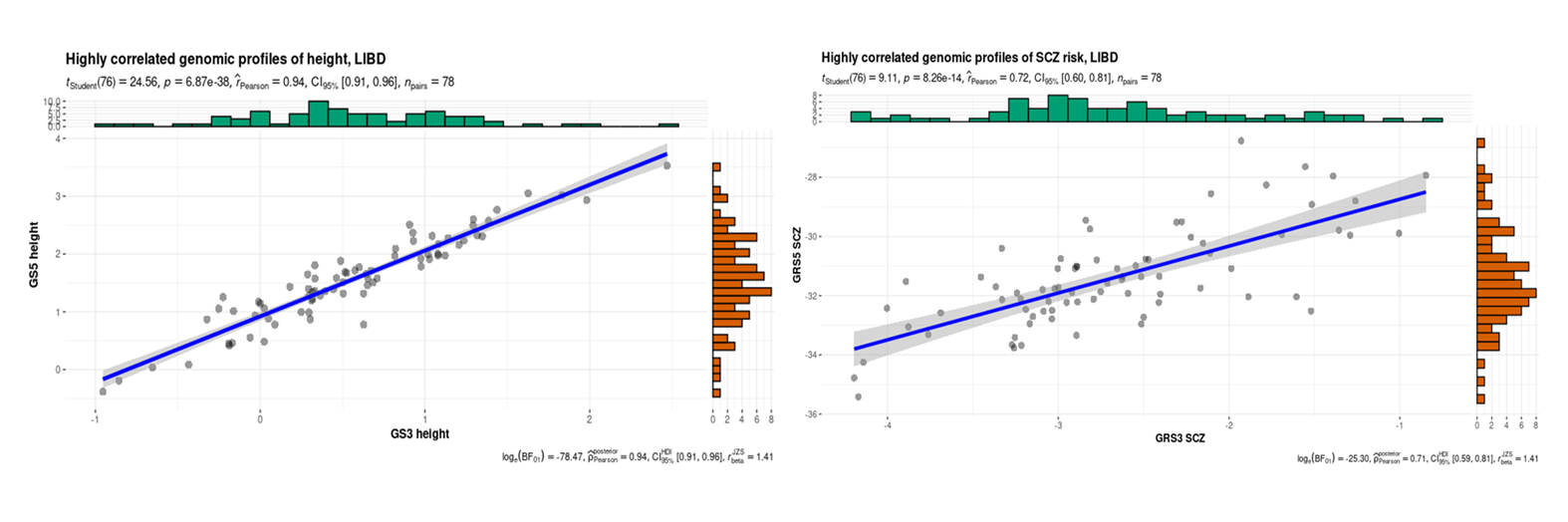

Supplement: S35 Fig — (TIF) [file pgen.1010989.s045.tif]

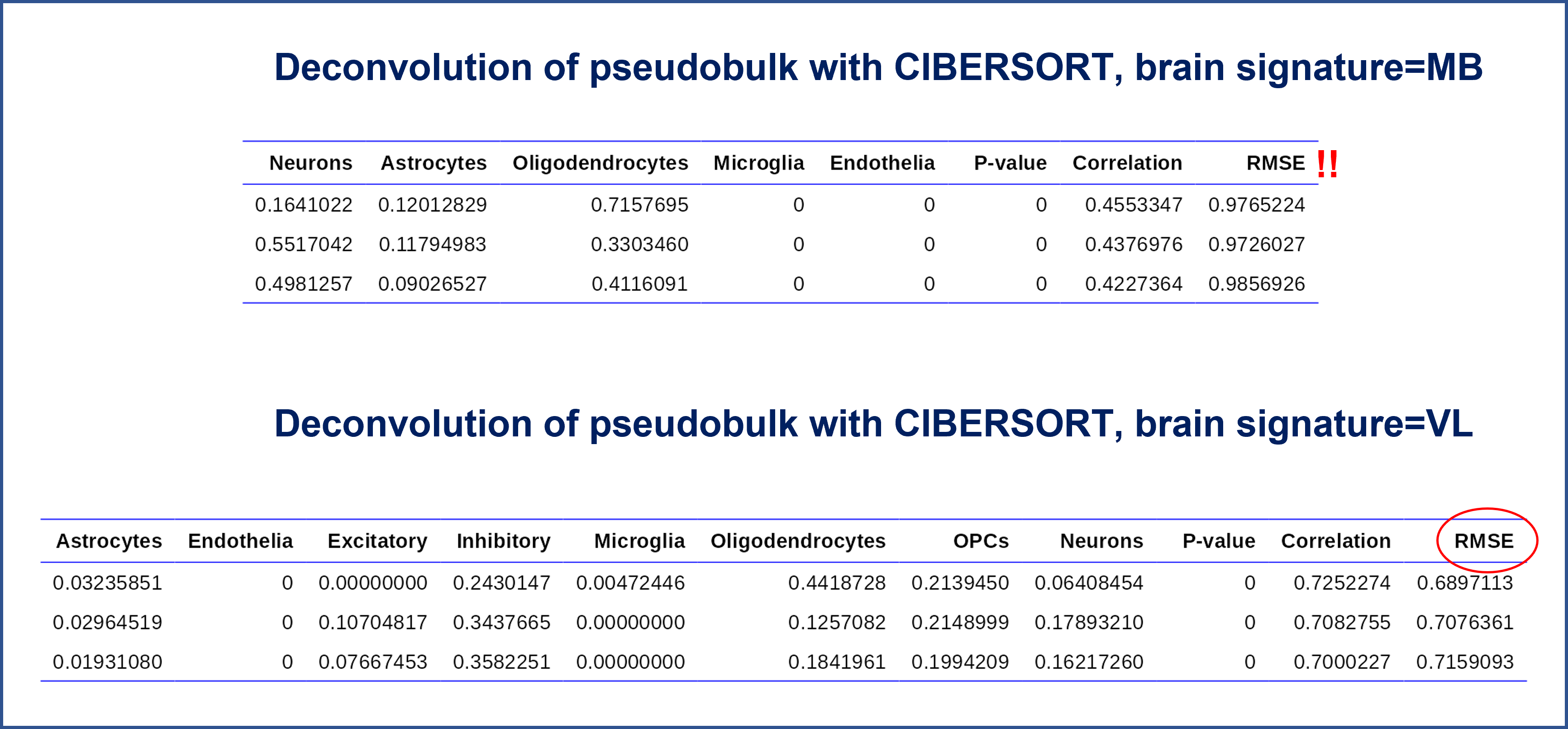

Supplement: S36 Fig — The algorithm fails though to estimate microglia and endothelia. (TIF) [file pgen.1010989.s046.tif]

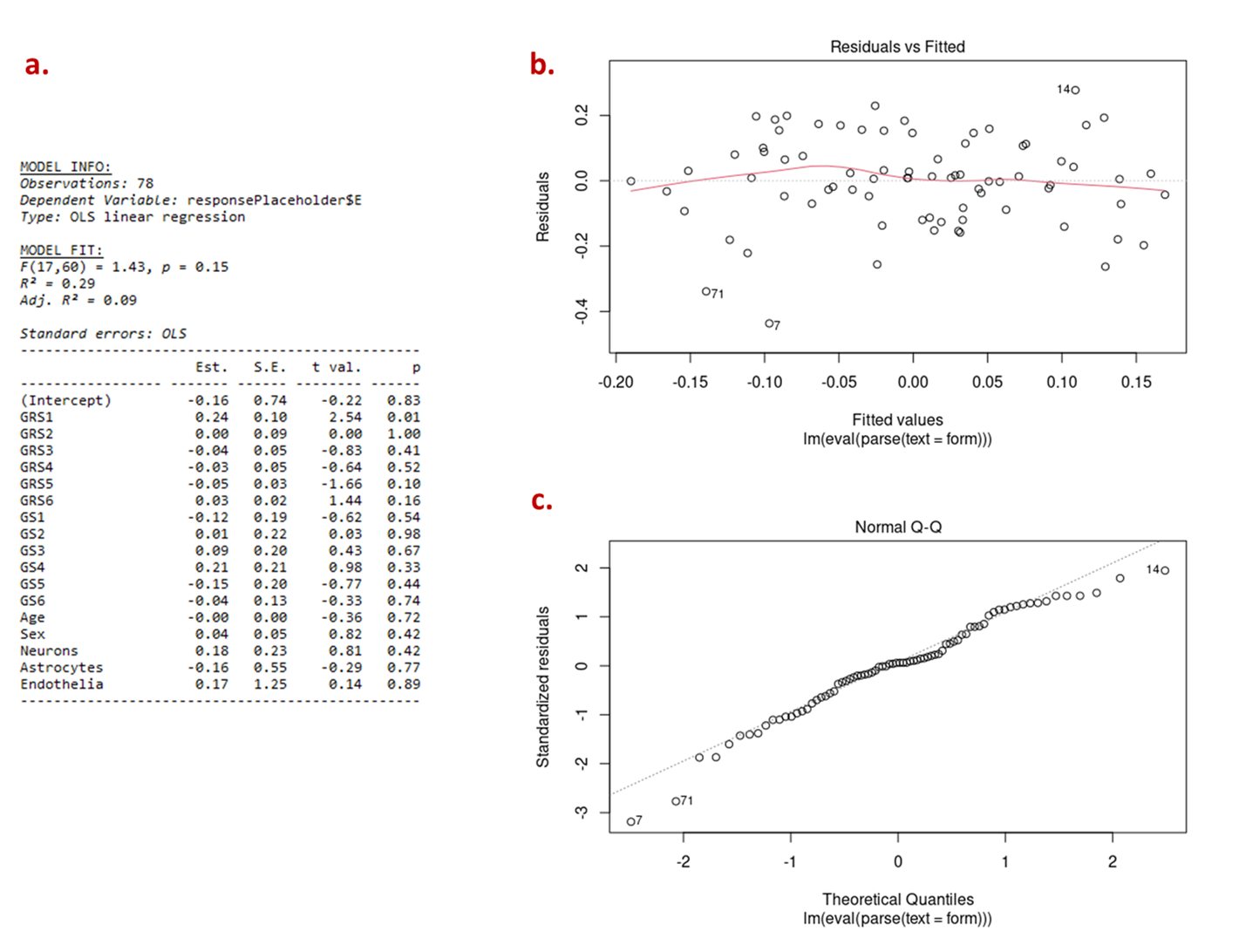

Supplement: S37 Fig — a. Summary statistics of the model; b. and c. Diagnostics plots of the model: residuals vs. fitted and normal quantile-quantile plots showing a relatively good fit for the model. (TIF) [file pgen.1010989.s047.tif]

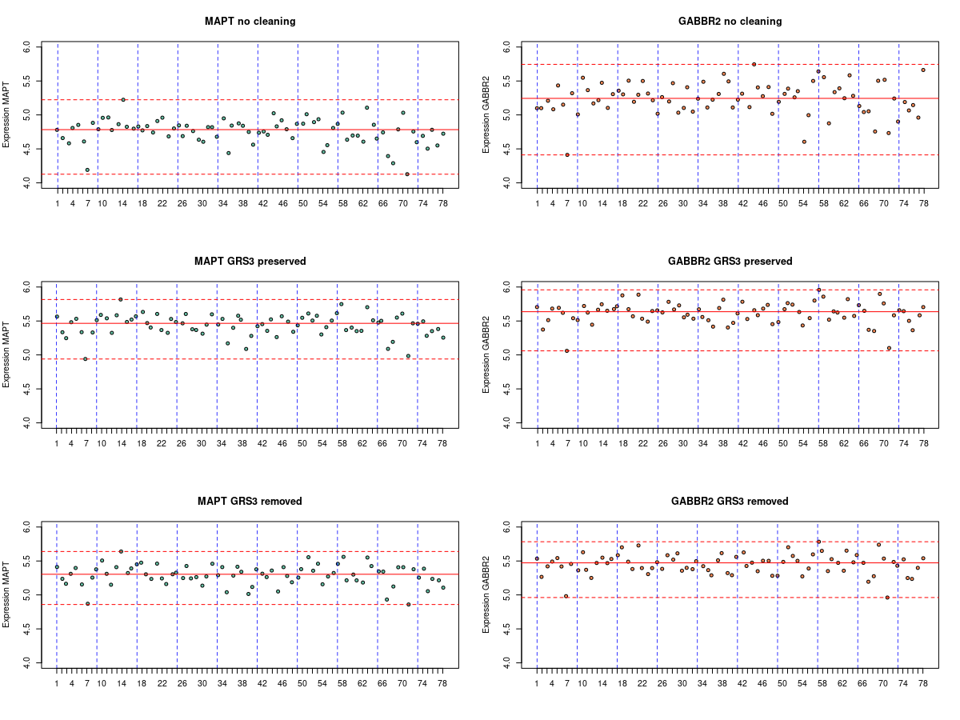

Supplement: S38 Fig — Small variations are observed in the expression data in the 3 scenarios (no “cleaning”, preserved GS3-SCZ and removed GS3-SCZ) (on x axis: the 78 LIBD DLPFC samples). (TIF) [file pgen.1010989.s048.tif]

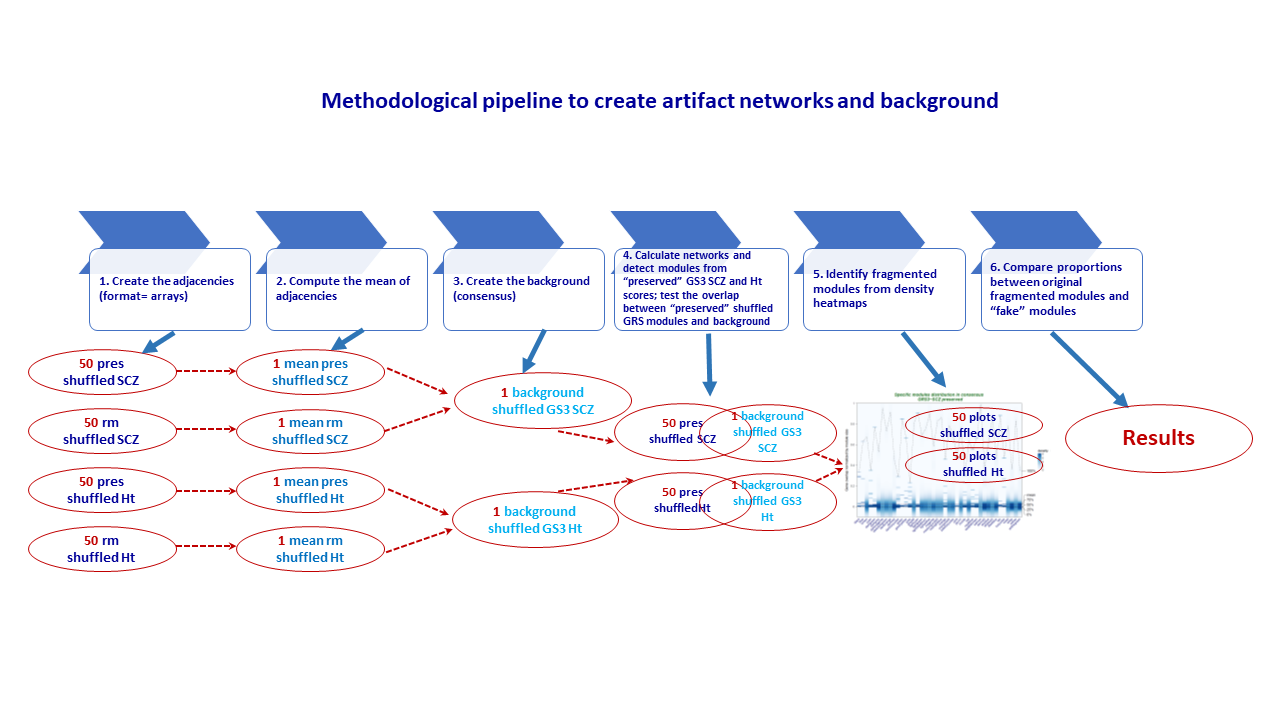

Supplement: S39 Fig — (TIF) [file pgen.1010989.s049.tif]
