# Supplementary material for: Investigating trait variability of gene co-expression network architecture in brain by controlling for genomic risk of schizophrenia
Source: PLoS Genet. 2023 Oct 13;19(10):e1010989. doi: 10.1371/journal.pgen.1010989 (PMC10599557; doi:10.1371/journal.pgen.1010989)
Supplement: S1 Appendix — (PDF) [file pgen.1010989.s050.pdf]

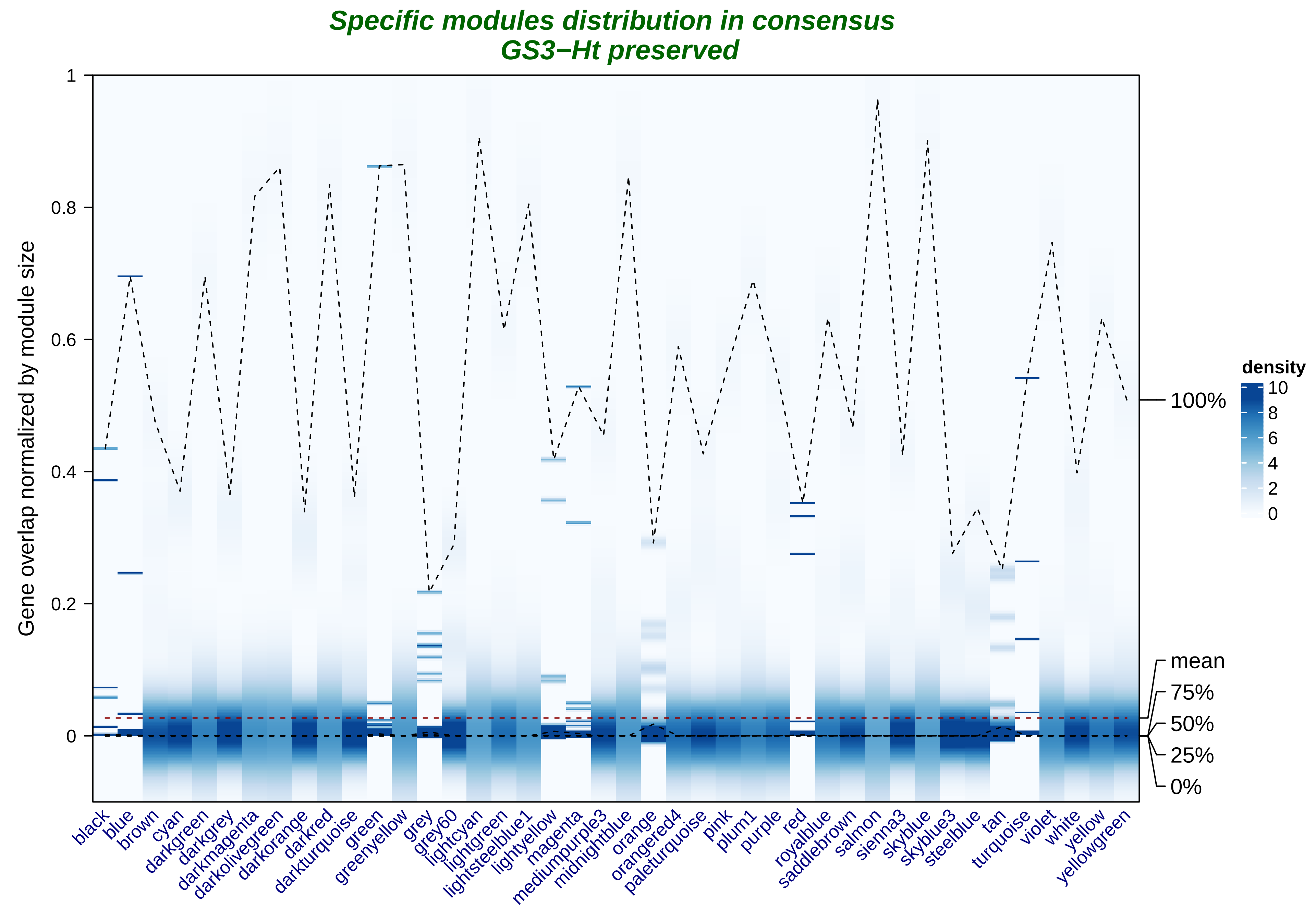
Specific modules distribution in consensus
GS3−Ht preserved
Gene overlap normalized by module size
1
0.8
0.6
0.4
0.2
0
density
10
8
6
4
2
0
100%
mean
75%
50%
25%
0%
black
blue
brown
cyan
darkgreen
darkgrey
darkmagenta
darkolivegreen
darkorange
darkred
darkturquoise
green
greenyellow
grey
grey60
lightcyan
lightgreen
lightsteelblue1
lightyellow
magenta
mediumpurple3
midnightblue
orange
orangered4
paleturquoise
pink
plum1
purple
red
royalblue
saddlebrown
salmon
sienna3
skyblue
skyblue3
steelblue
tan
turquoise
violet
white
yellow
yellowgreen

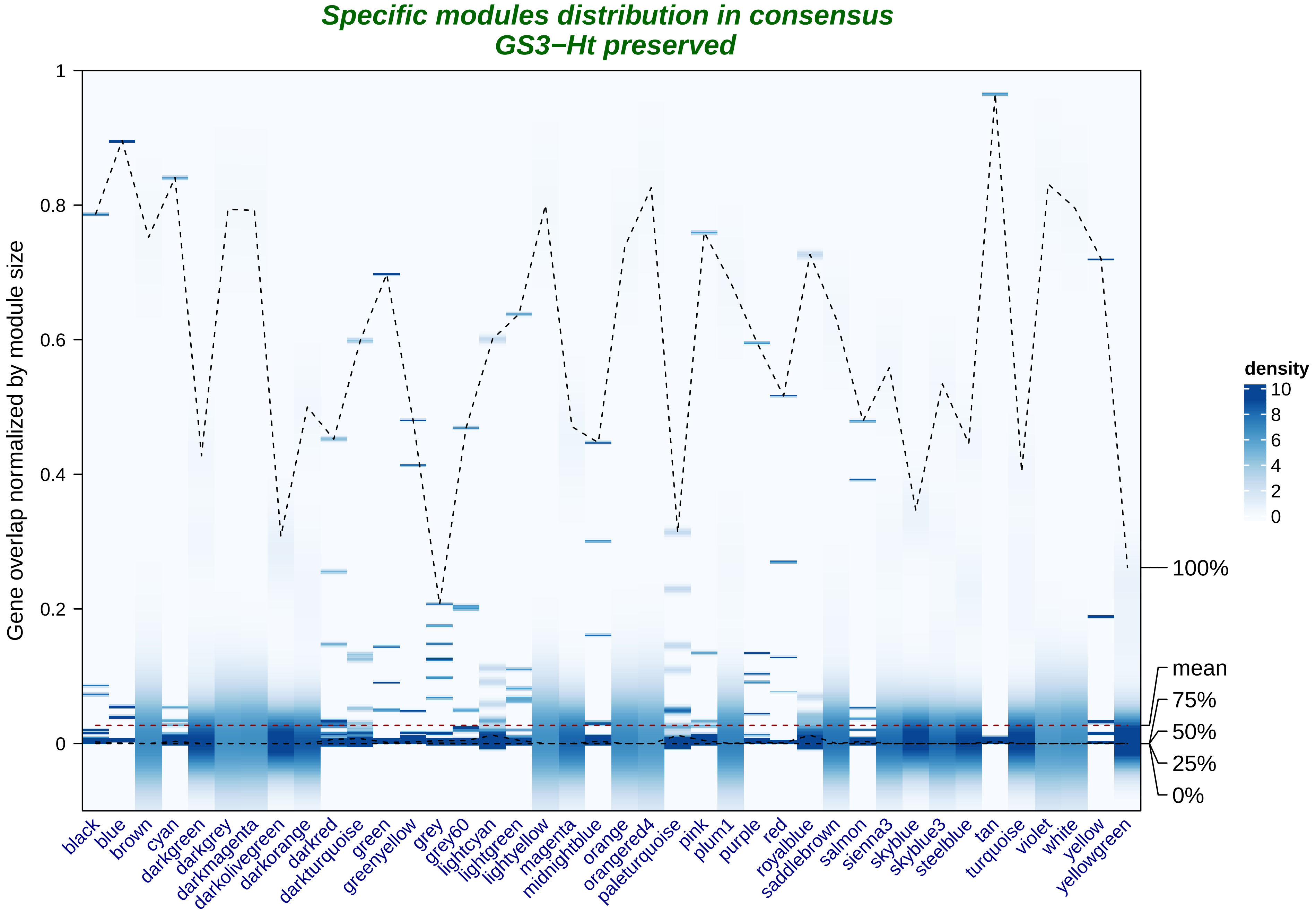
Specific modules distribution in consensus
GS3−Ht preserved
Gene overlap normalized by module size
1
0.8
0.6
0.4
0.2
0
density
10
8
6
4
2
0
100%
mean
75%
50%
25%
0%
black
blue
brown
cyan
darkgreen
darkgrey
darkmagenta
darkolivegreen
darkorange
darkred
darkturquoise
green
greenyellow
grey
grey60
lightcyan
lightgreen
lightyellow
magenta
midnightblue
orange
orangered4
paleturquoise
pink
plum1
purple
red
royalblue
saddlebrown
salmon
sienna3
skyblue
skyblue3
steelblue
tan
turquoise
violet
white
yellow
yellowgreen

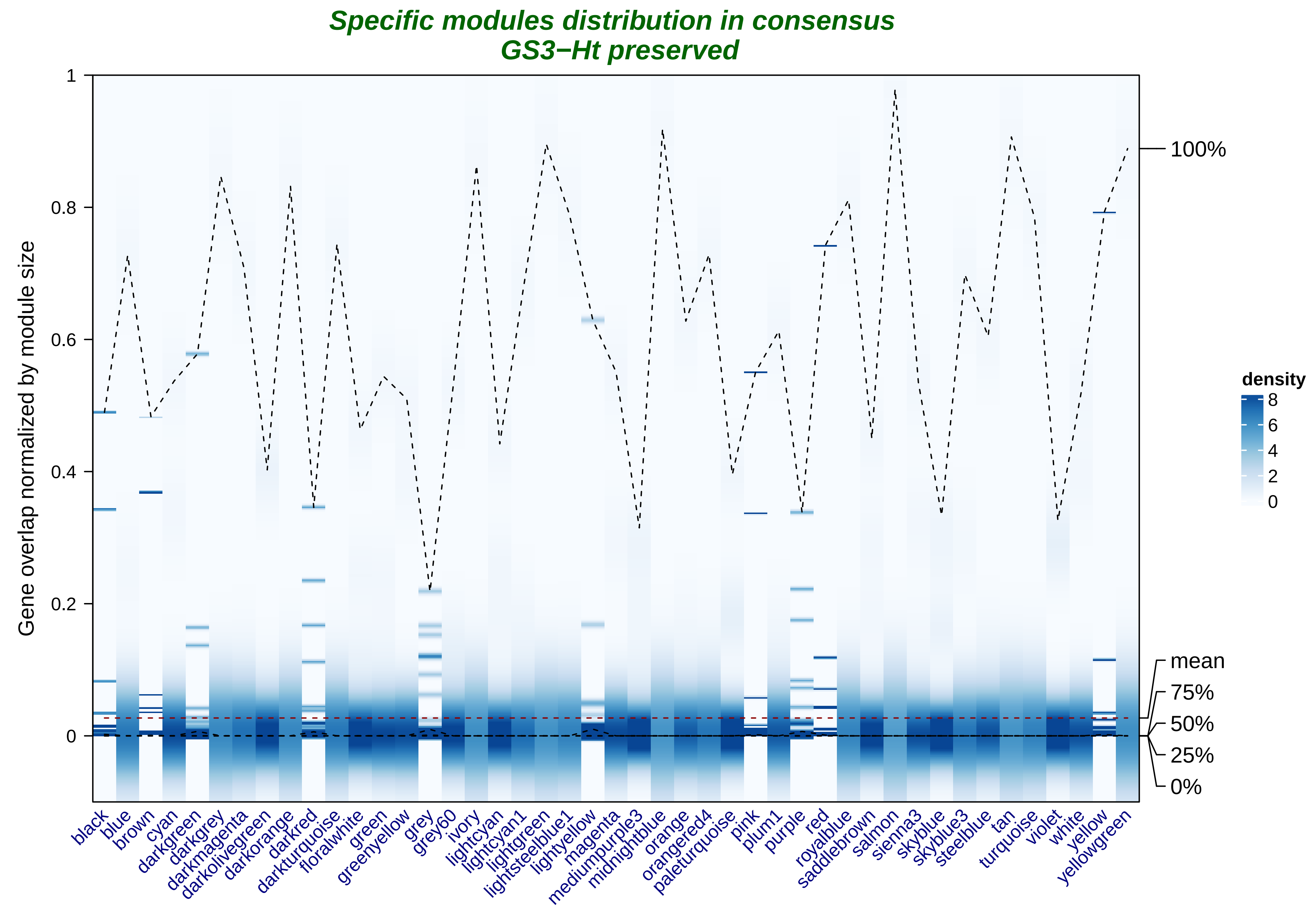

Specific modules distribution in consensus
GS3−Ht preserved
Gene overlap normalized by module size
1
0.8
0.6
0.4
0.2
0
100%
mean
75%
50%
25%
0%
density
8
6
4
2
0
black
blue
brown
cyan
darkgreen
darkgrey
darkmagenta
darkolivegreen
darkorange
darkred
darkturquoise
floralwhite
green
greenyellow
grey
grey60
ivory
lightcyan
lightcyan1
lightgreen
lightsteelblue1
lightyellow
magenta
mediumpurple3
midnightblue
orange
orangered4
paleturquoise
pink
plum1
purple
red
royalblue
saddlebrown
salmon
sienna3
skyblue
skyblue3
steelblue
tan
turquoise
violet
white
yellow
yellowgreen

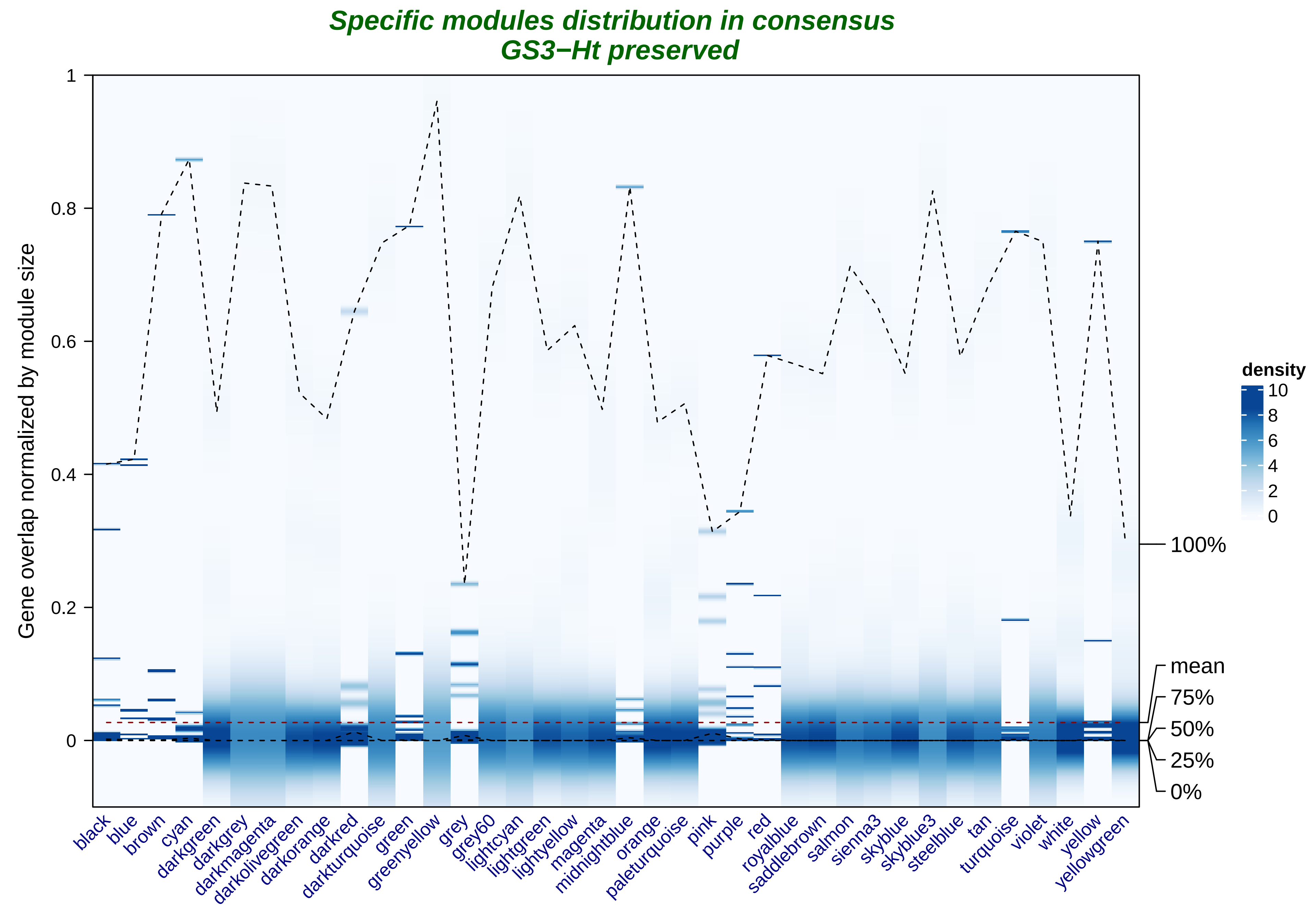
Specific modules distribution in consensus
GS3−Ht preserved
Gene overlap normalized by module size
1
0.8
0.6
0.4
0.2
0
density
10
8
6
4
2
0
100%
mean
75%
50%
25%
0%
black
blue
brown
cyan
darkgreen
darkgrey
darkmagenta
darkolivegreen
darkorange
darkred
darkturquoise
green
greenyellow
grey
grey60
lightcyan
lightgreen
lightyellow
magenta
midnightblue
orange
paleturquoise
pink
purple
red
royalblue
saddlebrown
salmon
sienna3
skyblue
skyblue3
steelblue
tan
turquoise
violet
white
yellow
yellowgreen

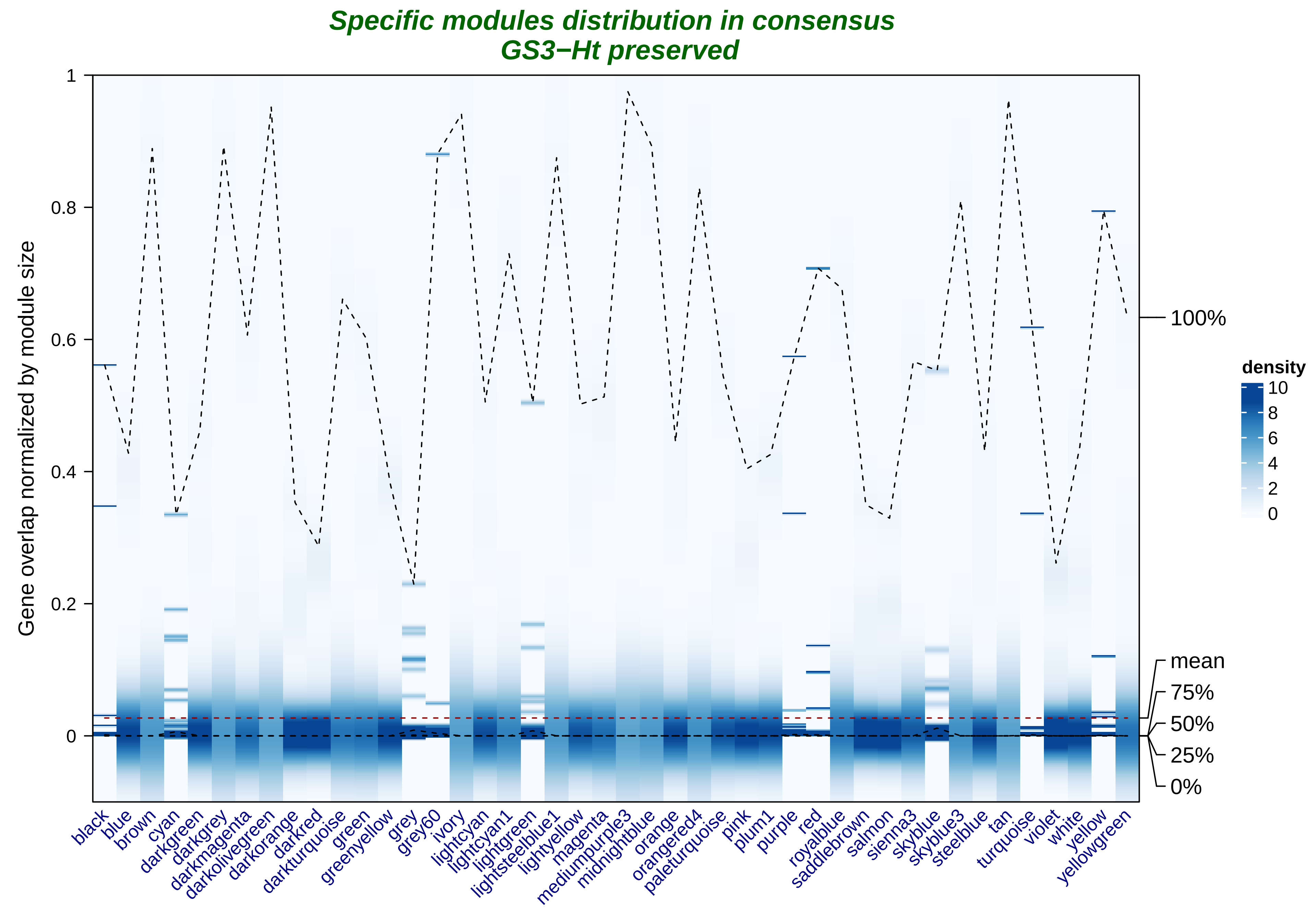

Specific modules distribution in consensus
GS3−Ht preserved
Gene overlap normalized by module size
1
0.8
0.6
0.4
0.2
0
100%
mean
75%
50%
25%
0%
density
10
8
6
4
2
0
black
blue
brown
cyan
darkgreen
darkgrey
darkmagenta
darkolivegreen
darkorange
darkred
darkturquoise
green
greenyellow
grey
grey60
ivory
lightcyan
lightcyan1
lightgreen
lightsteelblue1
lightyellow
magenta
mediumpurple3
midnightblue
orange
orangered4
paleturquoise
pink
plum1
purple
red
royalblue
saddlebrown
salmon
sienna3
skyblue
skyblue3
steelblue
tan
turquoise
violet
white
yellow
yellowgreen

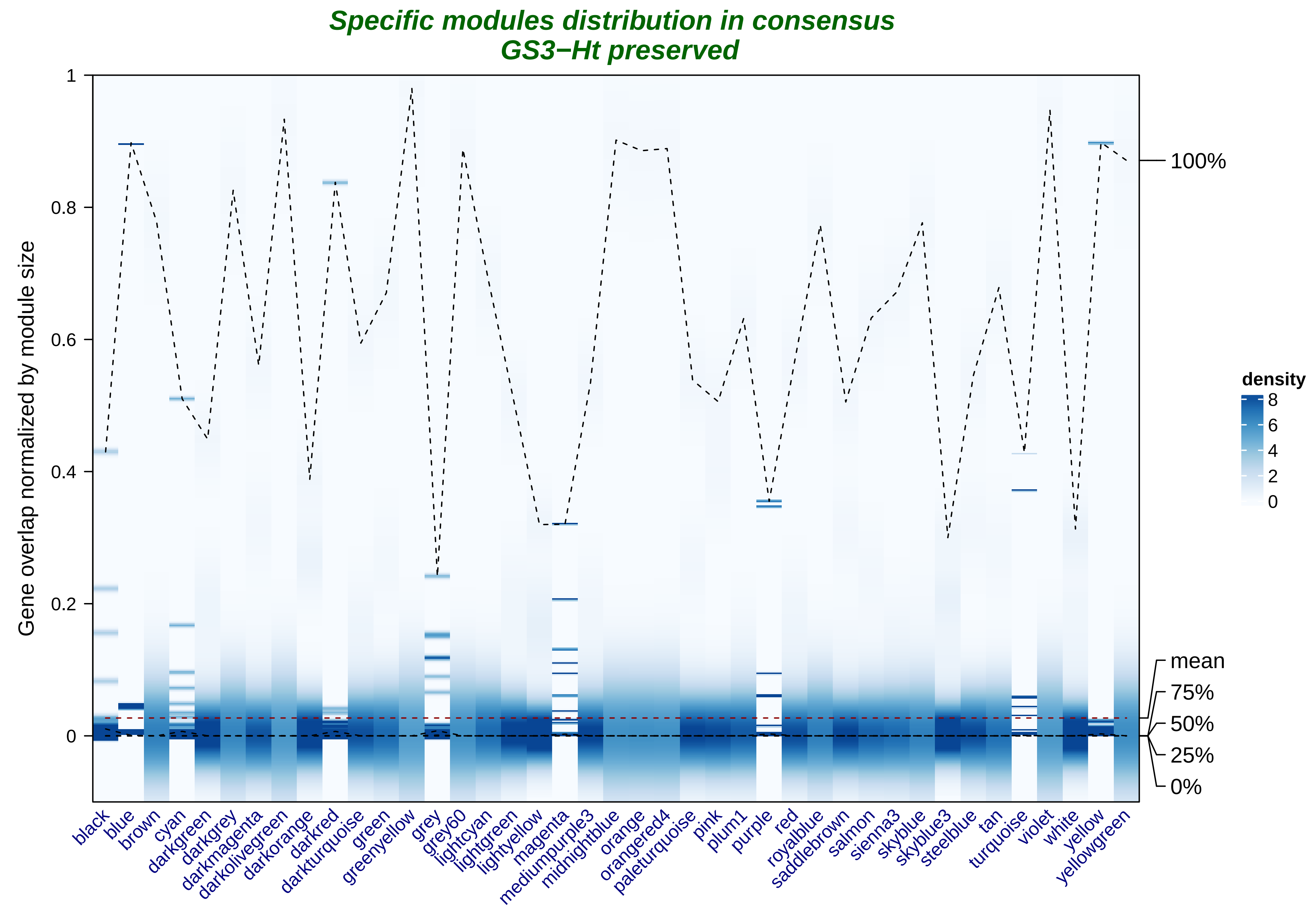
Specific modules distribution in consensus
GS3−Ht preserved
Gene overlap normalized by module size
1
0.8
0.6
0.4
0.2
0
100%
mean
75%
50%
25%
0%
density
8
6
4
2
0
black
blue
brown
cyan
darkgreen
darkgrey
darkmagenta
darkolivegreen
darkorange
darkred
darkturquoise
green
greenyellow
grey
grey60
lightcyan
lightgreen
lightyellow
magenta
mediumpurple3
midnightblue
orange
orangered4
paleturquoise
pink
plum1
purple
red
royalblue
saddlebrown
salmon
sienna3
skyblue
skyblue3
steelblue
tan
turquoise
violet
white
yellow
yellowgreen

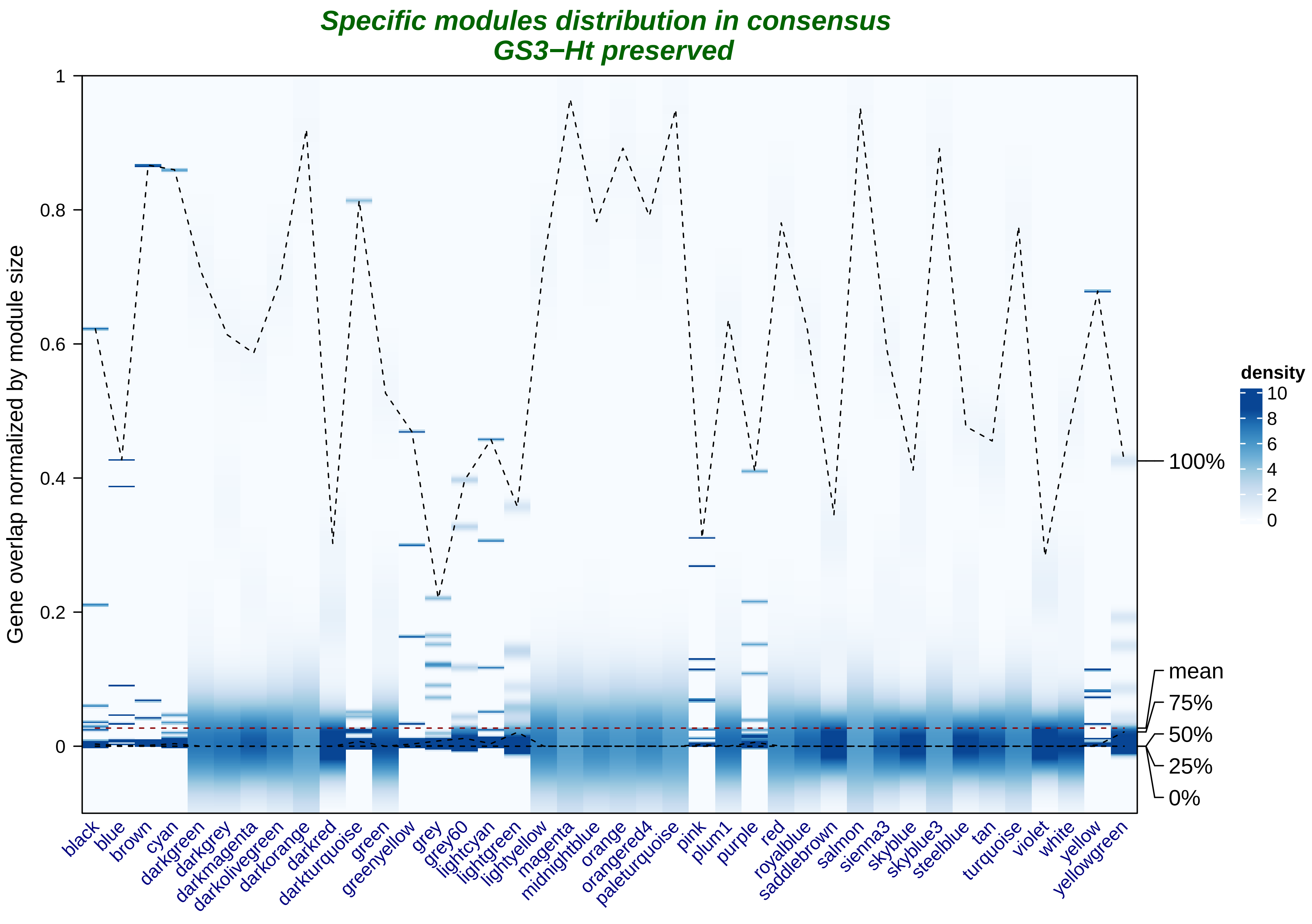

Specific modules distribution in consensus
GS3−Ht preserved
Gene overlap normalized by module size
1
0.8
0.6
0.4
0.2
0
black
blue
brown
cyan
darkgreen
darkgrey
darkmagenta
darkolivegreen
darkorange
darkred
darkturquoise
green
greenyellow
grey
grey60
lightcyan
lightgreen
lightyellow
magenta
midnightblue
orange
orangered4
paleturquoise
pink
plum1
purple
red
royalblue
saddlebrown
salmon
sienna3
skyblue
skyblue3
steelblue
tan
turquoise
violet
white
yellow
yellowgreen
100%
mean
75%
50%
25%
0%
density
10
8
6
4
2
0

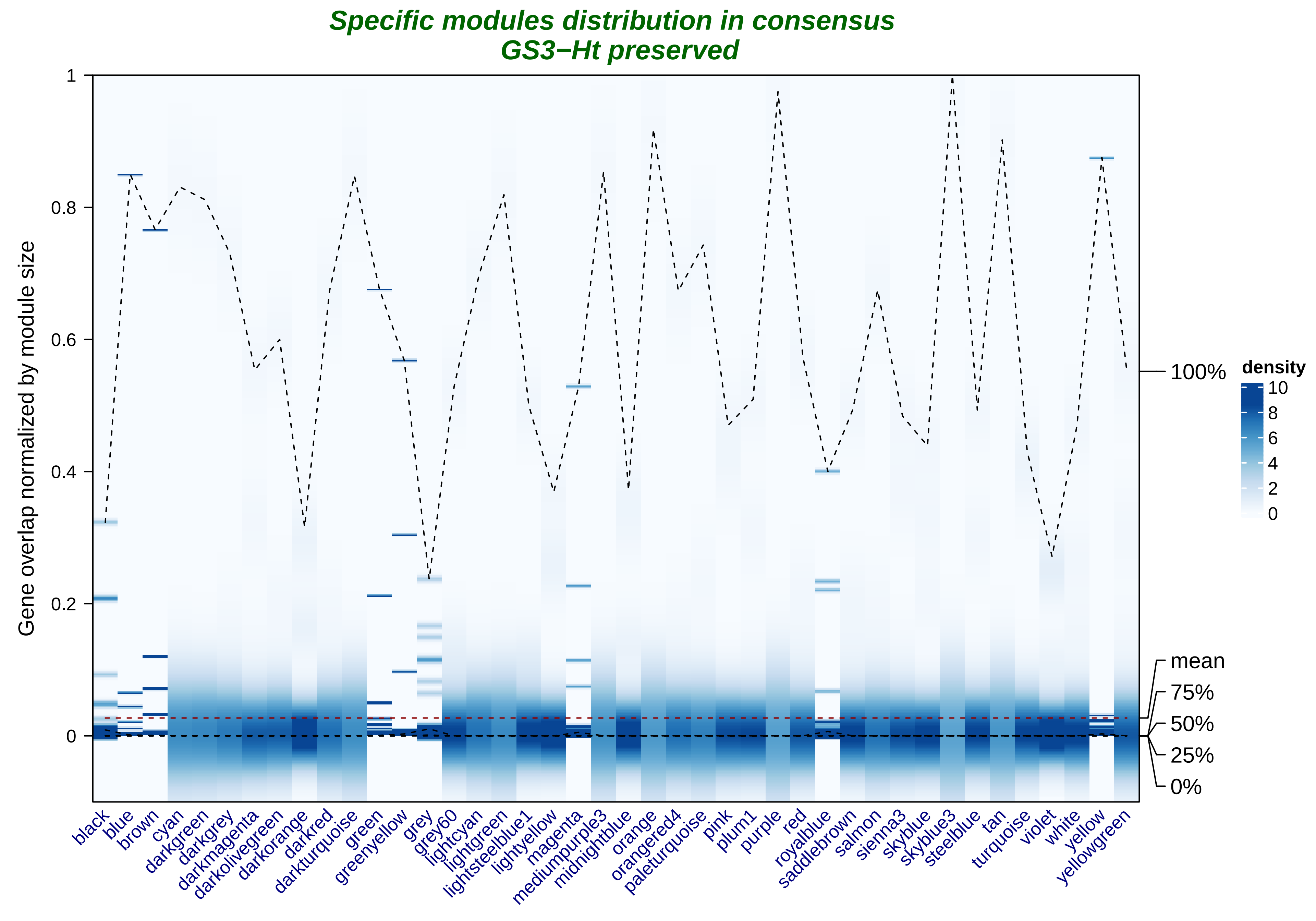
Specific modules distribution in consensus
GS3−Ht preserved
Gene overlap normalized by module size
1
0.8
0.6
0.4
0.2
0
100%
mean
75%
50%
25%
0%
density
10
8
6
4
2
0
black
blue
brown
cyan
darkgreen
darkgrey
darkmagenta
darkolivegreen
darkorange
darkred
darkturquoise
green
greenyellow
grey
grey60
lightcyan
lightgreen
lightsteelblue1
lightyellow
magenta
mediumpurple3
midnightblue
orange
orangered4
paleturquoise
pink
plum1
purple
red
royalblue
saddlebrown
salmon
sienna3
skyblue
skyblue3
steelblue
tan
turquoise
violet
white
yellow
yellowgreen

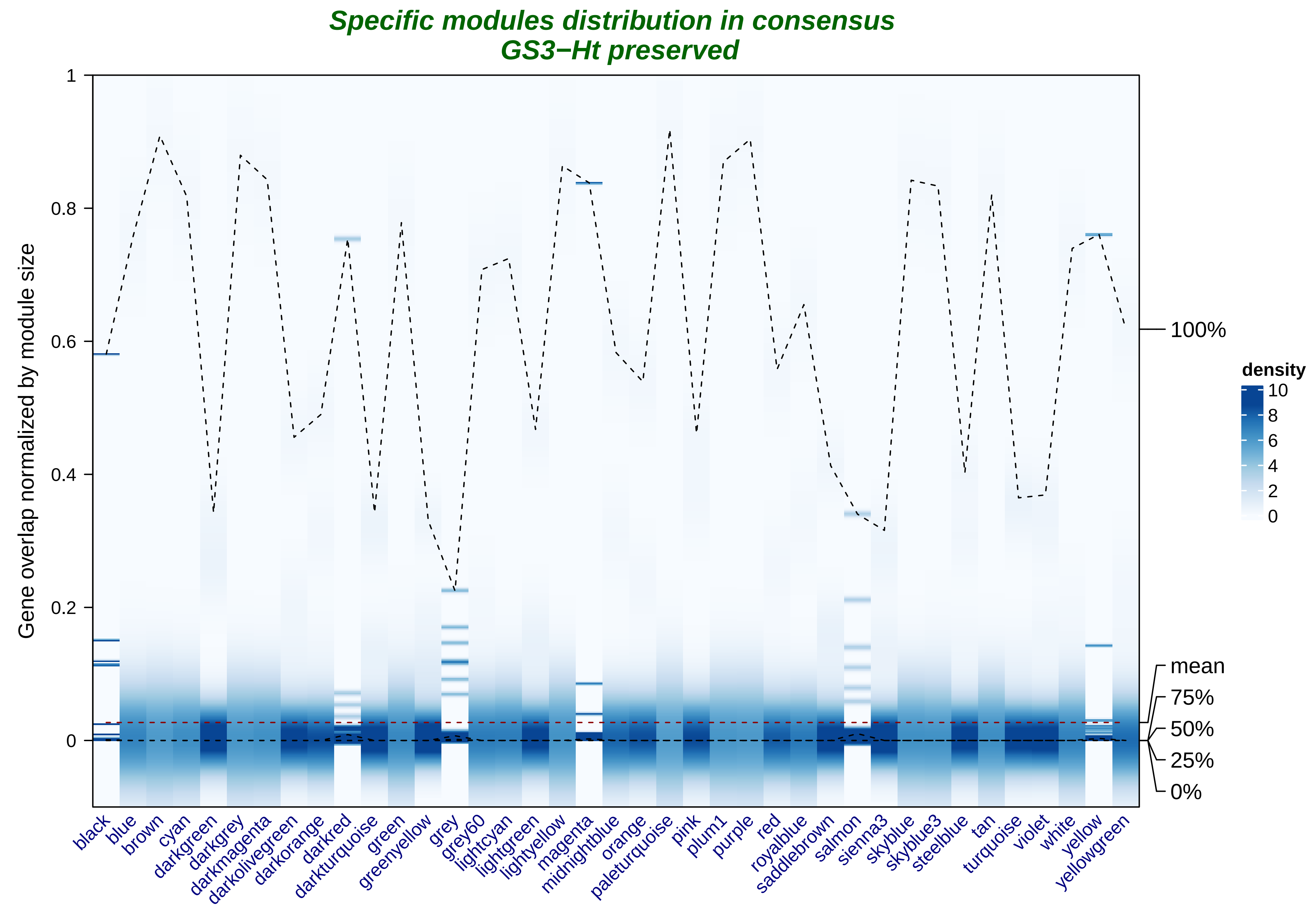
Specific modules distribution in consensus
GS3−Ht preserved
Gene overlap normalized by module size
1
0.8
0.6
0.4
0.2
0
100%
mean
75%
50%
25%
0%
density
10
8
6
4
2
0
black
blue
brown
cyan
darkgreen
darkgrey
darkmagenta
darkolivegreen
darkorange
darkred
darkturquoise
green
greenyellow
grey
grey60
lightcyan
lightgreen
lightyellow
magenta
midnightblue
orange
paleturquoise
pink
plum1
purple
red
royalblue
saddlebrown
salmon
sienna3
skyblue
skyblue3
steelblue
tan
turquoise
violet
white
yellow
yellowgreen

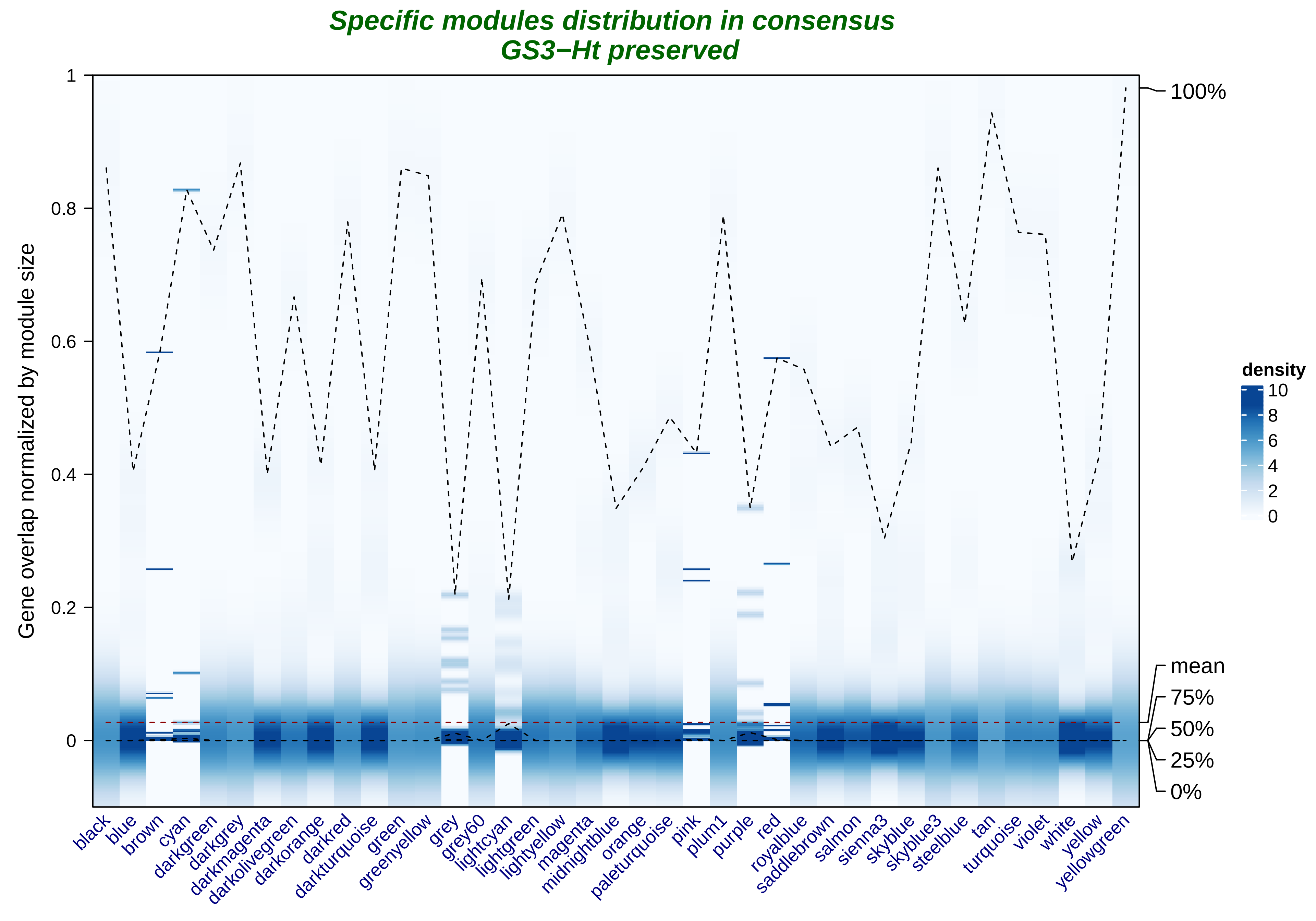

Specific modules distribution in consensus
GS3−Ht preserved
Gene overlap normalized by module size
1
0.8
0.6
0.4
0.2
0
100%
mean
75%
50%
25%
0%
density
10
8
6
4
2
0
black
blue
brown
cyan
darkgreen
darkgrey
darkmagenta
darkolivegreen
darkorange
darkred
darkturquoise
green
greenyellow
grey
grey60
lightcyan
lightgreen
lightyellow
magenta
midnightblue
orange
paleturquoise
pink
plum1
purple
red
royalblue
saddlebrown
salmon
sienna3
skyblue
skyblue3
steelblue
tan
turquoise
violet
white
yellow
yellowgreen

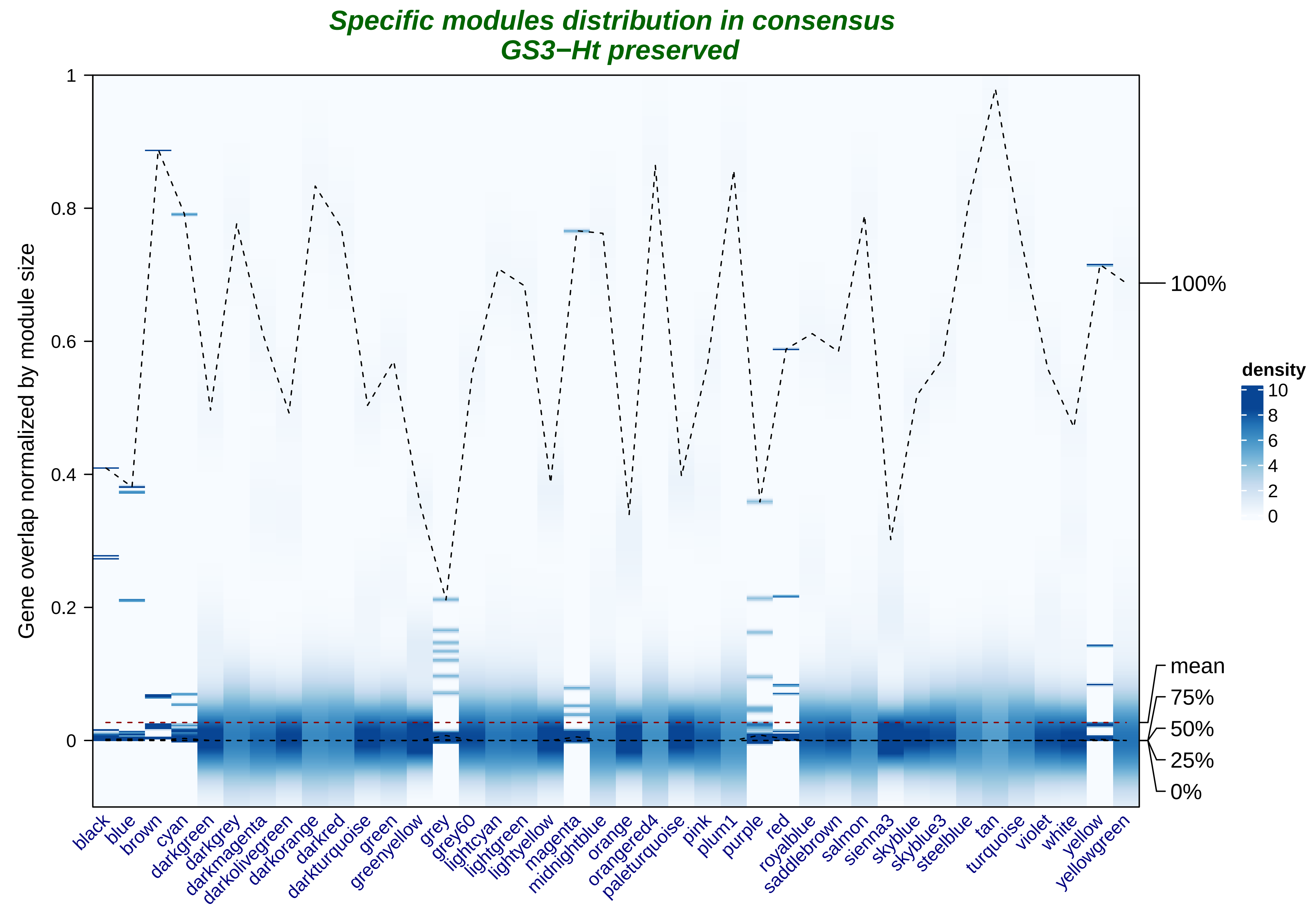

Specific modules distribution in consensus
GS3−Ht preserved
Gene overlap normalized by module size
1
0.8
0.6
0.4
0.2
0
100%
mean
75%
50%
25%
0%
density
10
8
6
4
2
0
black
blue
brown
cyan
darkgreen
darkgrey
darkmagenta
darkolivegreen
darkorange
darkred
darkturquoise
green
greenyellow
grey
grey60
lightcyan
lightgreen
lightyellow
magenta
midnightblue
orange
orangered4
paleturquoise
pink
plum1
purple
red
royalblue
saddlebrown
salmon
sienna3
skyblue
skyblue3
steelblue
tan
turquoise
violet
white
yellow
yellowgreen

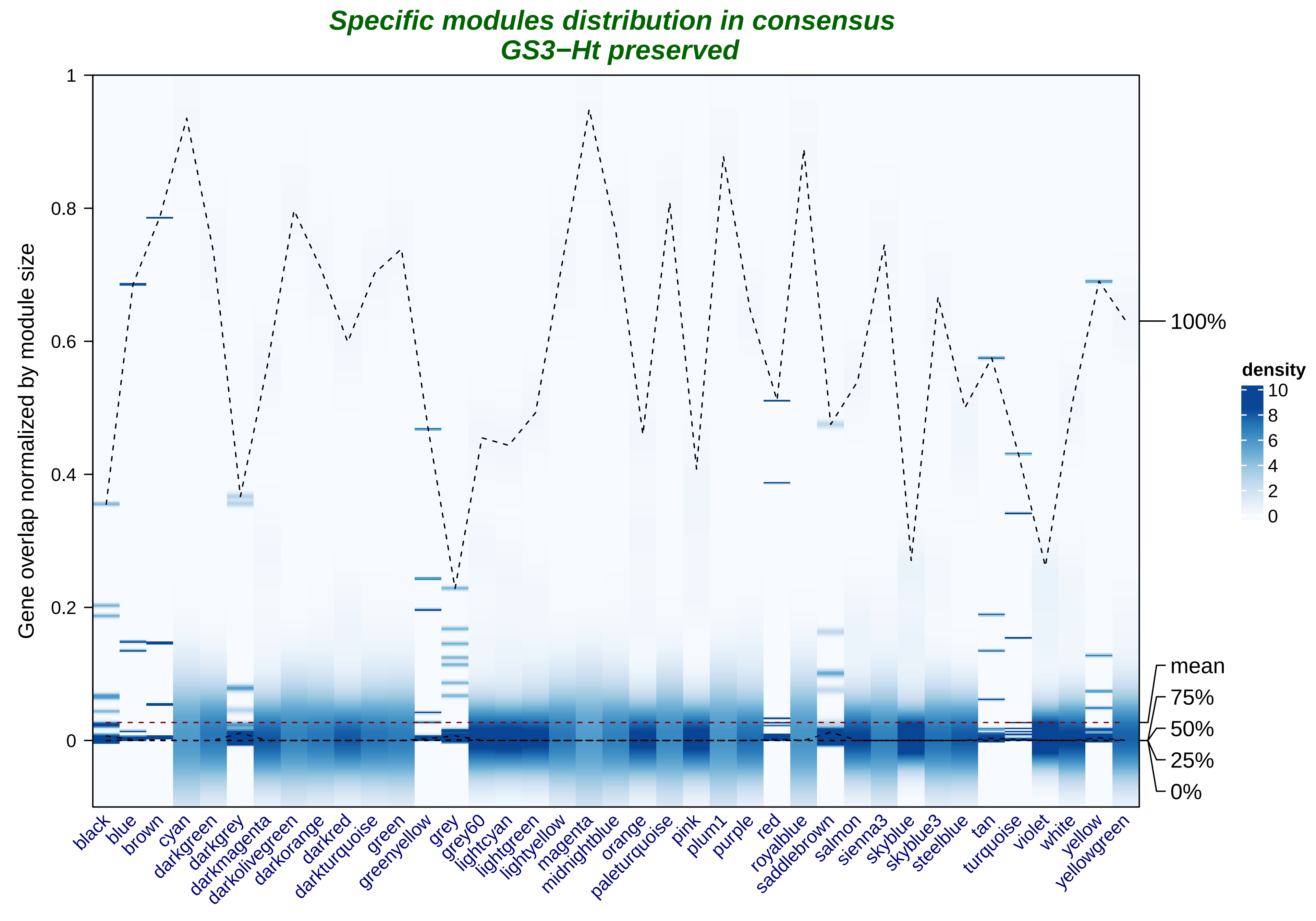

Specific modules distribution in consensus
GS3−Ht preserved
Gene overlap normalized by module size
1
0.8
0.6
0.4
0.2
0
100%
mean
75%
50%
25%
0%
density
10
8
6
4
2
0
black
blue
brown
cyan
darkgreen
darkgrey
darkmagenta
darkolivegreen
darkorange
darkred
darkturquoise
green
greenyellow
grey
grey60
lightcyan
lightgreen
lightyellow
magenta
midnightblue
orange
paleturquoise
pink
plum1
purple
red
royalblue
saddlebrown
salmon
sienna3
skyblue
skyblue3
steelblue
tan
turquoise
violet
white
yellow
yellowgreen

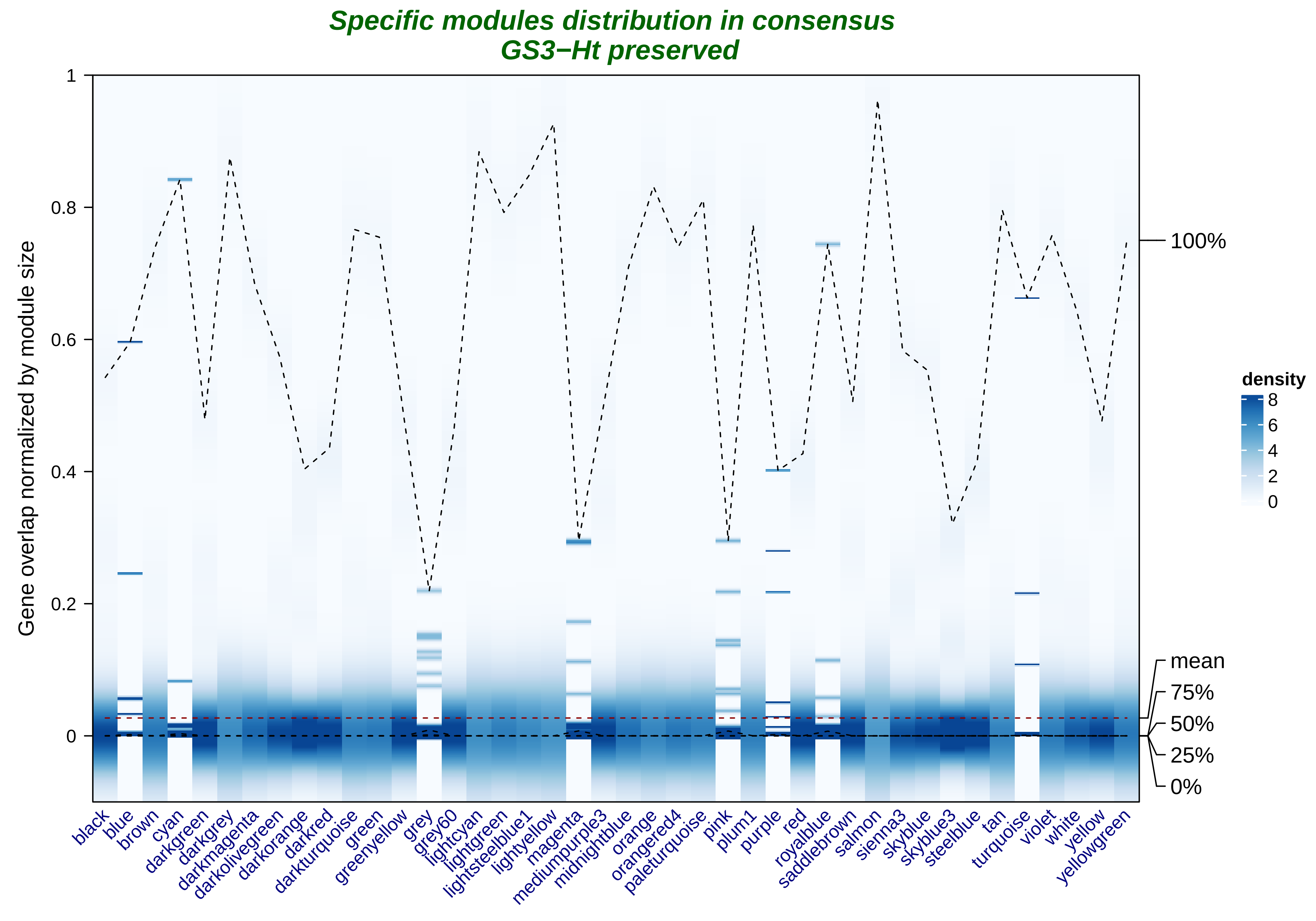

Specific modules distribution in consensus
GS3−Ht preserved
Gene overlap normalized by module size
1
0.8
0.6
0.4
0.2
0
100%
mean
75%
50%
25%
0%
density
8
6
4
2
0
black
blue
brown
cyan
darkgreen
darkgrey
darkmagenta
darkolivegreen
darkorange
darkred
darkturquoise
green
greenyellow
grey
grey60
lightcyan
lightgreen
lightsteelblue1
lightyellow
magenta
mediumpurple3
midnightblue
orange
orangered4
paleturquoise
pink
plum1
purple
red
royalblue
saddlebrown
salmon
sienna3
skyblue
skyblue3
steelblue
tan
turquoise
violet
white
yellow
yellowgreen

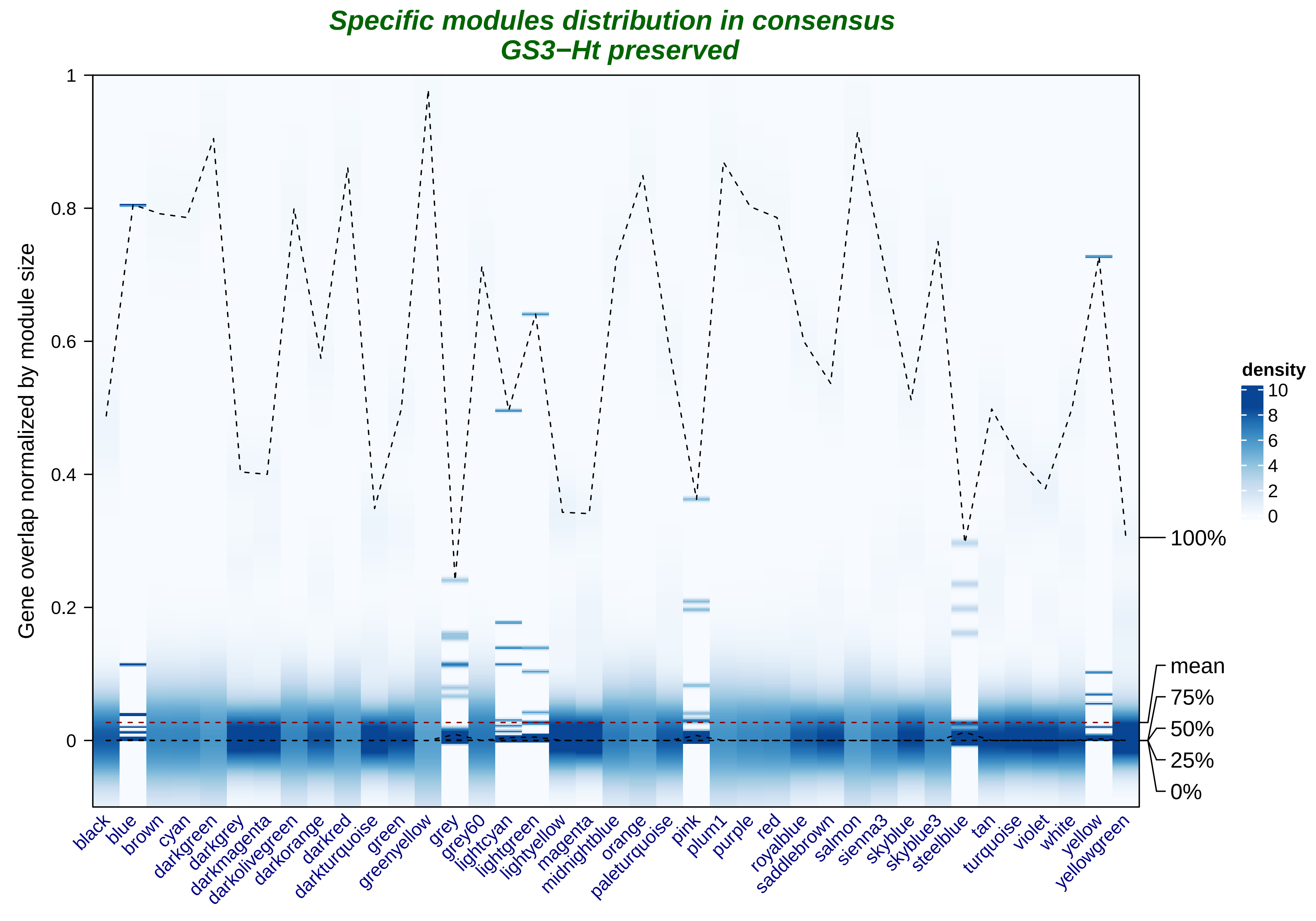

Specific modules distribution in consensus
GS3−Ht preserved
Gene overlap normalized by module size
1
0.8
0.6
0.4
0.2
0
density
10
8
6
4
2
0
100%
mean
75%
50%
25%
0%
black
blue
brown
cyan
darkgreen
darkgrey
darkmagenta
darkolivegreen
darkorange
darkred
darkturquoise
green
greenyellow
grey
grey60
lightcyan
lightgreen
lightyellow
magenta
midnightblue
orange
paleturquoise
pink
plum1
purple
red
royalblue
saddlebrown
salmon
sienna3
skyblue
skyblue3
steelblue
tan
turquoise
violet
white
yellow
yellowgreen

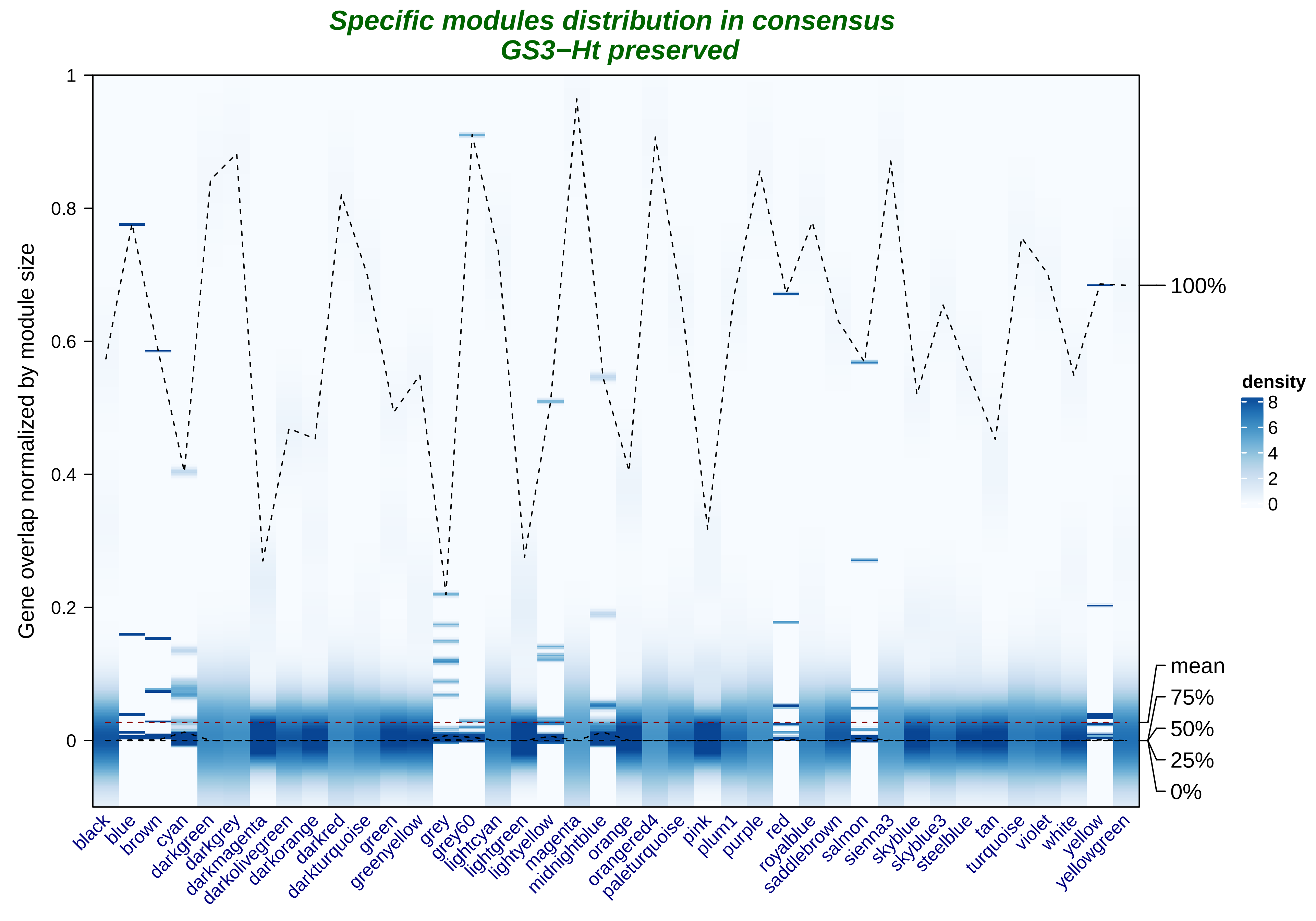

Specific modules distribution in consensus
GS3−Ht preserved
Gene overlap normalized by module size
1
0.8
0.6
0.4
0.2
0
100%
mean
75%
50%
25%
0%
density
8
6
4
2
0
black
blue
brown
cyan
darkgreen
darkgrey
darkmagenta
darkolivegreen
darkorange
darkred
darkturquoise
green
greenyellow
grey
grey60
lightcyan
lightgreen
lightyellow
magenta
midnightblue
orange
orangered4
paleturquoise
pink
plum1
purple
red
royalblue
saddlebrown
salmon
sienna3
skyblue
skyblue3
steelblue
tan
turquoise
violet
white
yellow
yellowgreen

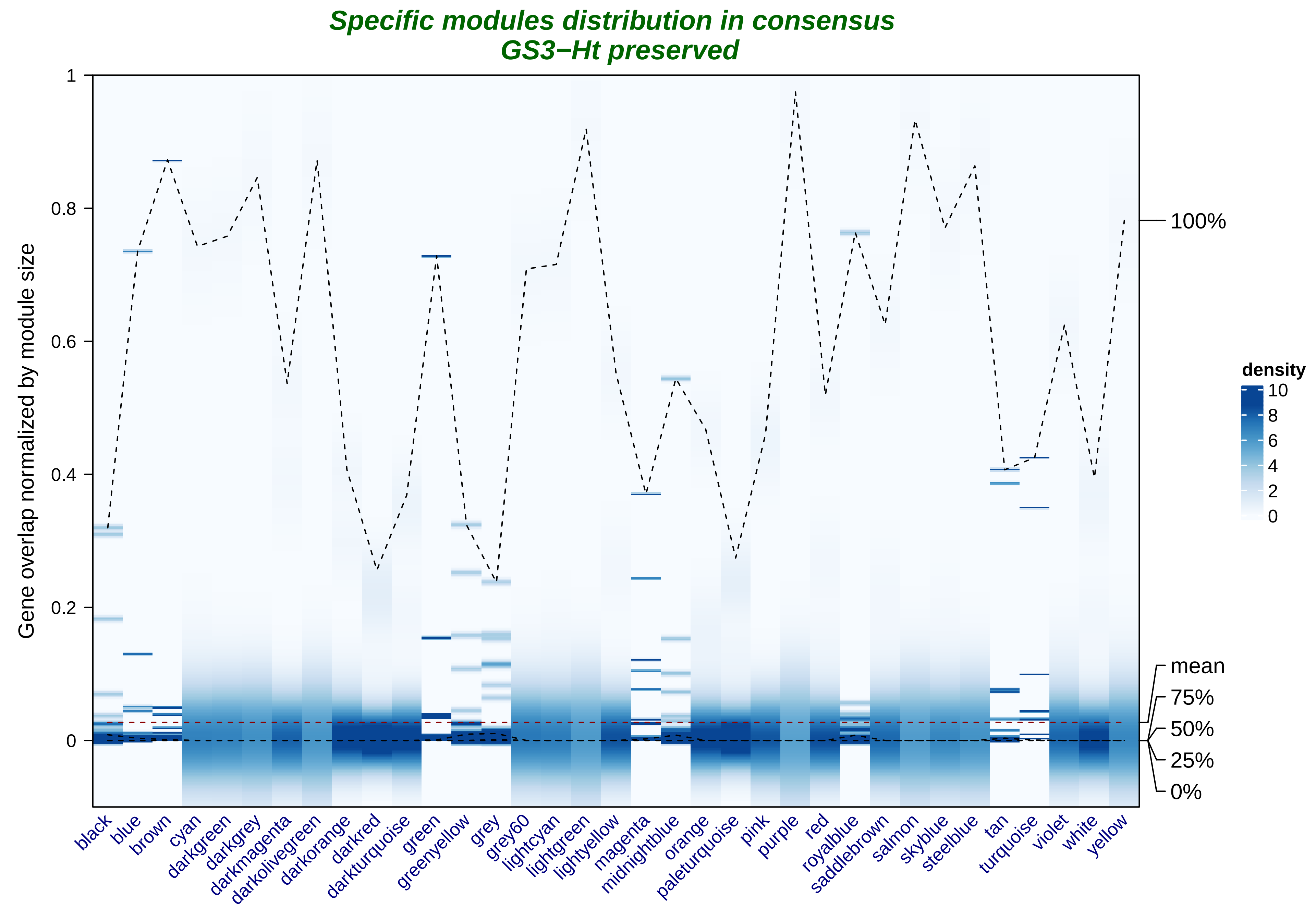

Specific modules distribution in consensus
GS3−Ht preserved
Gene overlap normalized by module size
1
0.8
0.6
0.4
0.2
0
100%
mean
75%
50%
25%
0%
density
10
8
6
4
2
0
black
blue
brown
cyan
darkgreen
darkgrey
darkmagenta
darkolivegreen
darkorange
darkred
darkturquoise
green
greenyellow
grey
grey60
lightcyan
lightgreen
lightyellow
magenta
midnightblue
orange
paleturquoise
pink
purple
red
royalblue
saddlebrown
salmon
skyblue
steelblue
tan
turquoise
violet
white
yellow

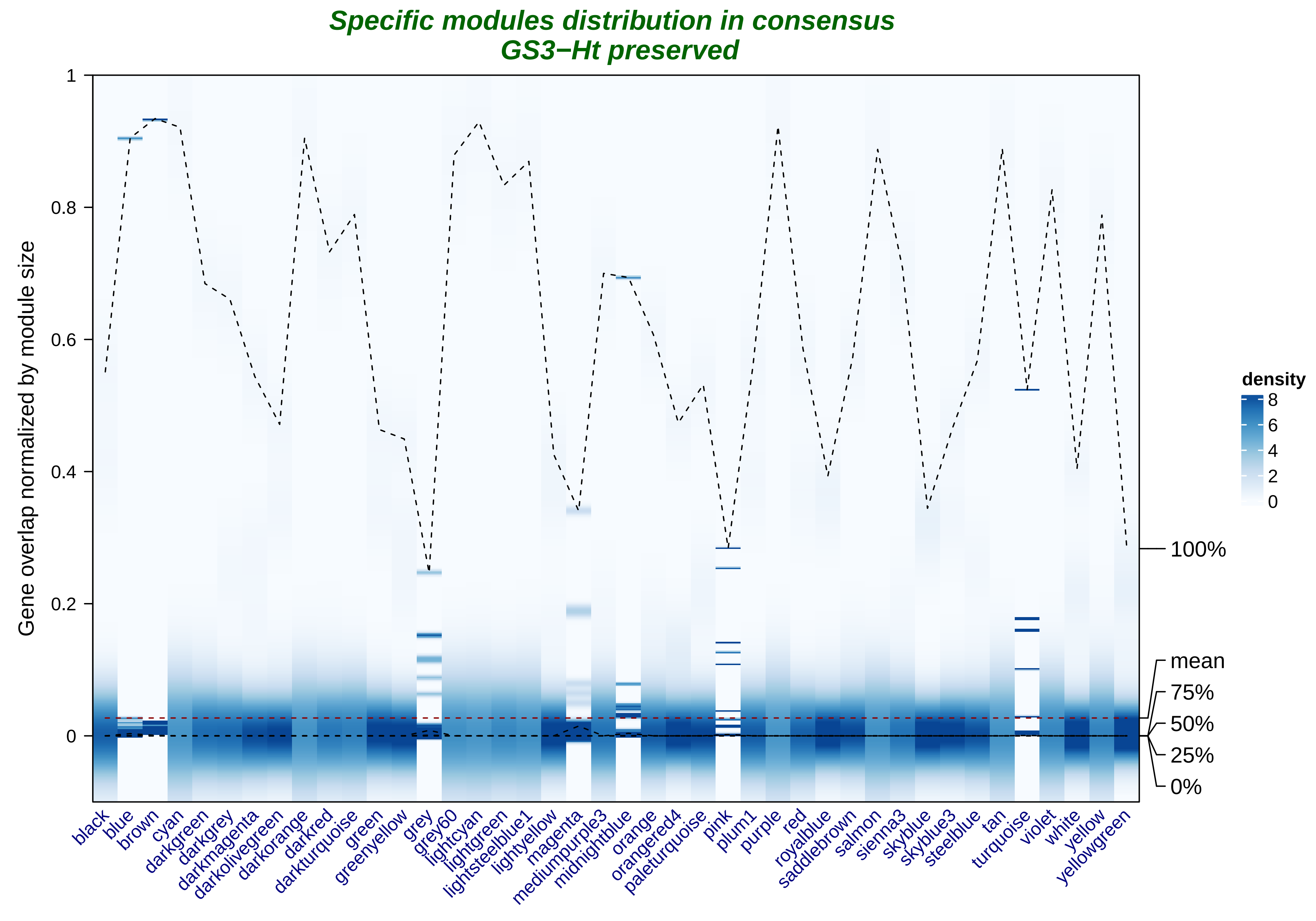

Specific modules distribution in consensus
GS3−Ht preserved
Gene overlap normalized by module size
1
0.8
0.6
0.4
0.2
0
density
8
6
4
2
0
100%
mean
75%
50%
25%
0%
black
blue
brown
cyan
darkgreen
darkgrey
darkmagenta
darkolivegreen
darkorange
darkred
darkturquoise
green
greenyellow
grey
grey60
lightcyan
lightgreen
lightsteelblue1
lightyellow
magenta
mediumpurple3
midnightblue
orange
orangered4
paleturquoise
pink
plum1
purple
red
royalblue
saddlebrown
salmon
sienna3
skyblue
skyblue3
steelblue
tan
turquoise
violet
white
yellow
yellowgreen

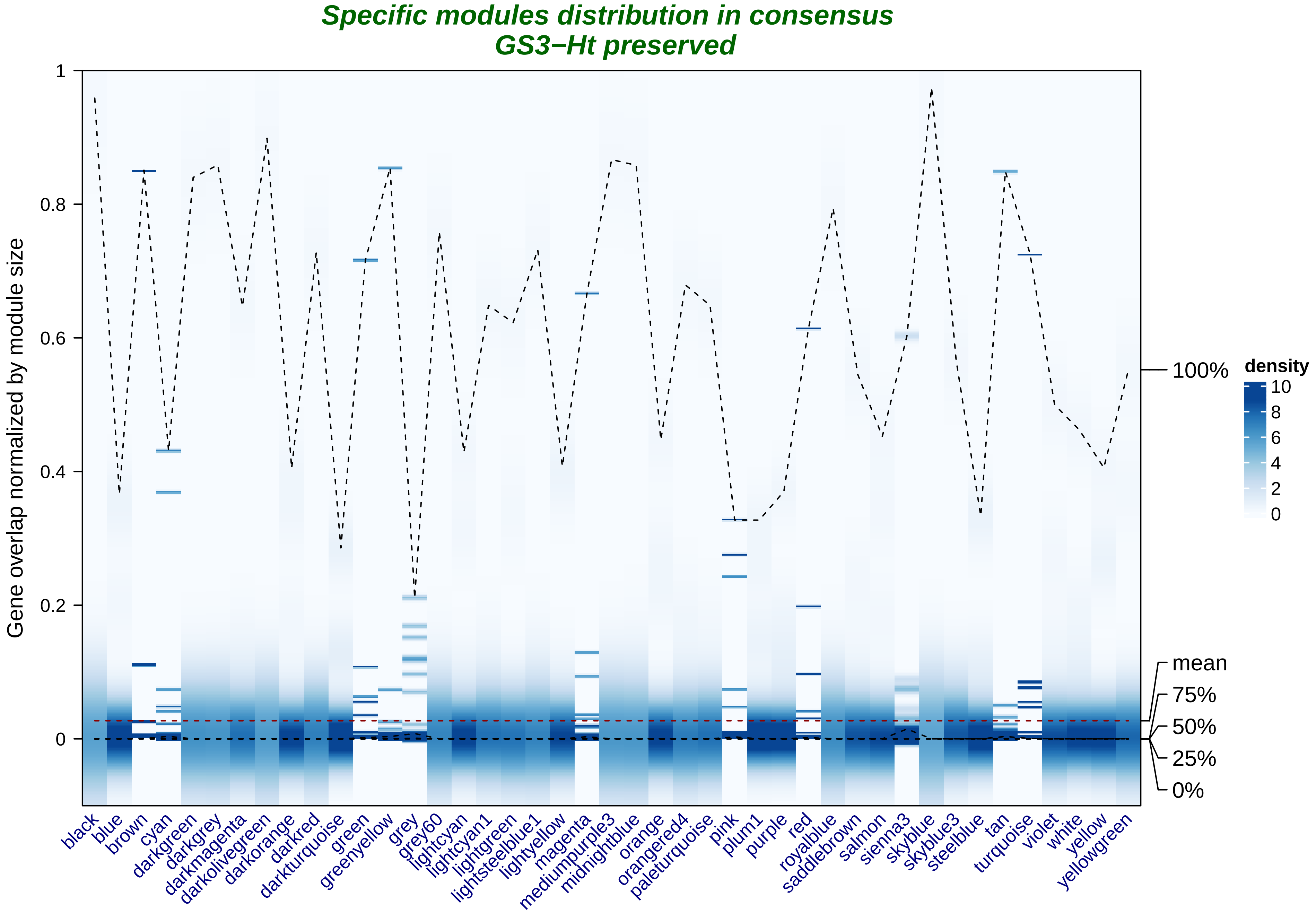

Specific modules distribution in consensus
GS3−Ht preserved
Gene overlap normalized by module size
1
0.8
0.6
0.4
0.2
0
100%
mean
75%
50%
25%
0%
density
10
8
6
4
2
0
black
blue
brown
cyan
darkgreen
darkgrey
darkmagenta
darkolivegreen
darkorange
darkred
darkturquoise
green
greenyellow
grey
grey60
lightcyan
lightcyan1
lightgreen
lightsteelblue1
lightyellow
magenta
mediumpurple3
midnightblue
orange
orangered4
paleturquoise
pink
plum1
purple
red
royalblue
saddlebrown
salmon
sienna3
skyblue
skyblue3
steelblue
tan
turquoise
violet
white
yellow
yellowgreen

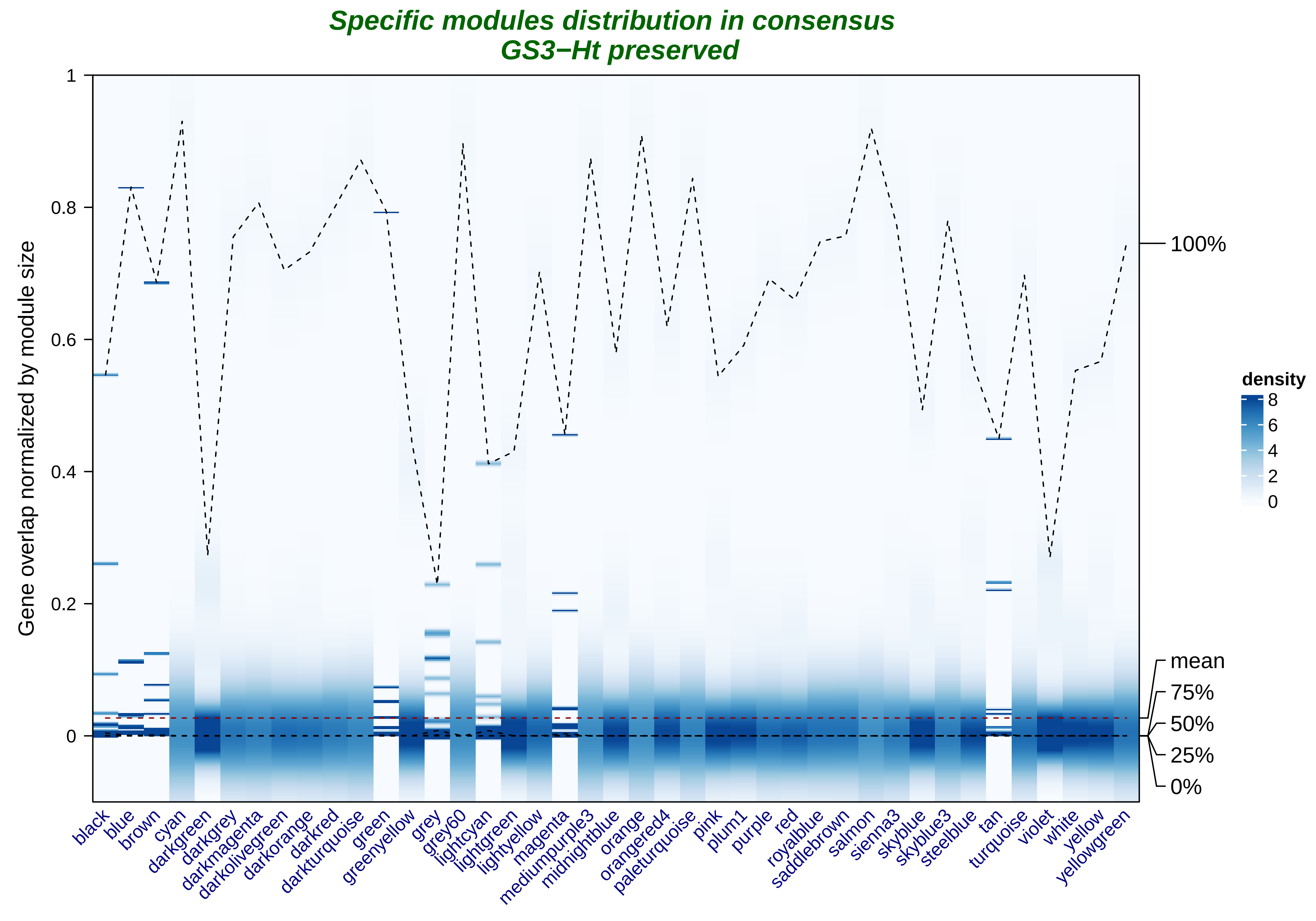
Specific modules distribution in consensus
GS3−Ht preserved
Gene overlap normalized by module size
1
0.8
0.6
0.4
0.2
0
density
8
6
4
2
0
100%
mean
75%
50%
25%
0%
black
blue
brown
cyan
darkgreen
darkgrey
darkmagenta
darkolivegreen
darkorange
darkred
darkturquoise
green
greenyellow
grey
grey60
lightcyan
lightgreen
lightyellow
magenta
mediumpurple3
midnightblue
orange
orangered4
paleturquoise
pink
plum1
purple
red
royalblue
saddlebrown
salmon
sienna3
skyblue
skyblue3
steelblue
tan
turquoise
violet
white
yellow
yellowgreen

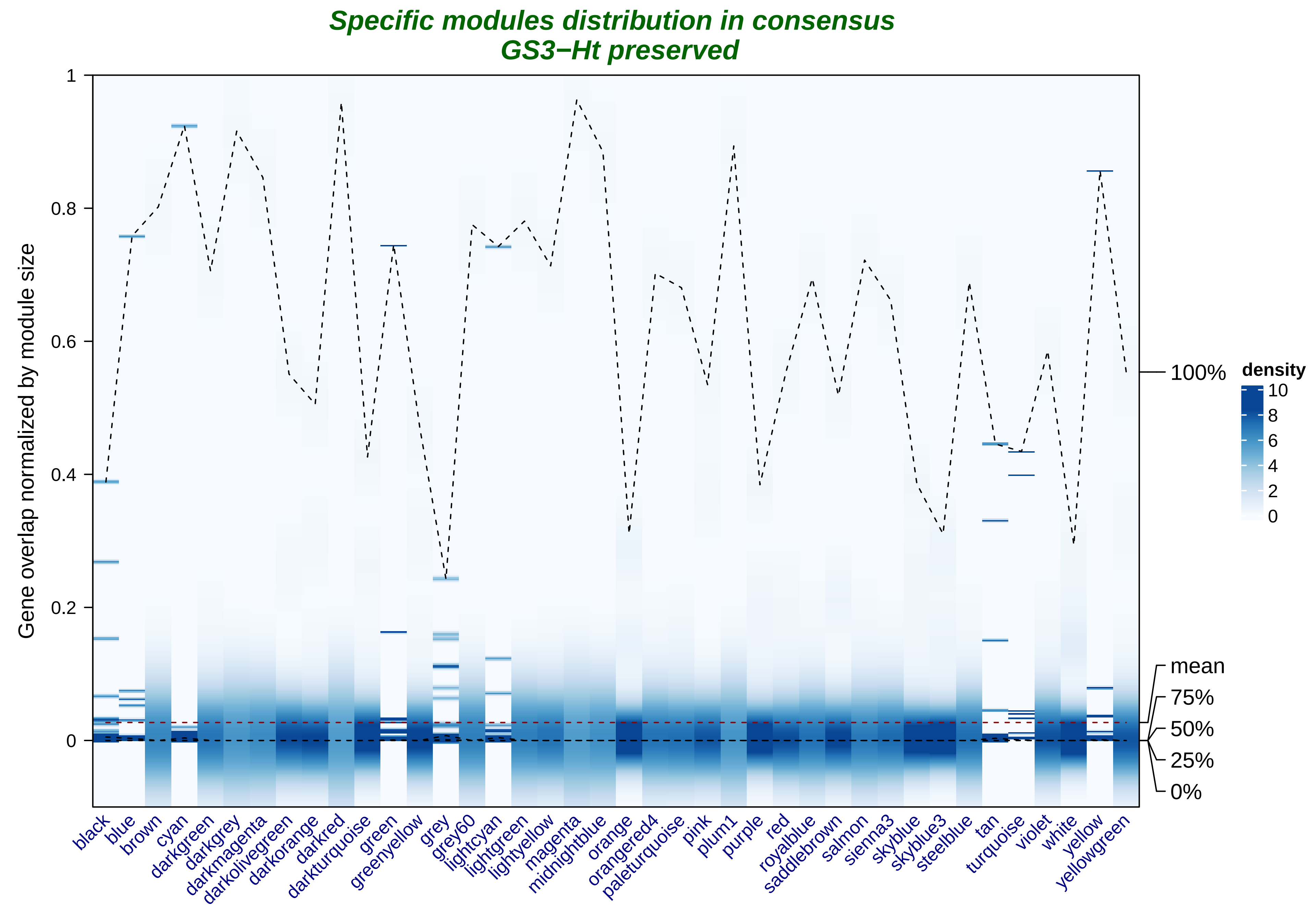

Specific modules distribution in consensus
GS3−Ht preserved
Gene overlap normalized by module size
1
0.8
0.6
0.4
0.2
0
100%
mean
75%
50%
25%
0%
density
10
8
6
4
2
0
black
blue
brown
cyan
darkgreen
darkgrey
darkmagenta
darkolivegreen
darkorange
darkred
darkturquoise
green
greenyellow
grey
grey60
lightcyan
lightgreen
lightyellow
magenta
midnightblue
orange
orangered4
paleturquoise
pink
plum1
purple
red
royalblue
saddlebrown
salmon
sienna3
skyblue
skyblue3
steelblue
tan
turquoise
violet
white
yellow
yellowgreen

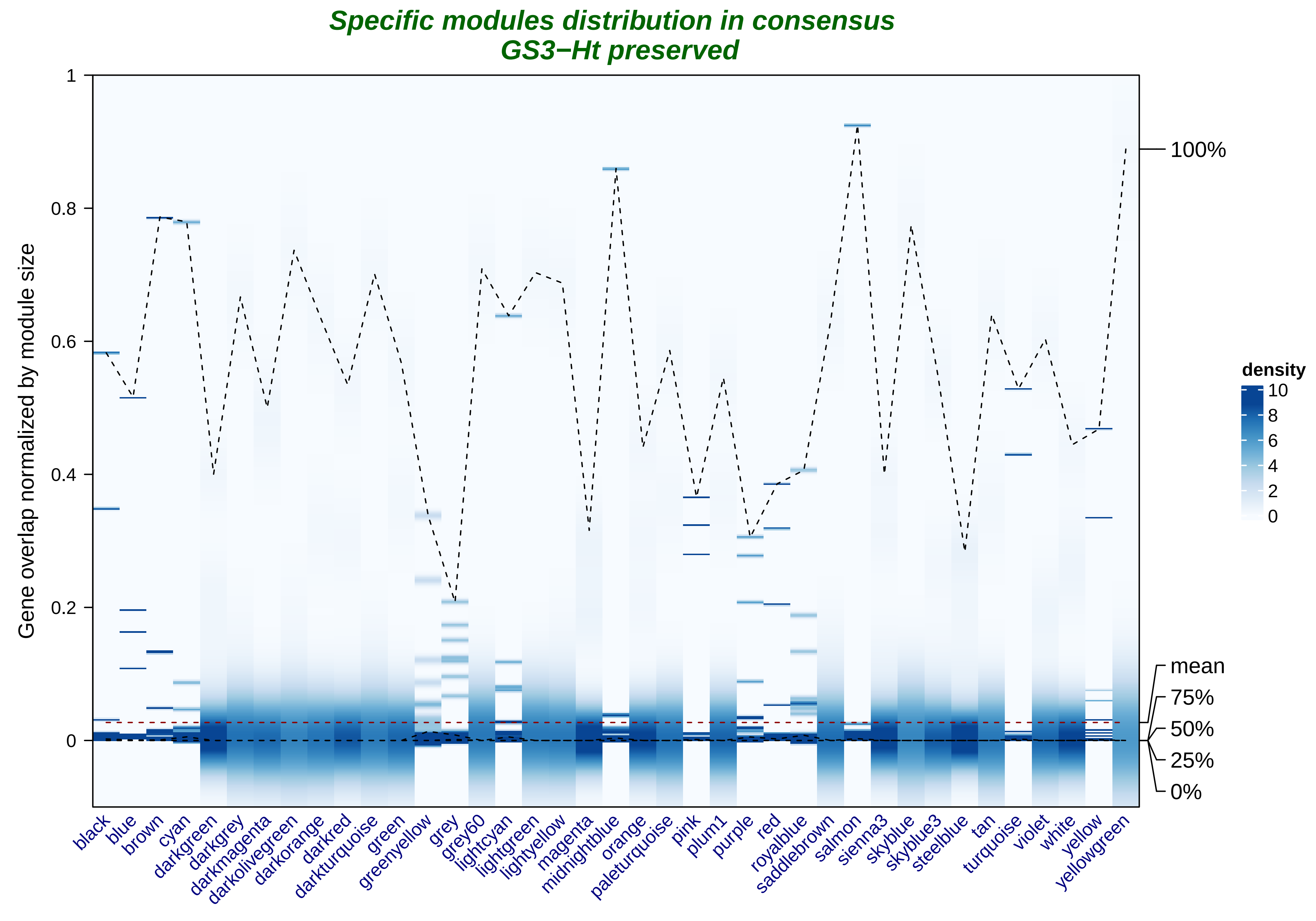

Specific modules distribution in consensus
GS3−Ht preserved
Gene overlap normalized by module size
1
0.8
0.6
0.4
0.2
0
100%
mean
75%
50%
25%
0%
density
10
8
6
4
2
0
black
blue
brown
cyan
darkgreen
darkgrey
darkmagenta
darkolivegreen
darkorange
darkred
darkturquoise
green
greenyellow
grey
grey60
lightcyan
lightgreen
lightyellow
magenta
midnightblue
orange
paleturquoise
pink
plum1
purple
red
royalblue
saddlebrown
salmon
sienna3
skyblue
skyblue3
steelblue
tan
turquoise
violet
white
yellow
yellowgreen

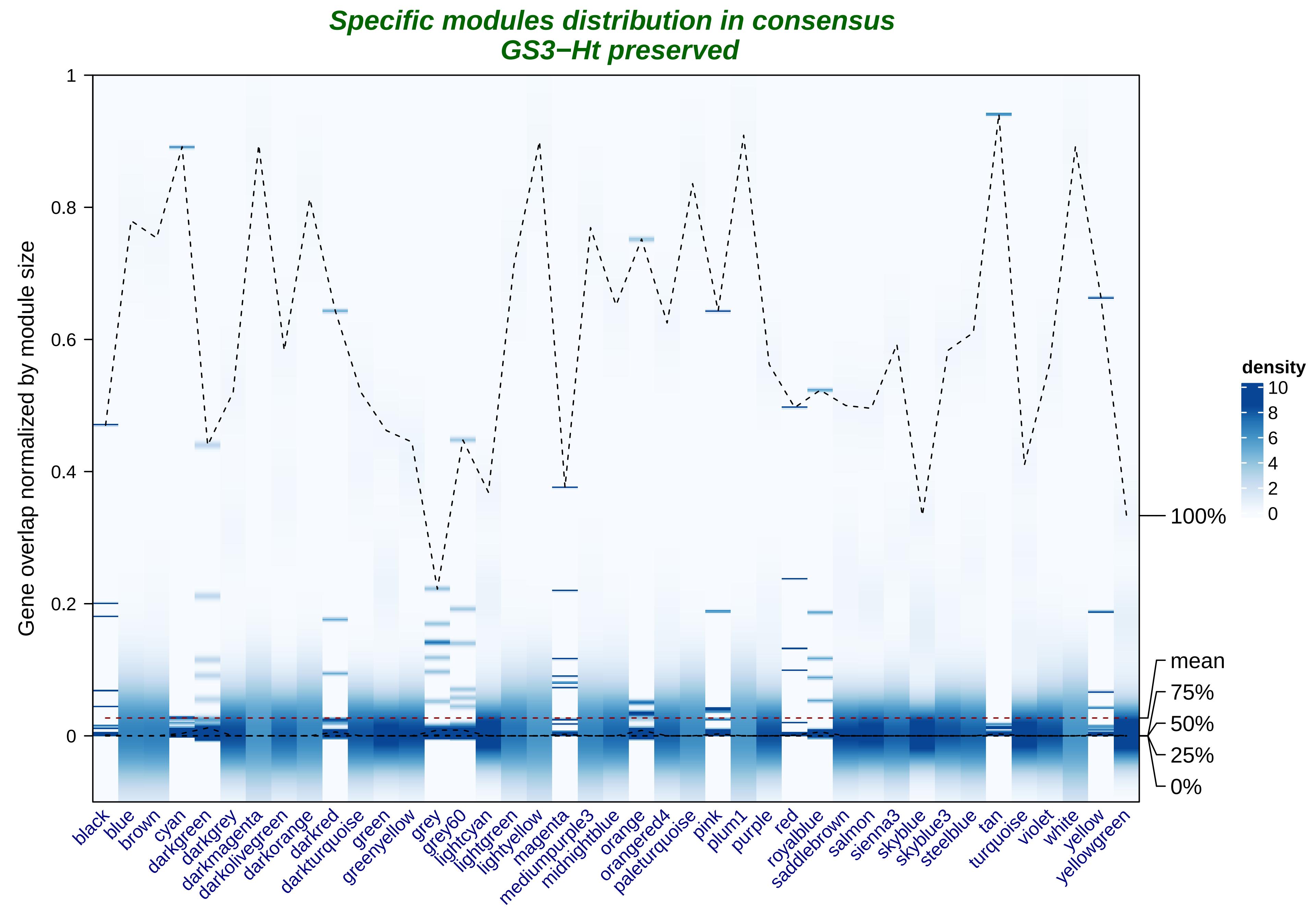

Specific modules distribution in consensus
GS3−Ht preserved
Gene overlap normalized by module size
1
0.8
0.6
0.4
0.2
0
density
10
8
6
4
2
0
100%
mean
75%
50%
25%
0%
black
blue
brown
cyan
darkgreen
darkgrey
darkmagenta
darkolivegreen
darkorange
darkred
darkturquoise
green
greenyellow
grey
grey60
lightcyan
lightgreen
lightyellow
magenta
mediumpurple3
midnightblue
orange
orangered4
paleturquoise
pink
plum1
purple
red
royalblue
saddlebrown
salmon
sienna3
skyblue
skyblue3
steelblue
tan
turquoise
violet
white
yellow
yellowgreen

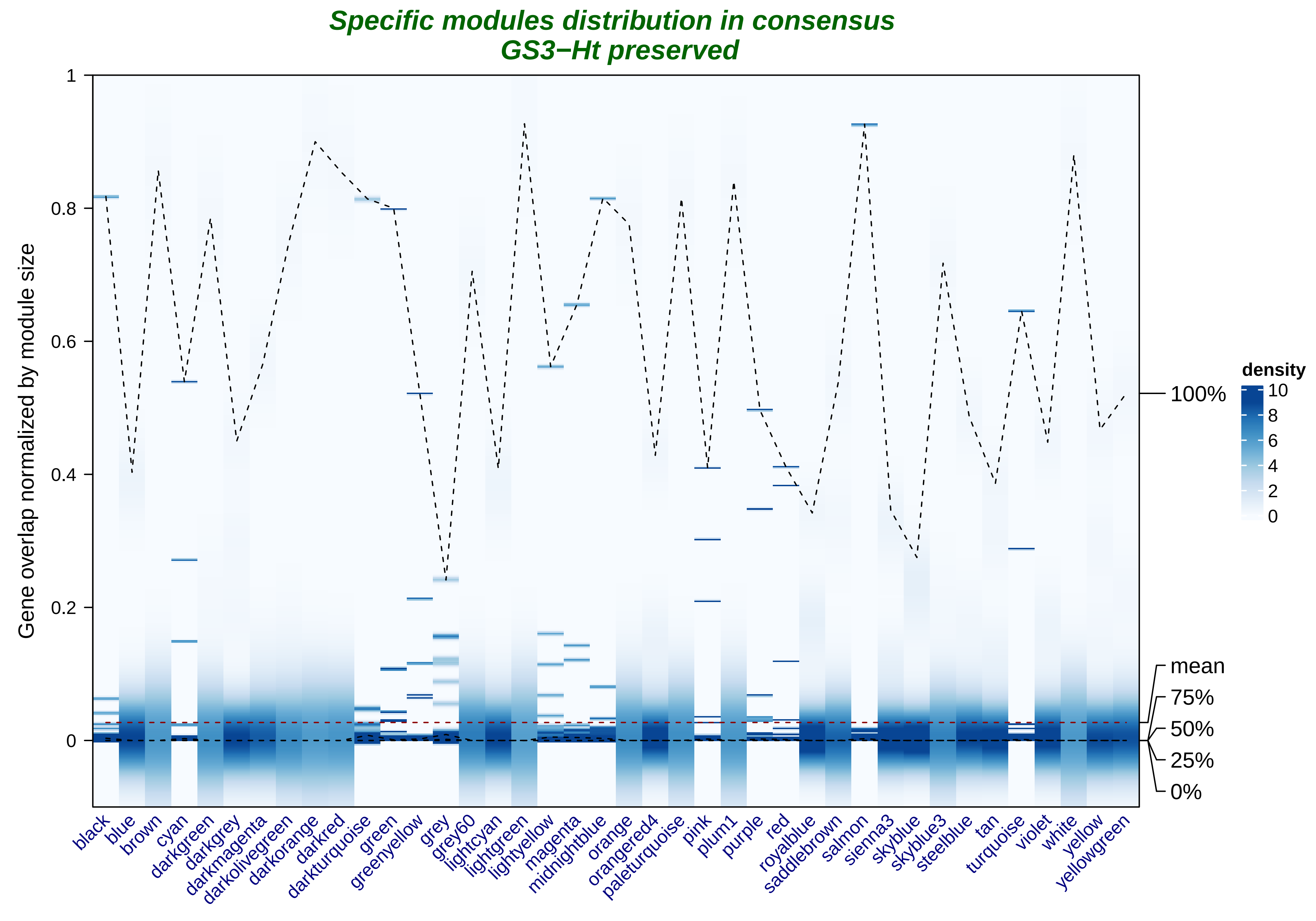

Specific modules distribution in consensus
GS3−Ht preserved
Gene overlap normalized by module size
1
0.8
0.6
0.4
0.2
0
density
10
8
6
4
2
0
100%
mean
75%
50%
25%
0%
black
blue
brown
cyan
darkgreen
darkgrey
darkmagenta
darkolivegreen
darkorange
darkred
darkturquoise
green
greenyellow
grey
grey60
lightcyan
lightgreen
lightyellow
magenta
midnightblue
orange
orangered4
paleturquoise
pink
plum1
purple
red
royalblue
saddlebrown
salmon
sienna3
skyblue
skyblue3
steelblue
tan
turquoise
violet
white
yellow
yellowgreen

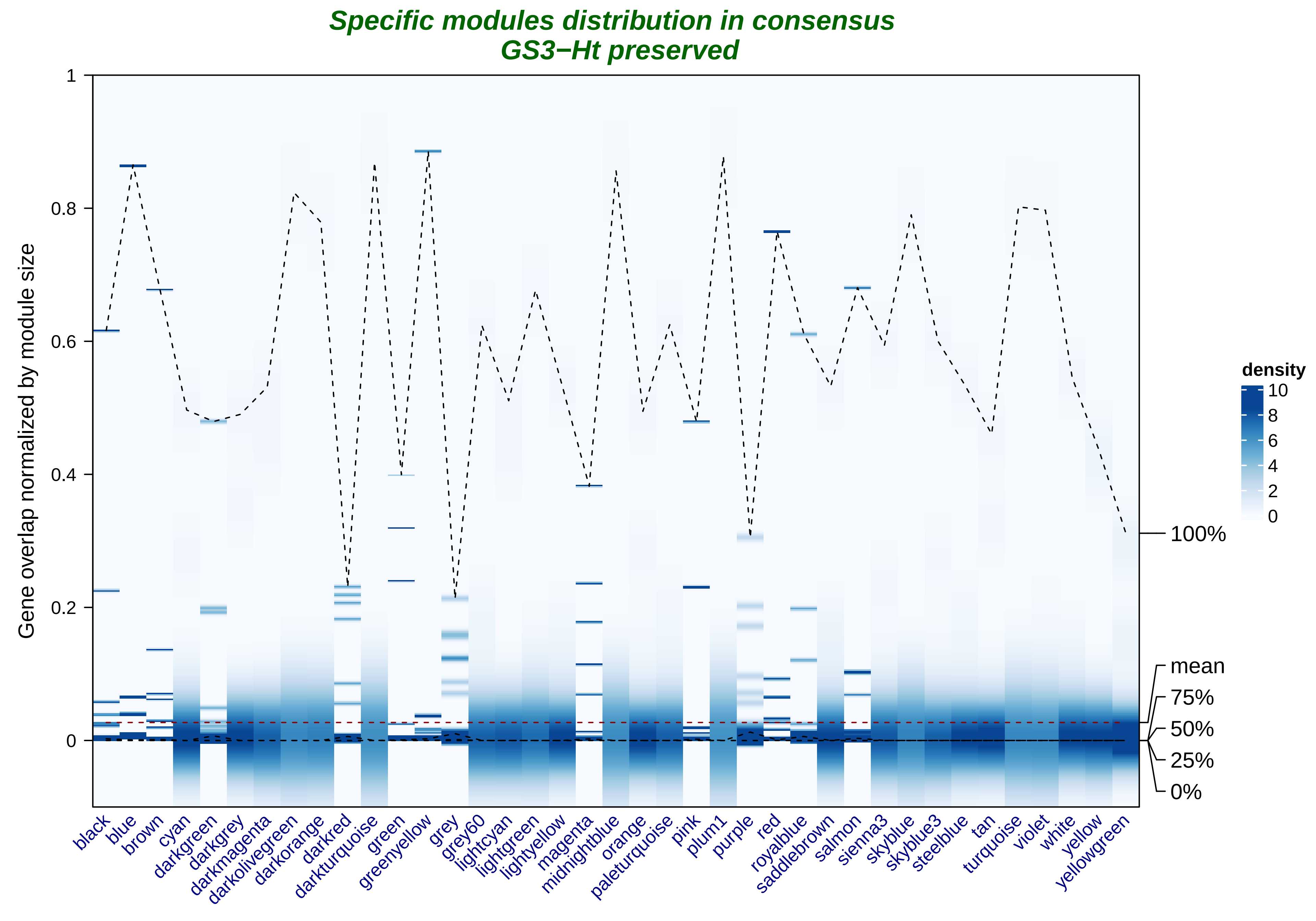
Specific modules distribution in consensus
GS3−Ht preserved
Gene overlap normalized by module size
1
0.8
0.6
0.4
0.2
0
density
10
8
6
4
2
0
100%
mean
75%
50%
25%
0%
black
blue
brown
cyan
darkgreen
darkgrey
darkmagenta
darkolivegreen
darkorange
darkred
darkturquoise
green
greenyellow
grey
grey60
lightcyan
lightgreen
lightyellow
magenta
midnightblue
orange
paleturquoise
pink
plum1
purple
red
royalblue
saddlebrown
salmon
sienna3
skyblue
skyblue3
steelblue
tan
turquoise
violet
white
yellow
yellowgreen

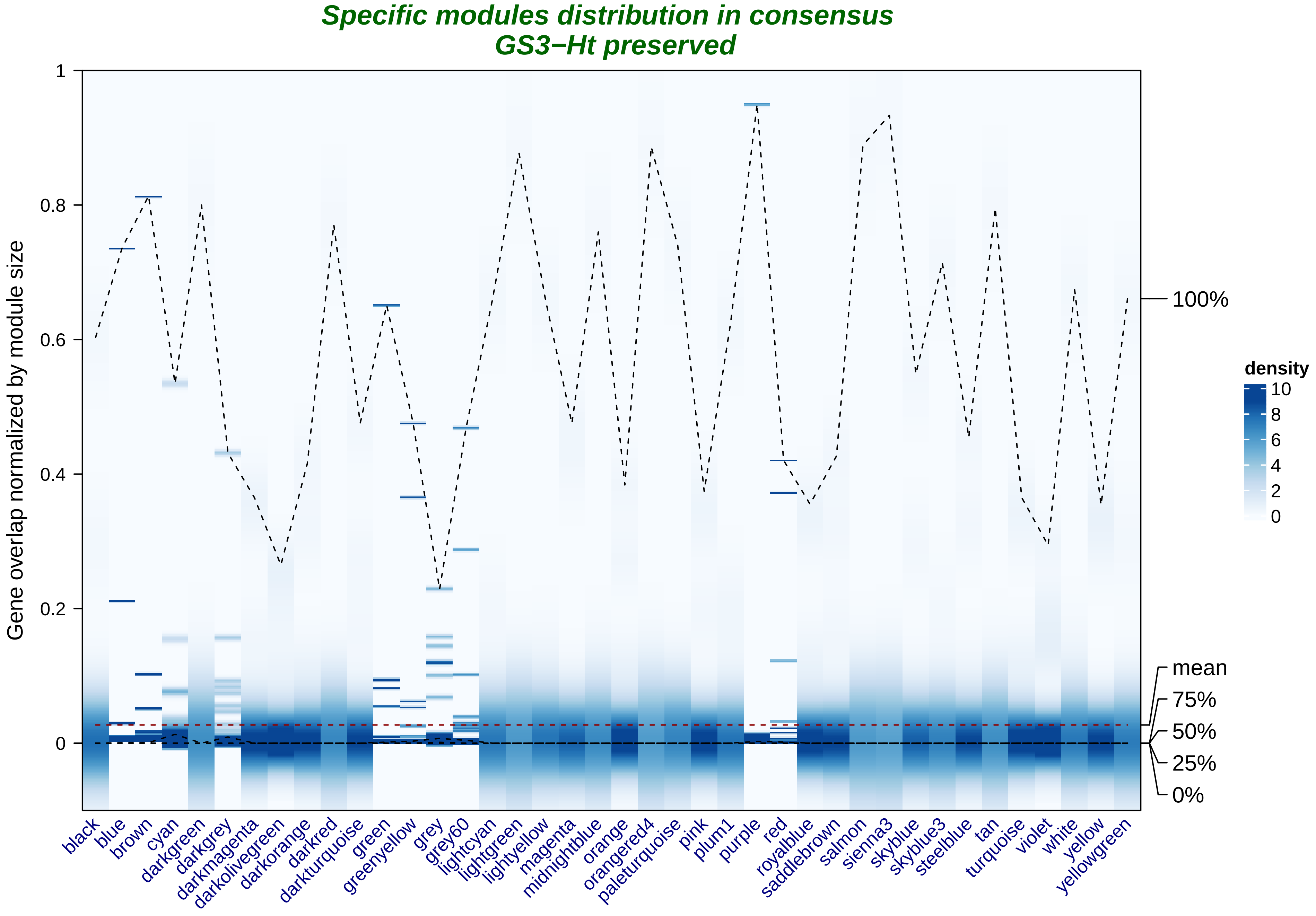

Specific modules distribution in consensus
GS3−Ht preserved
Gene overlap normalized by module size
1
0.8
0.6
0.4
0.2
0
100%
mean
75%
50%
25%
0%
density
10
8
6
4
2
0
black
blue
brown
cyan
darkgreen
darkgrey
darkmagenta
darkolivegreen
darkorange
darkred
darkturquoise
green
greenyellow
grey
grey60
lightcyan
lightgreen
lightyellow
magenta
midnightblue
orange
orangered4
paleturquoise
pink
plum1
purple
red
royalblue
saddlebrown
salmon
sienna3
skyblue
skyblue3
steelblue
tan
turquoise
violet
white
yellow
yellowgreen

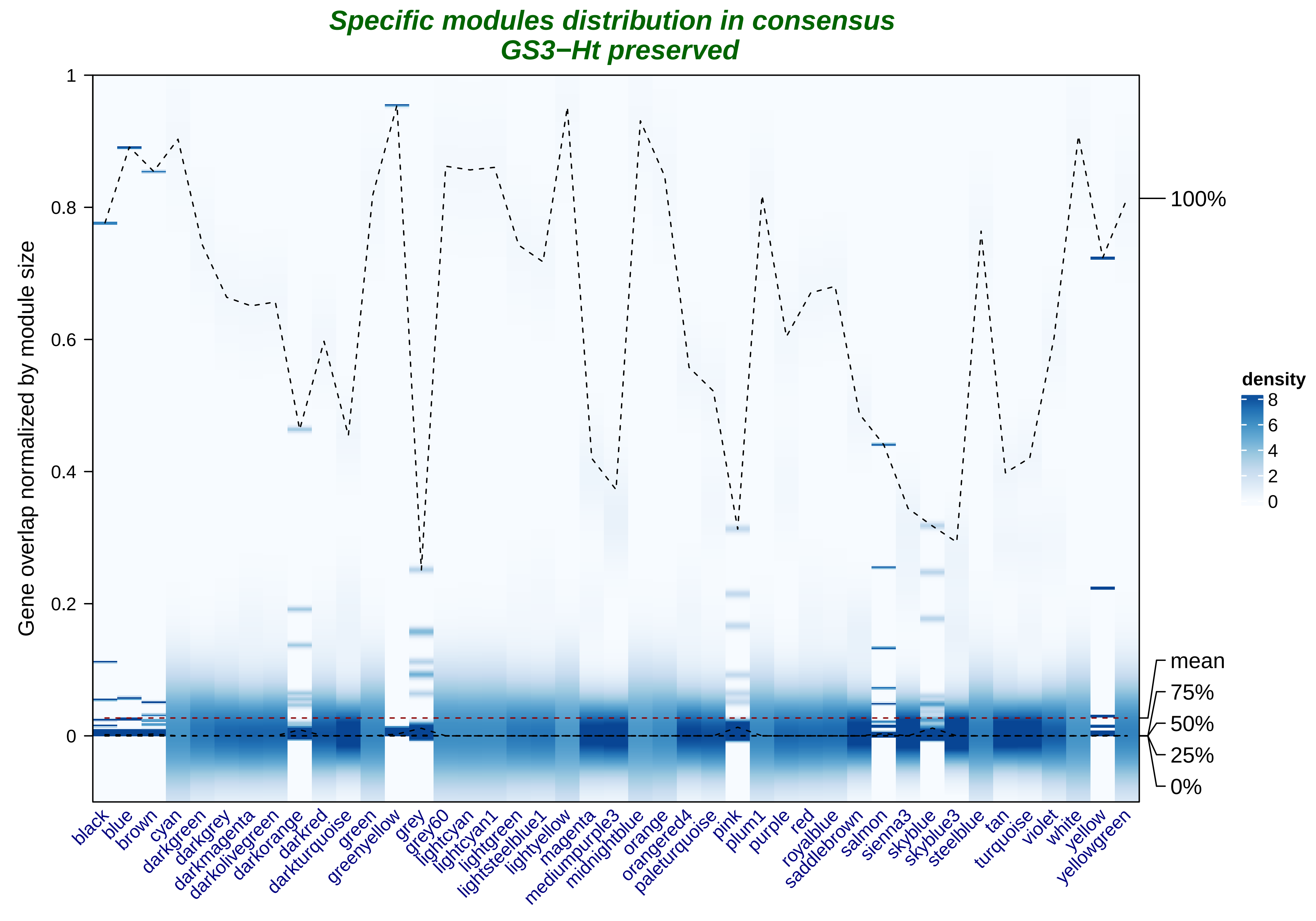

Specific modules distribution in consensus
GS3−Ht preserved
Gene overlap normalized by module size
1
0.8
0.6
0.4
0.2
0
100%
mean
75%
50%
25%
0%
density
8
6
4
2
0
black
blue
brown
cyan
darkgreen
darkgrey
darkmagenta
darkolivegreen
darkorange
darkred
darkturquoise
green
greenyellow
grey
grey60
lightcyan
lightcyan1
lightgreen
lightsteelblue1
lightyellow
magenta
mediumpurple3
midnightblue
orange
orangered4
paleturquoise
pink
plum1
purple
red
royalblue
saddlebrown
salmon
sienna3
skyblue
skyblue3
steelblue
tan
turquoise
violet
white
yellow
yellowgreen

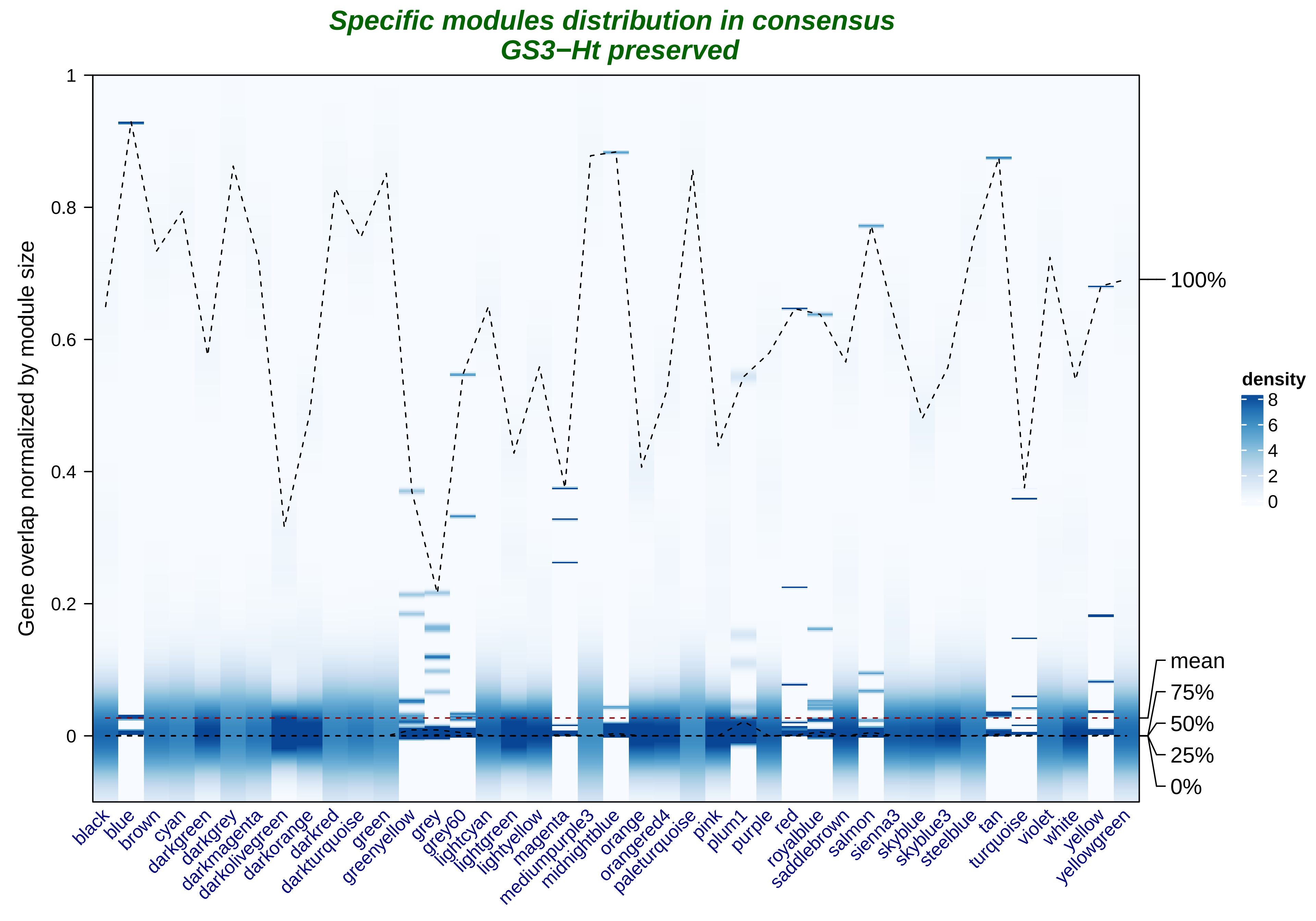

Specific modules distribution in consensus
GS3−Ht preserved
Gene overlap normalized by module size
1
0.8
0.6
0.4
0.2
0
100%
mean
75%
50%
25%
0%
density
8
6
4
2
0
black
blue
brown
cyan
darkgreen
darkgrey
darkmagenta
darkolivegreen
darkorange
darkred
darkturquoise
green
greenyellow
grey
grey60
lightcyan
lightgreen
lightyellow
magenta
mediumpurple3
midnightblue
orange
orangered4
paleturquoise
pink
plum1
purple
red
royalblue
saddlebrown
salmon
sienna3
skyblue
skyblue3
steelblue
tan
turquoise
violet
white
yellow
yellowgreen

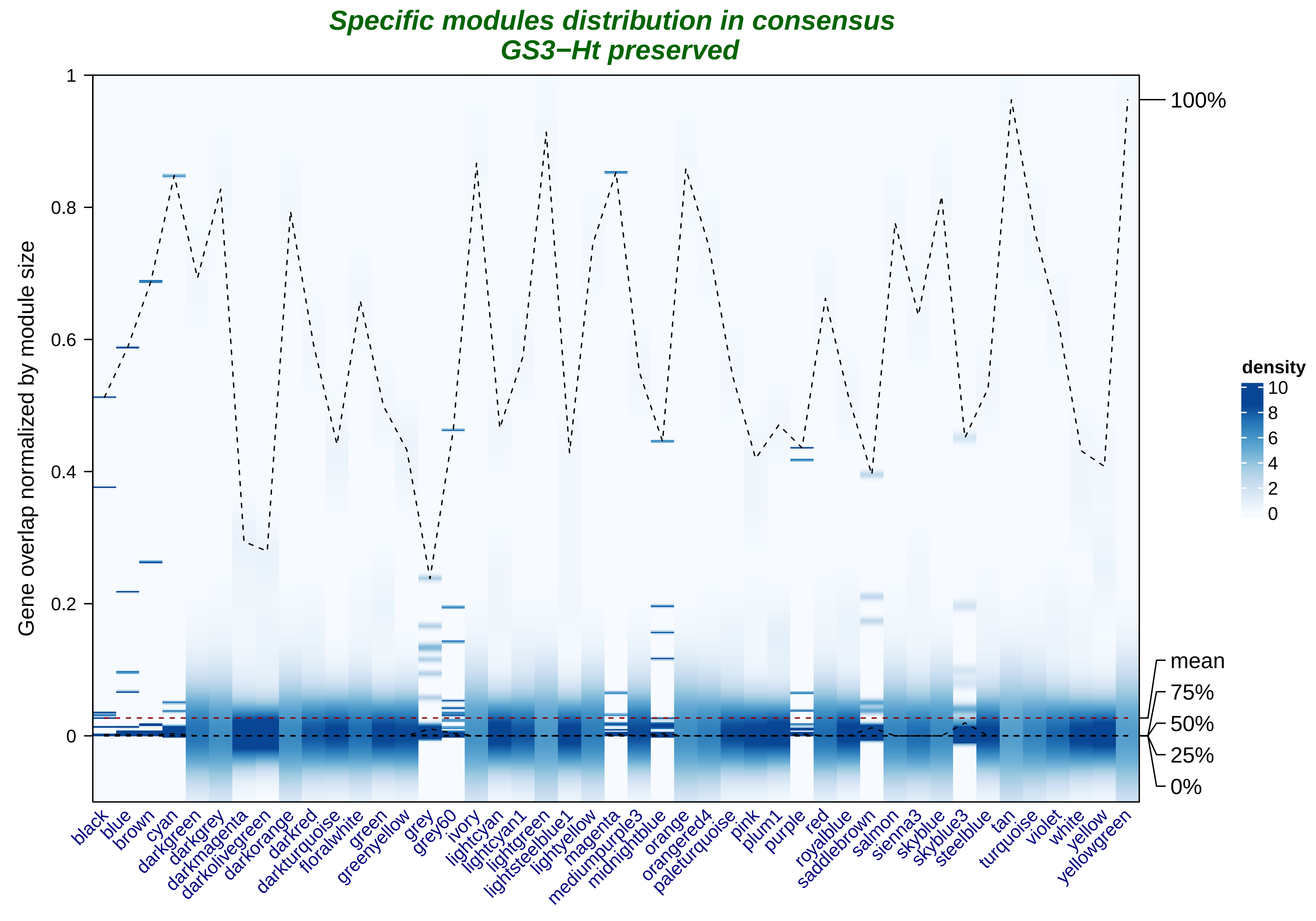
Specific modules distribution in consensus
GS3−Ht preserved
Gene overlap normalized by module size
1
0.8
0.6
0.4
0.2
0
100%
mean
75%
50%
25%
0%
density
10
8
6
4
2
0
black
blue
brown
cyan
darkgreen
darkgrey
darkmagenta
darkolivegreen
darkorange
darkred
darkturquoise
floralwhite
green
greenyellow
grey
grey60
ivory
lightcyan
lightcyan1
lightgreen
lightsteelblue1
lightyellow
magenta
mediumpurple3
midnightblue
orange
orangered4
paleturquoise
pink
plum1
purple
red
royalblue
saddlebrown
salmon
sienna3
skyblue
skyblue3
steelblue
tan
turquoise
violet
white
yellow
yellowgreen

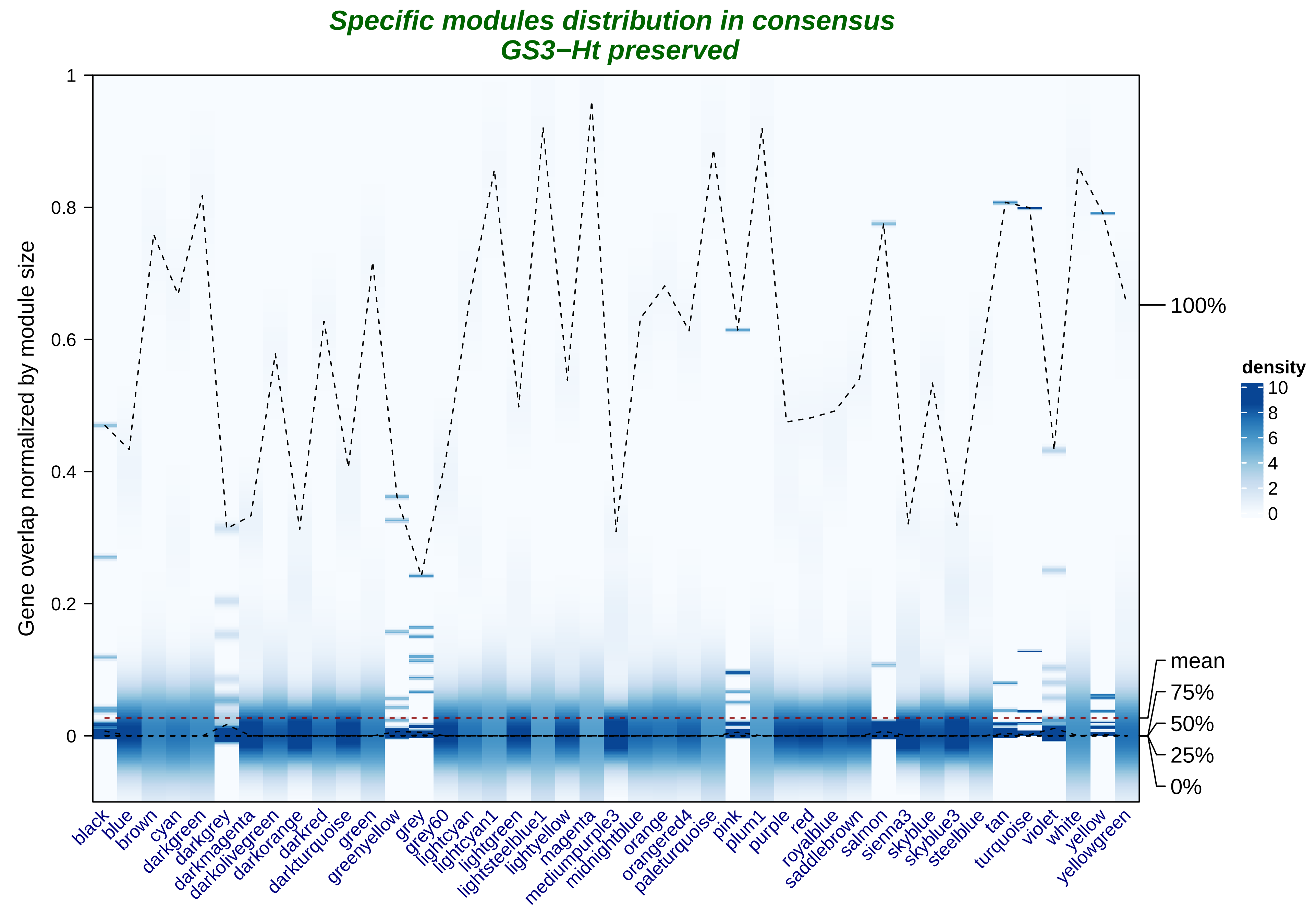

Specific modules distribution in consensus
GS3−Ht preserved
Gene overlap normalized by module size
1
0.8
0.6
0.4
0.2
0
100%
mean
75%
50%
25%
0%
density
10
8
6
4
2
0
black
blue
brown
cyan
darkgreen
darkgrey
darkmagenta
darkolivegreen
darkorange
darkred
darkturquoise
green
greenyellow
grey
grey60
lightcyan
lightcyan1
lightgreen
lightsteelblue1
lightyellow
magenta
mediumpurple3
midnightblue
orange
orangered4
paleturquoise
pink
plum1
purple
red
royalblue
saddlebrown
salmon
sienna3
skyblue
skyblue3
steelblue
tan
turquoise
violet
white
yellow
yellowgreen

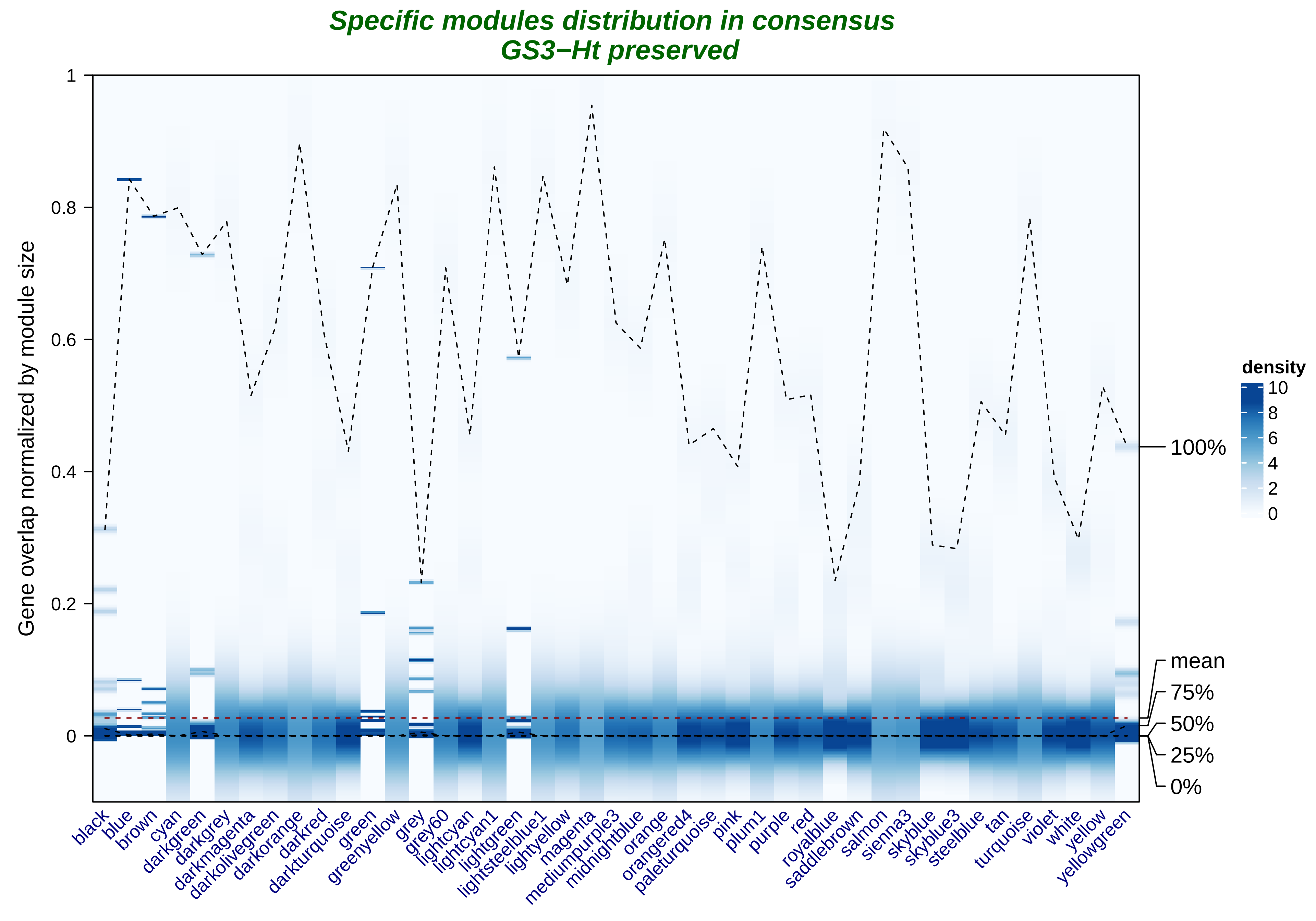

Specific modules distribution in consensus
GS3−Ht preserved
Gene overlap normalized by module size
1
0.8
0.6
0.4
0.2
0
density
10
8
6
4
2
0
100%
mean
75%
50%
25%
0%
black
blue
brown
cyan
darkgreen
darkgrey
darkmagenta
darkolivegreen
darkorange
darkred
darkturquoise
green
greenyellow
grey
grey60
lightcyan
lightcyan1
lightgreen
lightsteelblue1
lightyellow
magenta
mediumpurple3
midnightblue
orange
orangered4
paleturquoise
pink
plum1
purple
red
royalblue
saddlebrown
salmon
sienna3
skyblue
skyblue3
steelblue
tan
turquoise
violet
white
yellow
yellowgreen

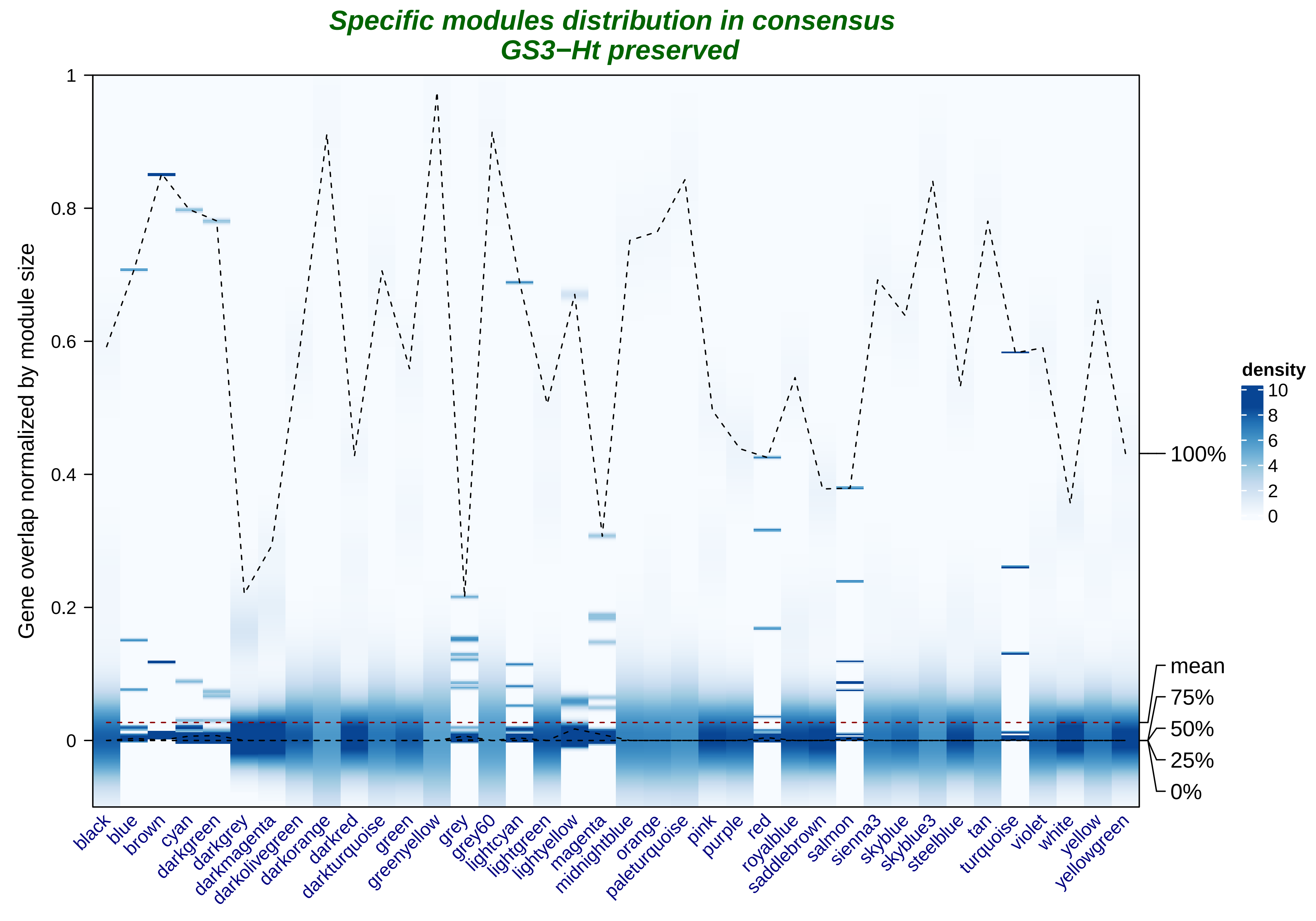

Specific modules distribution in consensus
GS3−Ht preserved
Gene overlap normalized by module size
1
0.8
0.6
0.4
0.2
0
density
10
8
6
4
2
0
100%
mean
75%
50%
25%
0%
black
blue
brown
cyan
darkgreen
darkgrey
darkmagenta
darkolivegreen
darkorange
darkred
darkturquoise
green
greenyellow
grey
grey60
lightcyan
lightgreen
lightyellow
magenta
midnightblue
orange
paleturquoise
pink
purple
red
royalblue
saddlebrown
salmon
sienna3
skyblue
skyblue3
steelblue
tan
turquoise
violet
white
yellow
yellowgreen

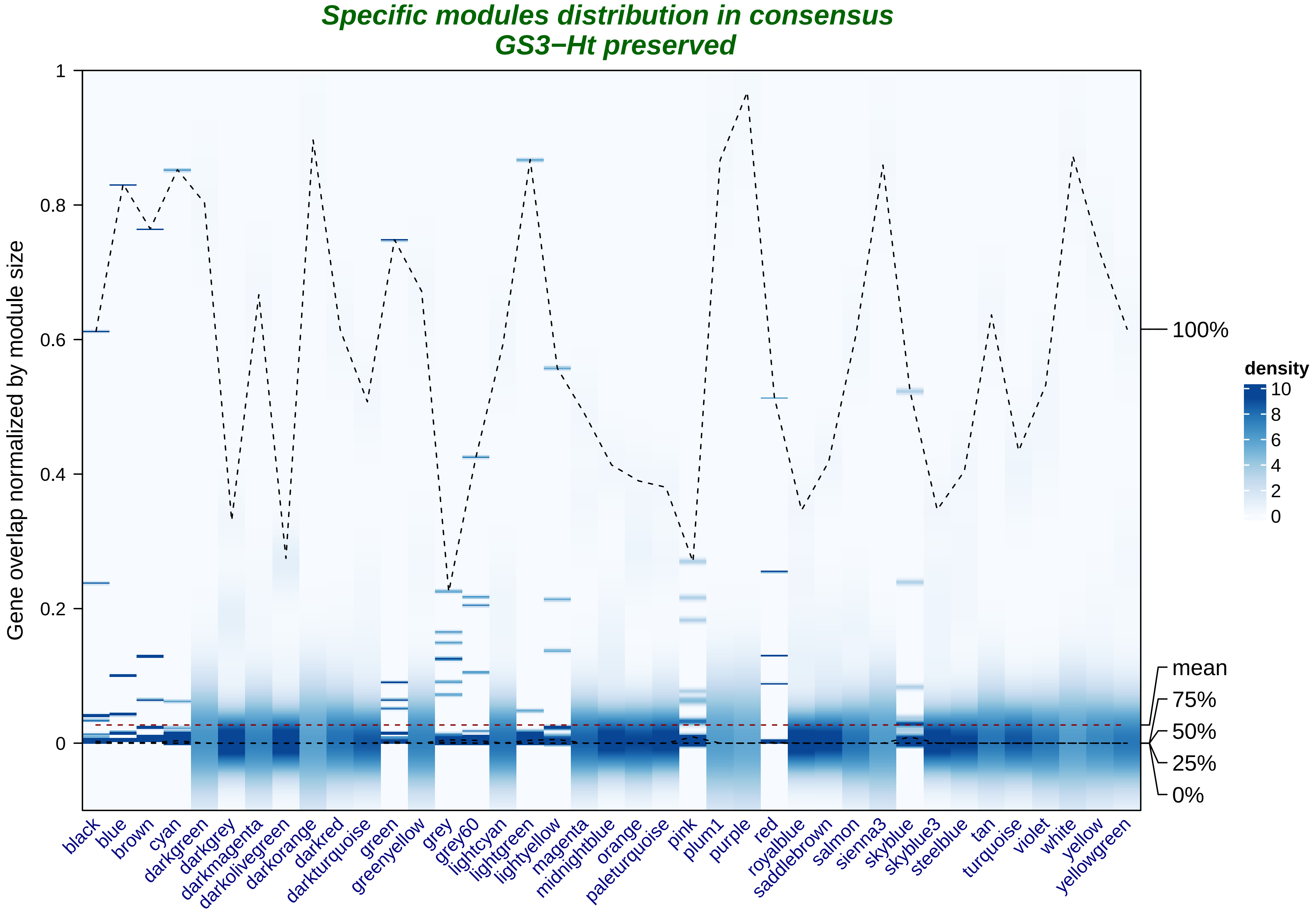

Specific modules distribution in consensus
GS3−Ht preserved
Gene overlap normalized by module size
1
0.8
0.6
0.4
0.2
0
100%
mean
75%
50%
25%
0%
density
10
8
6
4
2
0
black
blue
brown
cyan
darkgreen
darkgrey
darkmagenta
darkolivegreen
darkorange
darkred
darkturquoise
green
greenyellow
grey
grey60
lightcyan
lightgreen
lightyellow
magenta
midnightblue
orange
paleturquoise
pink
plum1
purple
red
royalblue
saddlebrown
salmon
sienna3
skyblue
skyblue3
steelblue
tan
turquoise
violet
white
yellow
yellowgreen

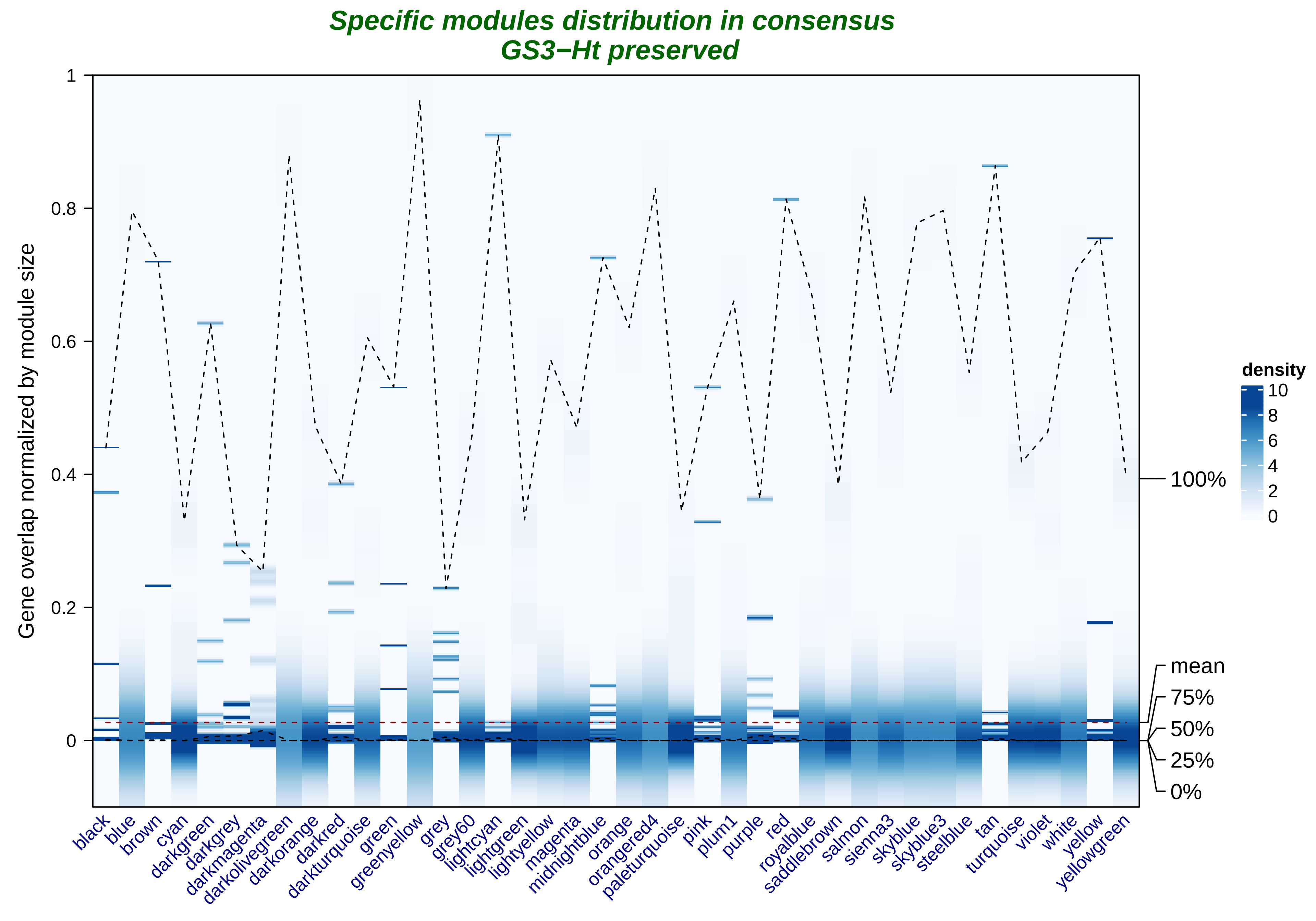
Specific modules distribution in consensus
GS3−Ht preserved
Gene overlap normalized by module size
1
0.8
0.6
0.4
0.2
0
100%
mean
75%
50%
25%
0%
density
10
8
6
4
2
0
black
blue
brown
cyan
darkgreen
darkgrey
darkmagenta
darkolivegreen
darkorange
darkred
darkturquoise
green
greenyellow
grey
grey60
lightcyan
lightgreen
lightyellow
magenta
midnightblue
orange
orangered4
paleturquoise
pink
plum1
purple
red
royalblue
saddlebrown
salmon
sienna3
skyblue
skyblue3
steelblue
tan
turquoise
violet
white
yellow
yellowgreen

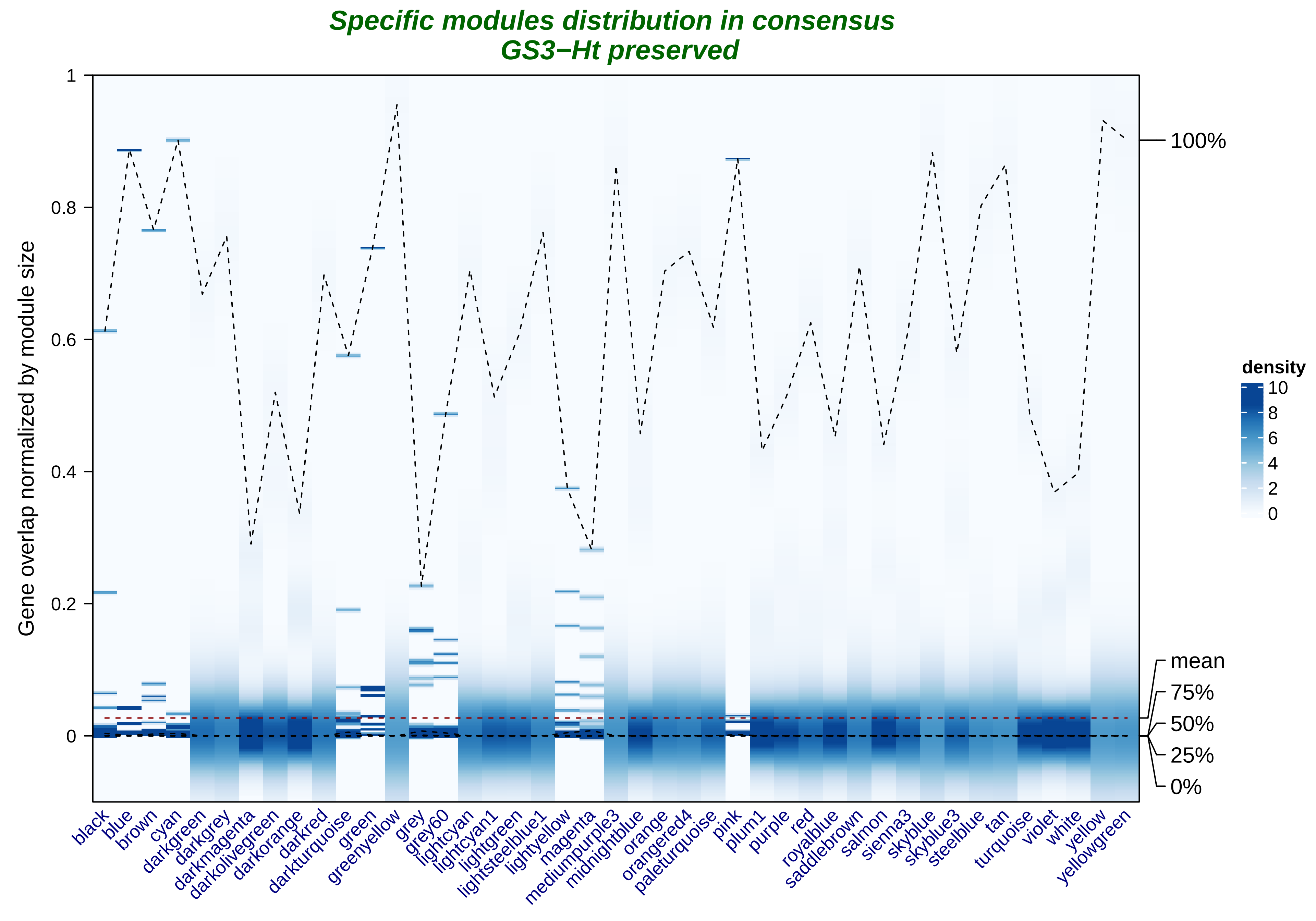

Specific modules distribution in consensus
GS3−Ht preserved
Gene overlap normalized by module size
1
0.8
0.6
0.4
0.2
0
100%
mean
75%
50%
25%
0%
density
10
8
6
4
2
0
black
blue
brown
cyan
darkgreen
darkgrey
darkmagenta
darkolivegreen
darkorange
darkred
darkturquoise
green
greenyellow
grey
grey60
lightcyan
lightcyan1
lightgreen
lightsteelblue1
lightyellow
magenta
mediumpurple3
midnightblue
orange
orangered4
paleturquoise
pink
plum1
purple
red
royalblue
saddlebrown
salmon
sienna3
skyblue
skyblue3
steelblue
tan
turquoise
violet
white
yellow
yellowgreen

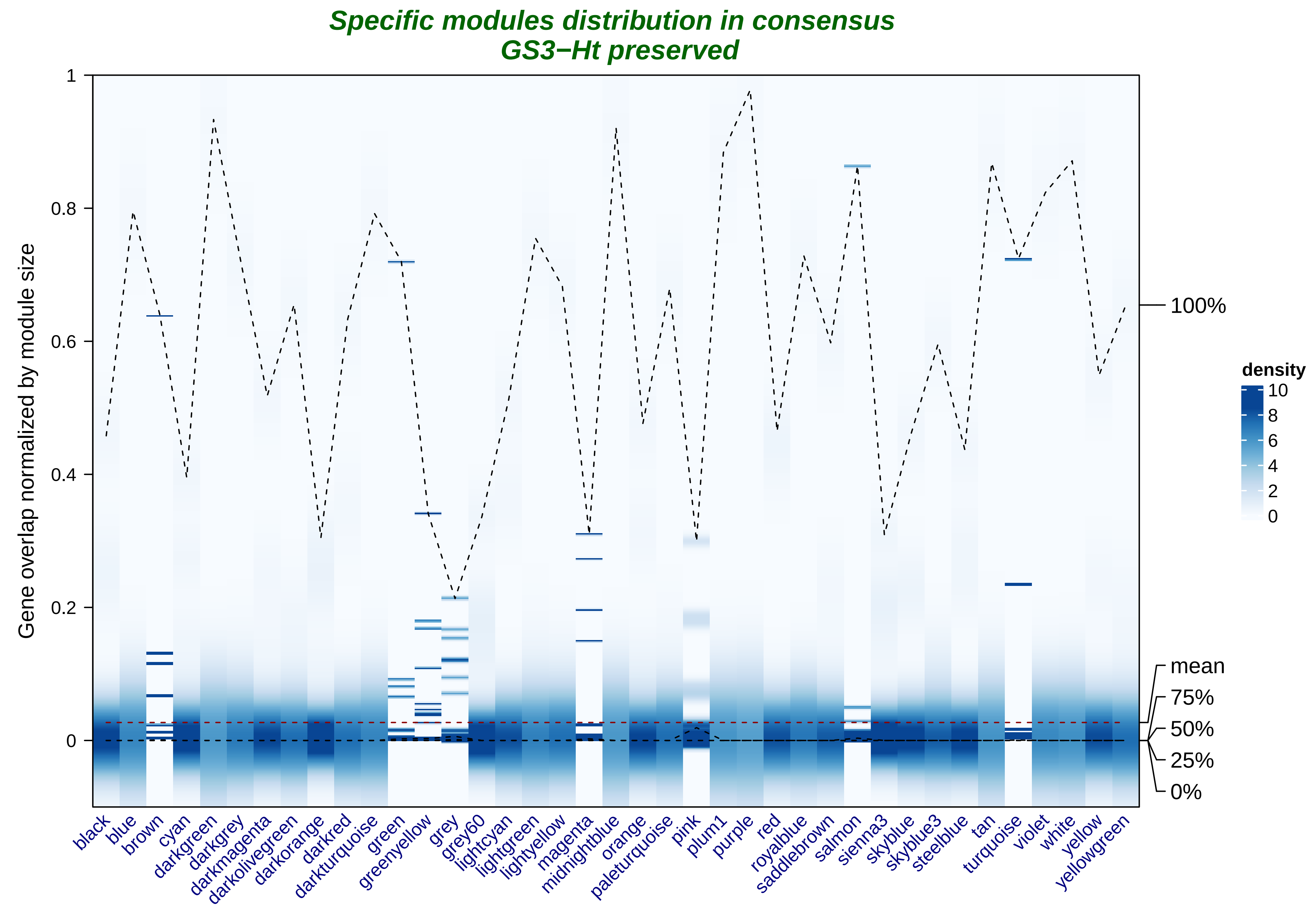

Specific modules distribution in consensus
GS3−Ht preserved
Gene overlap normalized by module size
1
0.8
0.6
0.4
0.2
0
100%
mean
75%
50%
25%
0%
density
10
8
6
4
2
0
black
blue
brown
cyan
darkgreen
darkgrey
darkmagenta
darkolivegreen
darkorange
darkred
darkturquoise
green
greenyellow
grey
grey60
lightcyan
lightgreen
lightyellow
magenta
midnightblue
orange
paleturquoise
pink
plum1
purple
red
royalblue
saddlebrown
salmon
sienna3
skyblue
skyblue3
steelblue
tan
turquoise
violet
white
yellow
yellowgreen

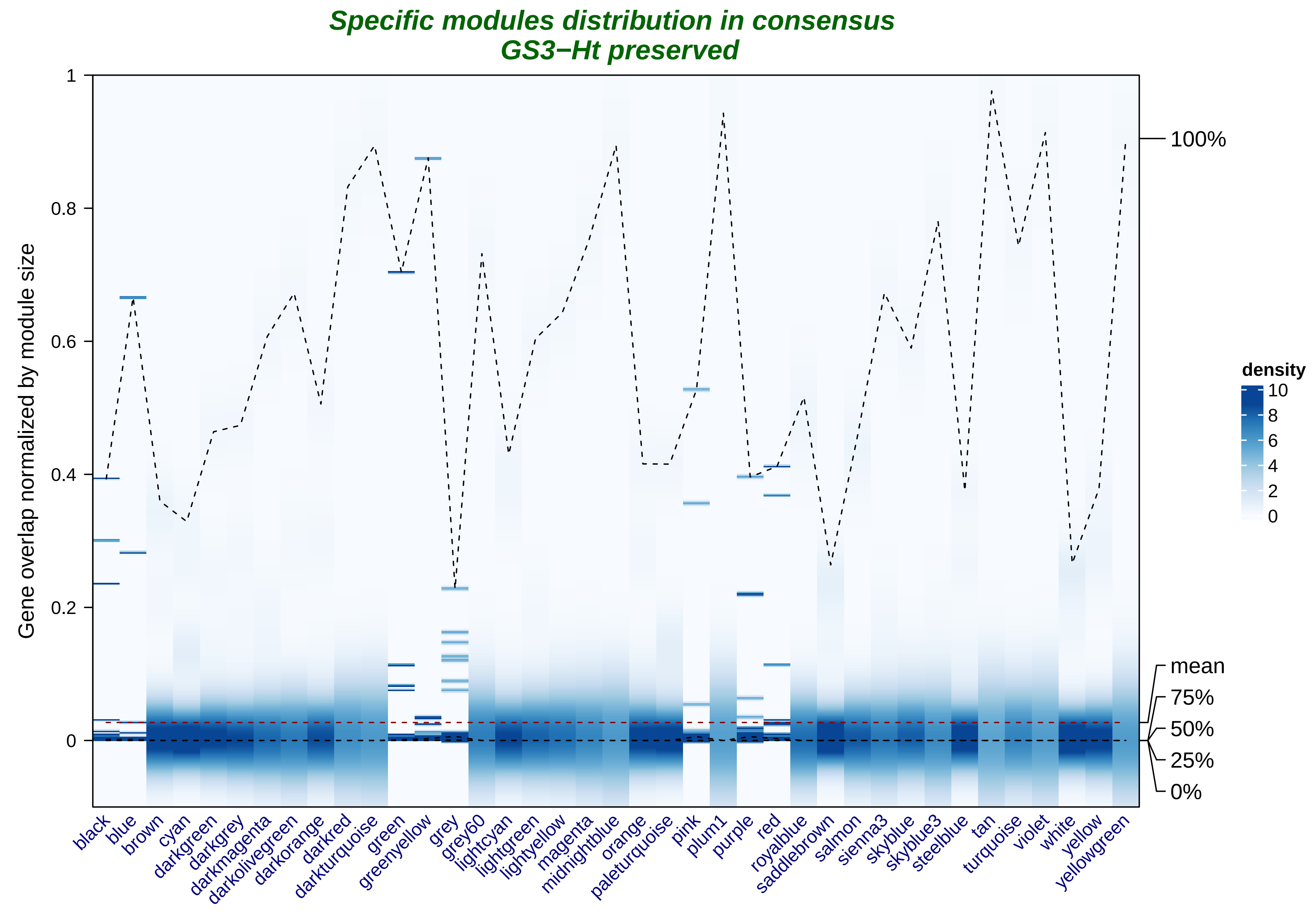
Specific modules distribution in consensus
GS3−Ht preserved
Gene overlap normalized by module size
1
0.8
0.6
0.4
0.2
0
100%
mean
75%
50%
25%
0%
density
10
8
6
4
2
0
black
blue
brown
cyan
darkgreen
darkgrey
darkmagenta
darkolivegreen
darkorange
darkred
darkturquoise
green
greenyellow
grey
grey60
lightcyan
lightgreen
lightyellow
magenta
midnightblue
orange
paleturquoise
pink
plum1
purple
red
royalblue
saddlebrown
salmon
sienna3
skyblue
skyblue3
steelblue
tan
turquoise
violet
white
yellow
yellowgreen

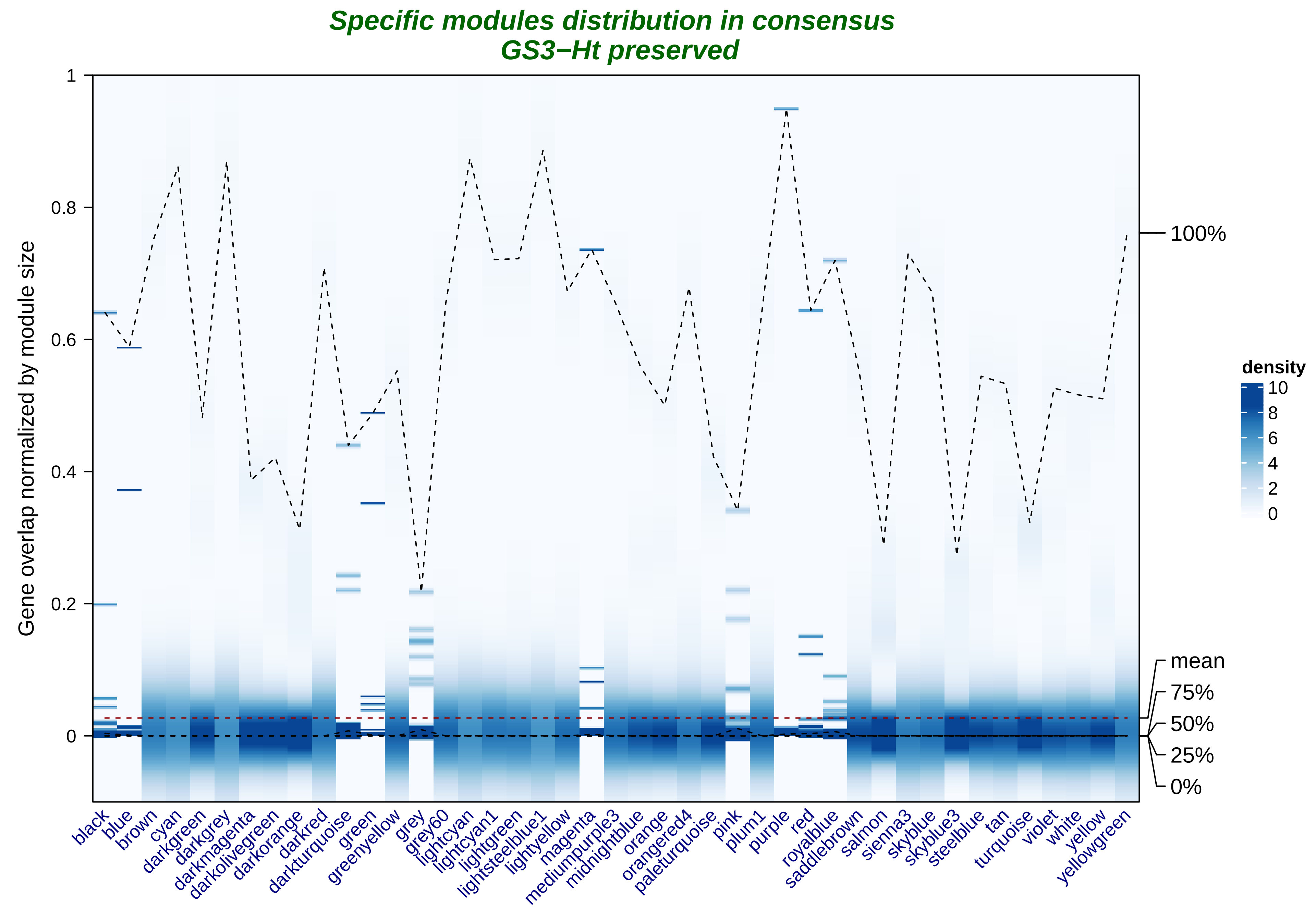

Specific modules distribution in consensus
GS3−Ht preserved
Gene overlap normalized by module size
1
0.8
0.6
0.4
0.2
0
100%
mean
75%
50%
25%
0%
density
10
8
6
4
2
0
black
blue
brown
cyan
darkgreen
darkgrey
darkmagenta
darkolivegreen
darkorange
darkred
darkturquoise
green
greenyellow
grey
grey60
lightcyan
lightcyan1
lightgreen
lightsteelblue1
lightyellow
magenta
mediumpurple3
midnightblue
orange
orangered4
paleturquoise
pink
plum1
purple
red
royalblue
saddlebrown
salmon
sienna3
skyblue
skyblue3
steelblue
tan
turquoise
violet
white
yellow
yellowgreen

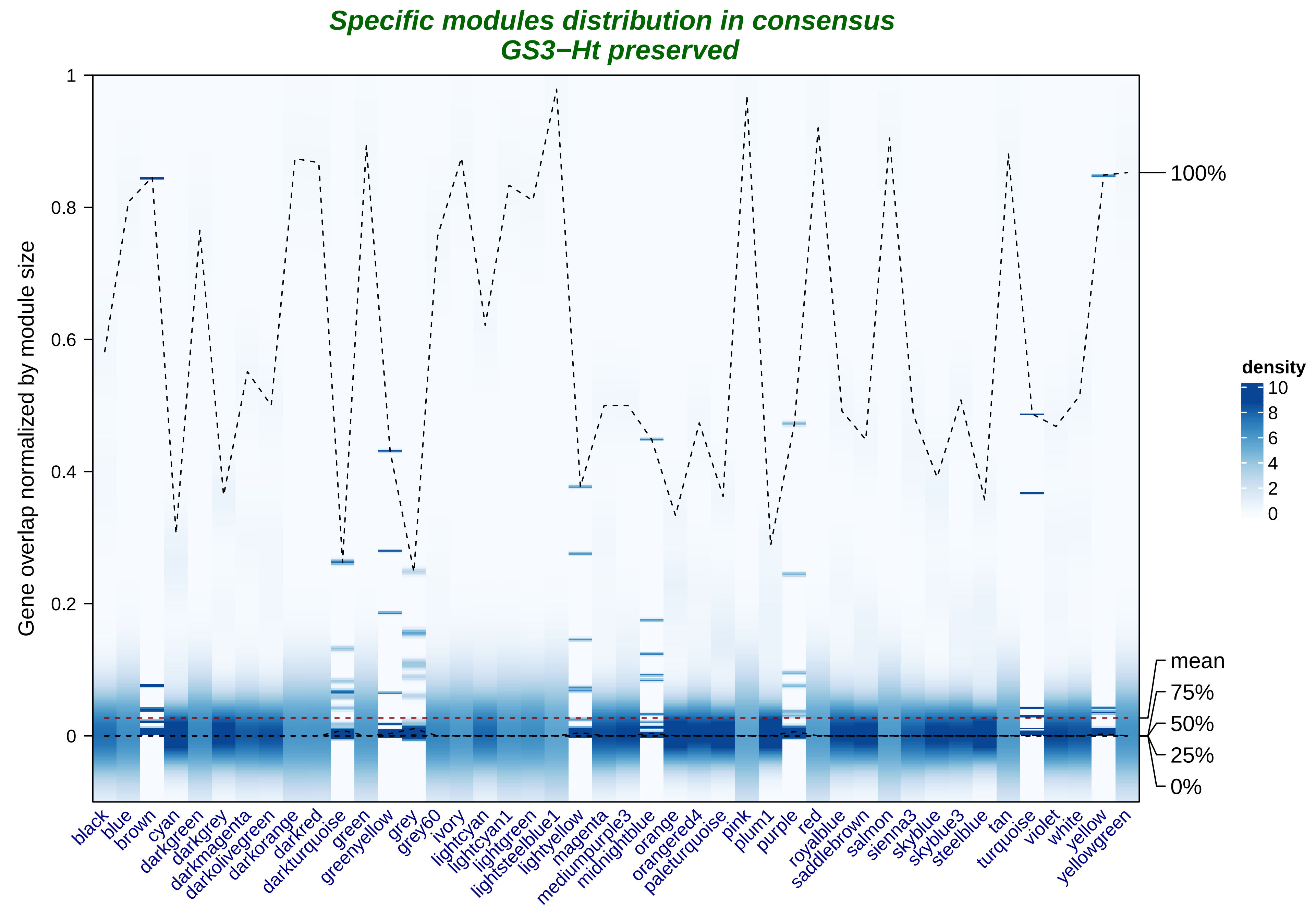
Specific modules distribution in consensus
GS3−Ht preserved
Gene overlap normalized by module size
1
0.8
0.6
0.4
0.2
0
100%
mean
75%
50%
25%
0%
density
10
8
6
4
2
0
black
blue
brown
cyan
darkgreen
darkgrey
darkmagenta
darkolivegreen
darkorange
darkred
darkturquoise
green
greenyellow
grey
grey60
ivory
lightcyan
lightcyan1
lightgreen
lightsteelblue1
lightyellow
magenta
mediumpurple3
midnightblue
orange
orangered4
paleturquoise
pink
plum1
purple
red
royalblue
saddlebrown
salmon
sienna3
skyblue
skyblue3
steelblue
tan
turquoise
violet
white
yellow
yellowgreen

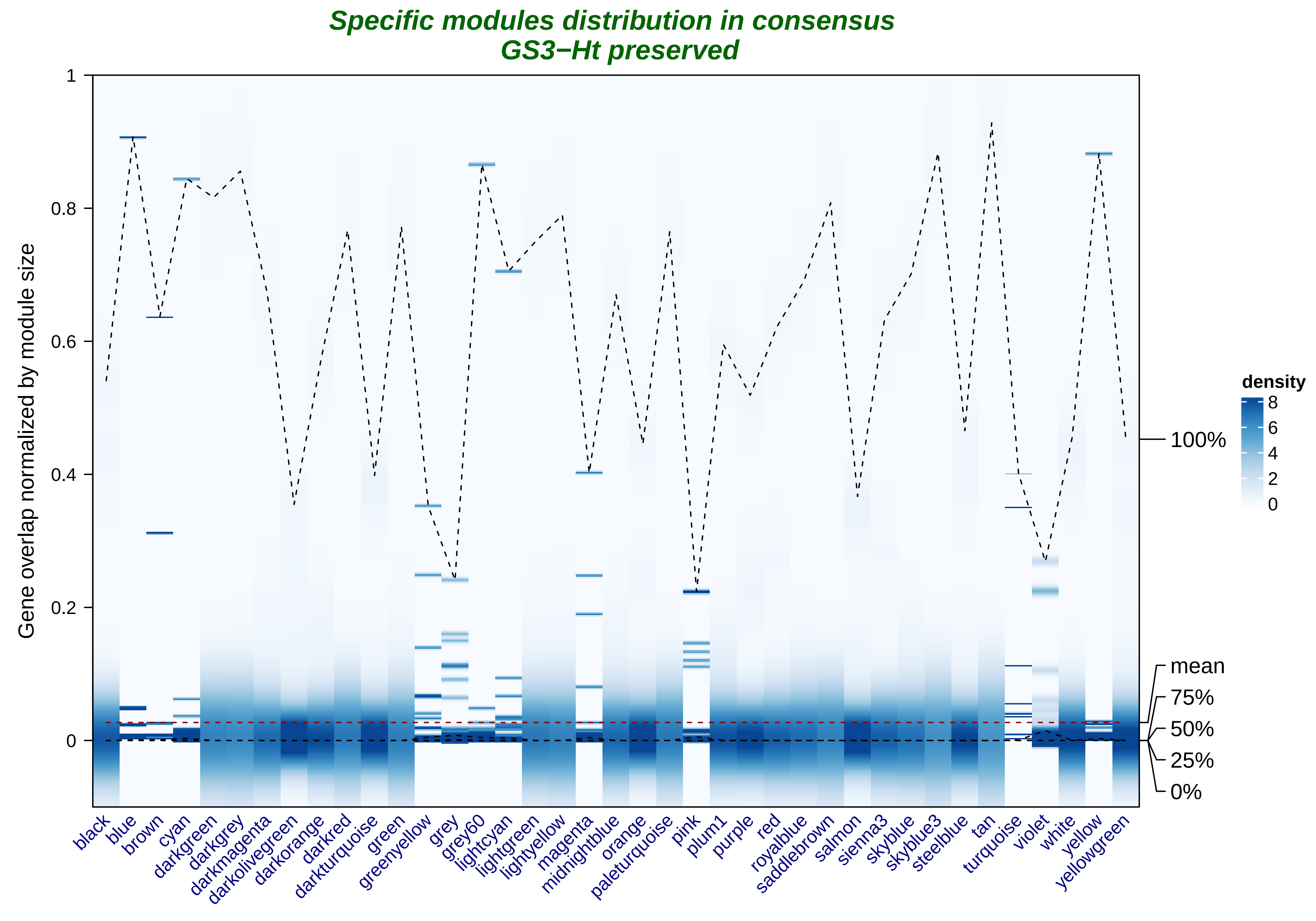
Specific modules distribution in consensus
GS3−Ht preserved
Gene overlap normalized by module size
1
0.8
0.6
0.4
0.2
0
density
8
6
4
2
0
100%
mean
75%
50%
25%
0%
black
blue
brown
cyan
darkgreen
darkgrey
darkmagenta
darkolivegreen
darkorange
darkred
darkturquoise
green
greenyellow
grey
grey60
lightcyan
lightgreen
lightyellow
magenta
midnightblue
orange
paleturquoise
pink
plum1
purple
red
royalblue
saddlebrown
salmon
sienna3
skyblue
skyblue3
steelblue
tan
turquoise
violet
white
yellow
yellowgreen

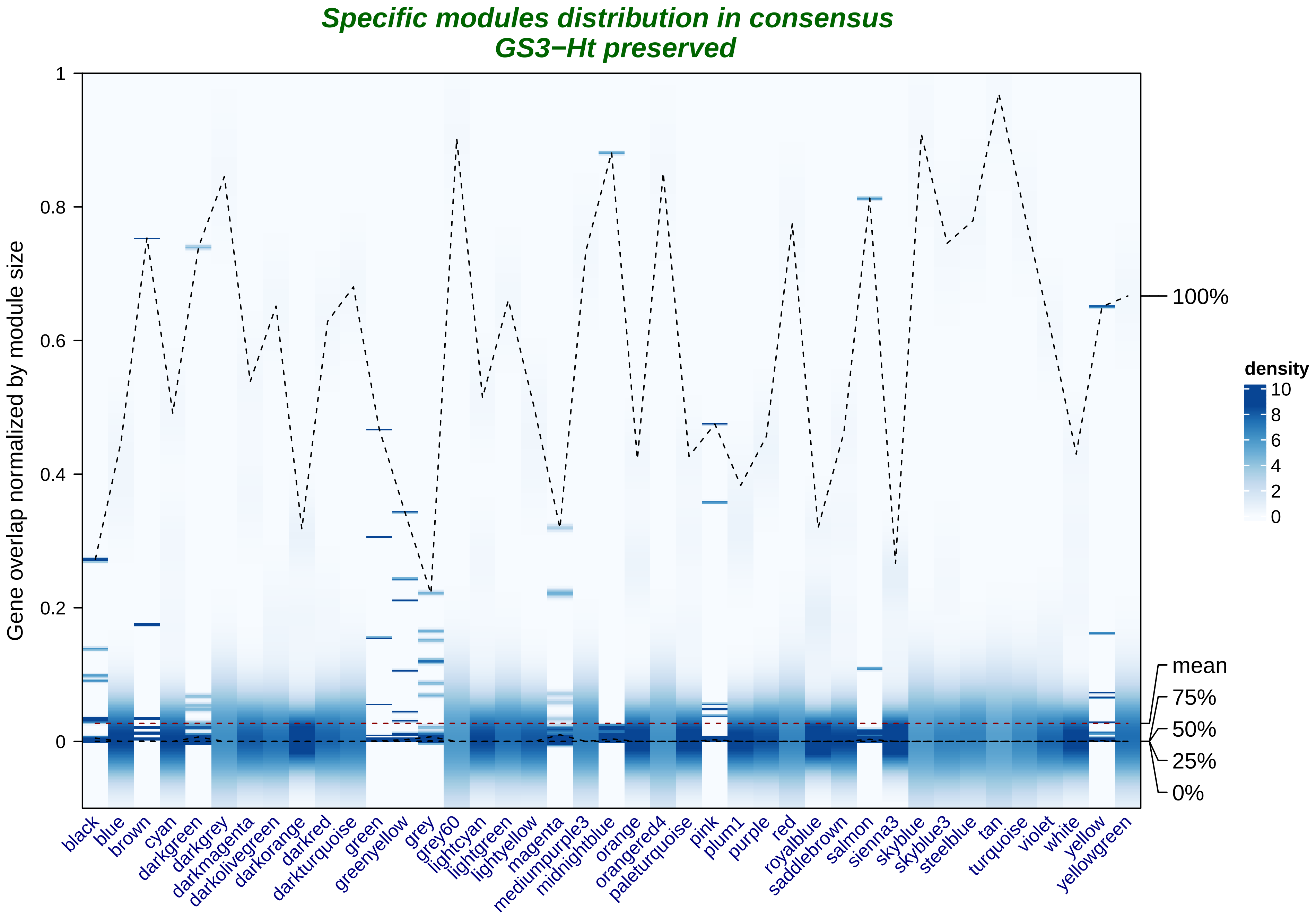
Specific modules distribution in consensus
GS3−Ht preserved
Gene overlap normalized by module size
1
0.8
0.6
0.4
0.2
0
100%
mean
75%
50%
25%
0%
density
10
8
6
4
2
0
black
blue
brown
cyan
darkgreen
darkgrey
darkmagenta
darkolivegreen
darkorange
darkred
darkturquoise
green
greenyellow
grey
grey60
lightcyan
lightgreen
lightyellow
magenta
mediumpurple3
midnightblue
orange
orangered4
paleturquoise
pink
plum1
purple
red
royalblue
saddlebrown
salmon
sienna3
skyblue
skyblue3
steelblue
tan
turquoise
violet
white
yellow
yellowgreen

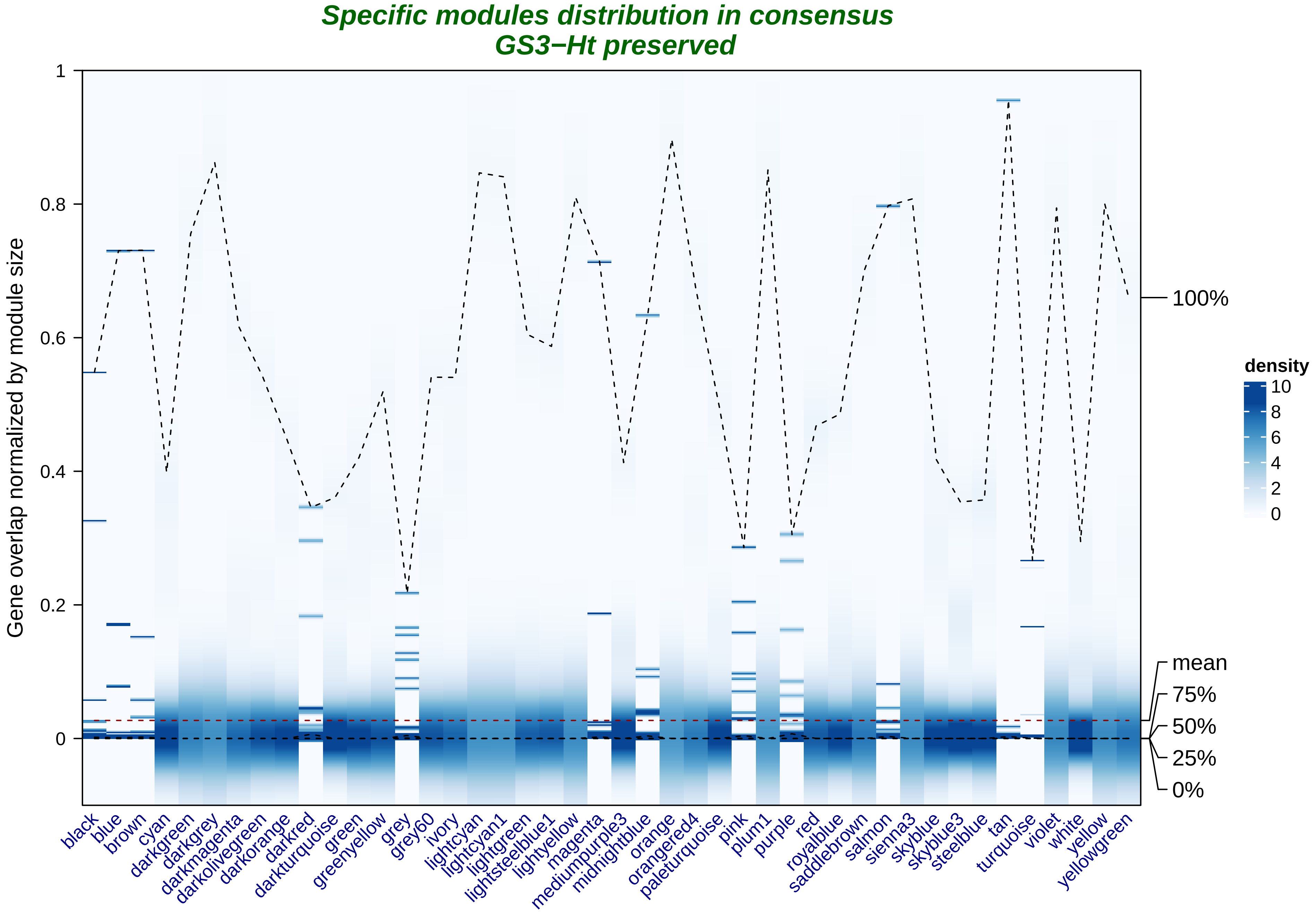
Specific modules distribution in consensus
GS3−Ht preserved
Gene overlap normalized by module size
1
0.8
0.6
0.4
0.2
0
100%
mean
75%
50%
25%
0%
density
10
8
6
4
2
0
black
blue
brown
cyan
darkgreen
darkgrey
darkmagenta
darkolivegreen
darkorange
darkred
darkturquoise
green
greenyellow
grey
grey60
ivory
lightcyan
lightcyan1
lightgreen
lightsteelblue1
lightyellow
magenta
mediumpurple3
midnightblue
orange
orangered4
paleturquoise
pink
plum1
purple
red
royalblue
saddlebrown
salmon
sienna3
skyblue
skyblue3
steelblue
tan
turquoise
violet
white
yellow
yellowgreen

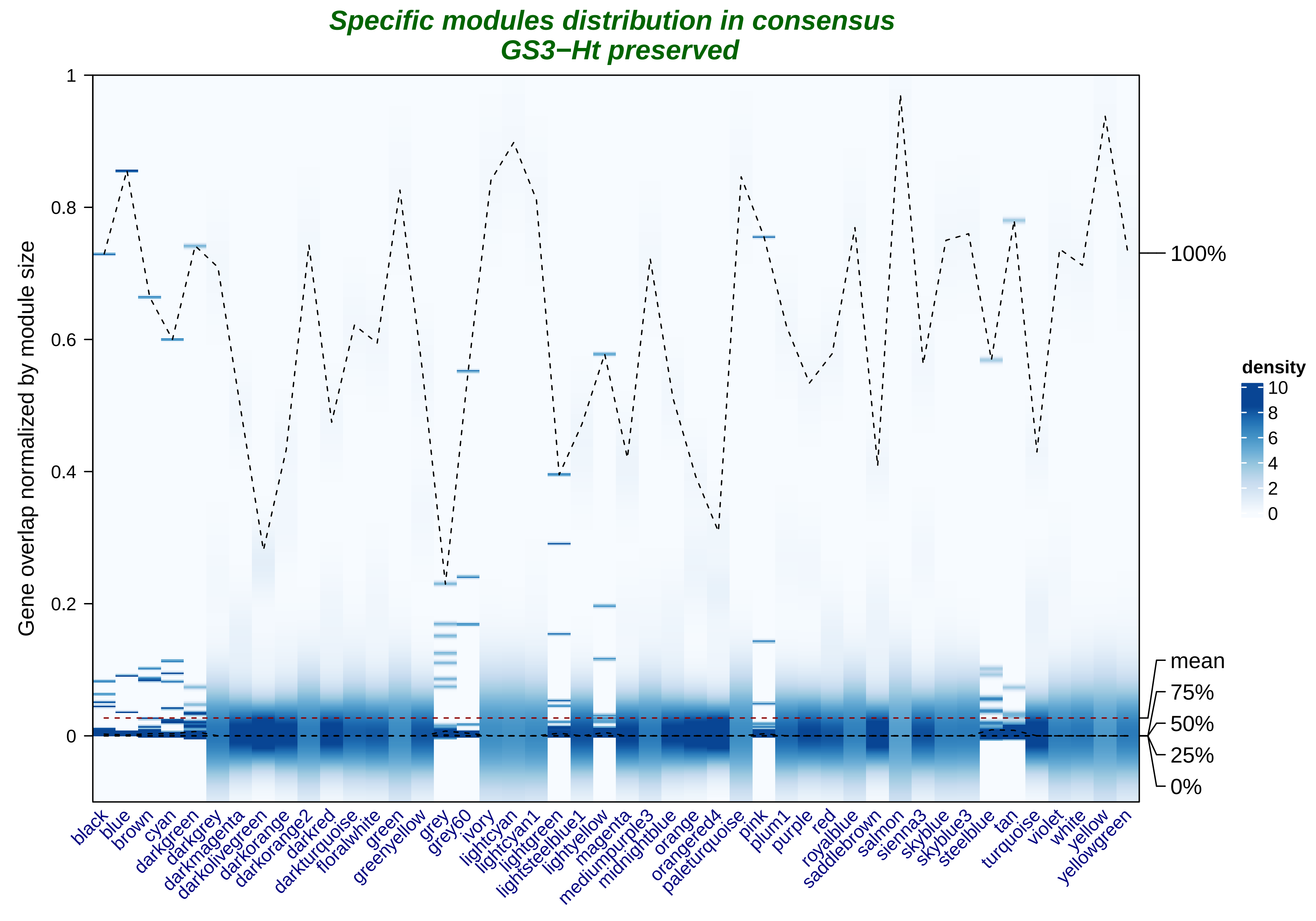
Specific modules distribution in consensus
GS3−Ht preserved
Gene overlap normalized by module size
1
0.8
0.6
0.4
0.2
0
100%
mean
75%
50%
25%
0%
density
10
8
6
4
2
0
black
blue
brown
cyan
darkgreen
darkgrey
darkmagenta
darkolivegreen
darkorange
darkorange2
darkred
darkturquoise
floralwhite
green
greenyellow
grey
grey60
ivory
lightcyan
lightcyan1
lightgreen
lightsteelblue1
lightyellow
magenta
mediumpurple3
midnightblue
orange
orangered4
paleturquoise
pink
plum1
purple
red
royalblue
saddlebrown
salmon
sienna3
skyblue
skyblue3
steelblue
tan
turquoise
violet
white
yellow
yellowgreen

Specific modules distribution in consensus GS3−Ht preserved

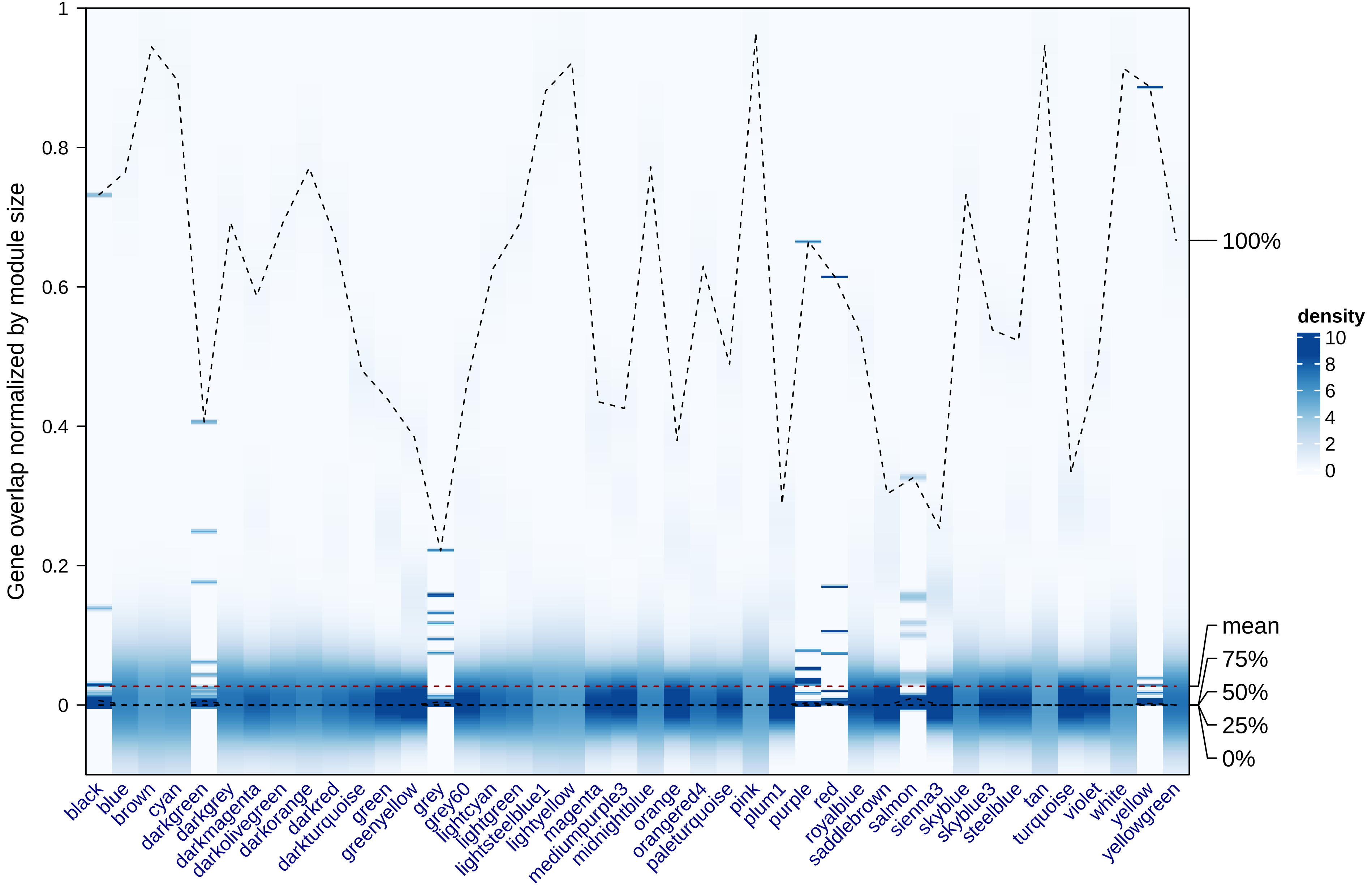

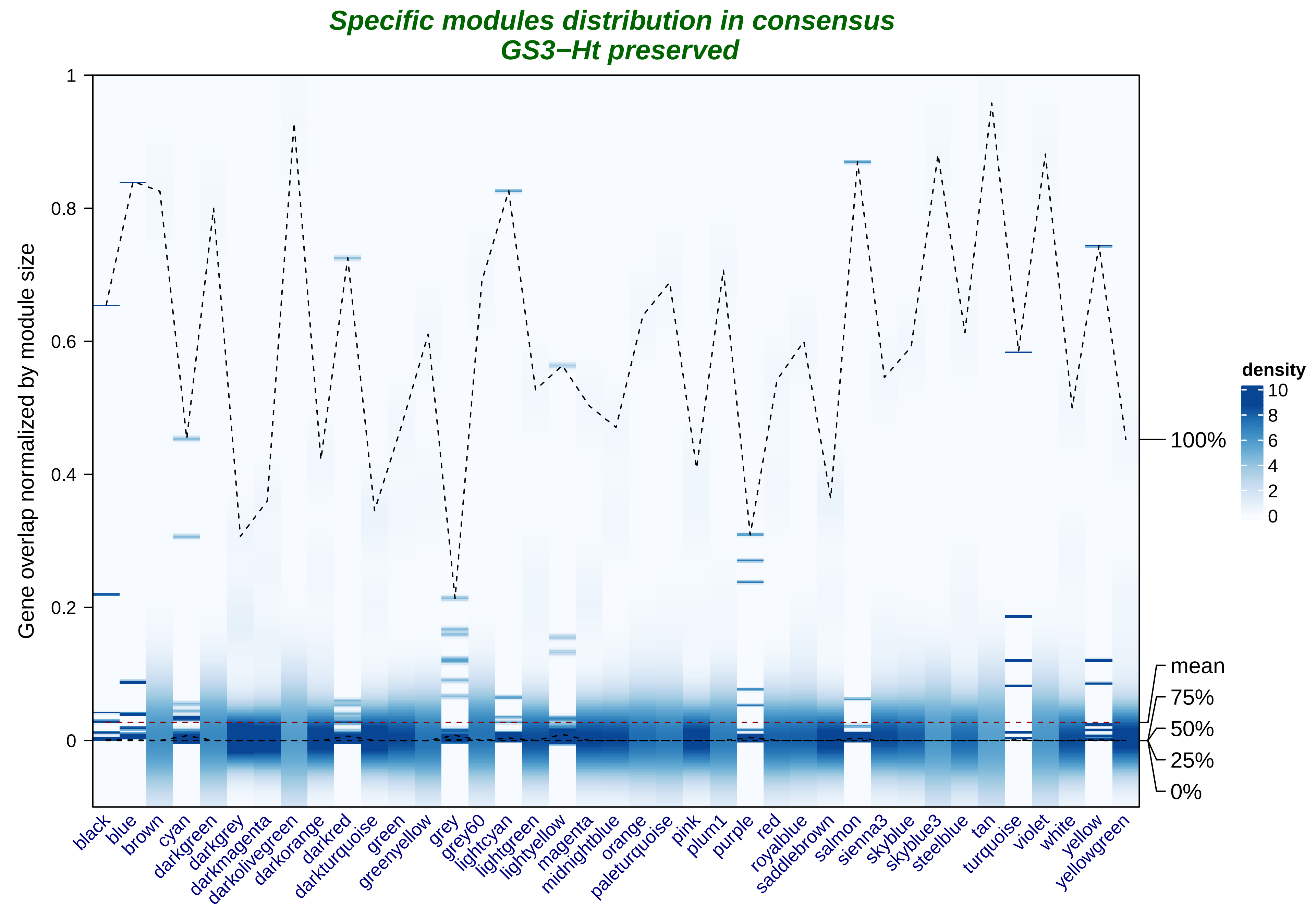
Specific modules distribution in consensus
GS3−Ht preserved
Gene overlap normalized by module size
1
0.8
0.6
0.4
0.2
0
density
10
8
6
4
2
0
100%
mean
75%
50%
25%
0%
black
blue
brown
cyan
darkgreen
darkgrey
darkmagenta
darkolivegreen
darkorange
darkred
darkturquoise
green
greenyellow
grey
grey60
lightcyan
lightgreen
lightyellow
magenta
midnightblue
orange
paleturquoise
pink
plum1
purple
red
royalblue
saddlebrown
salmon
sienna3
skyblue
skyblue3
steelblue
tan
turquoise
violet
white
yellow
yellowgreen

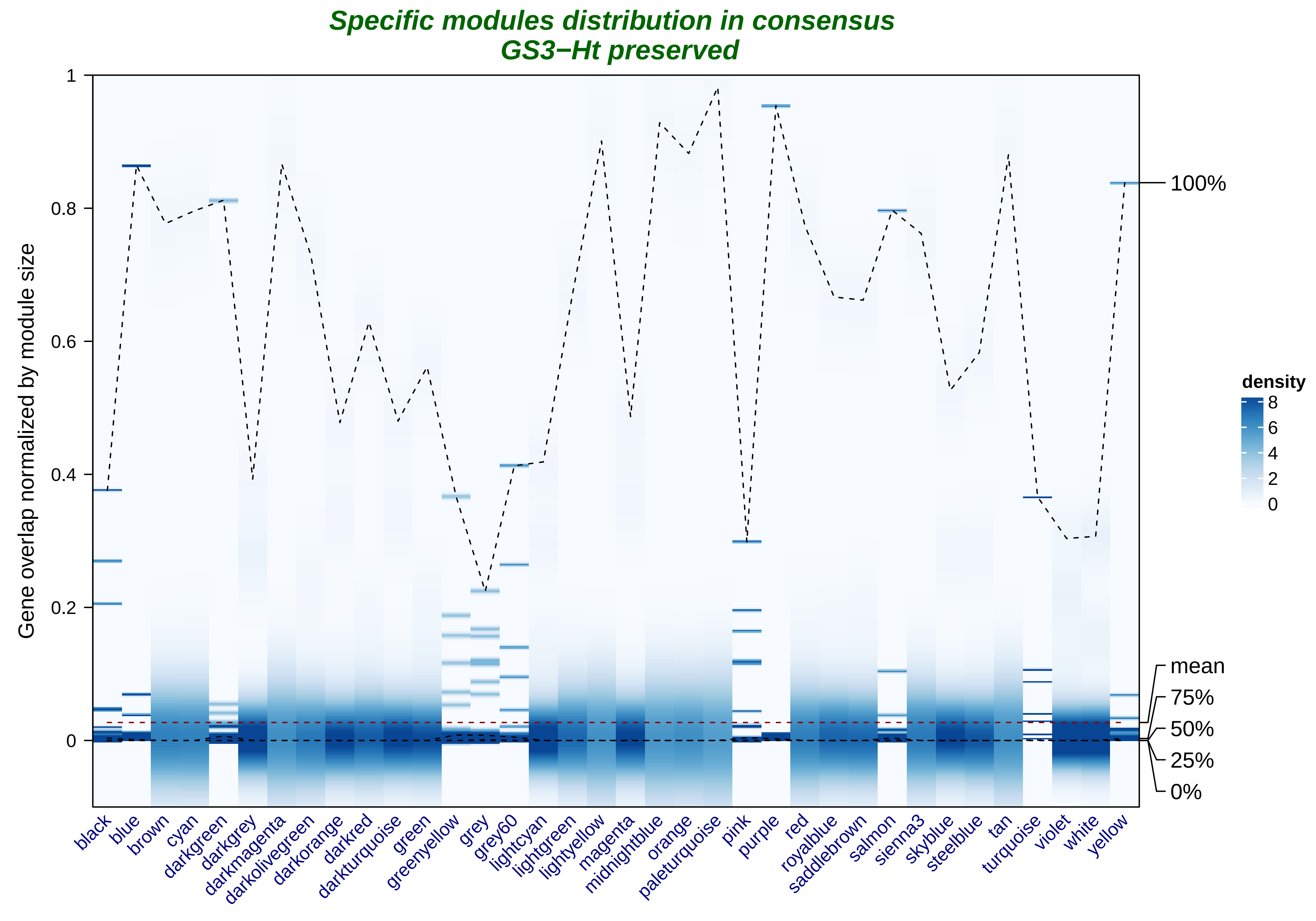

Specific modules distribution in consensus
GS3−Ht preserved
Gene overlap normalized by module size
1
0.8
0.6
0.4
0.2
0
100%
mean
75%
50%
25%
0%
density
8
6
4
2
0
black
blue
brown
cyan
darkgreen
darkgrey
darkmagenta
darkolivegreen
darkorange
darkred
darkturquoise
green
greenyellow
grey
grey60
lightcyan
lightgreen
lightyellow
magenta
midnightblue
orange
paleturquoise
pink
purple
red
royalblue
saddlebrown
salmon
sienna3
skyblue
steelblue
tan
turquoise
violet
white
yellow

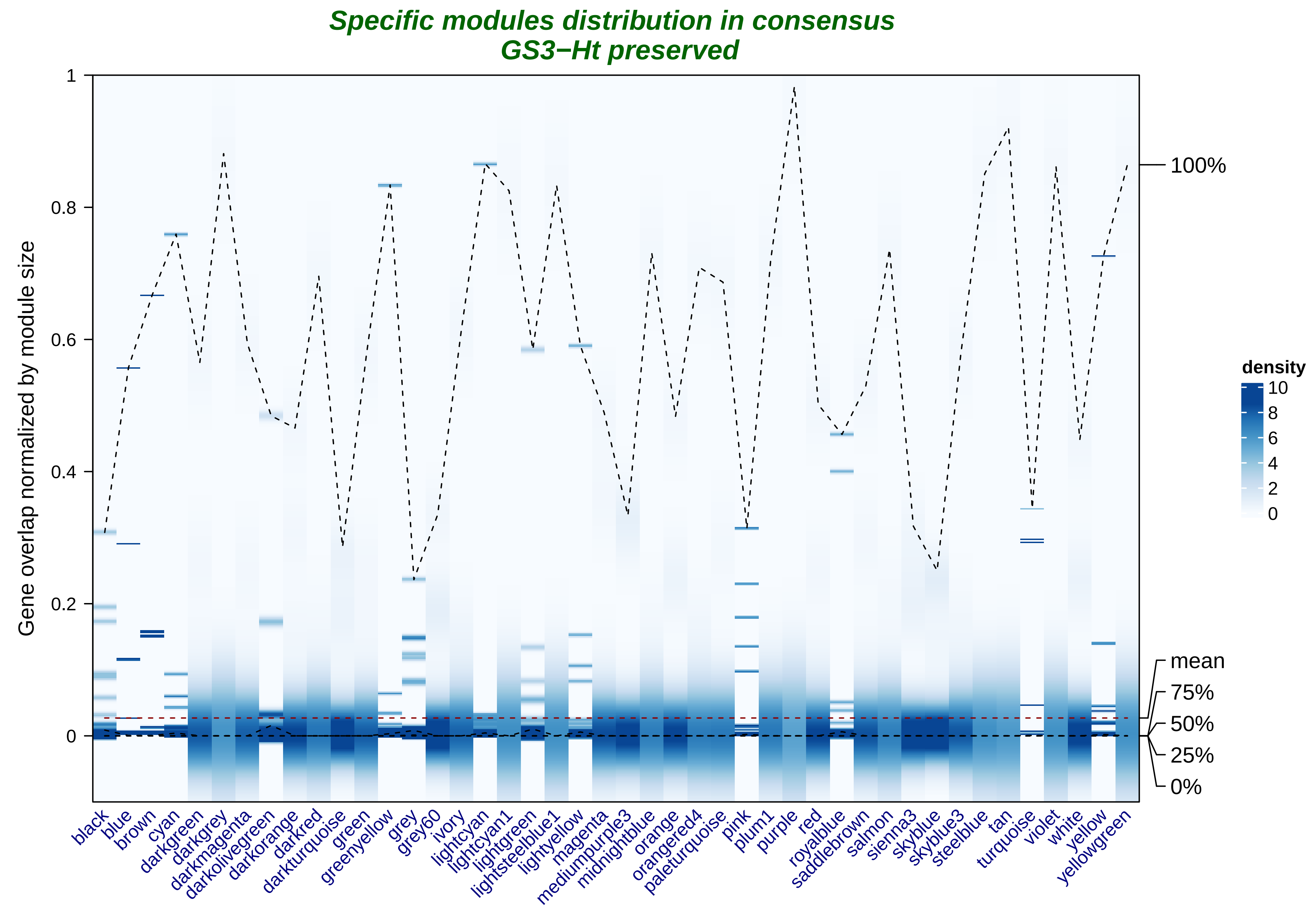

Specific modules distribution in consensus
GS3−Ht preserved
Gene overlap normalized by module size
1
0.8
0.6
0.4
0.2
0
100%
mean
75%
50%
25%
0%
density
10
8
6
4
2
0
black
blue
brown
cyan
darkgreen
darkgrey
darkmagenta
darkolivegreen
darkorange
darkred
darkturquoise
green
greenyellow
grey
grey60
ivory
lightcyan
lightcyan1
lightgreen
lightsteelblue1
lightyellow
magenta
mediumpurple3
midnightblue
orange
orangered4
paleturquoise
pink
plum1
purple
red
royalblue
saddlebrown
salmon
sienna3
skyblue
skyblue3
steelblue
tan
turquoise
violet
white
yellow
yellowgreen

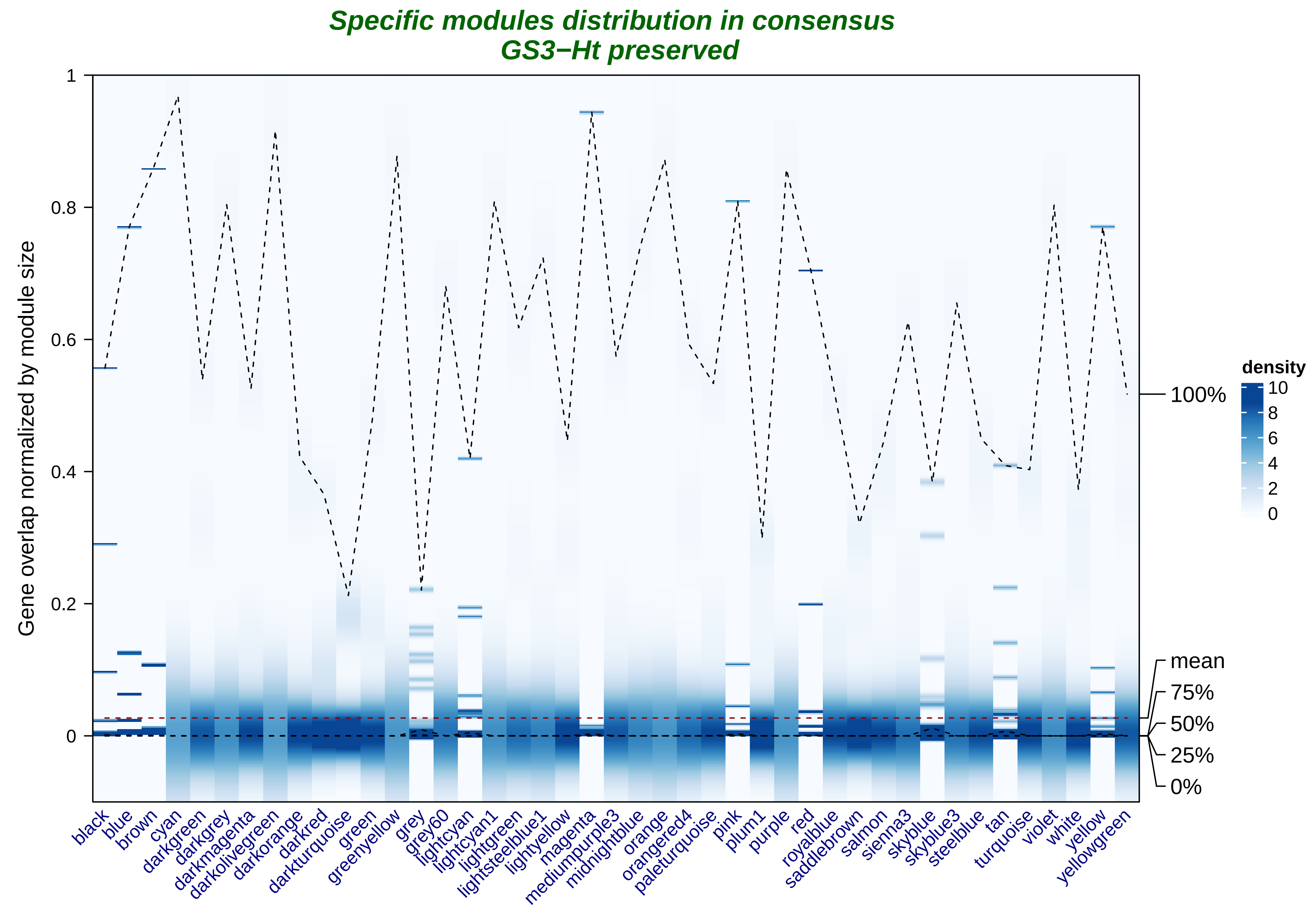

Specific modules distribution in consensus
GS3−Ht preserved
Gene overlap normalized by module size
1
0.8
0.6
0.4
0.2
0
density
10
8
6
4
2
0
100%
mean
75%
50%
25%
0%
black
blue
brown
cyan
darkgreen
darkgrey
darkmagenta
darkolivegreen
darkorange
darkred
darkturquoise
green
greenyellow
grey
grey60
lightcyan
lightcyan1
lightgreen
lightsteelblue1
lightyellow
magenta
mediumpurple3
midnightblue
orange
orangered4
paleturquoise
pink
plum1
purple
red
royalblue
saddlebrown
salmon
sienna3
skyblue
skyblue3
steelblue
tan
turquoise
violet
white
yellow
yellowgreen

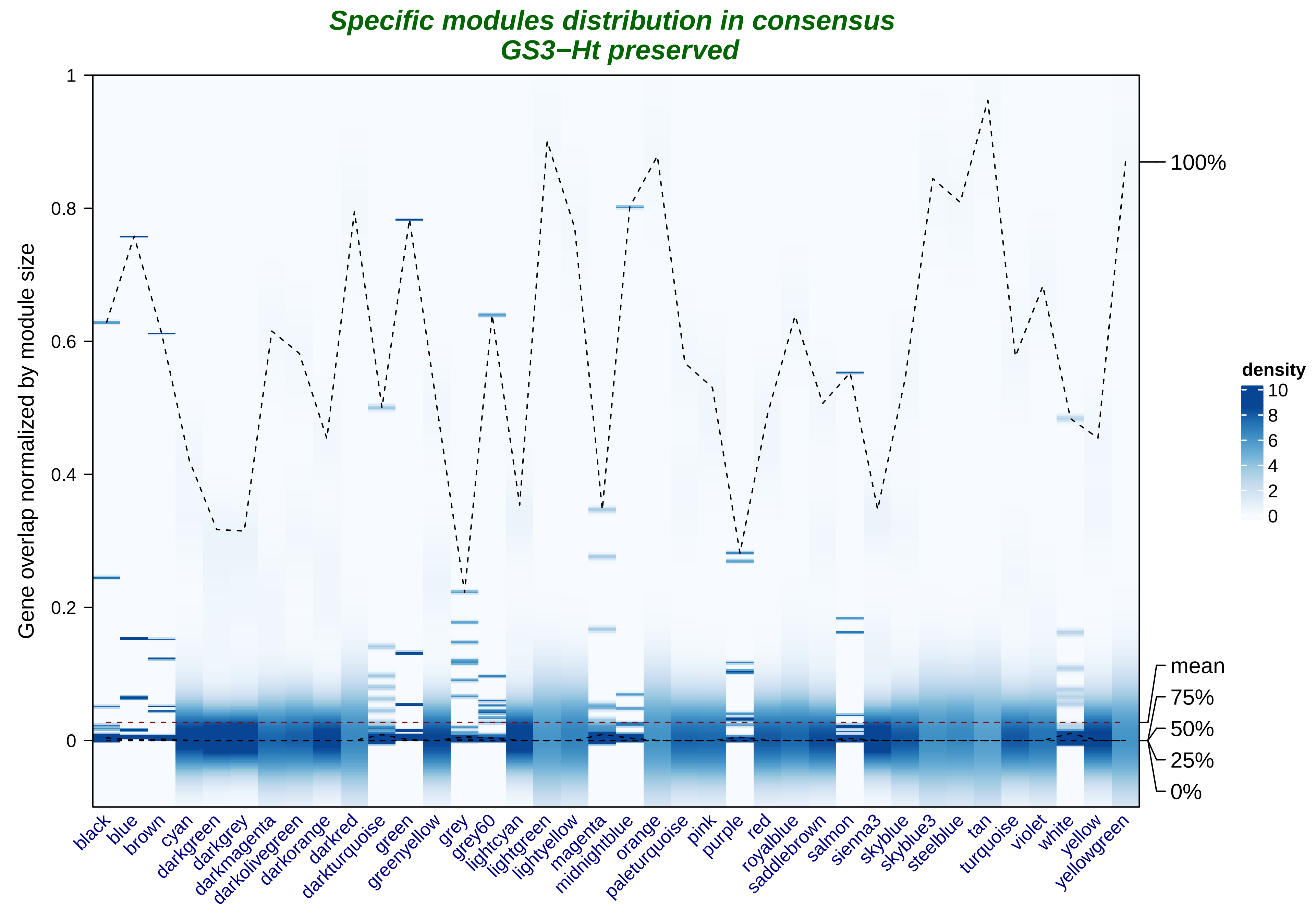
Specific modules distribution in consensus
GS3−Ht preserved
Gene overlap normalized by module size
1
0.8
0.6
0.4
0.2
0
100%
mean
75%
50%
25%
0%
density
10
8
6
4
2
0
black
blue
brown
cyan
darkgreen
darkgrey
darkmagenta
darkolivegreen
darkorange
darkred
darkturquoise
green
greenyellow
grey
grey60
lightcyan
lightgreen
lightyellow
magenta
midnightblue
orange
paleturquoise
pink
purple
red
royalblue
saddlebrown
salmon
sienna3
skyblue
skyblue3
steelblue
tan
turquoise
violet
white
yellow
yellowgreen

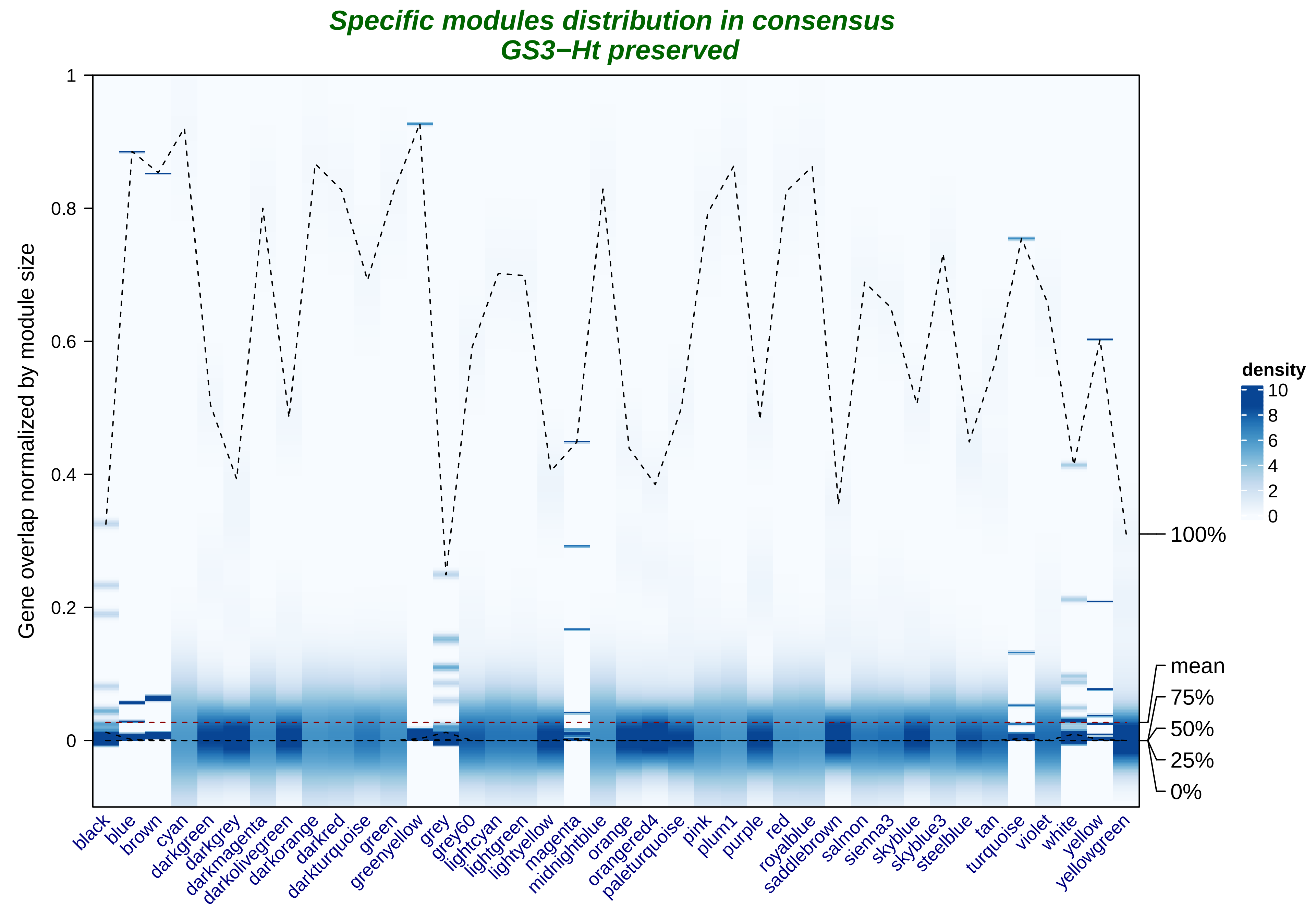
Specific modules distribution in consensus
GS3−Ht preserved
Gene overlap normalized by module size
1
0.8
0.6
0.4
0.2
0
density
10
8
6
4
2
0
100%
mean
75%
50%
25%
0%
black
blue
brown
cyan
darkgreen
darkgrey
darkmagenta
darkolivegreen
darkorange
darkred
darkturquoise
green
greenyellow
grey
grey60
lightcyan
lightgreen
lightyellow
magenta
midnightblue
orange
orangered4
paleturquoise
pink
plum1
purple
red
royalblue
saddlebrown
salmon
sienna3
skyblue
skyblue3
steelblue
tan
turquoise
violet
white
yellow
yellowgreen

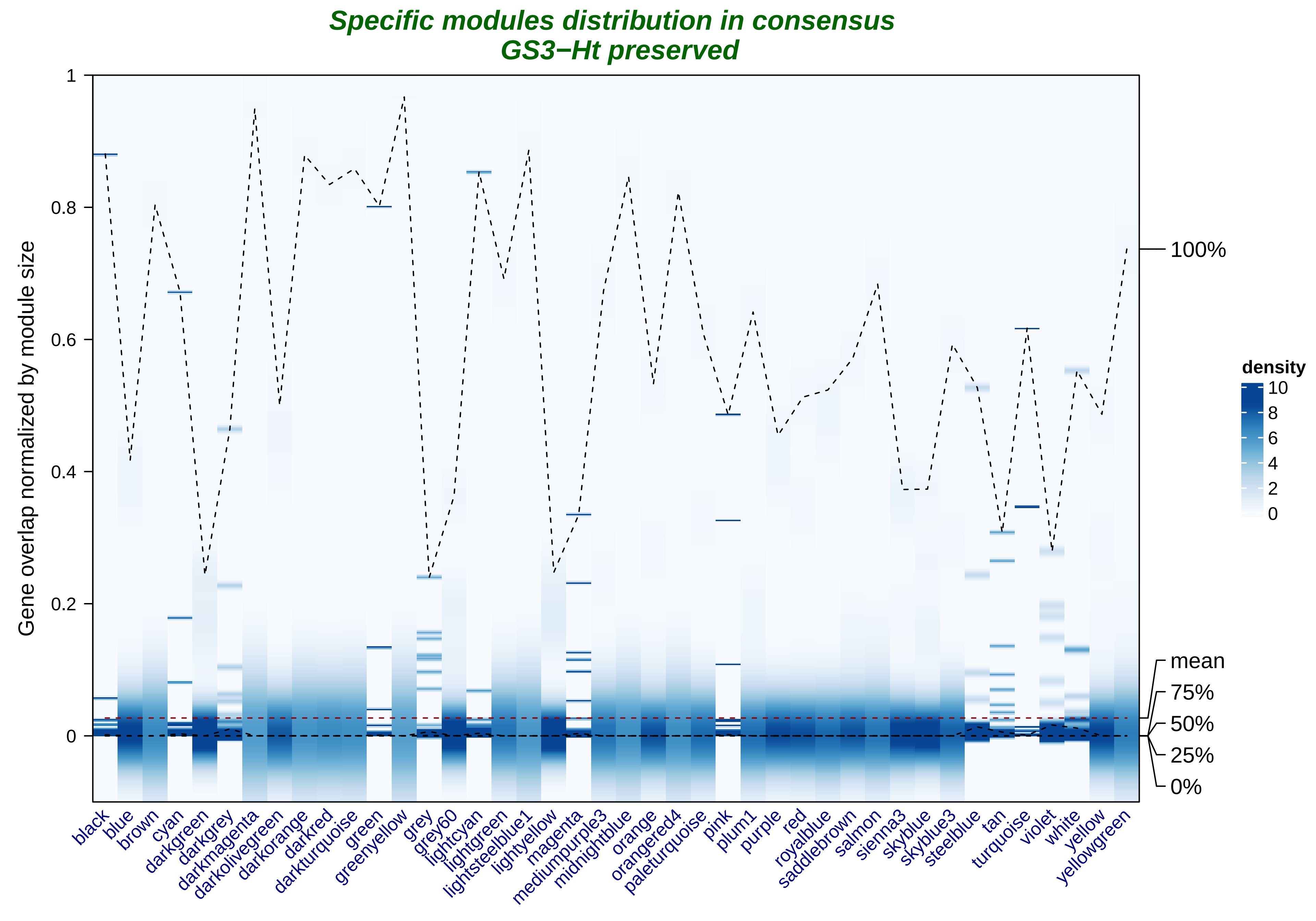

Specific modules distribution in consensus
GS3−Ht preserved
Gene overlap normalized by module size
1
0.8
0.6
0.4
0.2
0
100%
mean
75%
50%
25%
0%
density
10
8
6
4
2
0
black
blue
brown
cyan
darkgreen
darkgrey
darkmagenta
darkolivegreen
darkorange
darkred
darkturquoise
green
greenyellow
grey
grey60
lightcyan
lightgreen
lightsteelblue1
lightyellow
magenta
mediumpurple3
midnightblue
orange
orangered4
paleturquoise
pink
plum1
purple
red
royalblue
saddlebrown
salmon
sienna3
skyblue
skyblue3
steelblue
tan
turquoise
violet
white
yellow
yellowgreen

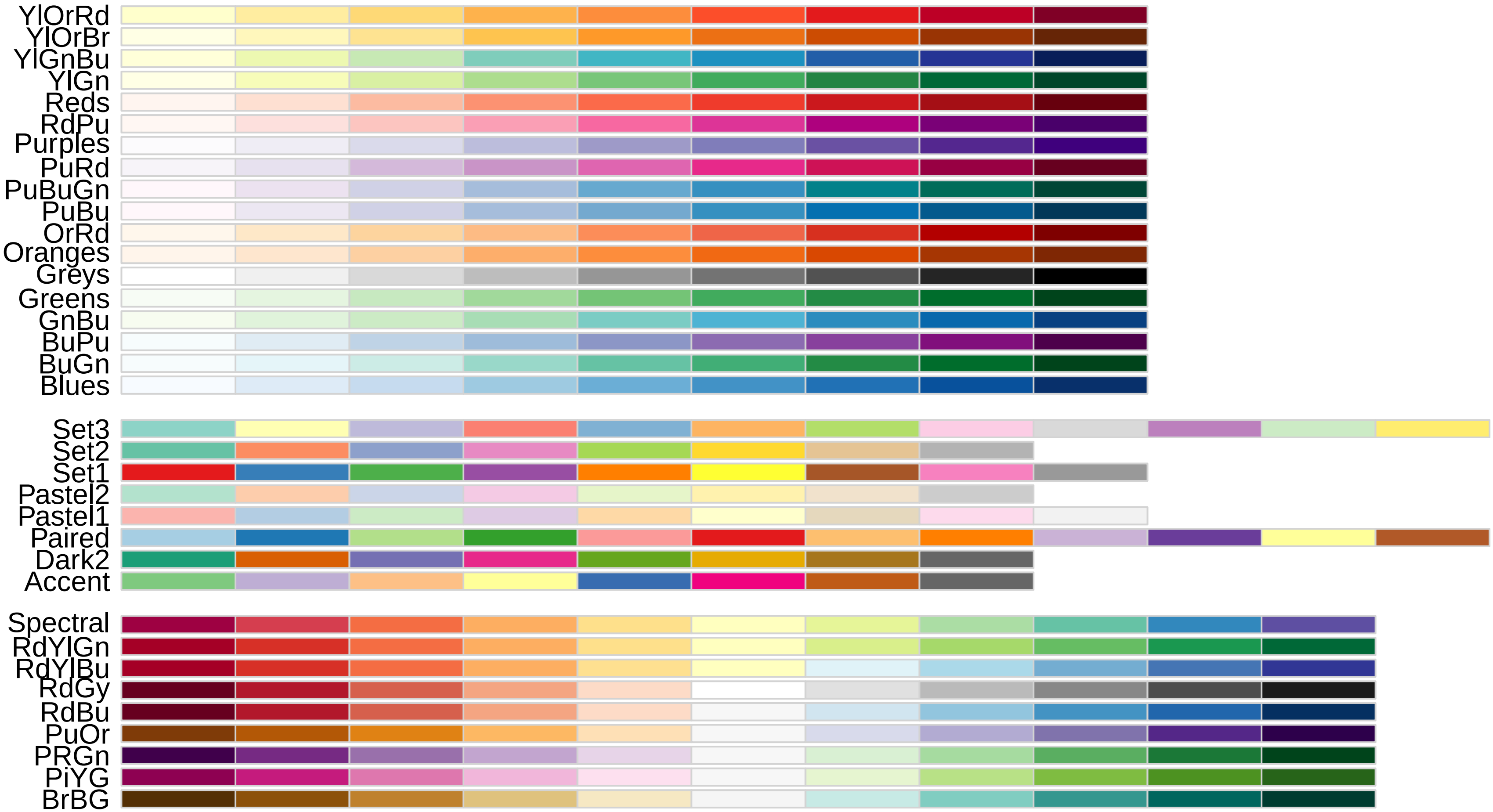
YlOrRd
YlOrBr
YlGnBu
YlGn
Reds
RdPu
Purples
PuRd
PuBuGn
PuBu
OrRd
Oranges
Greys
Greens
GnBu
BuPu
BuGn
Blues
Set3
Set2
Set1
Pastel2
Pastel1
Paired
Dark2
Accent
Spectral
RdYlGn
RdYlBu
RdGy
RdBu
PuOr
PRGn
PiYG
BrBG

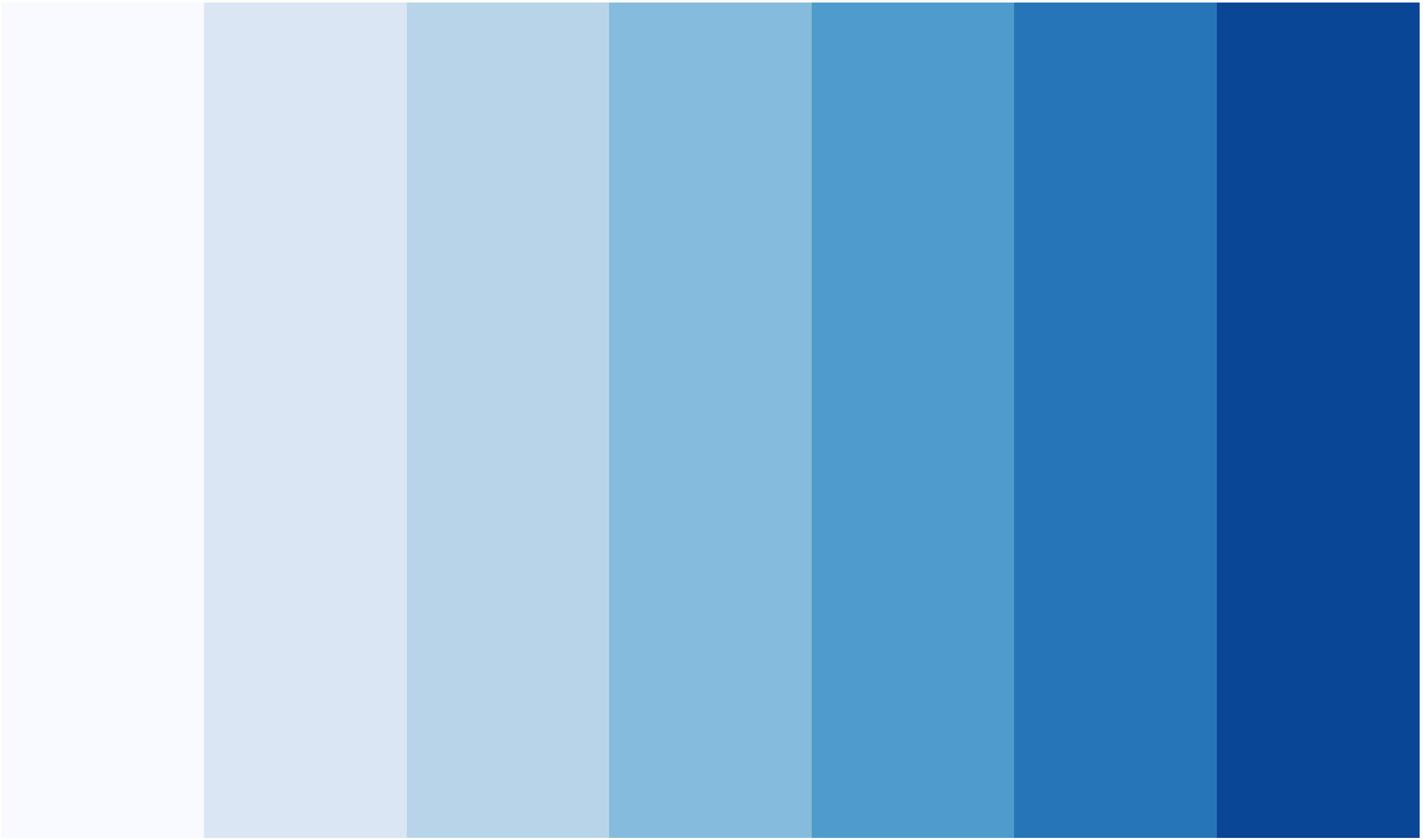

Blues (sequential)

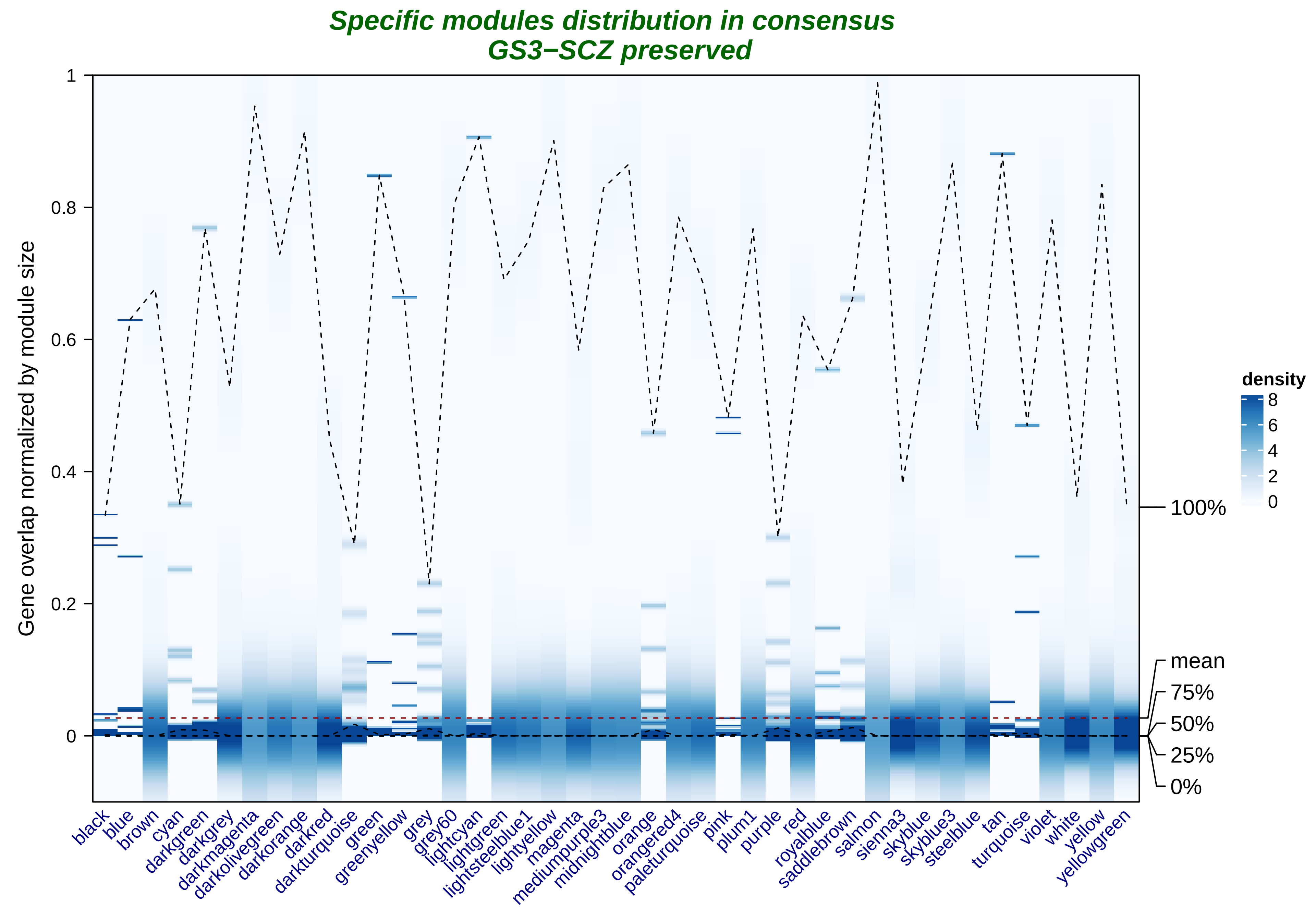

Specific modules distribution in consensus
GS3−SCZ preserved
Gene overlap normalized by module size
1
0.8
0.6
0.4
0.2
0
density
8
6
4
2
0
100%
mean
75%
50%
25%
0%
black
blue
brown
cyan
darkgreen
darkgrey
darkmagenta
darkolivegreen
darkorange
darkred
darkturquoise
green
greenyellow
grey
grey60
lightcyan
lightgreen
lightsteelblue1
lightyellow
magenta
mediumpurple3
midnightblue
orange
orangered4
paleturquoise
pink
plum1
purple
red
royalblue
saddlebrown
salmon
sienna3
skyblue
skyblue3
steelblue
tan
turquoise
violet
white
yellow
yellowgreen

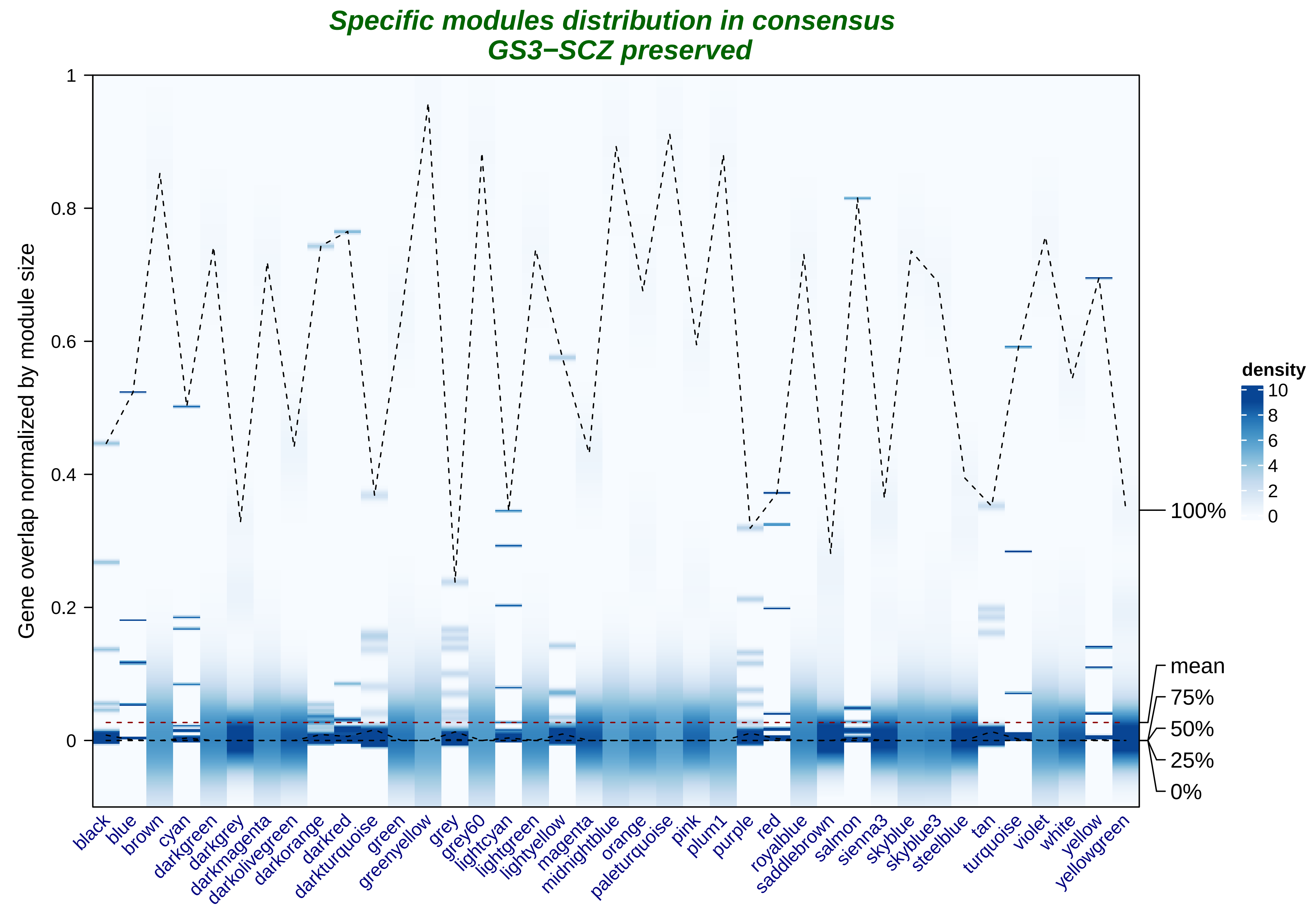

Specific modules distribution in consensus
GS3−SCZ preserved
Gene overlap normalized by module size
1
0.8
0.6
0.4
0.2
0
density
10
8
6
4
2
0
100%
mean
75%
50%
25%
0%
black
blue
brown
cyan
darkgreen
darkgrey
darkmagenta
darkolivegreen
darkorange
darkred
darkturquoise
green
greenyellow
grey
grey60
lightcyan
lightgreen
lightyellow
magenta
midnightblue
orange
paleturquoise
pink
plum1
purple
red
royalblue
saddlebrown
salmon
sienna3
skyblue
skyblue3
steelblue
tan
turquoise
violet
white
yellow
yellowgreen

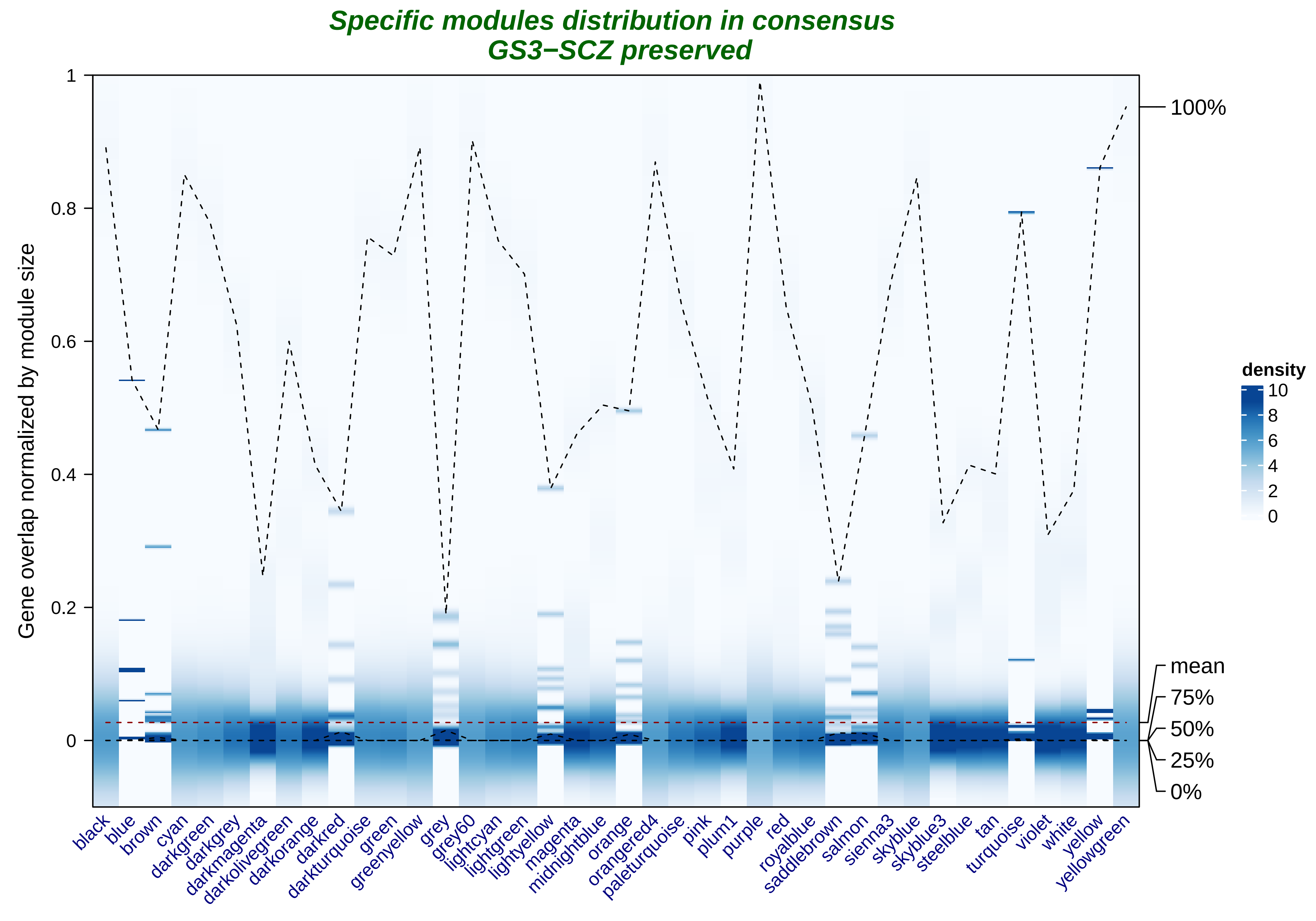

Specific modules distribution in consensus
GS3−SCZ preserved
Gene overlap normalized by module size
1
0.8
0.6
0.4
0.2
0
100%
mean
75%
50%
25%
0%
density
10
8
6
4
2
0
black
blue
brown
cyan
darkgreen
darkgrey
darkmagenta
darkolivegreen
darkorange
darkred
darkturquoise
green
greenyellow
grey
grey60
lightcyan
lightgreen
lightyellow
magenta
midnightblue
orange
orangered4
paleturquoise
pink
plum1
purple
red
royalblue
saddlebrown
salmon
sienna3
skyblue
skyblue3
steelblue
tan
turquoise
violet
white
yellow
yellowgreen

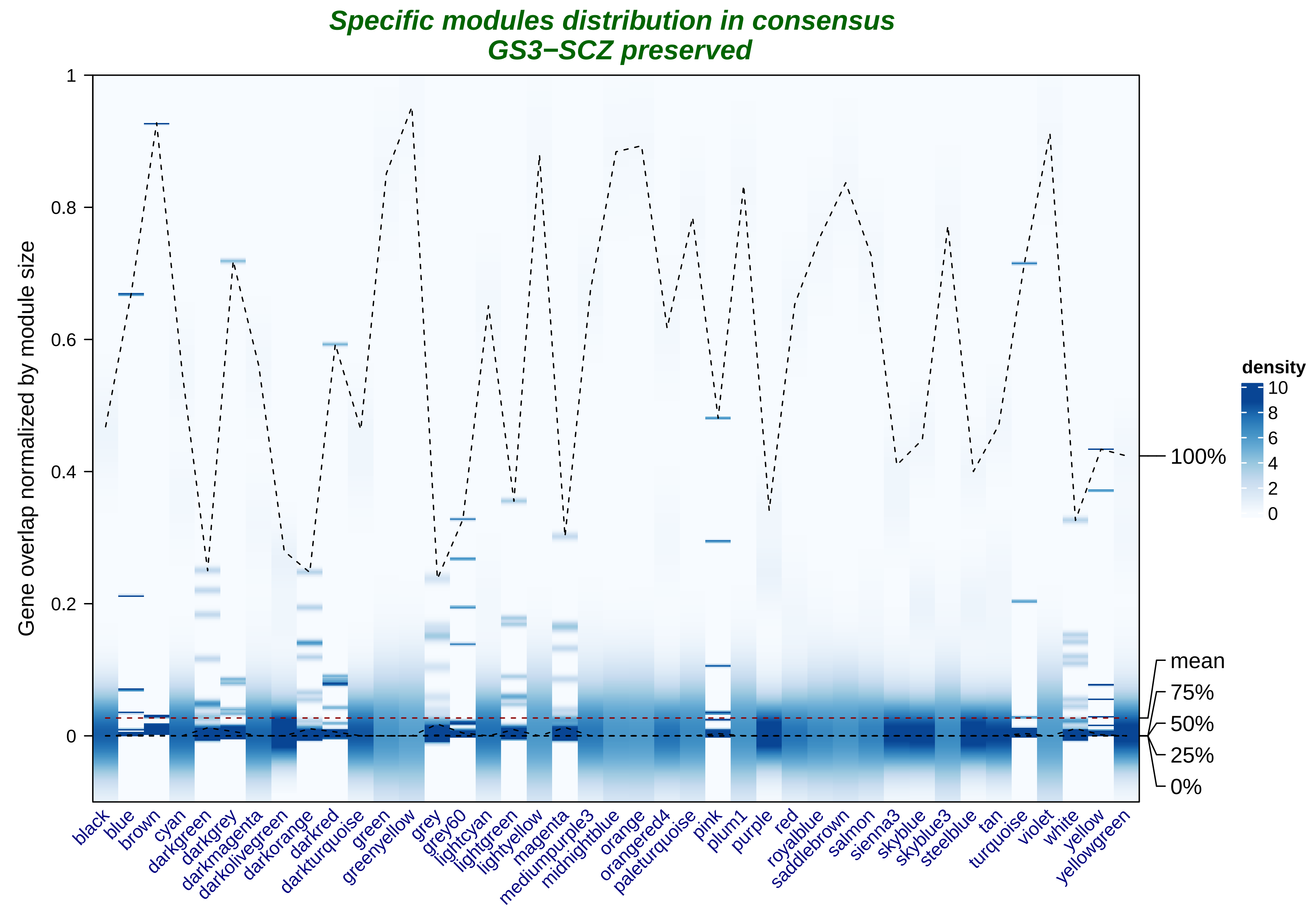

Specific modules distribution in consensus
GS3−SCZ preserved
Gene overlap normalized by module size
1
0.8
0.6
0.4
0.2
0
density
10
8
6
4
2
0
100%
mean
75%
50%
25%
0%
black
blue
brown
cyan
darkgreen
darkgrey
darkmagenta
darkolivegreen
darkorange
darkred
darkturquoise
green
greenyellow
grey
grey60
lightcyan
lightgreen
lightyellow
magenta
mediumpurple3
midnightblue
orange
orangered4
paleturquoise
pink
plum1
purple
red
royalblue
saddlebrown
salmon
sienna3
skyblue
skyblue3
steelblue
tan
turquoise
violet
white
yellow
yellowgreen

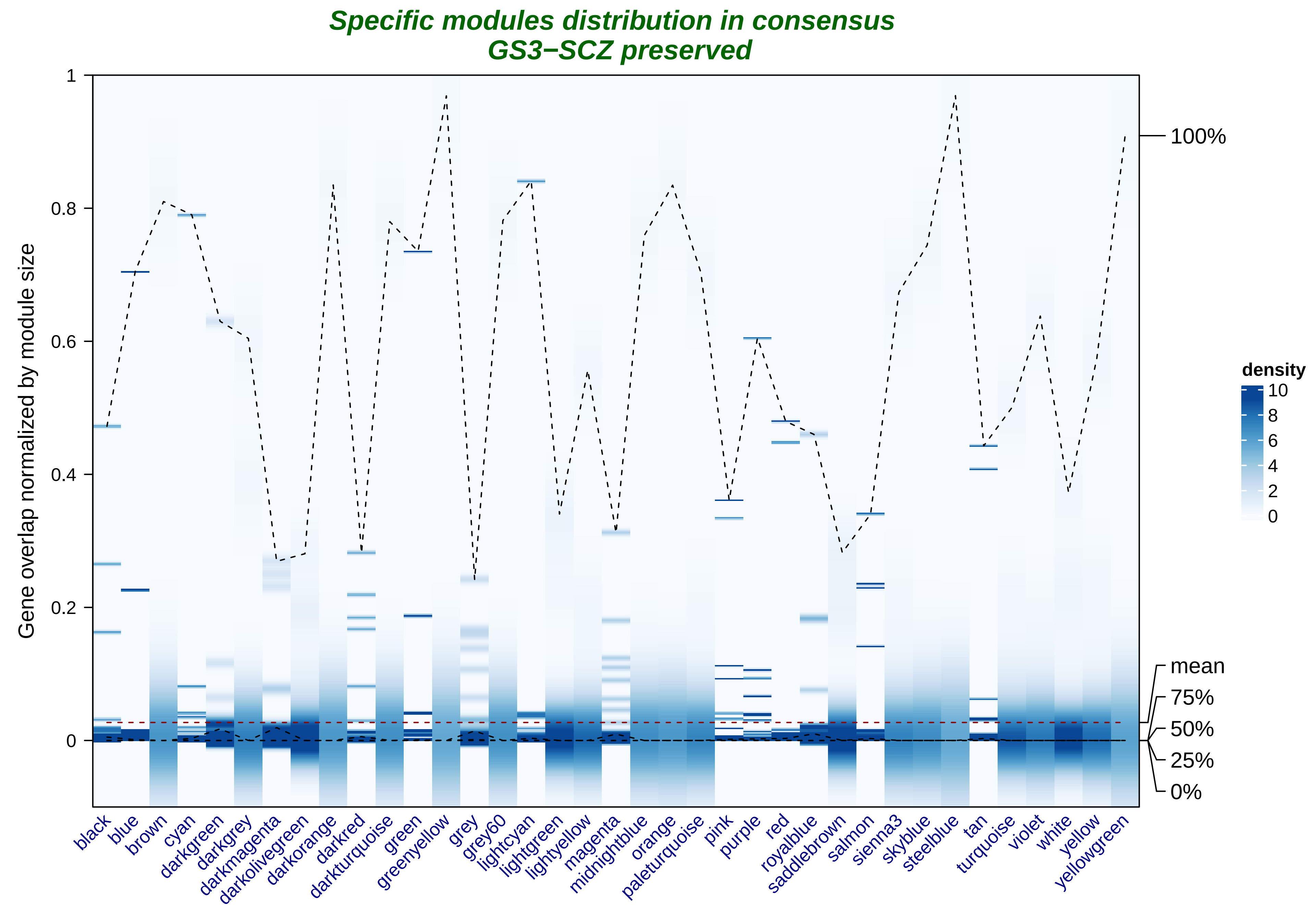
Specific modules distribution in consensus
GS3−SCZ preserved
Gene overlap normalized by module size
1
0.8
0.6
0.4
0.2
0
100%
mean
75%
50%
25%
0%
density
10
8
6
4
2
0
black
blue
brown
cyan
darkgreen
darkgrey
darkmagenta
darkolivegreen
darkorange
darkred
darkturquoise
green
greenyellow
grey
grey60
lightcyan
lightgreen
lightyellow
magenta
midnightblue
orange
paleturquoise
pink
purple
red
royalblue
saddlebrown
salmon
sienna3
skyblue
steelblue
tan
turquoise
violet
white
yellow
yellowgreen

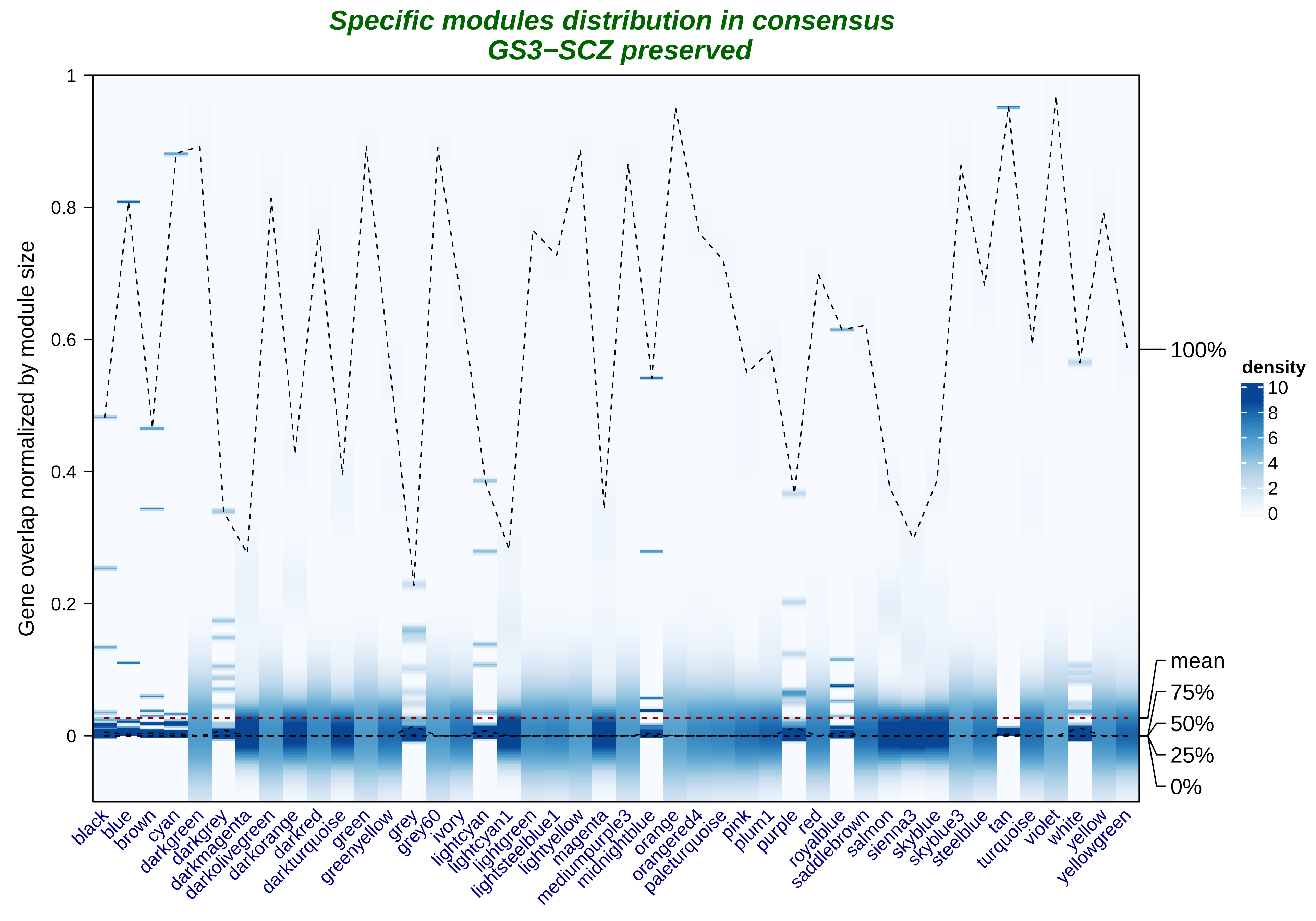

Specific modules distribution in consensus
GS3−SCZ preserved
Gene overlap normalized by module size
1
0.8
0.6
0.4
0.2
0
100%
mean
75%
50%
25%
0%
density
10
8
6
4
2
0
black
blue
brown
cyan
darkgreen
darkgrey
darkmagenta
darkolivegreen
darkorange
darkred
darkturquoise
green
greenyellow
grey
grey60
ivory
lightcyan
lightcyan1
lightgreen
lightsteelblue1
lightyellow
magenta
mediumpurple3
midnightblue
orange
orangered4
paleturquoise
pink
plum1
purple
red
royalblue
saddlebrown
salmon
sienna3
skyblue
skyblue3
steelblue
tan
turquoise
violet
white
yellow
yellowgreen

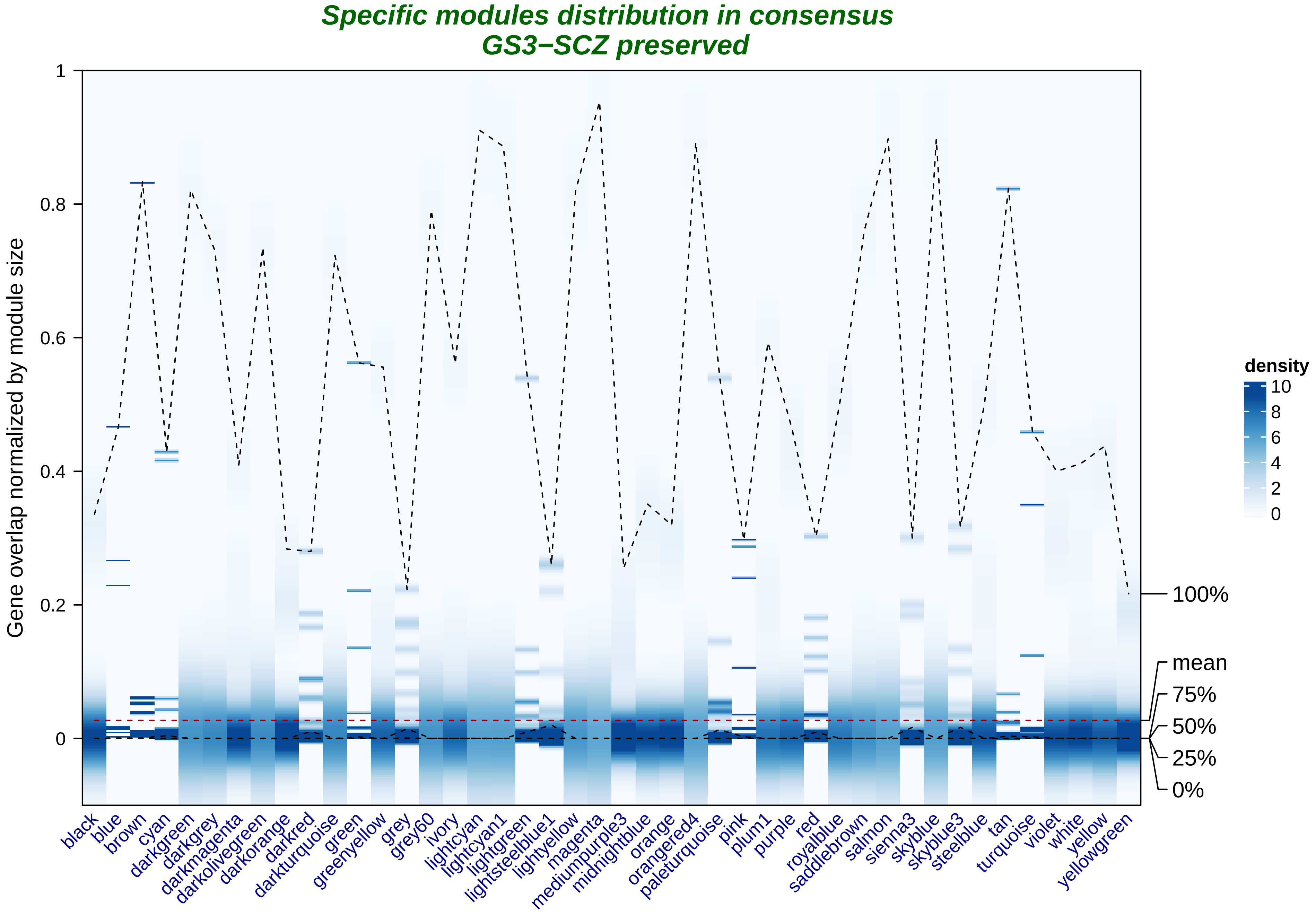

Specific modules distribution in consensus
GS3−SCZ preserved
Gene overlap normalized by module size
1
0.8
0.6
0.4
0.2
0
density
10
8
6
4
2
0
100%
mean
75%
50%
25%
0%
black
blue
brown
cyan
darkgreen
darkgrey
darkmagenta
darkolivegreen
darkorange
darkred
darkturquoise
green
greenyellow
grey
grey60
ivory
lightcyan
lightcyan1
lightgreen
lightsteelblue1
lightyellow
magenta
mediumpurple3
midnightblue
orange
orangered4
paleturquoise
pink
plum1
purple
red
royalblue
saddlebrown
salmon
sienna3
skyblue
skyblue3
steelblue
tan
turquoise
violet
white
yellow
yellowgreen

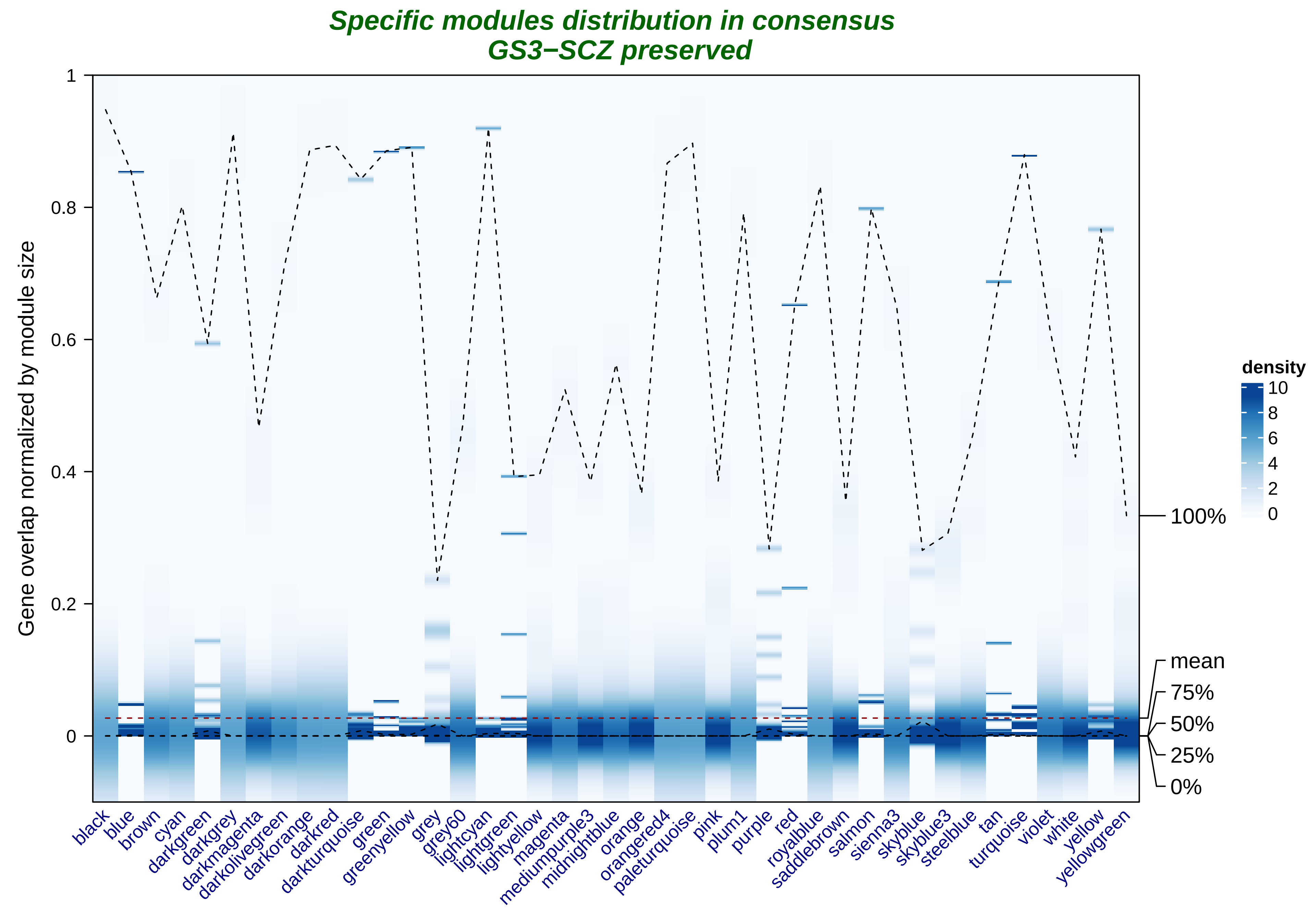
Specific modules distribution in consensus
GS3−SCZ preserved
Gene overlap normalized by module size
1
0.8
0.6
0.4
0.2
0
black
blue
brown
cyan
darkgreen
darkgrey
darkmagenta
darkolivegreen
darkorange
darkred
darkturquoise
green
greenyellow
grey
grey60
lightcyan
lightgreen
lightyellow
magenta
mediumpurple3
midnightblue
orange
orangered4
paleturquoise
pink
plum1
purple
red
royalblue
saddlebrown
salmon
sienna3
skyblue
skyblue3
steelblue
tan
turquoise
violet
white
yellow
yellowgreen
100%
mean
75%
50%
25%
0%
density
10
8
6
4
2
0

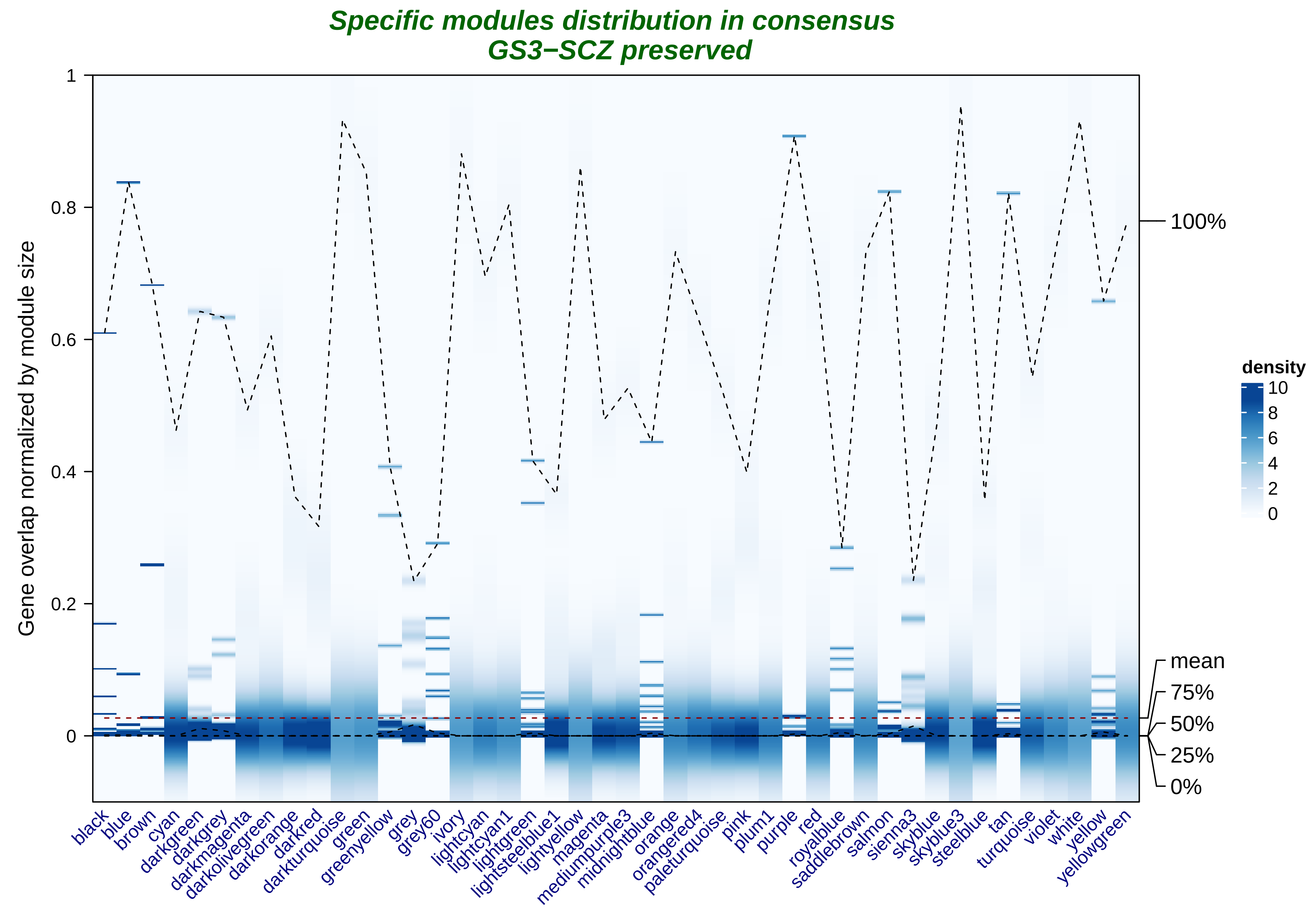

Specific modules distribution in consensus
GS3−SCZ preserved
Gene overlap normalized by module size
1
0.8
0.6
0.4
0.2
0
100%
mean
75%
50%
25%
0%
density
10
8
6
4
2
0
black
blue
brown
cyan
darkgreen
darkgrey
darkmagenta
darkolivegreen
darkorange
darkred
darkturquoise
green
greenyellow
grey
grey60
ivory
lightcyan
lightcyan1
lightgreen
lightsteelblue1
lightyellow
magenta
mediumpurple3
midnightblue
orange
orangered4
paleturquoise
pink
plum1
purple
red
royalblue
saddlebrown
salmon
sienna3
skyblue
skyblue3
steelblue
tan
turquoise
violet
white
yellow
yellowgreen

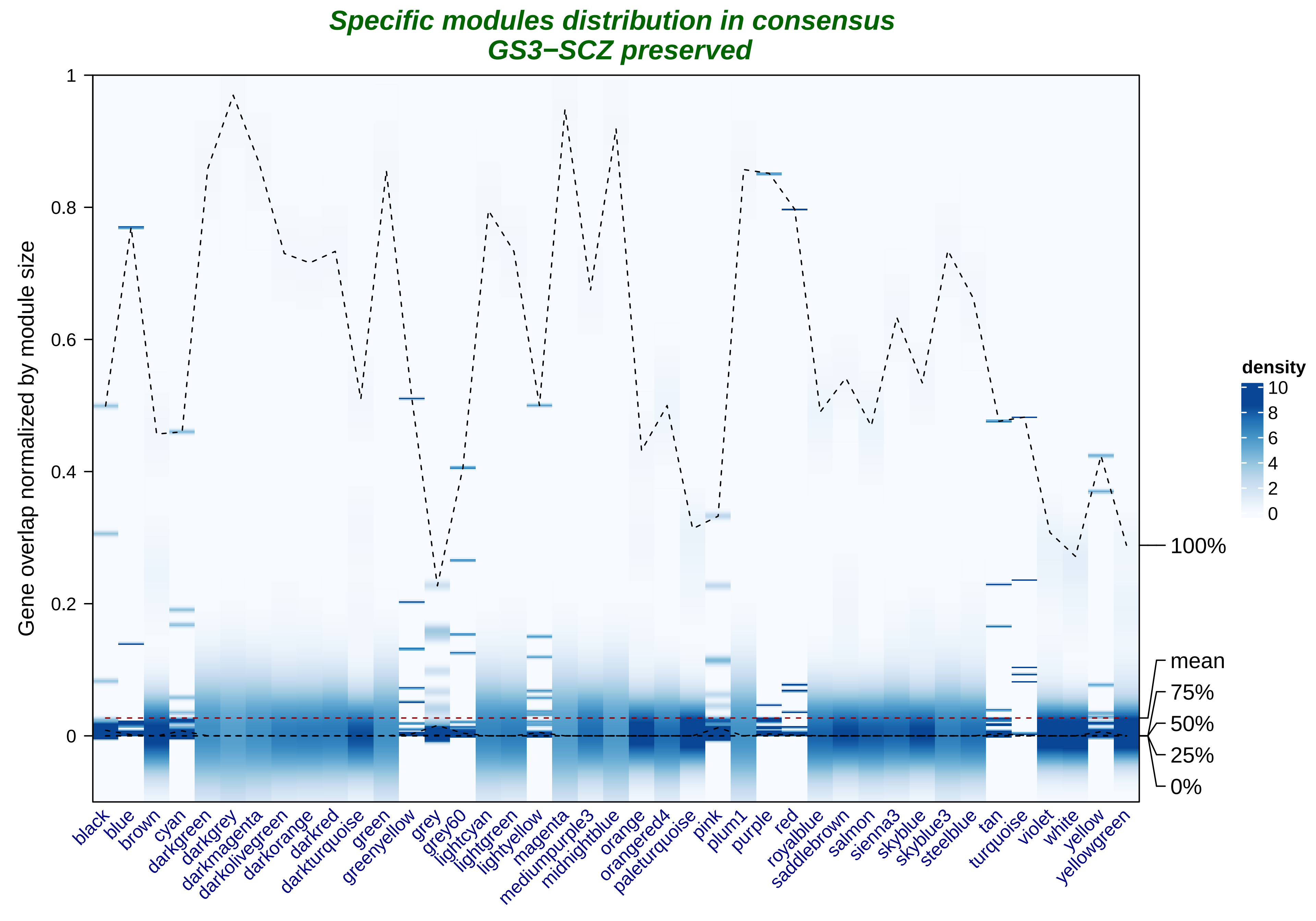

Specific modules distribution in consensus
GS3−SCZ preserved
Gene overlap normalized by module size
1
0.8
0.6
0.4
0.2
0
black
blue
brown
cyan
darkgreen
darkgrey
darkmagenta
darkolivegreen
darkorange
darkred
darkturquoise
green
greenyellow
grey
grey60
lightcyan
lightgreen
lightyellow
magenta
mediumpurple3
midnightblue
orange
orangered4
paleturquoise
pink
plum1
purple
red
royalblue
saddlebrown
salmon
sienna3
skyblue
skyblue3
steelblue
tan
turquoise
violet
white
yellow
yellowgreen
100%
mean
75%
50%
25%
0%
density
10
8
6
4
2
0

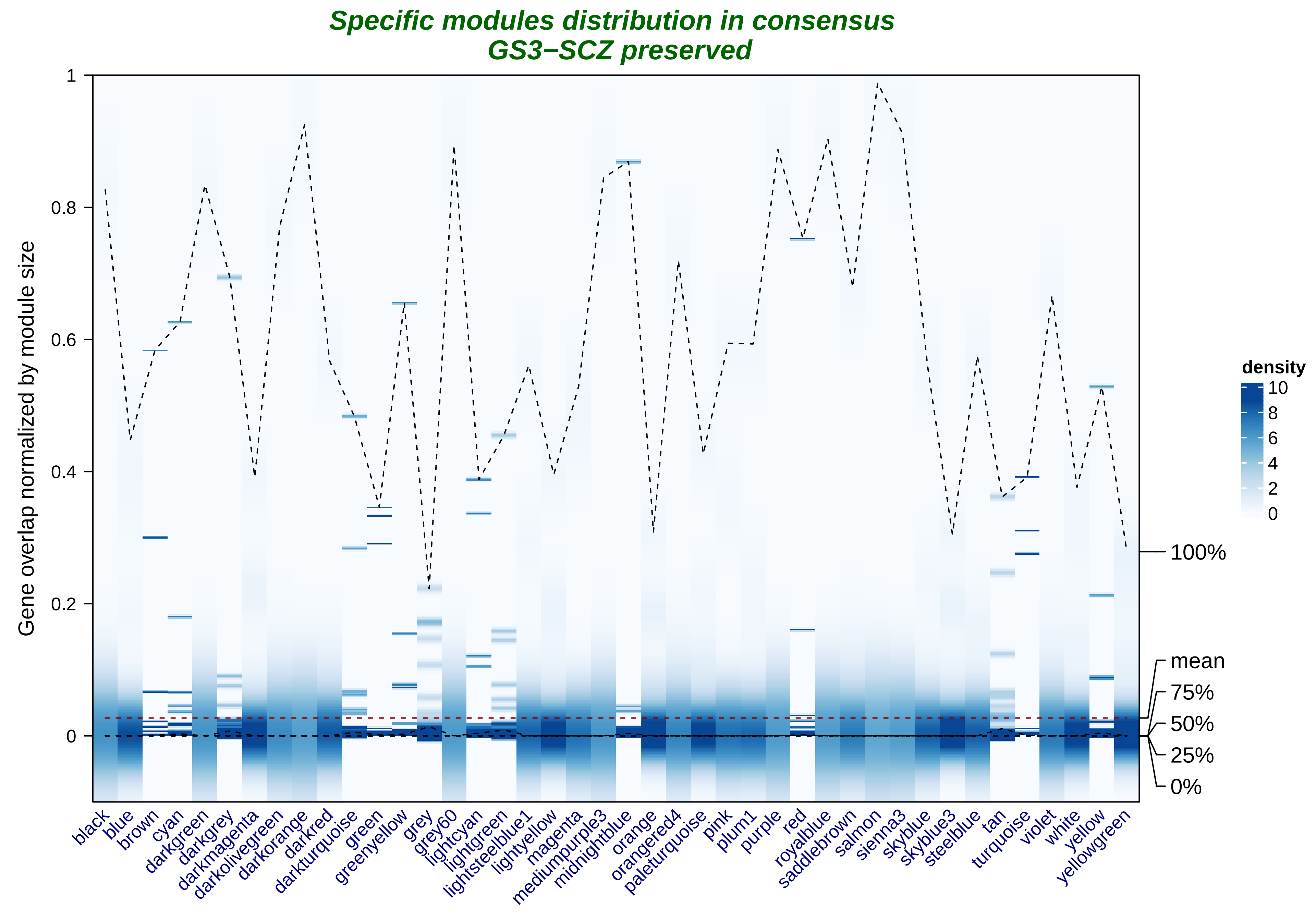

Specific modules distribution in consensus
GS3−SCZ preserved
Gene overlap normalized by module size
1
0.8
0.6
0.4
0.2
0
density
10
8
6
4
2
0
100%
mean
75%
50%
25%
0%
black
blue
brown
cyan
darkgreen
darkgrey
darkmagenta
darkolivegreen
darkorange
darkred
darkturquoise
green
greenyellow
grey
grey60
lightcyan
lightgreen
lightsteelblue1
lightyellow
magenta
mediumpurple3
midnightblue
orange
orangered4
paleturquoise
pink
plum1
purple
red
royalblue
saddlebrown
salmon
sienna3
skyblue
skyblue3
steelblue
tan
turquoise
violet
white
yellow
yellowgreen

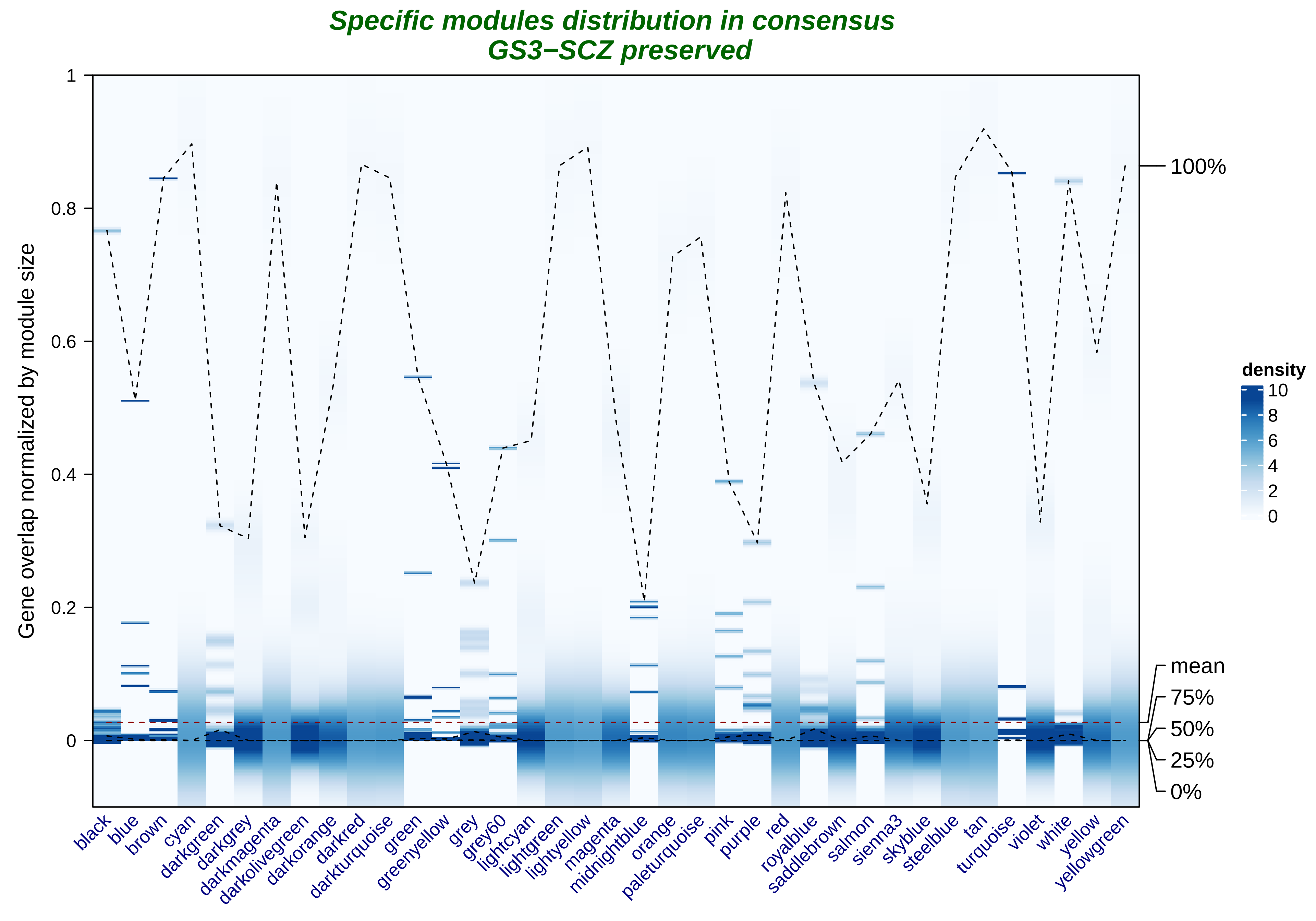

Specific modules distribution in consensus
GS3−SCZ preserved
Gene overlap normalized by module size
1
0.8
0.6
0.4
0.2
0
100%
mean
75%
50%
25%
0%
density
10
8
6
4
2
0
black
blue
brown
cyan
darkgreen
darkgrey
darkmagenta
darkolivegreen
darkorange
darkred
darkturquoise
green
greenyellow
grey
grey60
lightcyan
lightgreen
lightyellow
magenta
midnightblue
orange
paleturquoise
pink
purple
red
royalblue
saddlebrown
salmon
sienna3
skyblue
steelblue
tan
turquoise
violet
white
yellow
yellowgreen

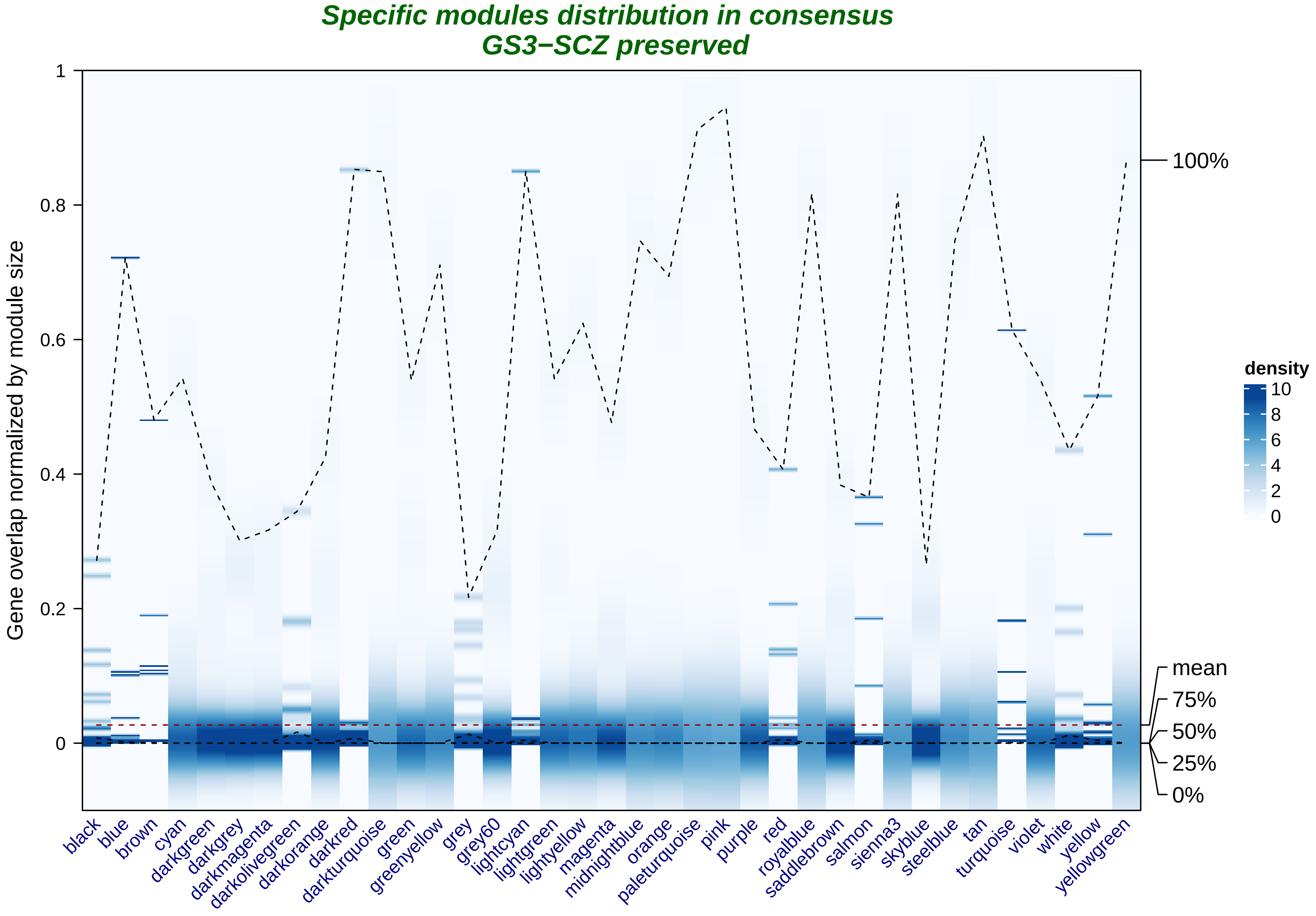

Specific modules distribution in consensus
GS3−SCZ preserved
Gene overlap normalized by module size
1
0.8
0.6
0.4
0.2
0
100%
mean
75%
50%
25%
0%
density
10
8
6
4
2
0
black
blue
brown
cyan
darkgreen
darkgrey
darkmagenta
darkolivegreen
darkorange
darkred
darkturquoise
green
greenyellow
grey
grey60
lightcyan
lightgreen
lightyellow
magenta
midnightblue
orange
paleturquoise
pink
purple
red
royalblue
saddlebrown
salmon
sienna3
skyblue
steelblue
tan
turquoise
violet
white
yellow
yellowgreen

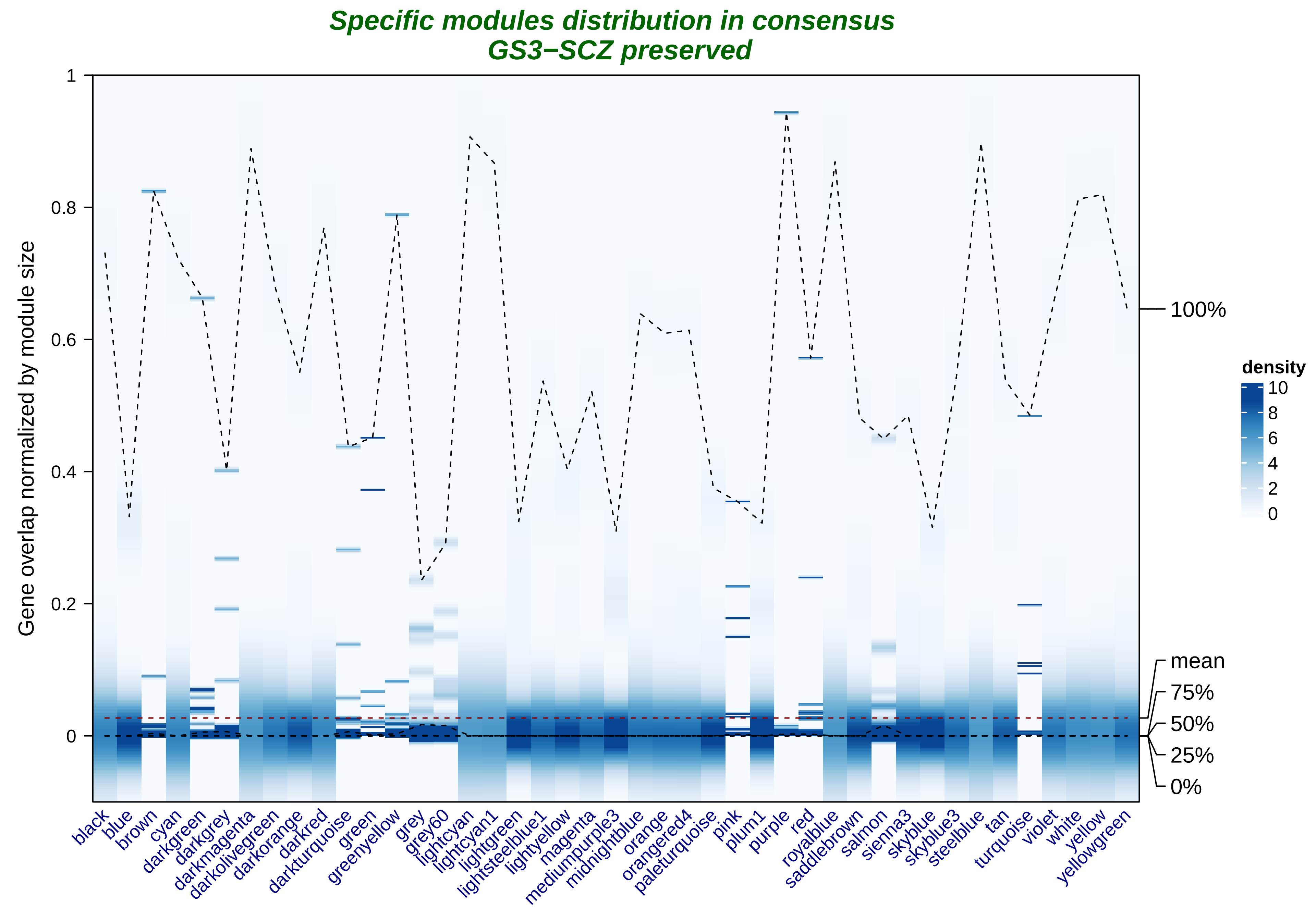

Specific modules distribution in consensus
GS3−SCZ preserved
Gene overlap normalized by module size
1
0.8
0.6
0.4
0.2
0
100%
mean
75%
50%
25%
0%
density
10
8
6
4
2
0
black
blue
brown
cyan
darkgreen
darkgrey
darkmagenta
darkolivegreen
darkorange
darkred
darkturquoise
green
greenyellow
grey
grey60
lightcyan
lightcyan1
lightgreen
lightsteelblue1
lightyellow
magenta
mediumpurple3
midnightblue
orange
orangered4
paleturquoise
pink
plum1
purple
red
royalblue
saddlebrown
salmon
sienna3
skyblue
skyblue3
steelblue
tan
turquoise
violet
white
yellow
yellowgreen

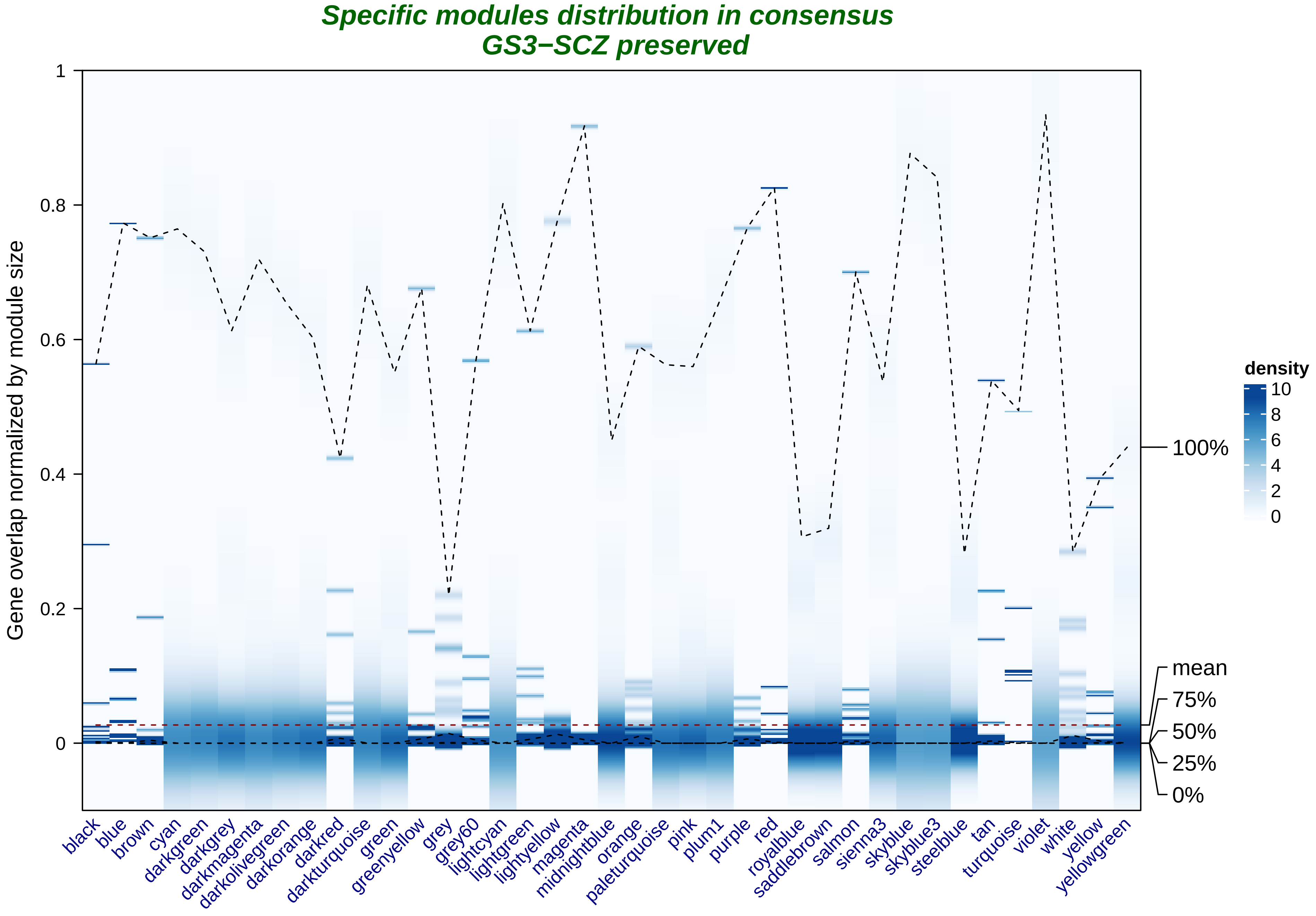

Specific modules distribution in consensus
GS3−SCZ preserved
Gene overlap normalized by module size
1
0.8
0.6
0.4
0.2
0
density
10
8
6
4
2
0
100%
mean
75%
50%
25%
0%
black
blue
brown
cyan
darkgreen
darkgrey
darkmagenta
darkolivegreen
darkorange
darkred
darkturquoise
green
greenyellow
grey
grey60
lightcyan
lightgreen
lightyellow
magenta
midnightblue
orange
paleturquoise
pink
plum1
purple
red
royalblue
saddlebrown
salmon
sienna3
skyblue
skyblue3
steelblue
tan
turquoise
violet
white
yellow
yellowgreen

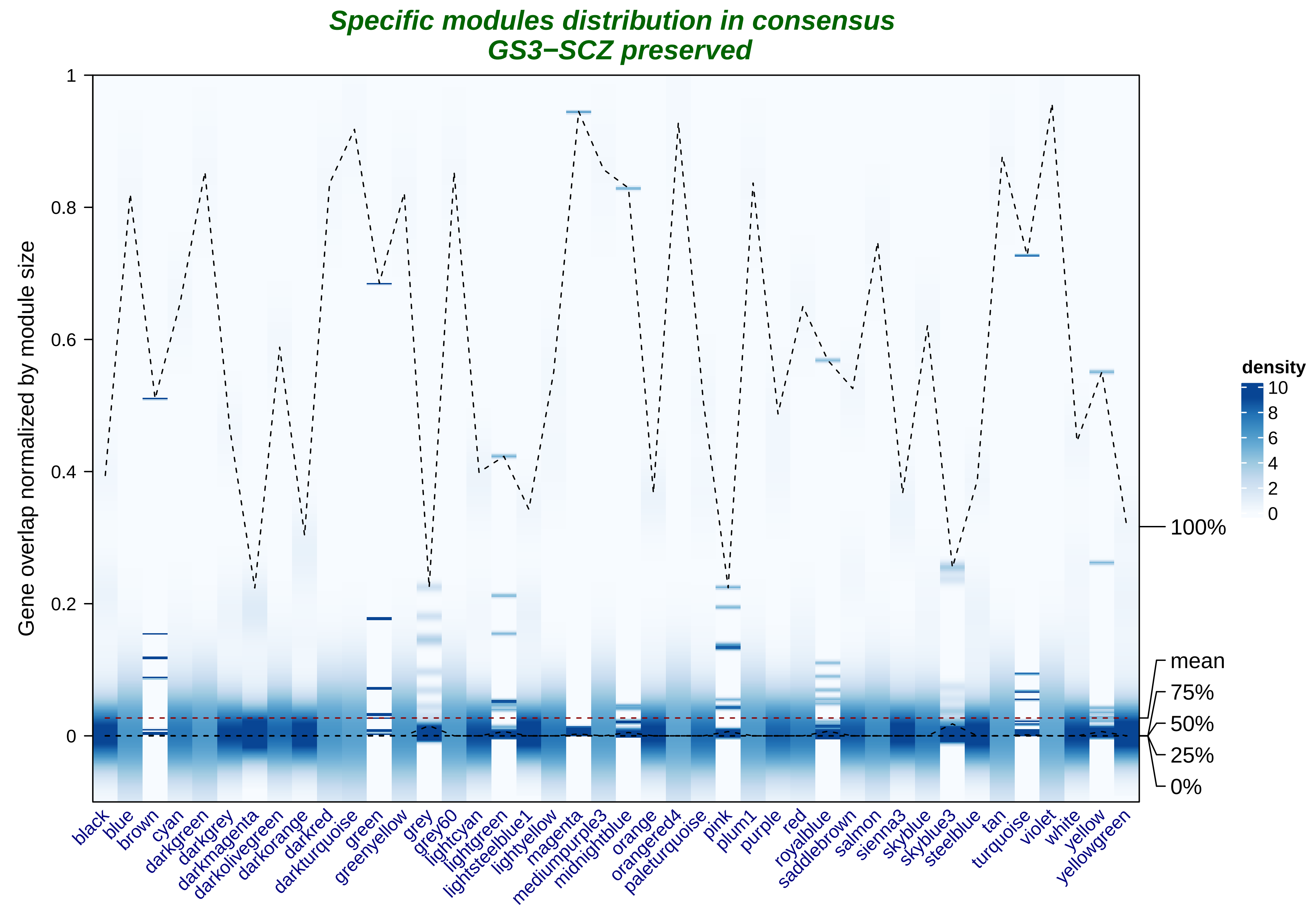
Specific modules distribution in consensus
GS3−SCZ preserved
Gene overlap normalized by module size
1
0.8
0.6
0.4
0.2
0
density
10
8
6
4
2
0
100%
mean
75%
50%
25%
0%
black
blue
brown
cyan
darkgreen
darkgrey
darkmagenta
darkolivegreen
darkorange
darkred
darkturquoise
green
greenyellow
grey
grey60
lightcyan
lightgreen
lightsteelblue1
lightyellow
magenta
mediumpurple3
midnightblue
orange
orangered4
paleturquoise
pink
plum1
purple
red
royalblue
saddlebrown
salmon
sienna3
skyblue
skyblue3
steelblue
tan
turquoise
violet
white
yellow
yellowgreen

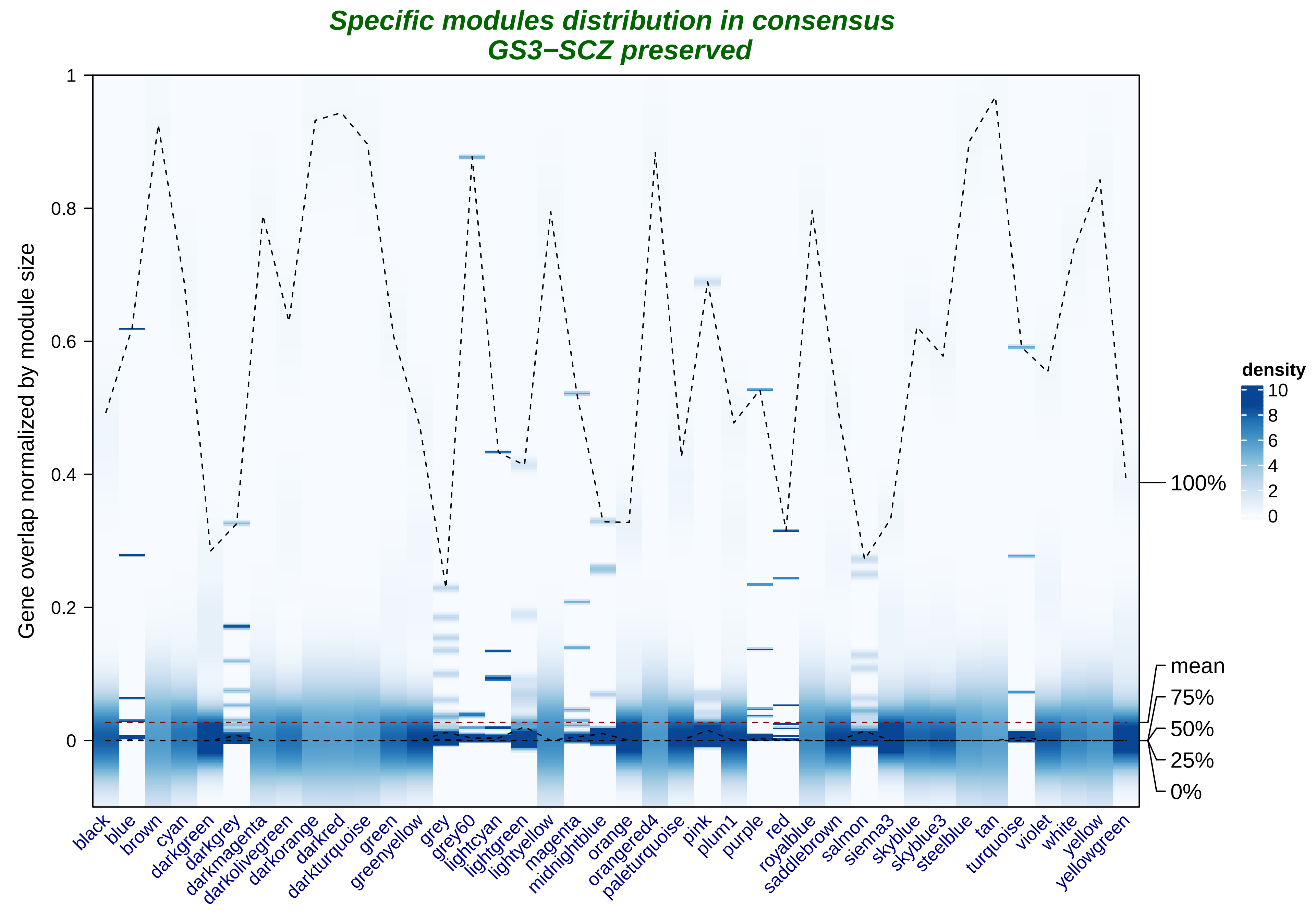

Specific modules distribution in consensus
GS3−SCZ preserved
Gene overlap normalized by module size
1
0.8
0.6
0.4
0.2
0
density
10
8
6
4
2
0
100%
mean
75%
50%
25%
0%
black
blue
brown
cyan
darkgreen
darkgrey
darkmagenta
darkolivegreen
darkorange
darkred
darkturquoise
green
greenyellow
grey
grey60
lightcyan
lightgreen
lightyellow
magenta
midnightblue
orange
orangered4
paleturquoise
pink
plum1
purple
red
royalblue
saddlebrown
salmon
sienna3
skyblue
skyblue3
steelblue
tan
turquoise
violet
white
yellow
yellowgreen

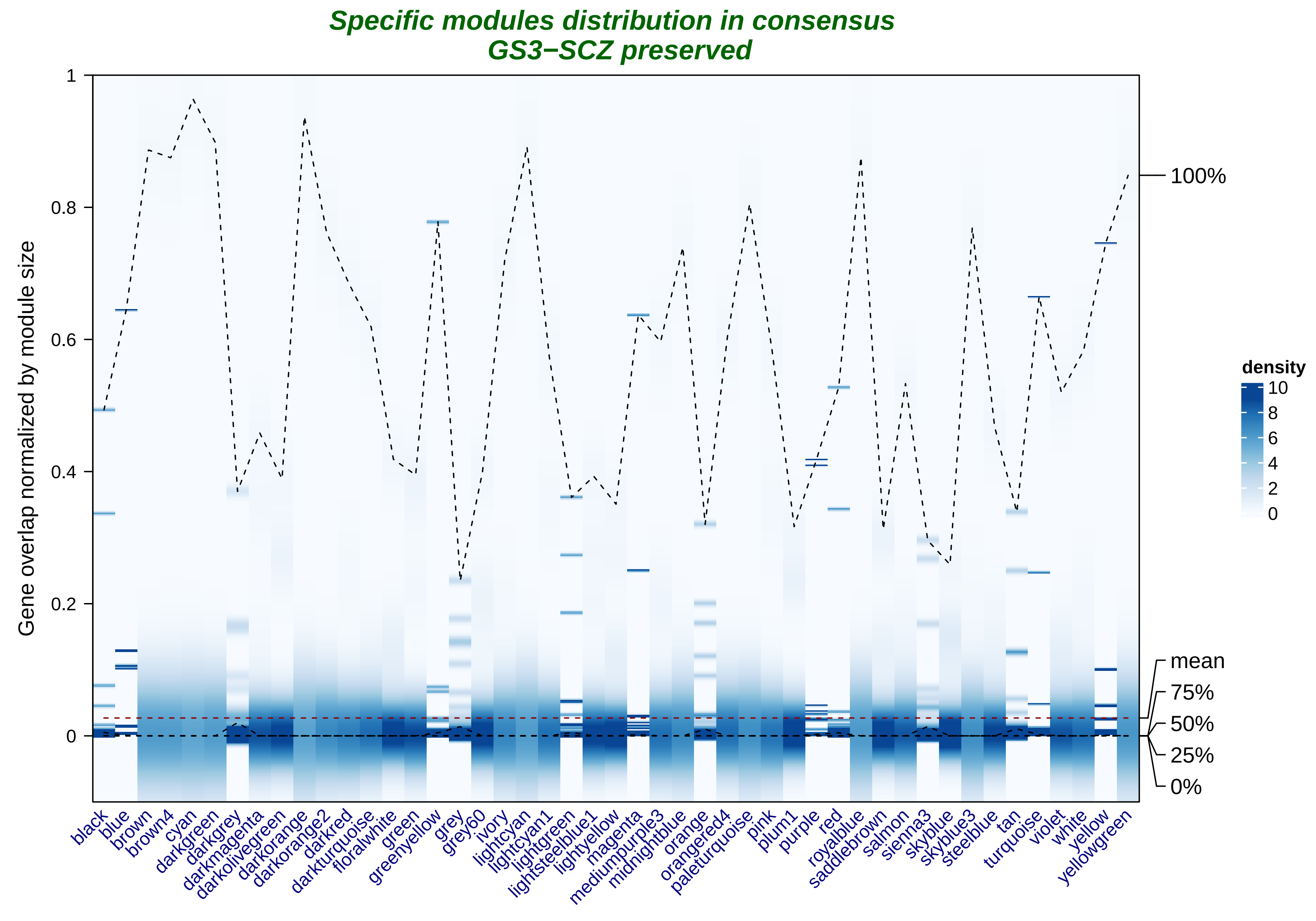

Specific modules distribution in consensus
GS3−SCZ preserved
Gene overlap normalized by module size
1
0.8
0.6
0.4
0.2
0
100%
mean
75%
50%
25%
0%
density
10
8
6
4
2
0
black
blue
brown
brown4
cyan
darkgreen
darkgrey
darkmagenta
darkolivegreen
darkorange
darkorange2
darkred
darkturquoise
floralwhite
green
greenyellow
grey
grey60
ivory
lightcyan
lightcyan1
lightgreen
lightsteelblue1
lightyellow
magenta
mediumpurple3
midnightblue
orange
orangered4
paleturquoise
pink
plum1
purple
red
royalblue
saddlebrown
salmon
sienna3
skyblue
skyblue3
steelblue
tan
turquoise
violet
white
yellow
yellowgreen

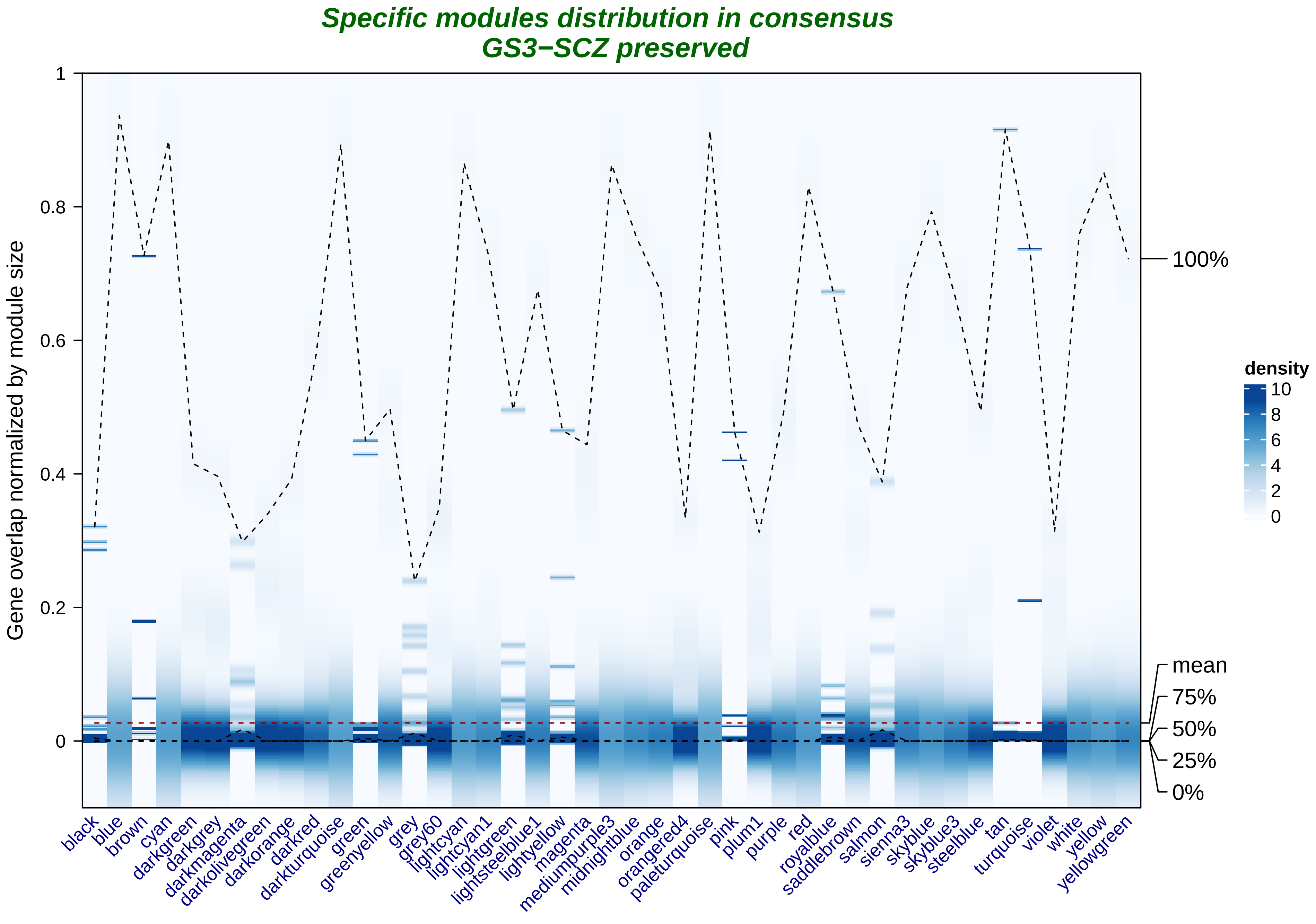

Specific modules distribution in consensus
GS3−SCZ preserved
Gene overlap normalized by module size
1
0.8
0.6
0.4
0.2
0
100%
mean
75%
50%
25%
0%
density
10
8
6
4
2
0
black
blue
brown
cyan
darkgreen
darkgrey
darkmagenta
darkolivegreen
darkorange
darkred
darkturquoise
green
greenyellow
grey
grey60
lightcyan
lightcyan1
lightgreen
lightsteelblue1
lightyellow
magenta
mediumpurple3
midnightblue
orange
orangered4
paleturquoise
pink
plum1
purple
red
royalblue
saddlebrown
salmon
sienna3
skyblue
skyblue3
steelblue
tan
turquoise
violet
white
yellow
yellowgreen

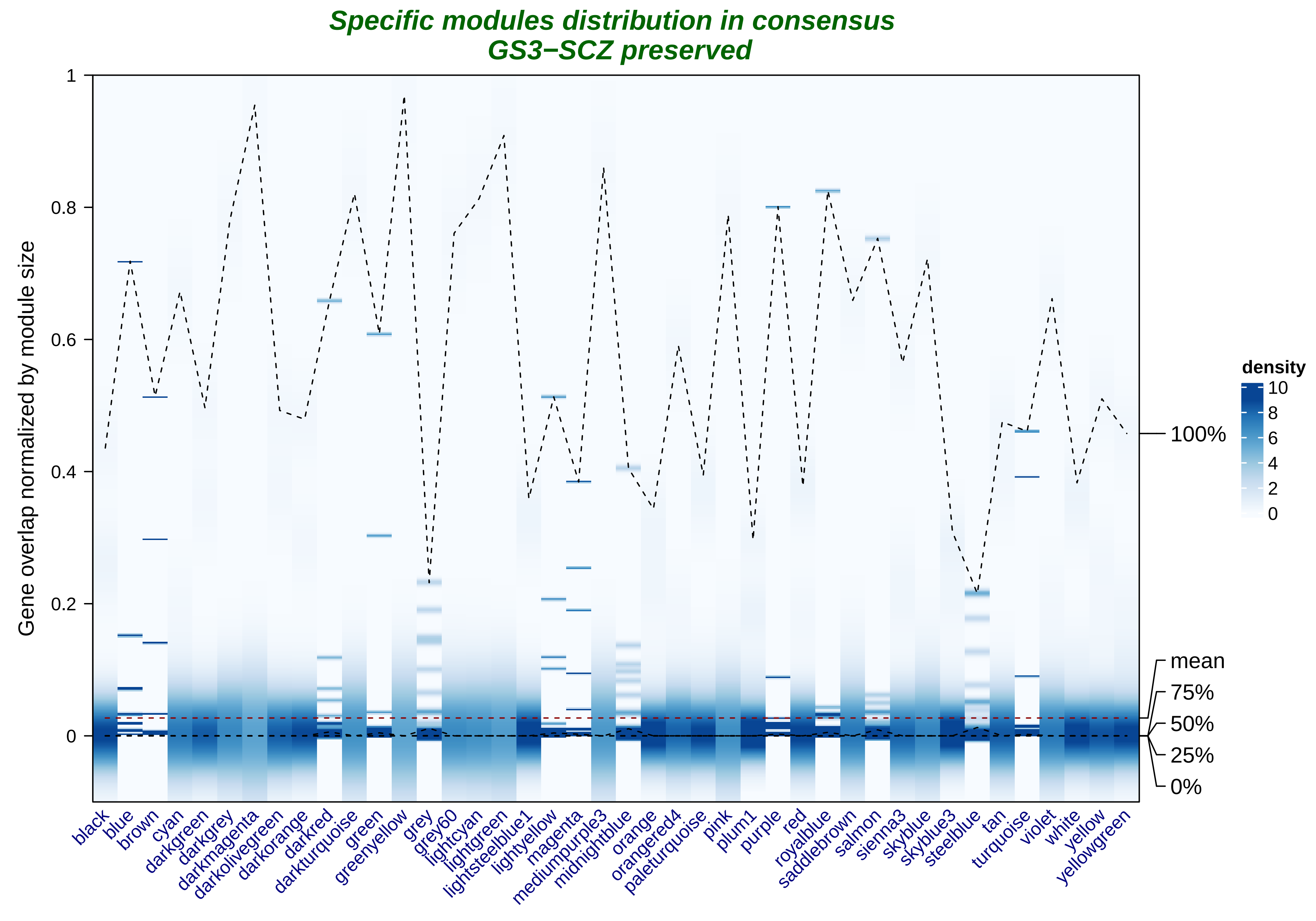

Specific modules distribution in consensus
GS3−SCZ preserved
Gene overlap normalized by module size
1
0.8
0.6
0.4
0.2
0
density
10
8
6
4
2
0
100%
mean
75%
50%
25%
0%
black
blue
brown
cyan
darkgreen
darkgrey
darkmagenta
darkolivegreen
darkorange
darkred
darkturquoise
green
greenyellow
grey
grey60
lightcyan
lightgreen
lightsteelblue1
lightyellow
magenta
mediumpurple3
midnightblue
orange
orangered4
paleturquoise
pink
plum1
purple
red
royalblue
saddlebrown
salmon
sienna3
skyblue
skyblue3
steelblue
tan
turquoise
violet
white
yellow
yellowgreen

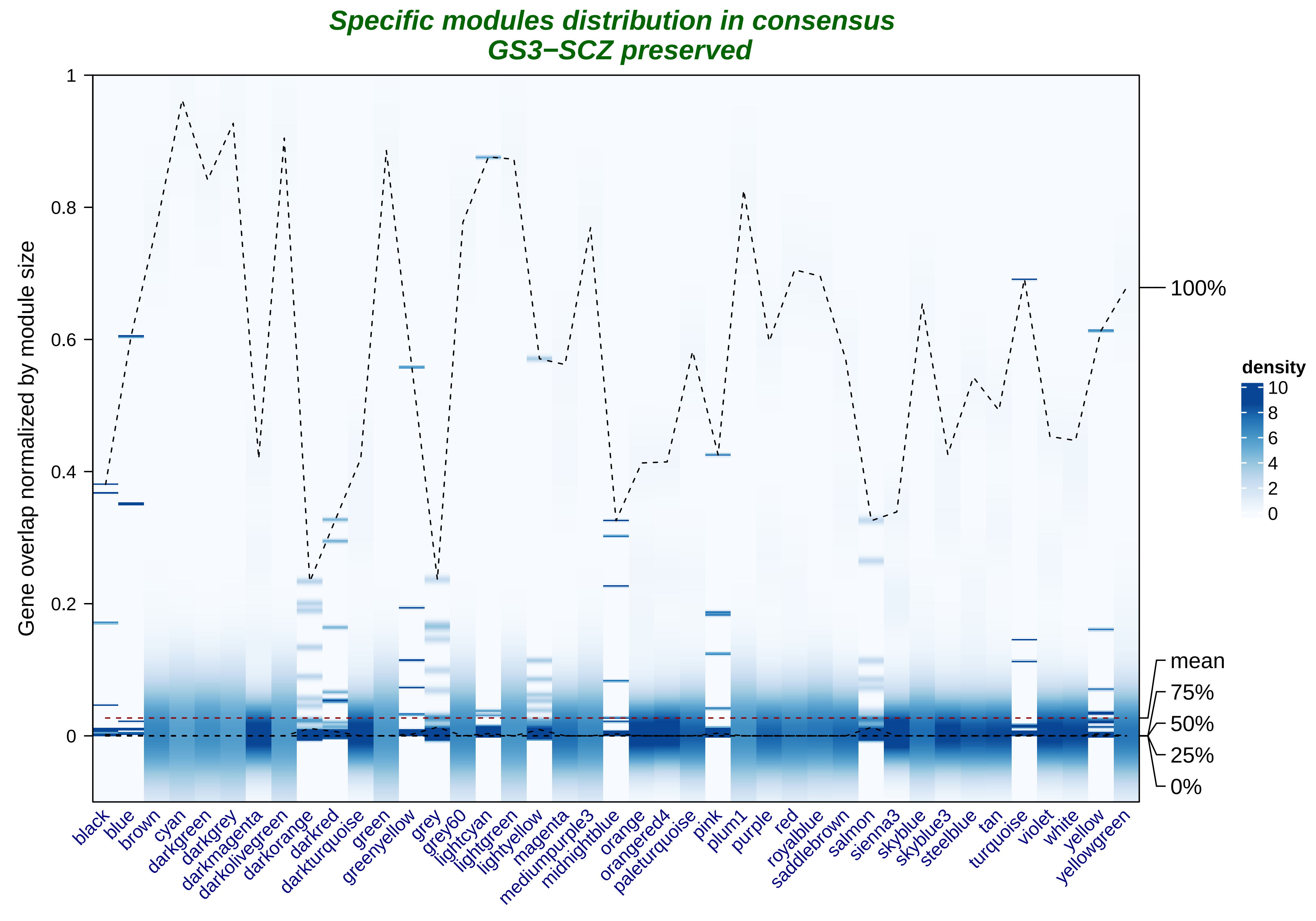

Specific modules distribution in consensus
GS3−SCZ preserved
Gene overlap normalized by module size
1
0.8
0.6
0.4
0.2
0
100%
mean
75%
50%
25%
0%
density
10
8
6
4
2
0
black
blue
brown
cyan
darkgreen
darkgrey
darkmagenta
darkolivegreen
darkorange
darkred
darkturquoise
green
greenyellow
grey
grey60
lightcyan
lightgreen
lightyellow
magenta
mediumpurple3
midnightblue
orange
orangered4
paleturquoise
pink
plum1
purple
red
royalblue
saddlebrown
salmon
sienna3
skyblue
skyblue3
steelblue
tan
turquoise
violet
white
yellow
yellowgreen

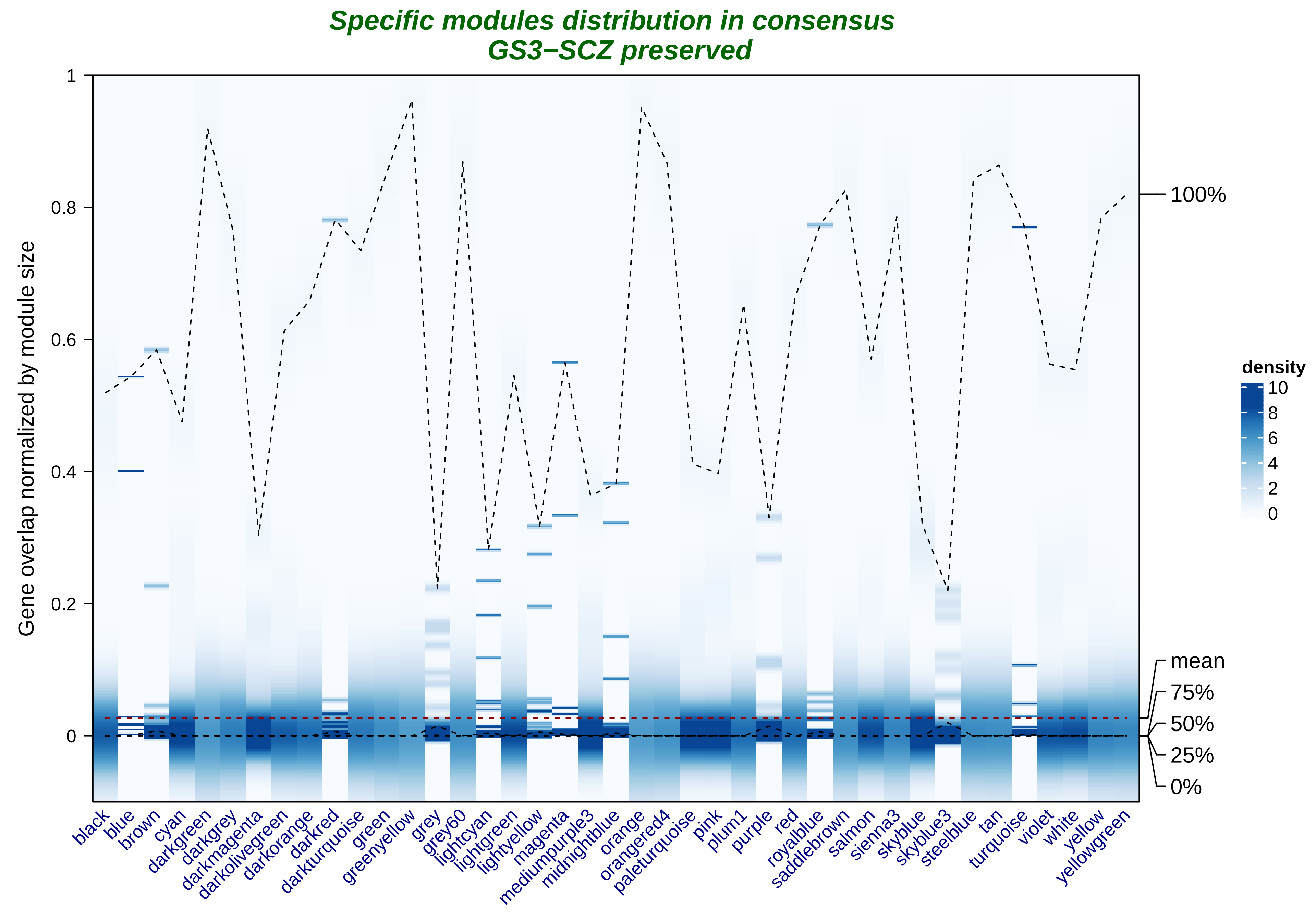

Specific modules distribution in consensus
GS3−SCZ preserved
Gene overlap normalized by module size
1
0.8
0.6
0.4
0.2
0
100%
mean
75%
50%
25%
0%
density
10
8
6
4
2
0
black
blue
brown
cyan
darkgreen
darkgrey
darkmagenta
darkolivegreen
darkorange
darkred
darkturquoise
green
greenyellow
grey
grey60
lightcyan
lightgreen
lightyellow
magenta
mediumpurple3
midnightblue
orange
orangered4
paleturquoise
pink
plum1
purple
red
royalblue
saddlebrown
salmon
sienna3
skyblue
skyblue3
steelblue
tan
turquoise
violet
white
yellow
yellowgreen

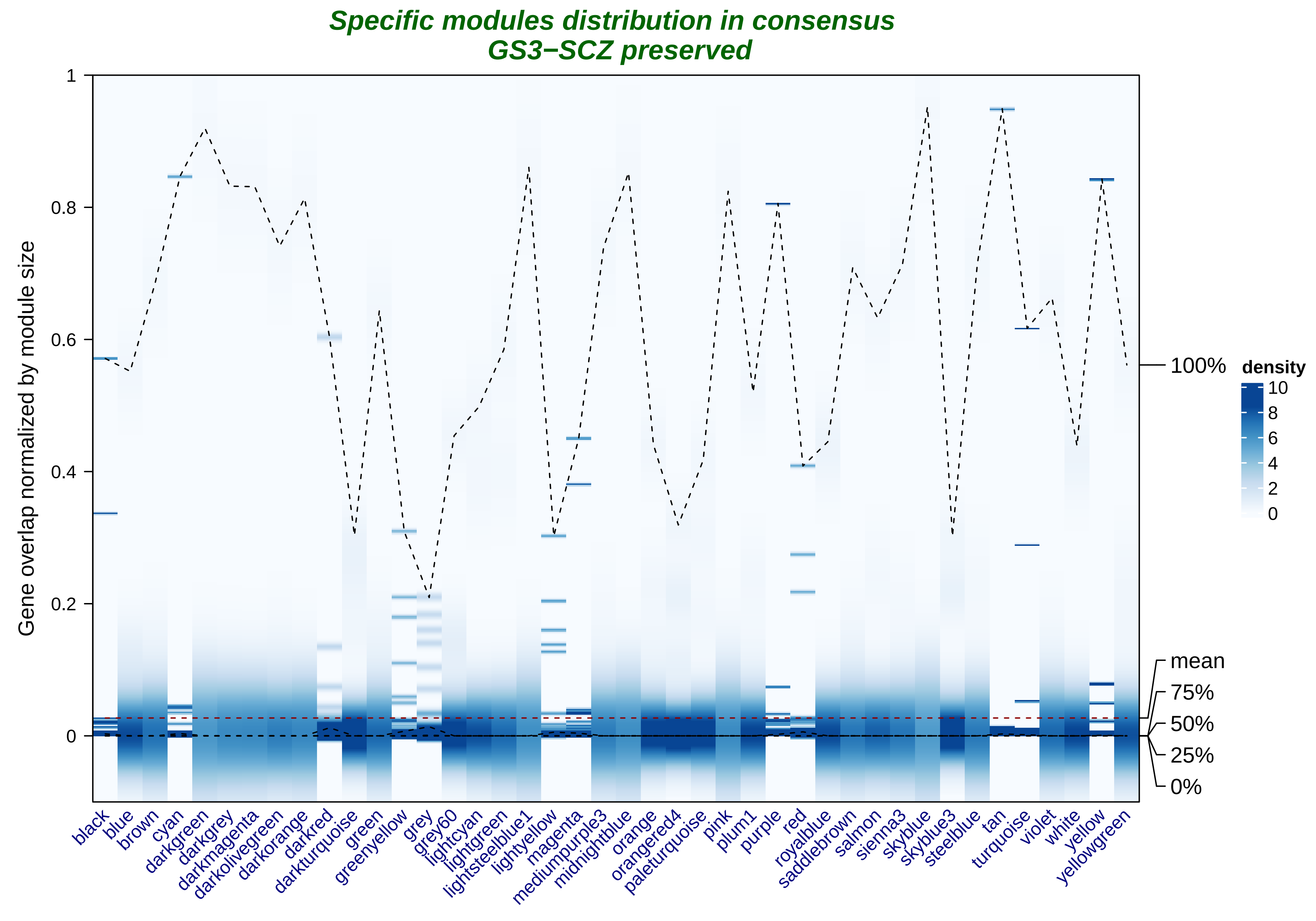

Specific modules distribution in consensus
GS3−SCZ preserved
Gene overlap normalized by module size
1
0.8
0.6
0.4
0.2
0
100%
mean
75%
50%
25%
0%
density
10
8
6
4
2
0
black
blue
brown
cyan
darkgreen
darkgrey
darkmagenta
darkolivegreen
darkorange
darkred
darkturquoise
green
greenyellow
grey
grey60
lightcyan
lightgreen
lightsteelblue1
lightyellow
magenta
mediumpurple3
midnightblue
orange
orangered4
paleturquoise
pink
plum1
purple
red
royalblue
saddlebrown
salmon
sienna3
skyblue
skyblue3
steelblue
tan
turquoise
violet
white
yellow
yellowgreen

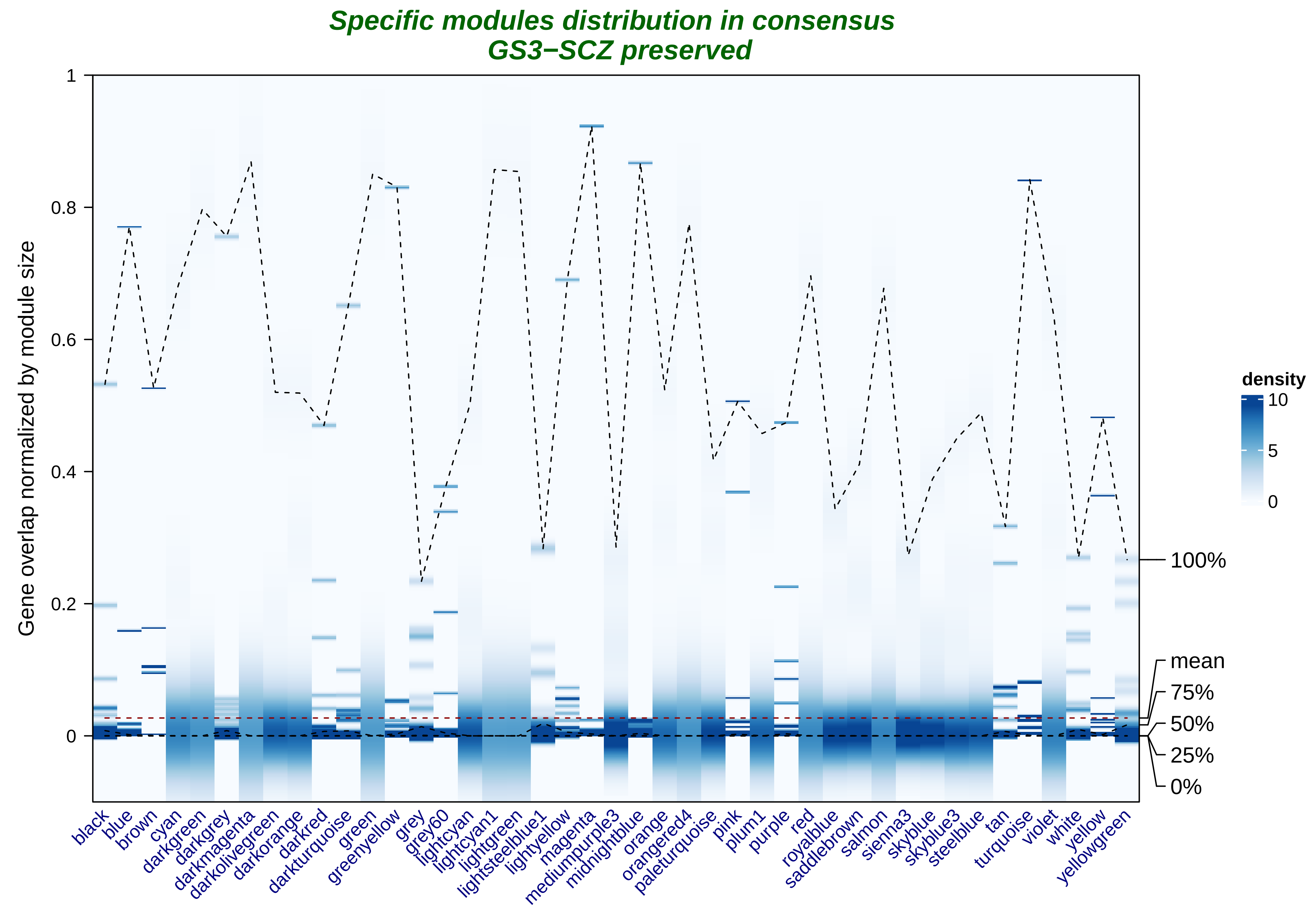

Specific modules distribution in consensus
GS3−SCZ preserved
Gene overlap normalized by module size
1
0.8
0.6
0.4
0.2
0
density
10
5
0
100%
mean
75%
50%
25%
0%
black
blue
brown
cyan
darkgreen
darkgrey
darkmagenta
darkolivegreen
darkorange
darkred
darkturquoise
green
greenyellow
grey
grey60
lightcyan
lightcyan1
lightgreen
lightsteelblue1
lightyellow
magenta
mediumpurple3
midnightblue
orange
orangered4
paleturquoise
pink
plum1
purple
red
royalblue
saddlebrown
salmon
sienna3
skyblue
skyblue3
steelblue
tan
turquoise
violet
white
yellow
yellowgreen

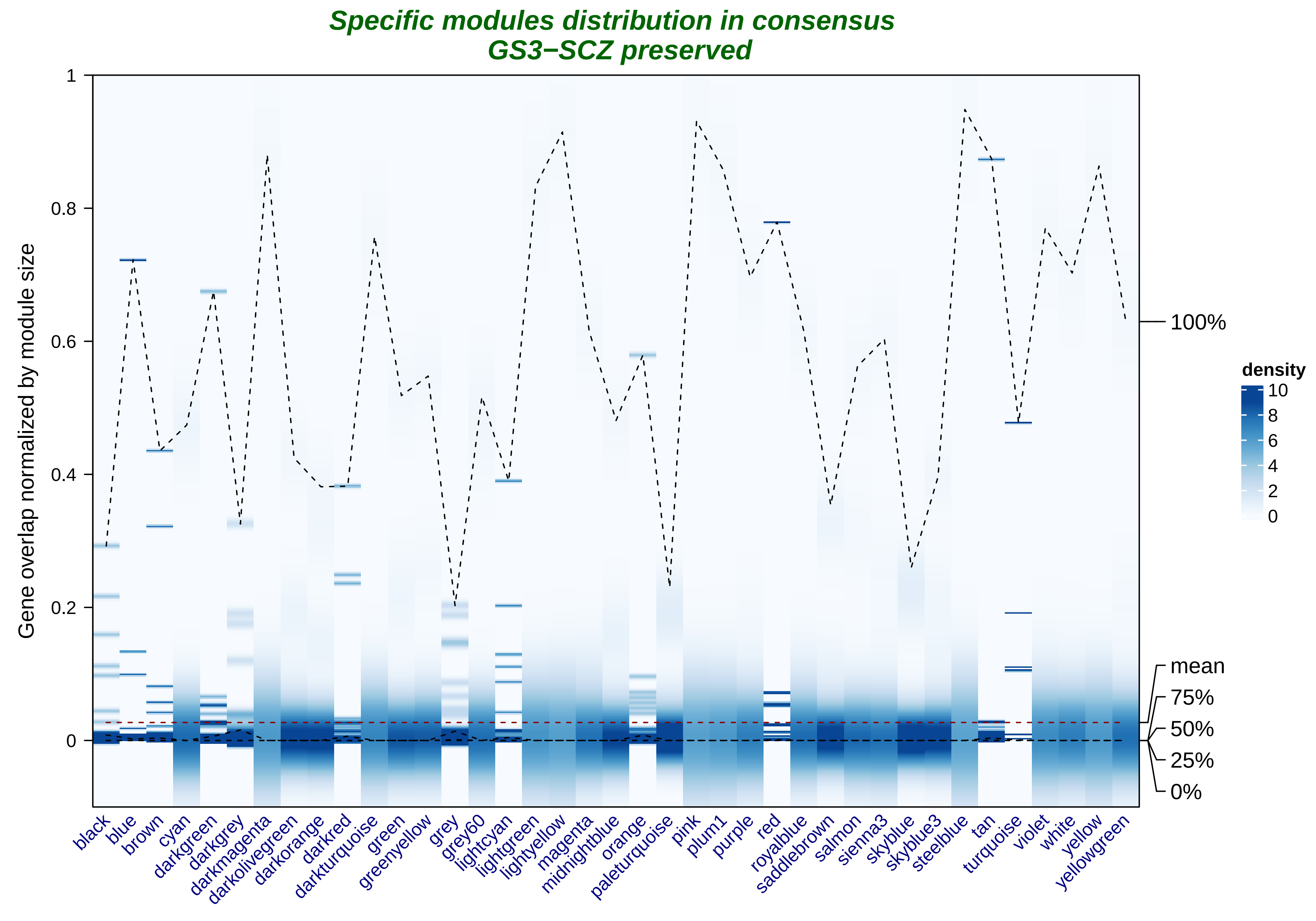
Specific modules distribution in consensus
GS3−SCZ preserved
Gene overlap normalized by module size
1
0.8
0.6
0.4
0.2
0
100%
mean
75%
50%
25%
0%
density
10
8
6
4
2
0
black
blue
brown
cyan
darkgreen
darkgrey
darkmagenta
darkolivegreen
darkorange
darkred
darkturquoise
green
greenyellow
grey
grey60
lightcyan
lightgreen
lightyellow
magenta
midnightblue
orange
paleturquoise
pink
plum1
purple
red
royalblue
saddlebrown
salmon
sienna3
skyblue
skyblue3
steelblue
tan
turquoise
violet
white
yellow
yellowgreen

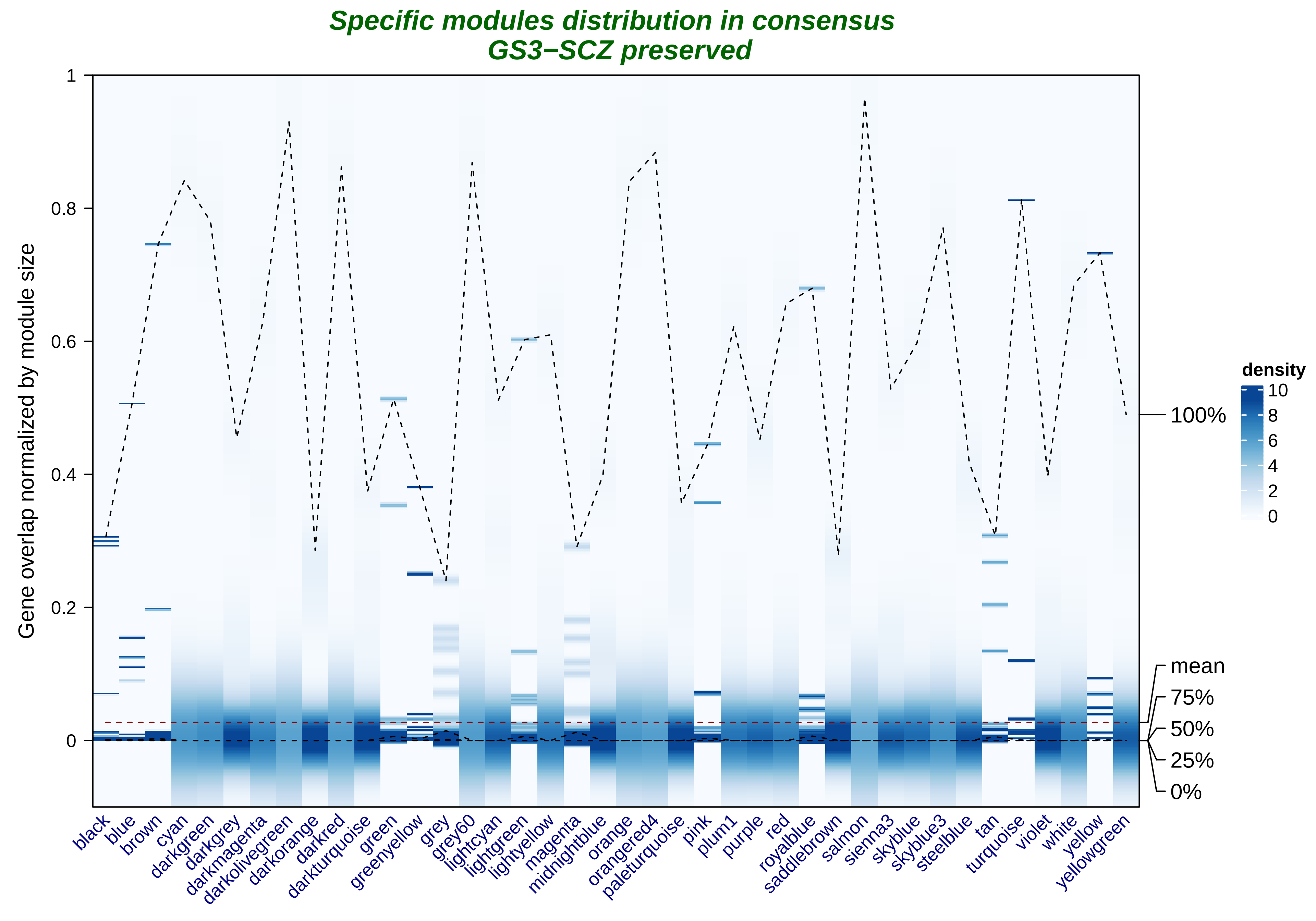

Specific modules distribution in consensus
GS3−SCZ preserved
Gene overlap normalized by module size
1
0.8
0.6
0.4
0.2
0
100%
mean
75%
50%
25%
0%
density
10
8
6
4
2
0
black
blue
brown
cyan
darkgreen
darkgrey
darkmagenta
darkolivegreen
darkorange
darkred
darkturquoise
green
greenyellow
grey
grey60
lightcyan
lightgreen
lightyellow
magenta
midnightblue
orange
orangered4
paleturquoise
pink
plum1
purple
red
royalblue
saddlebrown
salmon
sienna3
skyblue
skyblue3
steelblue
tan
turquoise
violet
white
yellow
yellowgreen

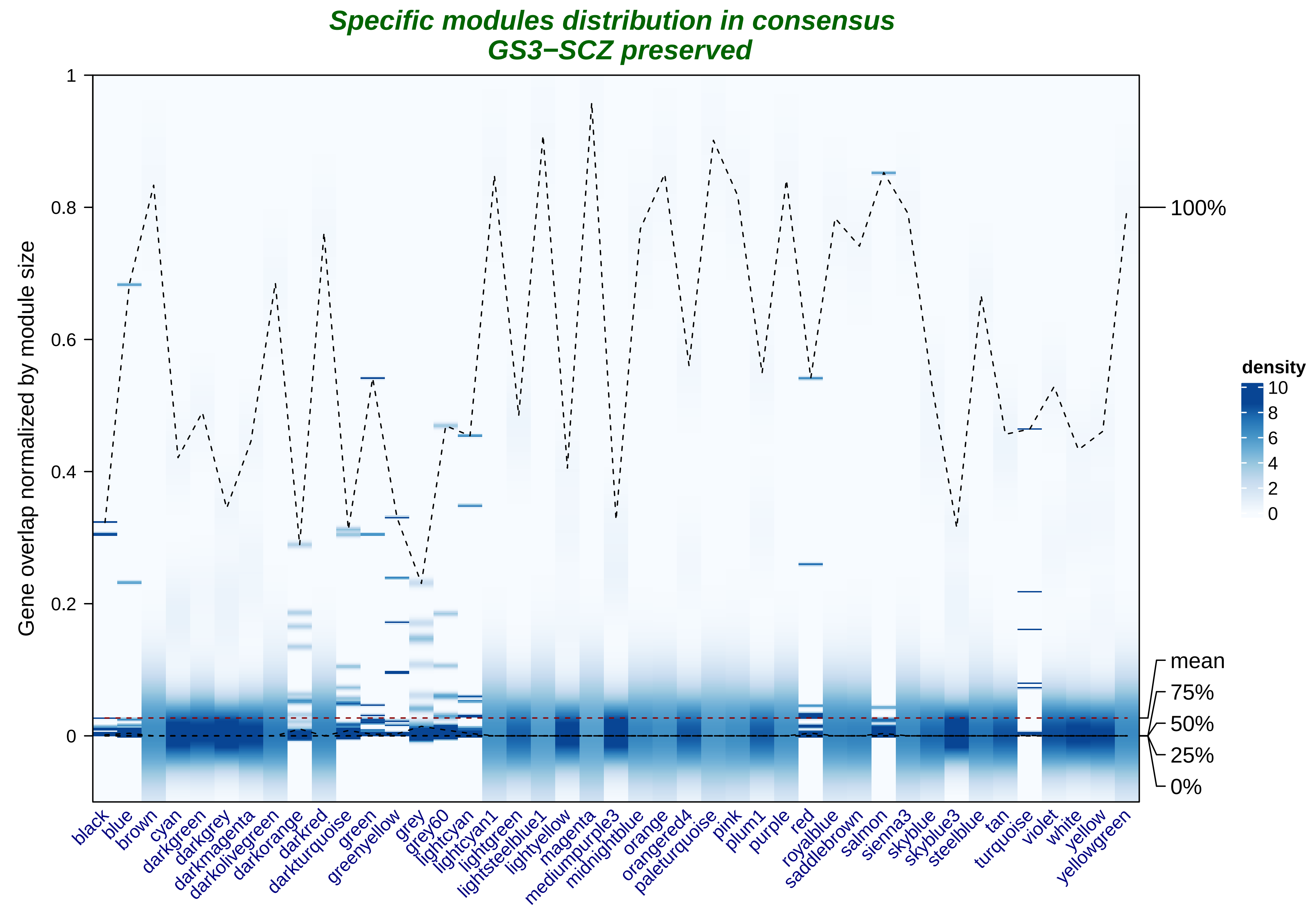

Specific modules distribution in consensus
GS3−SCZ preserved
Gene overlap normalized by module size
1
0.8
0.6
0.4
0.2
0
100%
mean
75%
50%
25%
0%
density
10
8
6
4
2
0
black
blue
brown
cyan
darkgreen
darkgrey
darkmagenta
darkolivegreen
darkorange
darkred
darkturquoise
green
greenyellow
grey
grey60
lightcyan
lightcyan1
lightgreen
lightsteelblue1
lightyellow
magenta
mediumpurple3
midnightblue
orange
orangered4
paleturquoise
pink
plum1
purple
red
royalblue
saddlebrown
salmon
sienna3
skyblue
skyblue3
steelblue
tan
turquoise
violet
white
yellow
yellowgreen

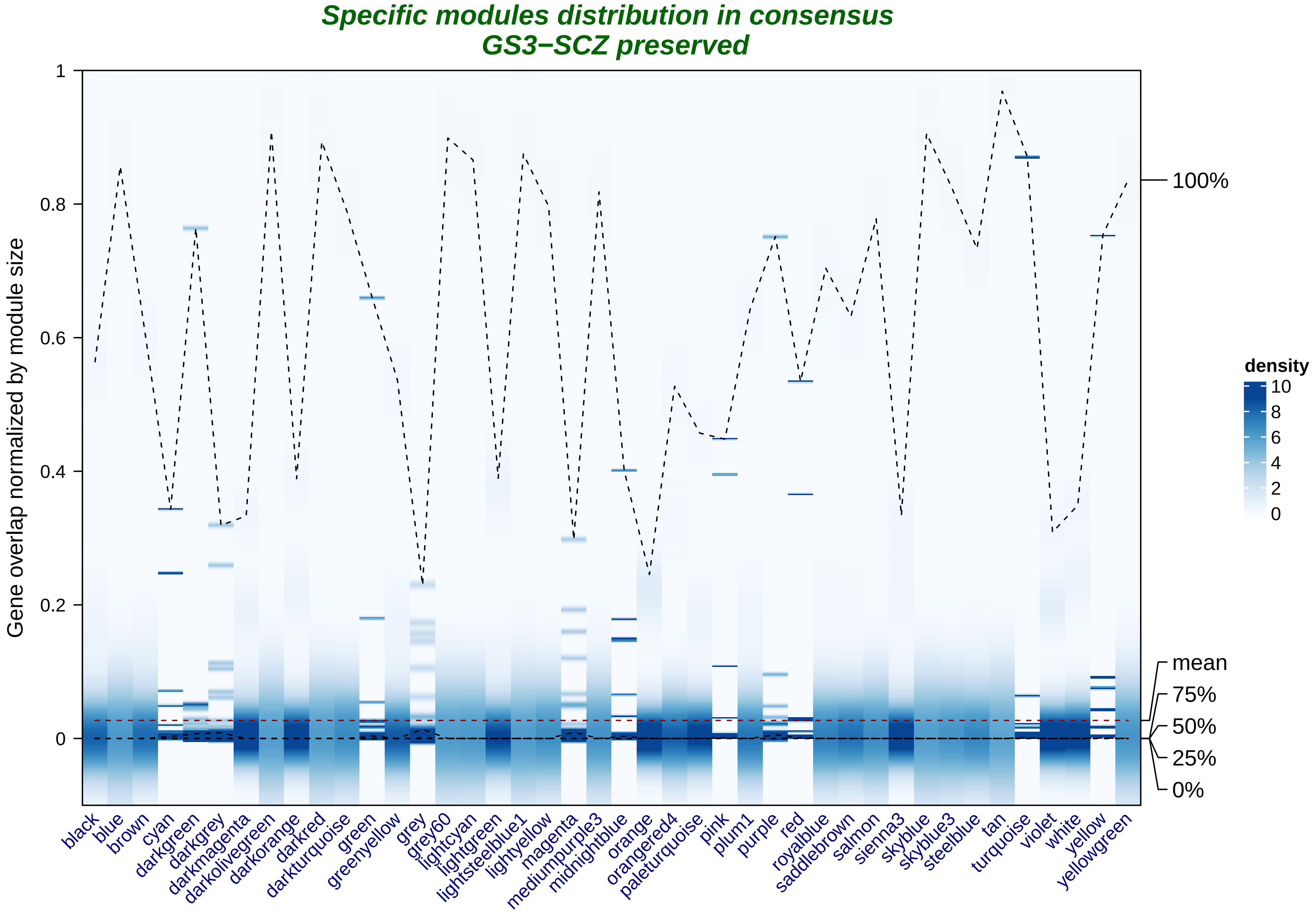

Specific modules distribution in consensus
GS3−SCZ preserved
Gene overlap normalized by module size
1
0.8
0.6
0.4
0.2
0
100%
mean
75%
50%
25%
0%
density
10
8
6
4
2
0
black
blue
brown
cyan
darkgreen
darkgrey
darkmagenta
darkolivegreen
darkorange
darkred
darkturquoise
green
greenyellow
grey
grey60
lightcyan
lightgreen
lightsteelblue1
lightyellow
magenta
mediumpurple3
midnightblue
orange
orangered4
paleturquoise
pink
plum1
purple
red
royalblue
saddlebrown
salmon
sienna3
skyblue
skyblue3
steelblue
tan
turquoise
violet
white
yellow
yellowgreen

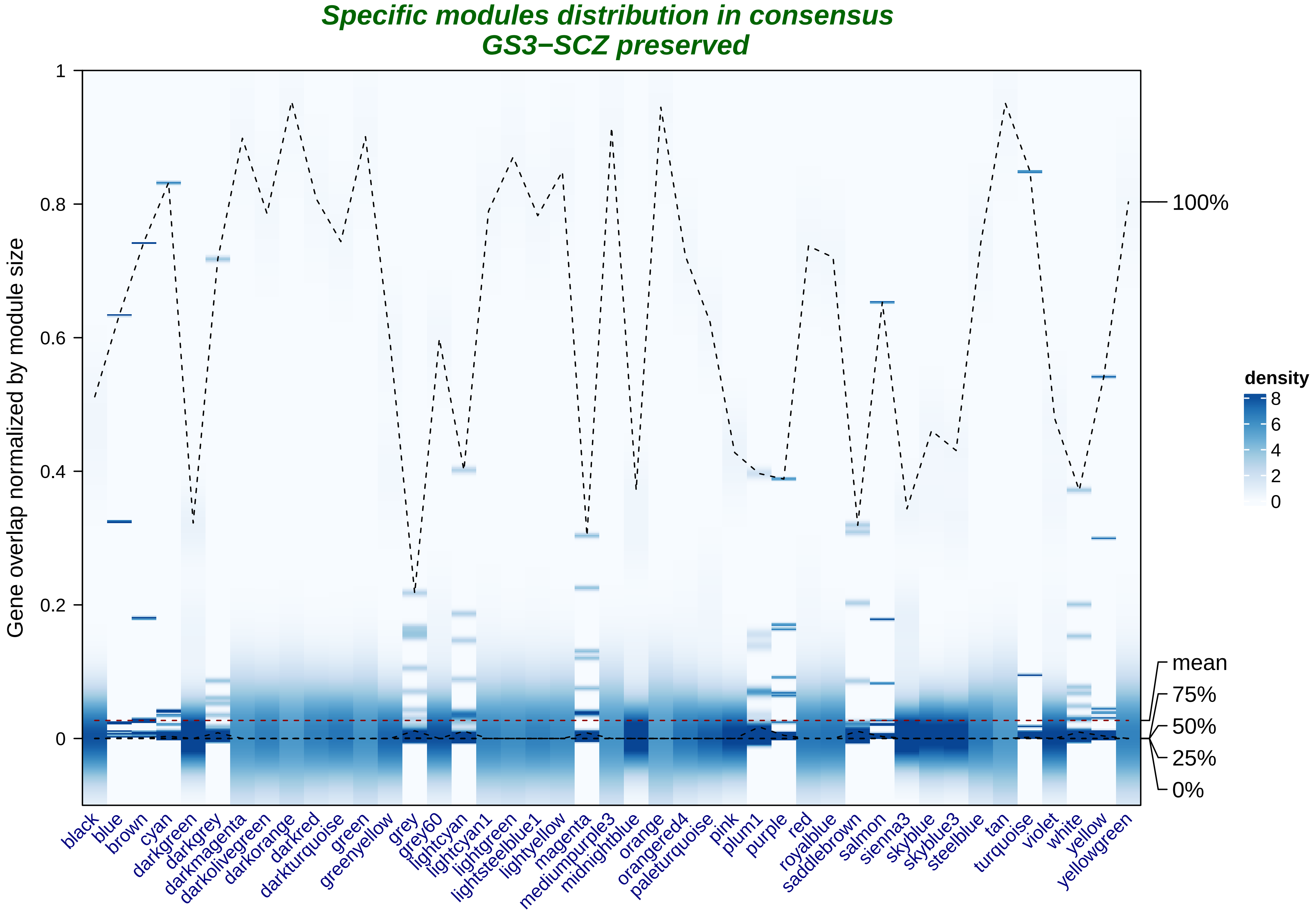

Specific modules distribution in consensus
GS3−SCZ preserved
Gene overlap normalized by module size
1
0.8
0.6
0.4
0.2
0
100%
mean
75%
50%
25%
0%
density
8
6
4
2
0
black
blue
brown
cyan
darkgreen
darkgrey
darkmagenta
darkolivegreen
darkorange
darkred
darkturquoise
green
greenyellow
grey
grey60
lightcyan
lightcyan1
lightgreen
lightsteelblue1
lightyellow
magenta
mediumpurple3
midnightblue
orange
orangered4
paleturquoise
pink
plum1
purple
red
royalblue
saddlebrown
salmon
sienna3
skyblue
skyblue3
steelblue
tan
turquoise
violet
white
yellow
yellowgreen

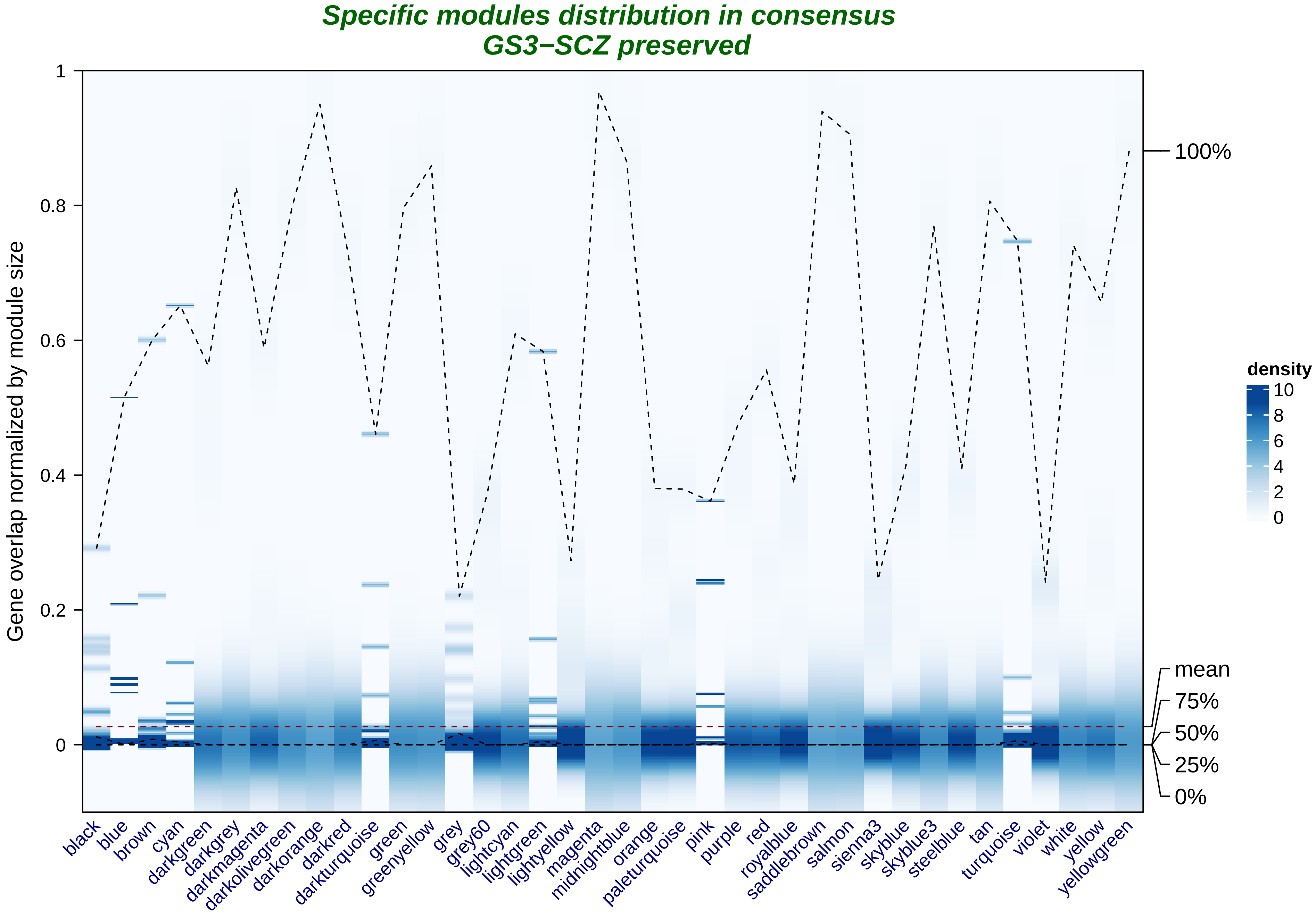

Specific modules distribution in consensus
GS3−SCZ preserved
Gene overlap normalized by module size
1
0.8
0.6
0.4
0.2
0
100%
mean
75%
50%
25%
0%
density
10
8
6
4
2
0
black
blue
brown
cyan
darkgreen
darkgrey
darkmagenta
darkolivegreen
darkorange
darkred
darkturquoise
green
greenyellow
grey
grey60
lightcyan
lightgreen
lightyellow
magenta
midnightblue
orange
paleturquoise
pink
purple
red
royalblue
saddlebrown
salmon
sienna3
skyblue
skyblue3
steelblue
tan
turquoise
violet
white
yellow
yellowgreen

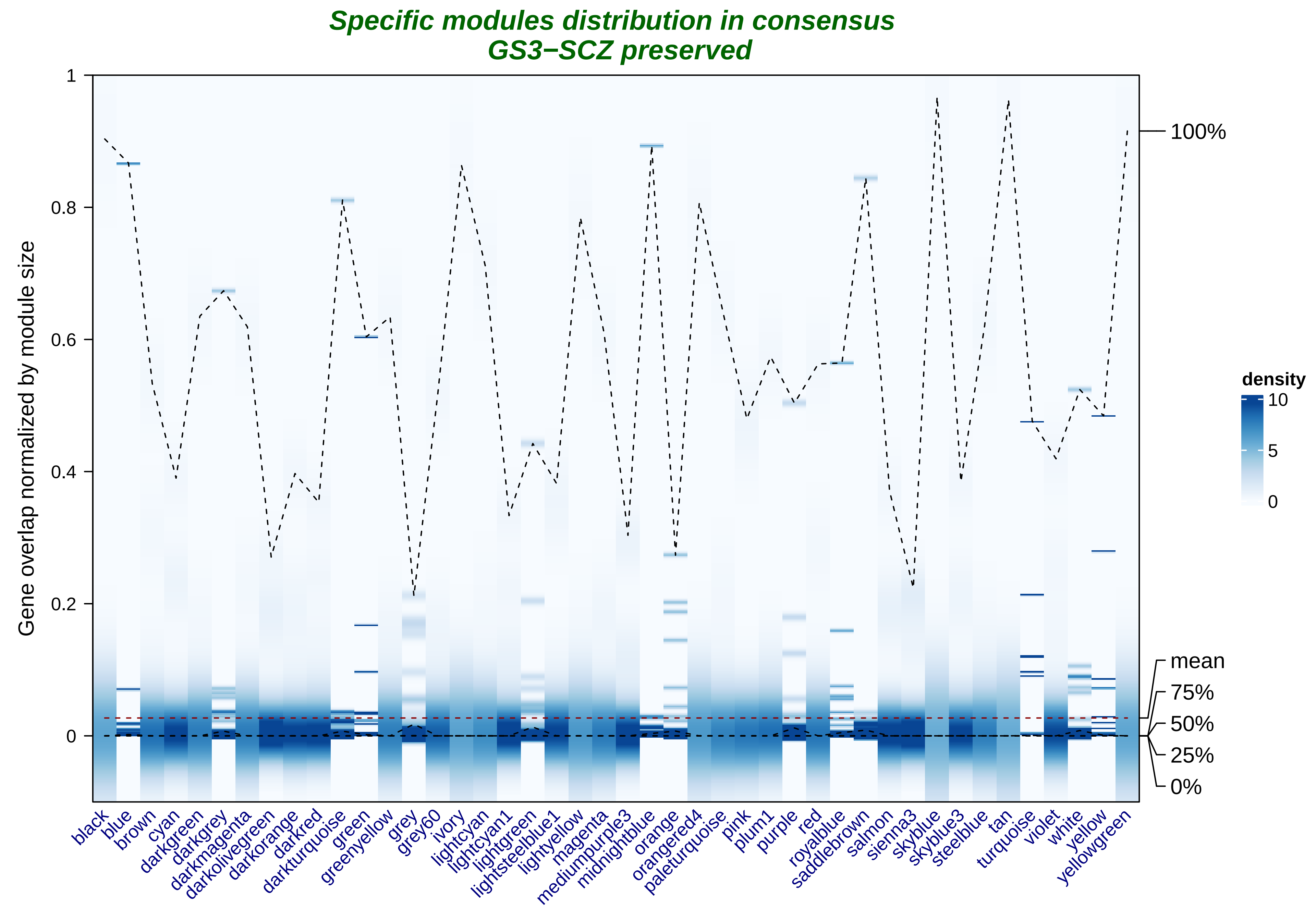

Specific modules distribution in consensus
GS3−SCZ preserved
Gene overlap normalized by module size
1
0.8
0.6
0.4
0.2
0
100%
mean
75%
50%
25%
0%
density
10
5
0
black
blue
brown
cyan
darkgreen
darkgrey
darkmagenta
darkolivegreen
darkorange
darkred
darkturquoise
green
greenyellow
grey
grey60
ivory
lightcyan
lightcyan1
lightgreen
lightsteelblue1
lightyellow
magenta
mediumpurple3
midnightblue
orange
orangered4
paleturquoise
pink
plum1
purple
red
royalblue
saddlebrown
salmon
sienna3
skyblue
skyblue3
steelblue
tan
turquoise
violet
white
yellow
yellowgreen

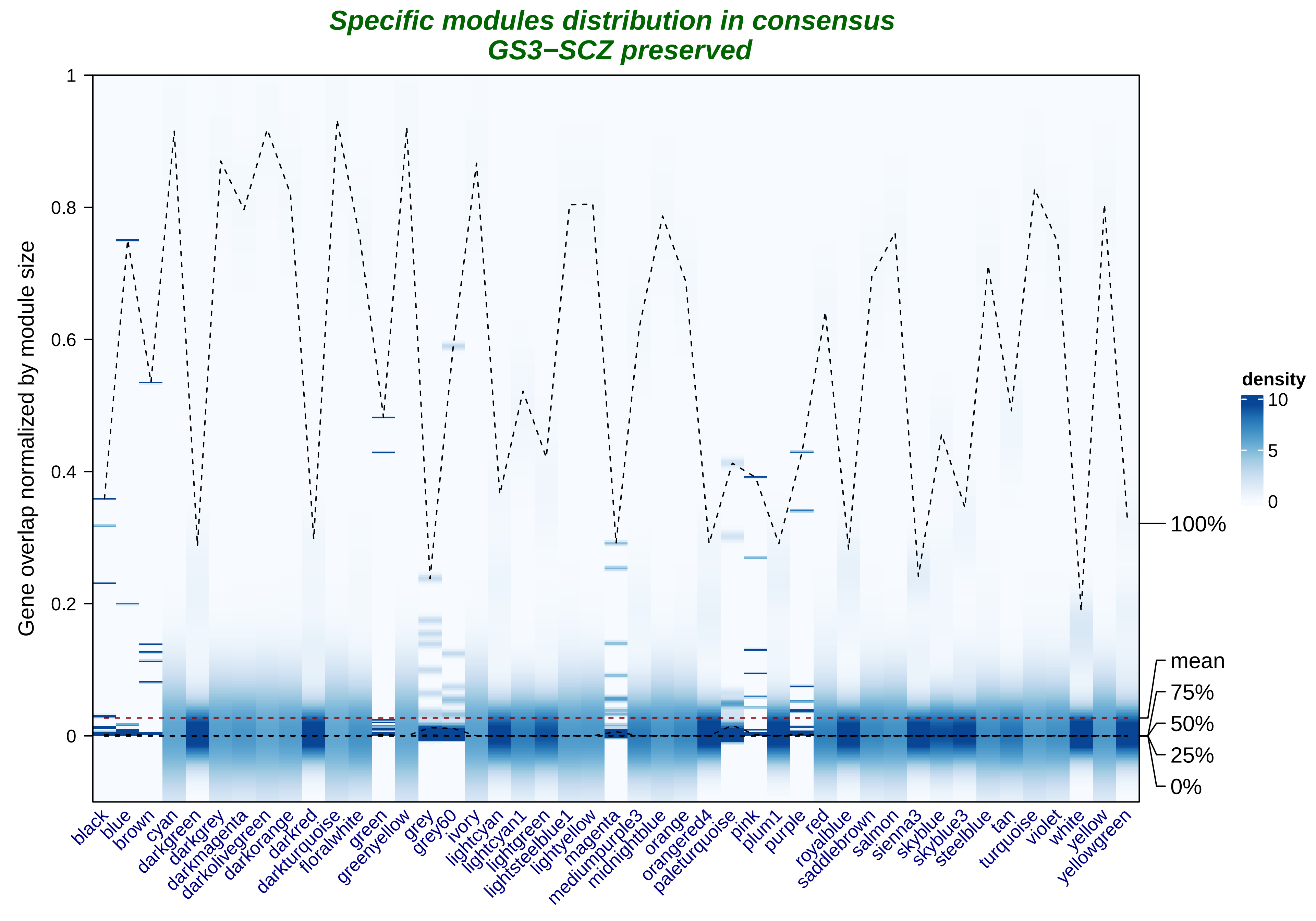
Specific modules distribution in consensus
GS3−SCZ preserved
Gene overlap normalized by module size
1
0.8
0.6
0.4
0.2
0
density
10
5
0
100%
mean
75%
50%
25%
0%
black
blue
brown
cyan
darkgreen
darkgrey
darkmagenta
darkolivegreen
darkorange
darkred
darkturquoise
floralwhite
green
greenyellow
grey
grey60
ivory
lightcyan
lightcyan1
lightgreen
lightsteelblue1
lightyellow
magenta
mediumpurple3
midnightblue
orange
orangered4
paleturquoise
pink
plum1
purple
red
royalblue
saddlebrown
salmon
sienna3
skyblue
skyblue3
steelblue
tan
turquoise
violet
white
yellow
yellowgreen

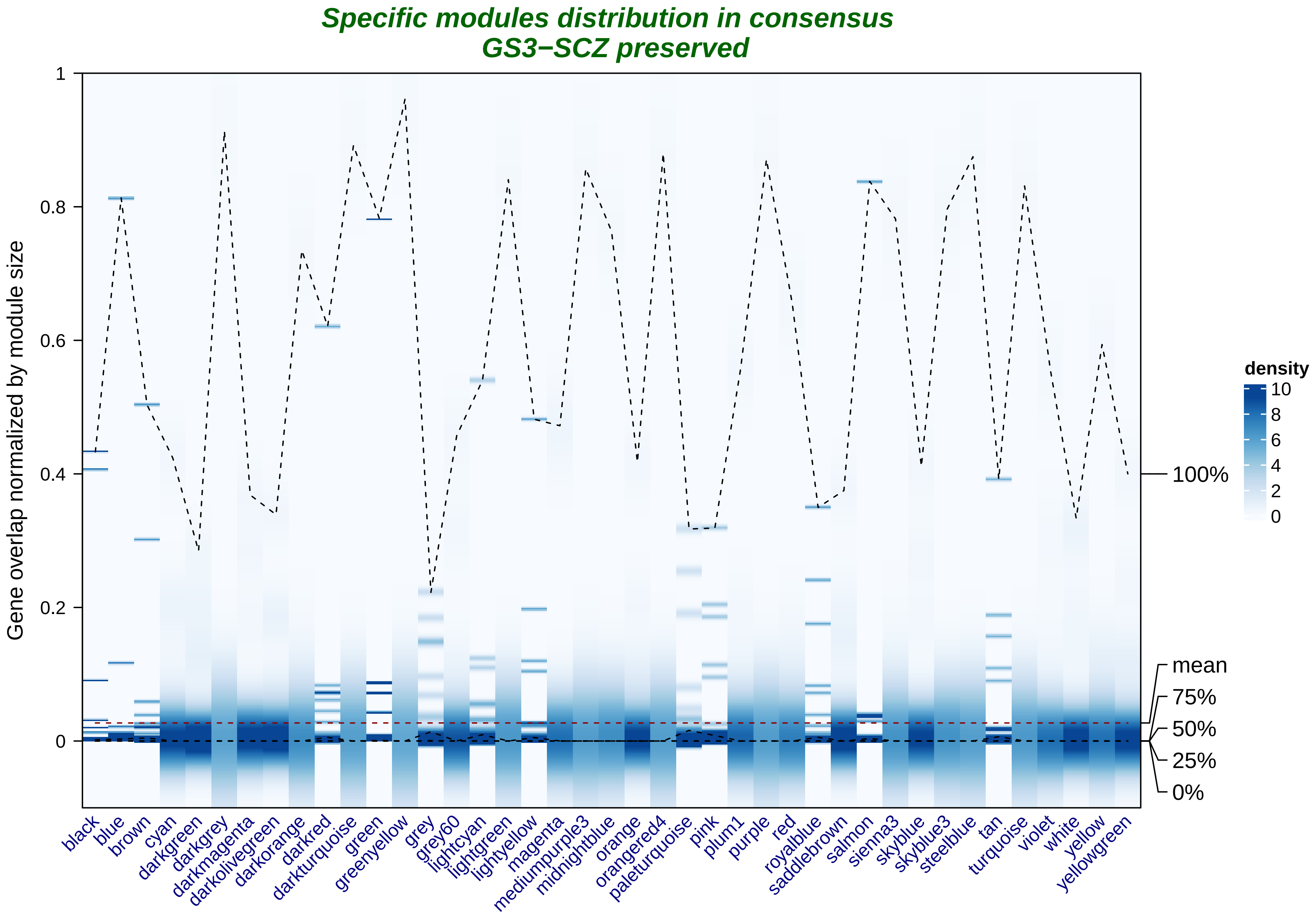

Specific modules distribution in consensus
GS3−SCZ preserved
Gene overlap normalized by module size
1
0.8
0.6
0.4
0.2
0
100%
mean
75%
50%
25%
0%
density
10
8
6
4
2
0
black
blue
brown
cyan
darkgreen
darkgrey
darkmagenta
darkolivegreen
darkorange
darkred
darkturquoise
green
greenyellow
grey
grey60
lightcyan
lightgreen
lightyellow
magenta
mediumpurple3
midnightblue
orange
orangered4
paleturquoise
pink
plum1
purple
red
royalblue
saddlebrown
salmon
sienna3
skyblue
skyblue3
steelblue
tan
turquoise
violet
white
yellow
yellowgreen

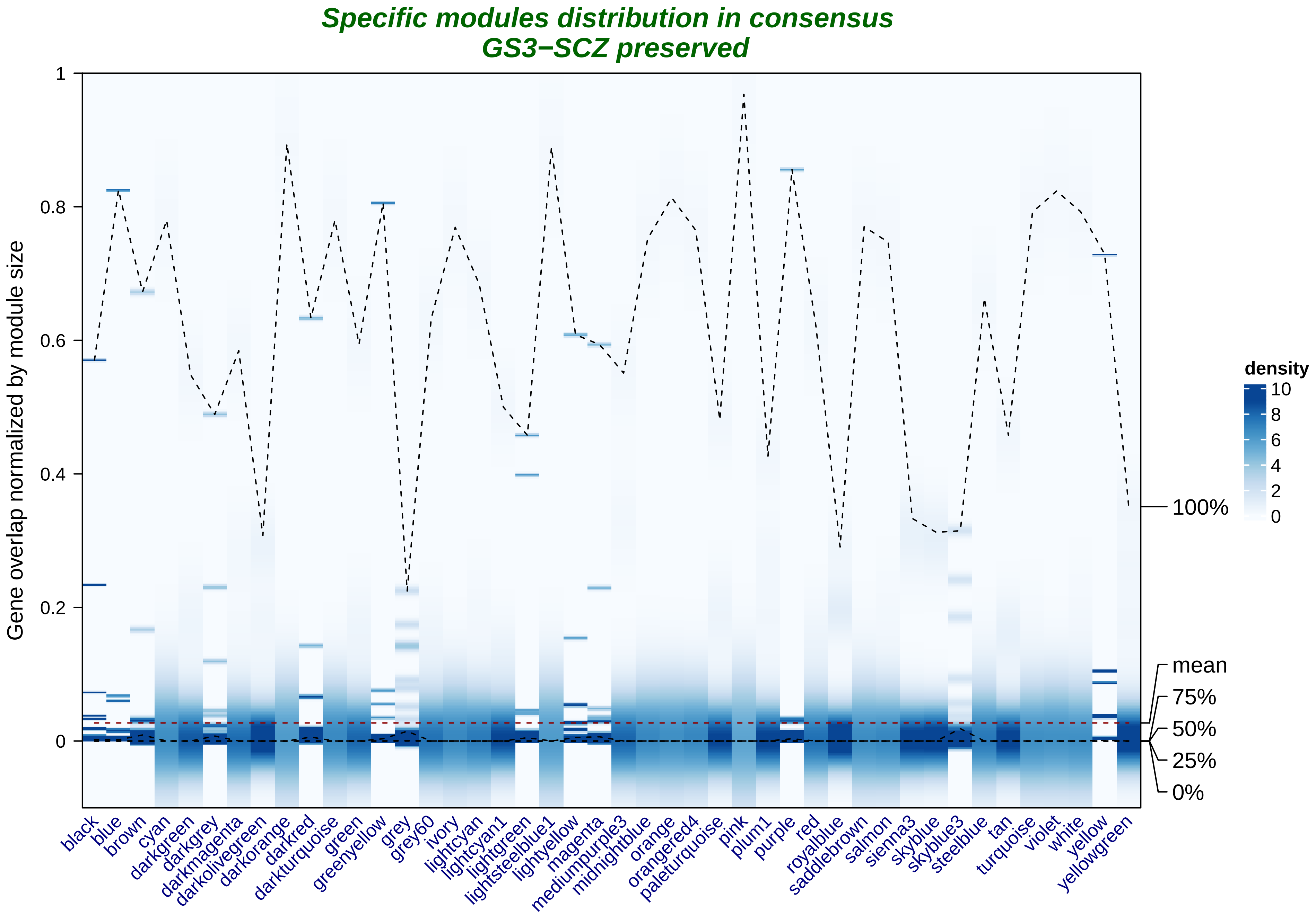

Specific modules distribution in consensus
GS3−SCZ preserved
Gene overlap normalized by module size
1
0.8
0.6
0.4
0.2
0
100%
mean
75%
50%
25%
0%
density
10
8
6
4
2
0
black
blue
brown
cyan
darkgreen
darkgrey
darkmagenta
darkolivegreen
darkorange
darkred
darkturquoise
green
greenyellow
grey
grey60
ivory
lightcyan
lightcyan1
lightgreen
lightsteelblue1
lightyellow
magenta
mediumpurple3
midnightblue
orange
orangered4
paleturquoise
pink
plum1
purple
red
royalblue
saddlebrown
salmon
sienna3
skyblue
skyblue3
steelblue
tan
turquoise
violet
white
yellow
yellowgreen

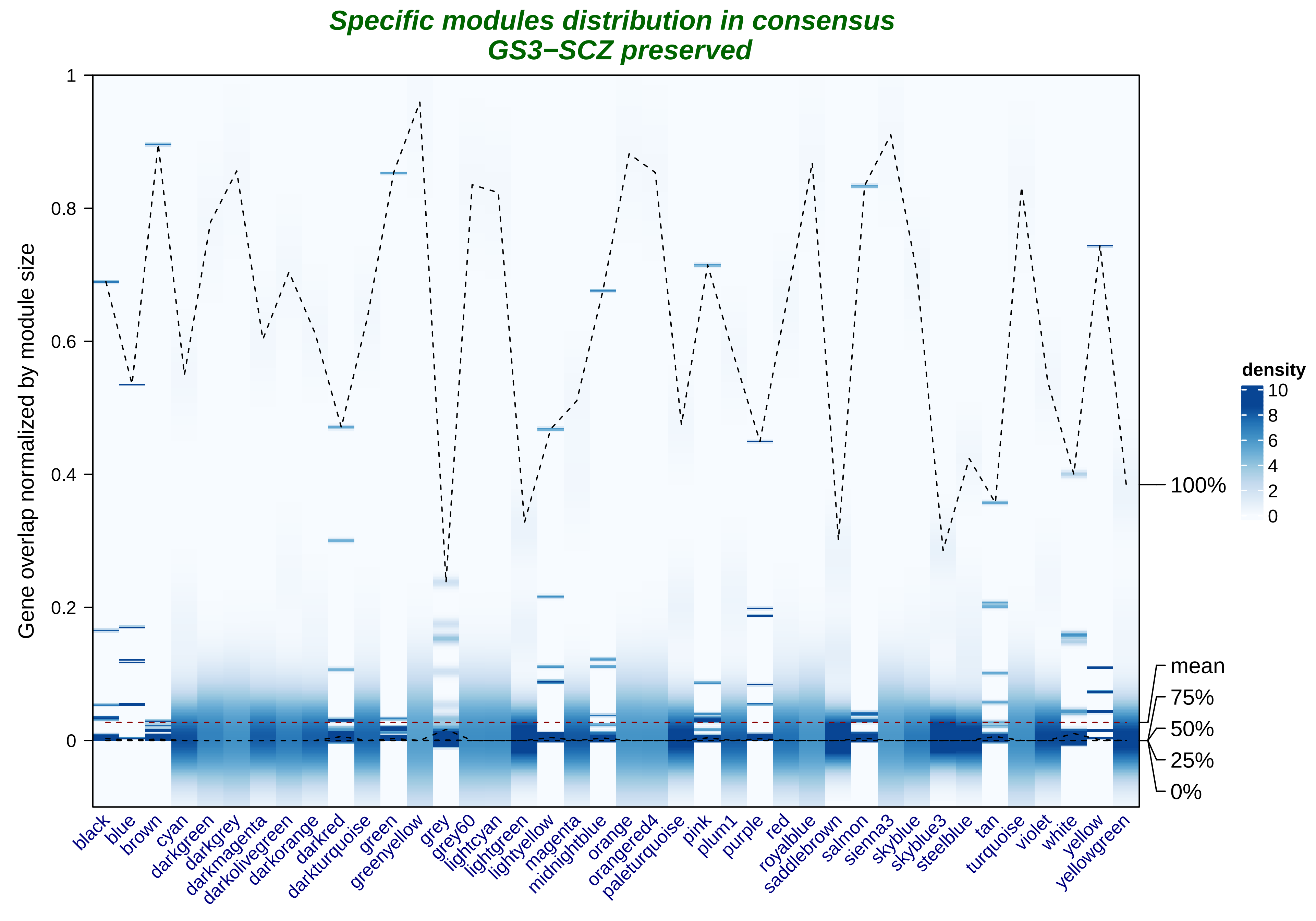

Specific modules distribution in consensus
GS3−SCZ preserved
Gene overlap normalized by module size
1
0.8
0.6
0.4
0.2
0
density
10
8
6
4
2
0
100%
mean
75%
50%
25%
0%
black
blue
brown
cyan
darkgreen
darkgrey
darkmagenta
darkolivegreen
darkorange
darkred
darkturquoise
green
greenyellow
grey
grey60
lightcyan
lightgreen
lightyellow
magenta
midnightblue
orange
orangered4
paleturquoise
pink
plum1
purple
red
royalblue
saddlebrown
salmon
sienna3
skyblue
skyblue3
steelblue
tan
turquoise
violet
white
yellow
yellowgreen

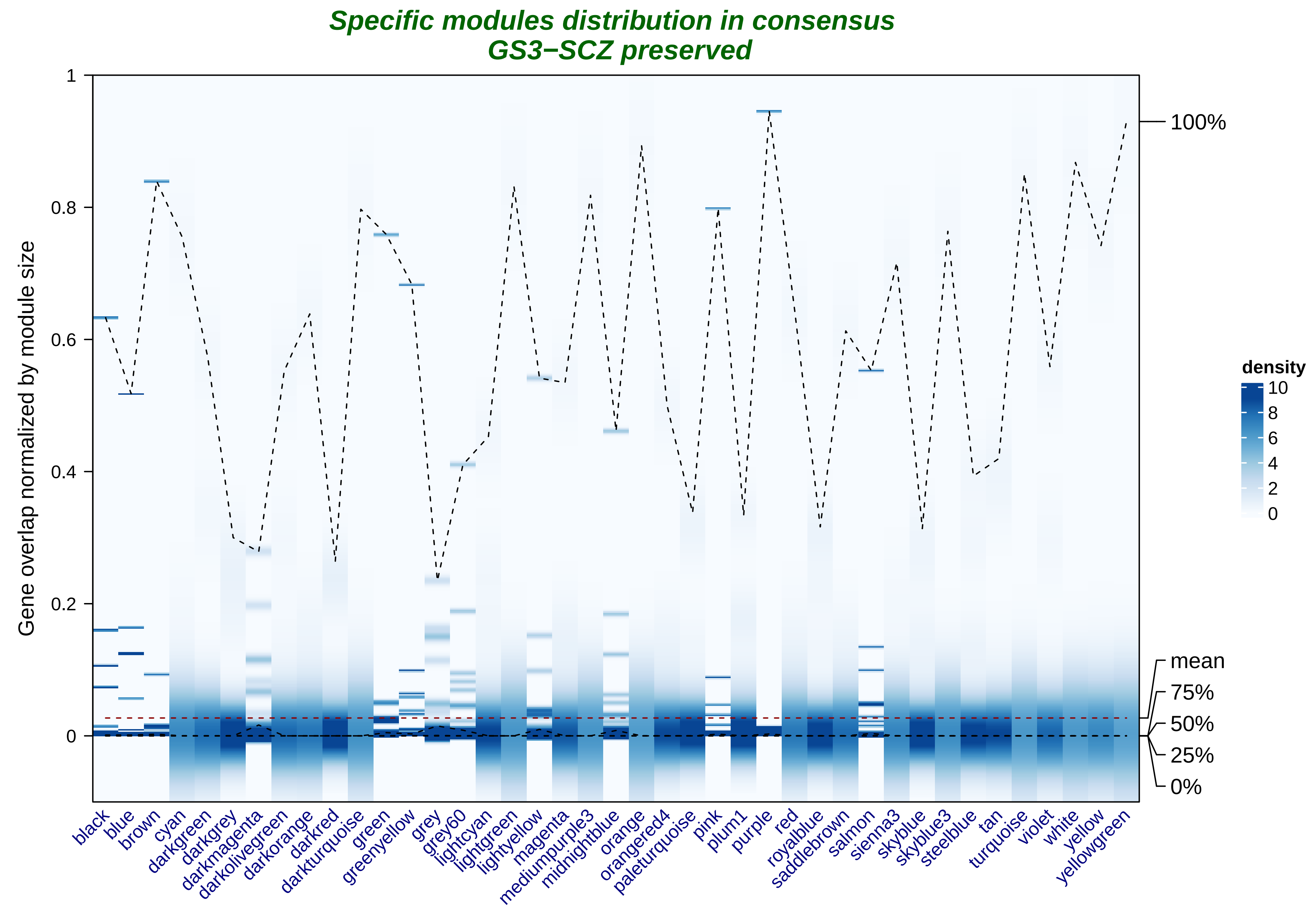

Specific modules distribution in consensus
GS3−SCZ preserved
Gene overlap normalized by module size
1
0.8
0.6
0.4
0.2
0
100%
mean
75%
50%
25%
0%
density
10
8
6
4
2
0
black
blue
brown
cyan
darkgreen
darkgrey
darkmagenta
darkolivegreen
darkorange
darkred
darkturquoise
green
greenyellow
grey
grey60
lightcyan
lightgreen
lightyellow
magenta
mediumpurple3
midnightblue
orange
orangered4
paleturquoise
pink
plum1
purple
red
royalblue
saddlebrown
salmon
sienna3
skyblue
skyblue3
steelblue
tan
turquoise
violet
white
yellow
yellowgreen

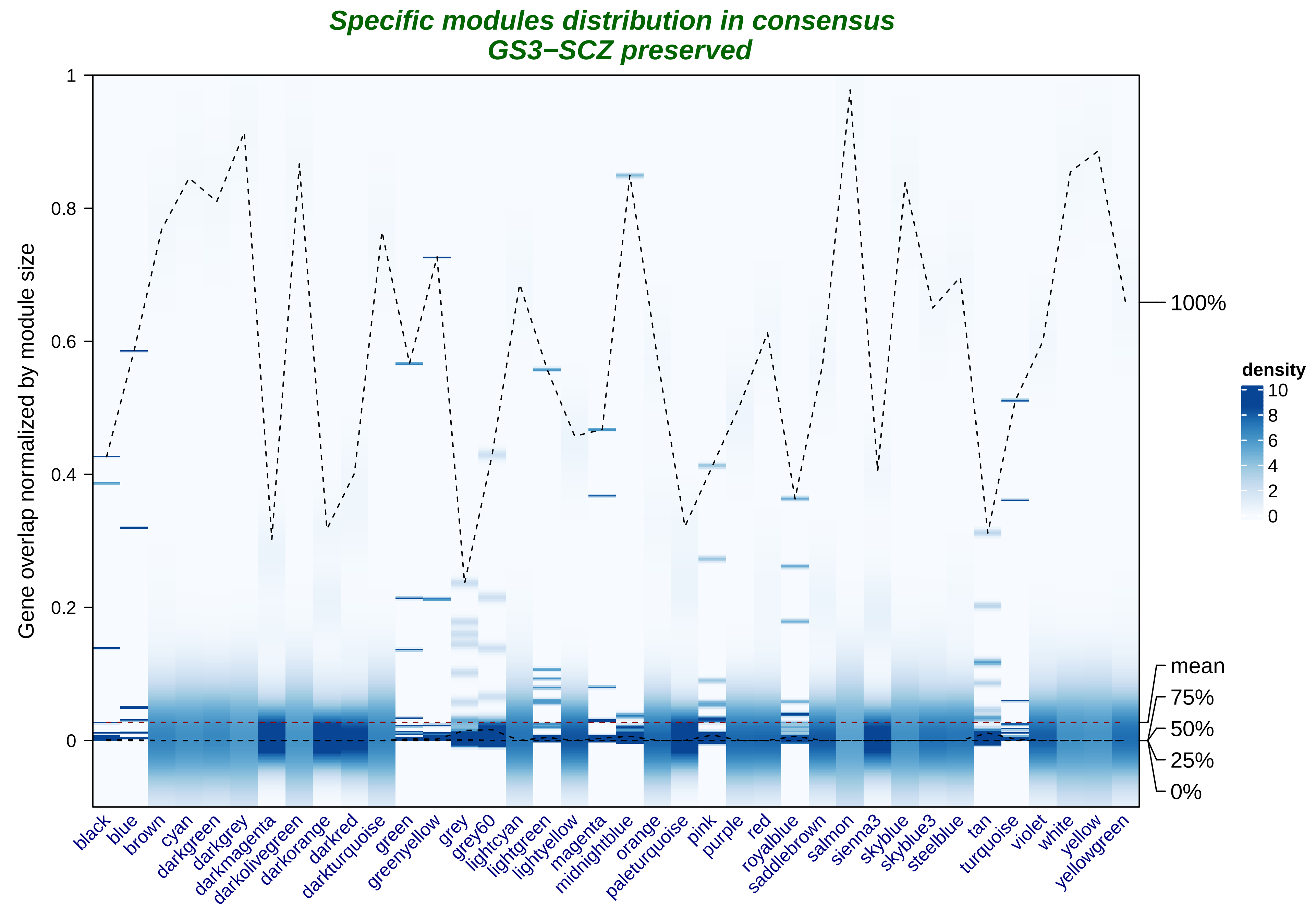

Specific modules distribution in consensus
GS3−SCZ preserved
Gene overlap normalized by module size
1
0.8
0.6
0.4
0.2
0
100%
mean
75%
50%
25%
0%
density
10
8
6
4
2
0
black
blue
brown
cyan
darkgreen
darkgrey
darkmagenta
darkolivegreen
darkorange
darkred
darkturquoise
green
greenyellow
grey
grey60
lightcyan
lightgreen
lightyellow
magenta
midnightblue
orange
paleturquoise
pink
purple
red
royalblue
saddlebrown
salmon
sienna3
skyblue
skyblue3
steelblue
tan
turquoise
violet
white
yellow
yellowgreen

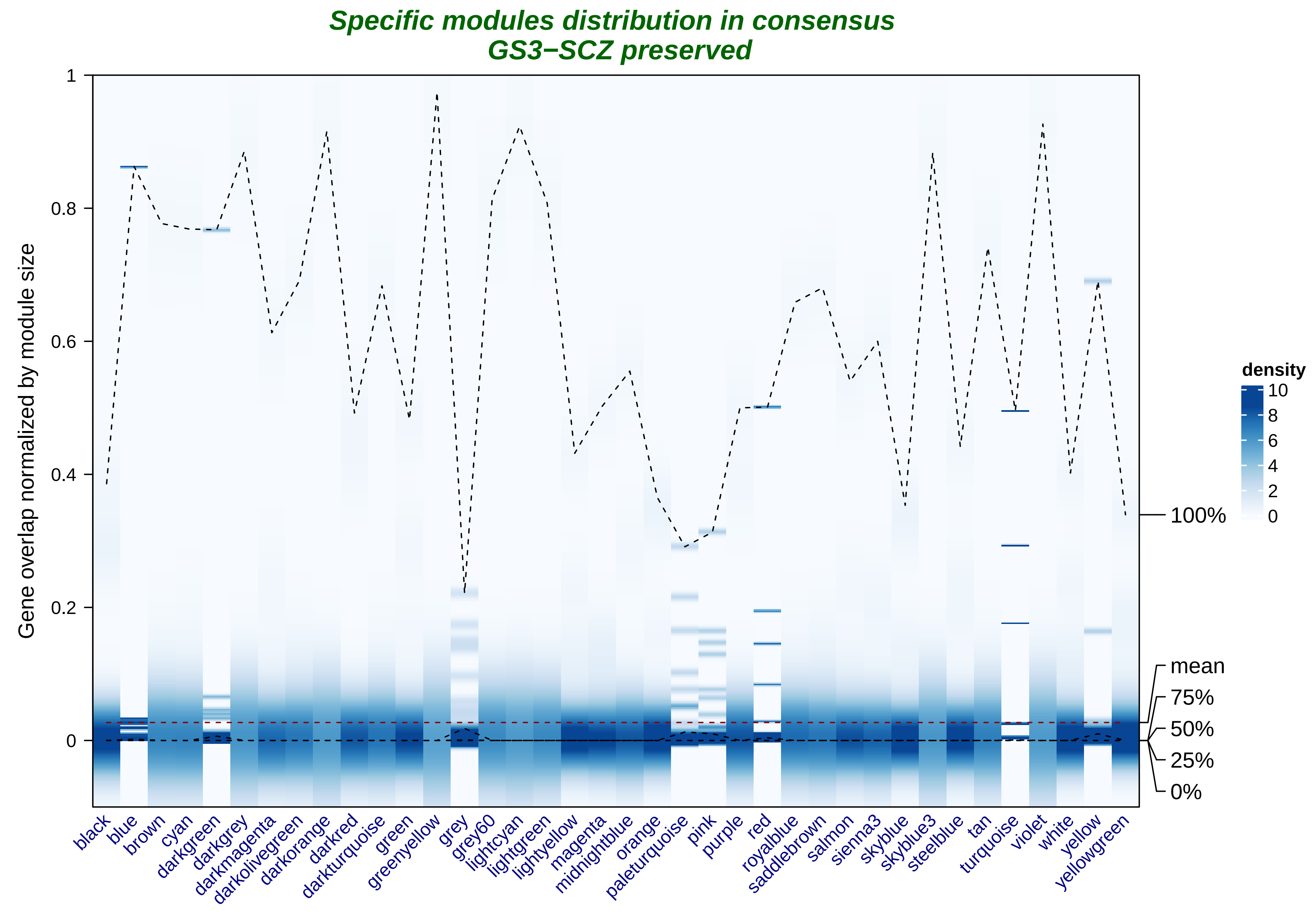

Specific modules distribution in consensus
GS3−SCZ preserved
Gene overlap normalized by module size
1
0.8
0.6
0.4
0.2
0
density
10
8
6
4
2
0
100%
mean
75%
50%
25%
0%
black
blue
brown
cyan
darkgreen
darkgrey
darkmagenta
darkolivegreen
darkorange
darkred
darkturquoise
green
greenyellow
grey
grey60
lightcyan
lightgreen
lightyellow
magenta
midnightblue
orange
paleturquoise
pink
purple
red
royalblue
saddlebrown
salmon
sienna3
skyblue
skyblue3
steelblue
tan
turquoise
violet
white
yellow
yellowgreen

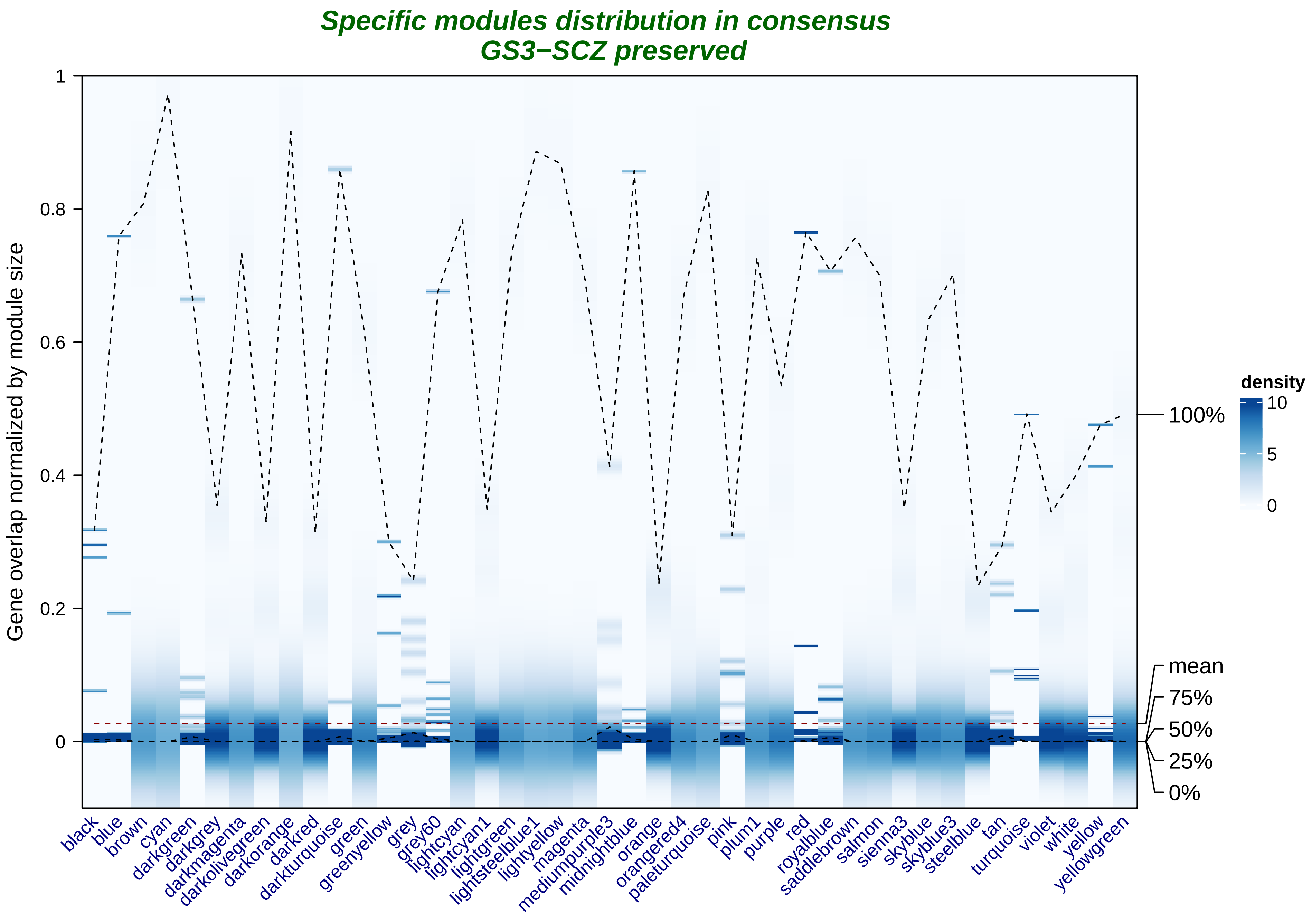

Specific modules distribution in consensus
GS3−SCZ preserved
Gene overlap normalized by module size
1
0.8
0.6
0.4
0.2
0
density
10
5
0
100%
mean
75%
50%
25%
0%
black
blue
brown
cyan
darkgreen
darkgrey
darkmagenta
darkolivegreen
darkorange
darkred
darkturquoise
green
greenyellow
grey
grey60
lightcyan
lightcyan1
lightgreen
lightsteelblue1
lightyellow
magenta
mediumpurple3
midnightblue
orange
orangered4
paleturquoise
pink
plum1
purple
red
royalblue
saddlebrown
salmon
sienna3
skyblue
skyblue3
steelblue
tan
turquoise
violet
white
yellow
yellowgreen

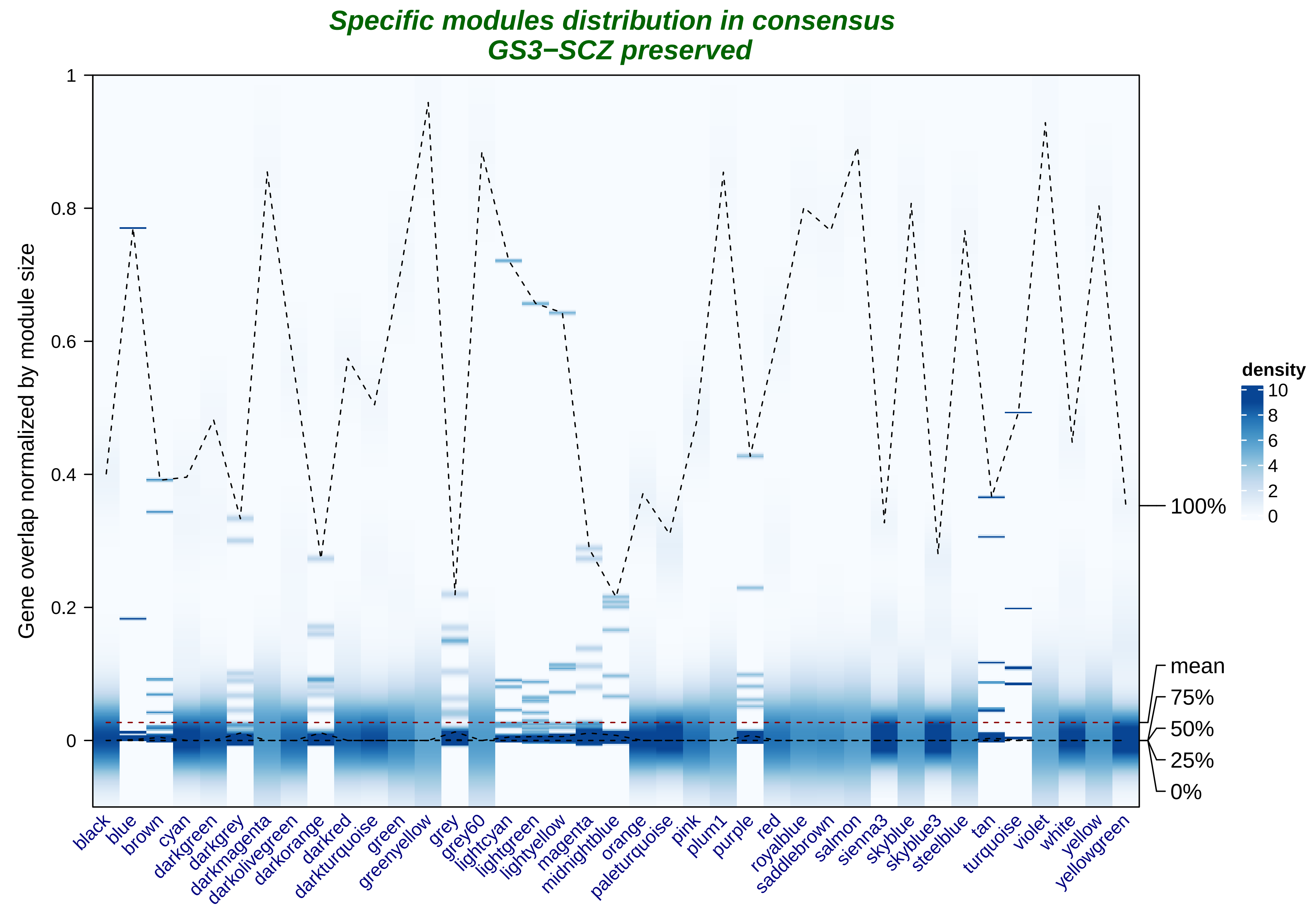

Specific modules distribution in consensus
GS3−SCZ preserved
Gene overlap normalized by module size
1
0.8
0.6
0.4
0.2
0
black
blue
brown
cyan
darkgreen
darkgrey
darkmagenta
darkolivegreen
darkorange
darkred
darkturquoise
green
greenyellow
grey
grey60
lightcyan
lightgreen
lightyellow
magenta
midnightblue
orange
paleturquoise
pink
plum1
purple
red
royalblue
saddlebrown
salmon
sienna3
skyblue
skyblue3
steelblue
tan
turquoise
violet
white
yellow
yellowgreen
100%
mean
75%
50%
25%
0%
density
10
8
6
4
2
0

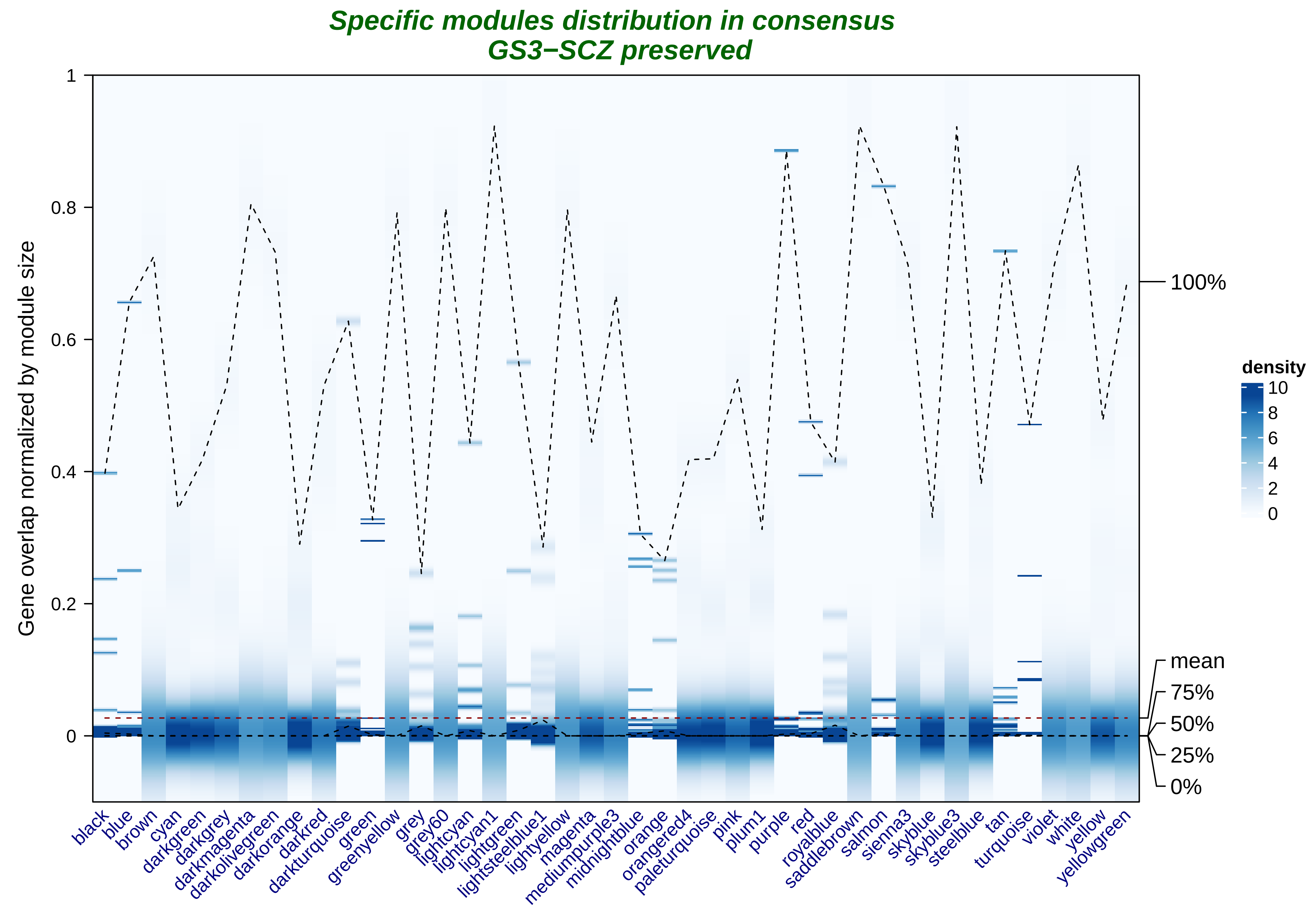

Specific modules distribution in consensus
GS3−SCZ preserved
Gene overlap normalized by module size
1
0.8
0.6
0.4
0.2
0
100%
mean
75%
50%
25%
0%
density
10
8
6
4
2
0
black
blue
brown
cyan
darkgreen
darkgrey
darkmagenta
darkolivegreen
darkorange
darkred
darkturquoise
green
greenyellow
grey
grey60
lightcyan
lightcyan1
lightgreen
lightsteelblue1
lightyellow
magenta
mediumpurple3
midnightblue
orange
orangered4
paleturquoise
pink
plum1
purple
red
royalblue
saddlebrown
salmon
sienna3
skyblue
skyblue3
steelblue
tan
turquoise
violet
white
yellow
yellowgreen

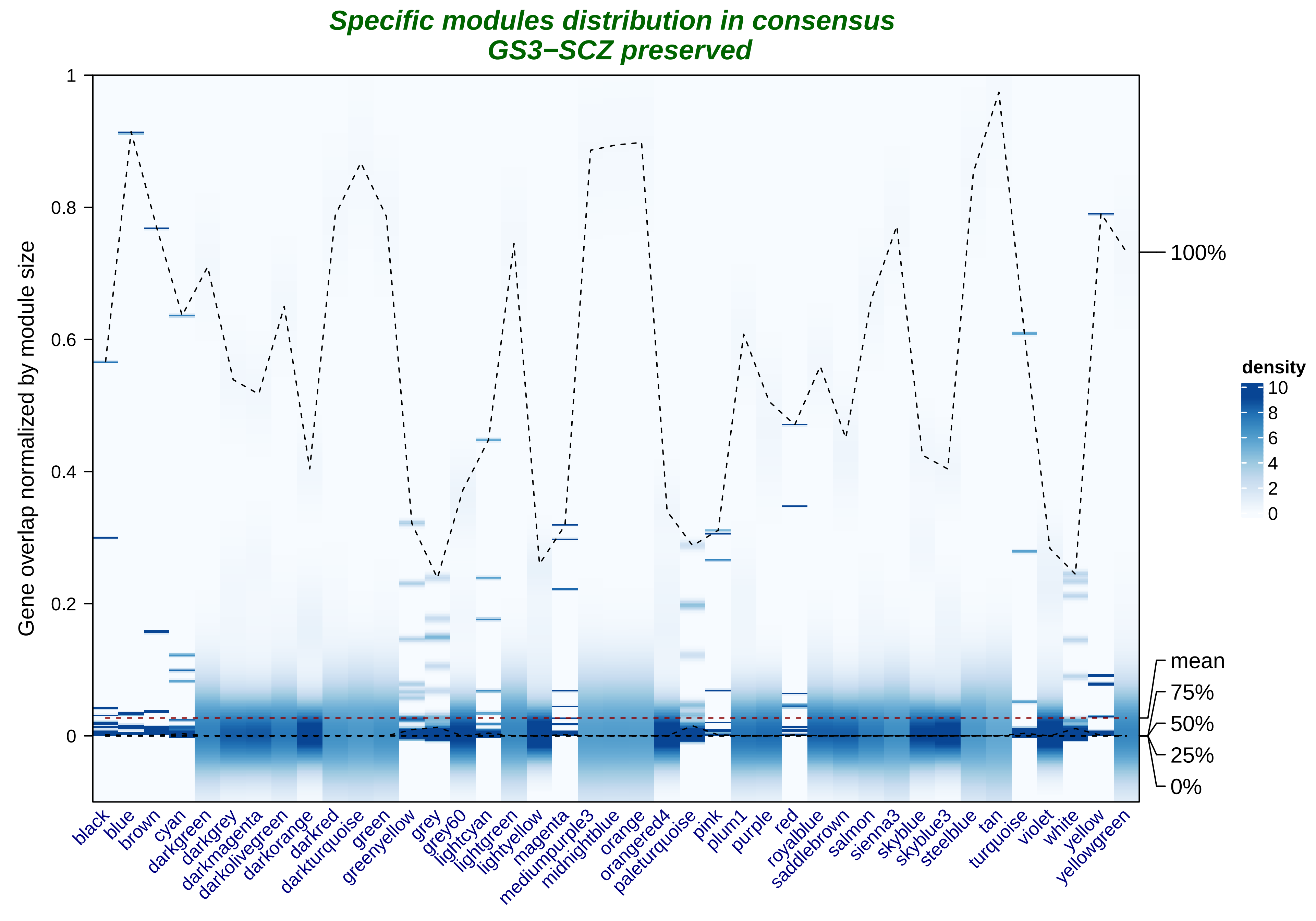

Specific modules distribution in consensus
GS3−SCZ preserved
Gene overlap normalized by module size
1
0.8
0.6
0.4
0.2
0
100%
mean
75%
50%
25%
0%
density
10
8
6
4
2
0
black
blue
brown
cyan
darkgreen
darkgrey
darkmagenta
darkolivegreen
darkorange
darkred
darkturquoise
green
greenyellow
grey
grey60
lightcyan
lightgreen
lightyellow
magenta
mediumpurple3
midnightblue
orange
orangered4
paleturquoise
pink
plum1
purple
red
royalblue
saddlebrown
salmon
sienna3
skyblue
skyblue3
steelblue
tan
turquoise
violet
white
yellow
yellowgreen

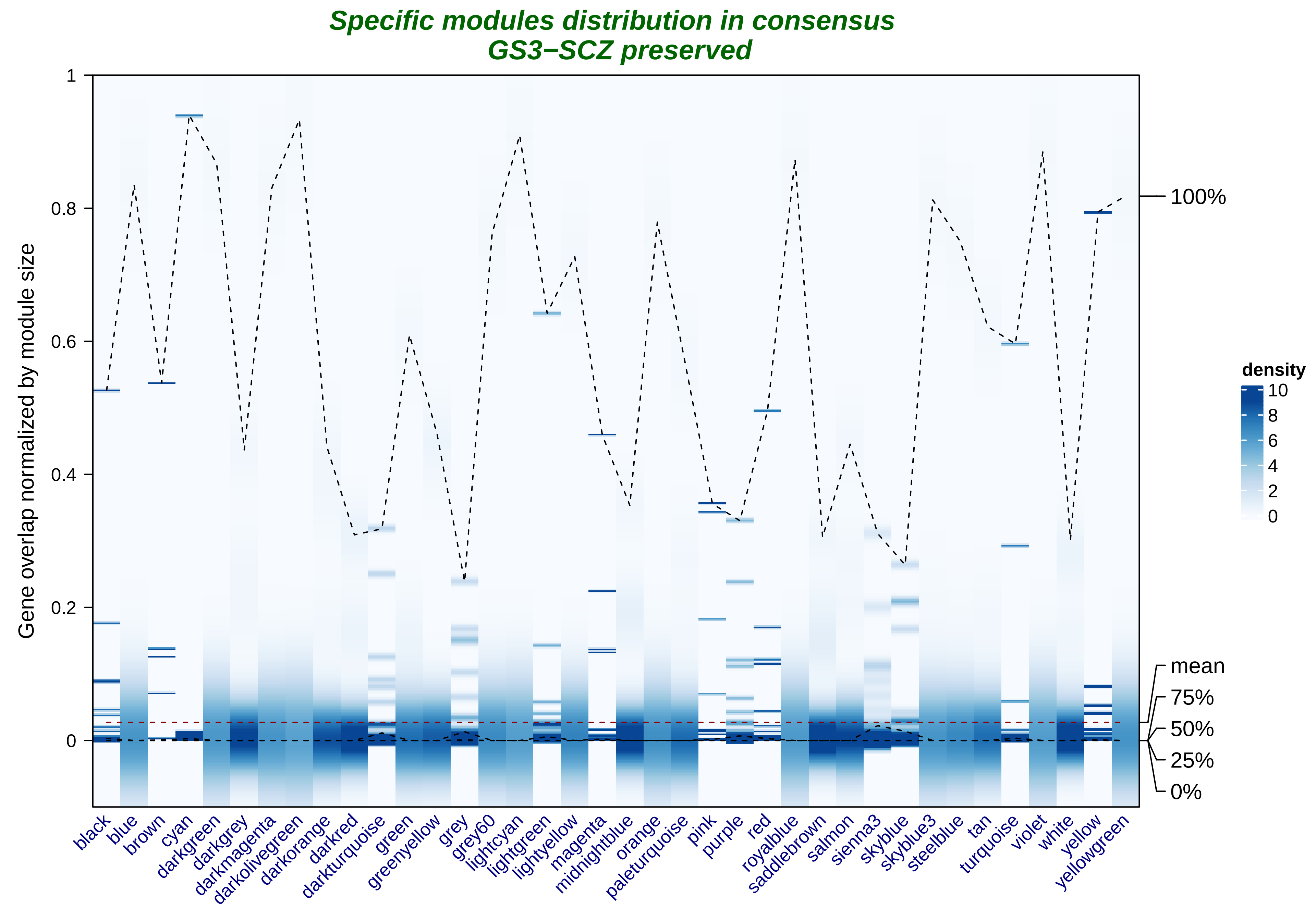

Specific modules distribution in consensus
GS3−SCZ preserved
Gene overlap normalized by module size
1
0.8
0.6
0.4
0.2
0
100%
mean
75%
50%
25%
0%
density
10
8
6
4
2
0
black
blue
brown
cyan
darkgreen
darkgrey
darkmagenta
darkolivegreen
darkorange
darkred
darkturquoise
green
greenyellow
grey
grey60
lightcyan
lightgreen
lightyellow
magenta
midnightblue
orange
paleturquoise
pink
purple
red
royalblue
saddlebrown
salmon
sienna3
skyblue
skyblue3
steelblue
tan
turquoise
violet
white
yellow
yellowgreen

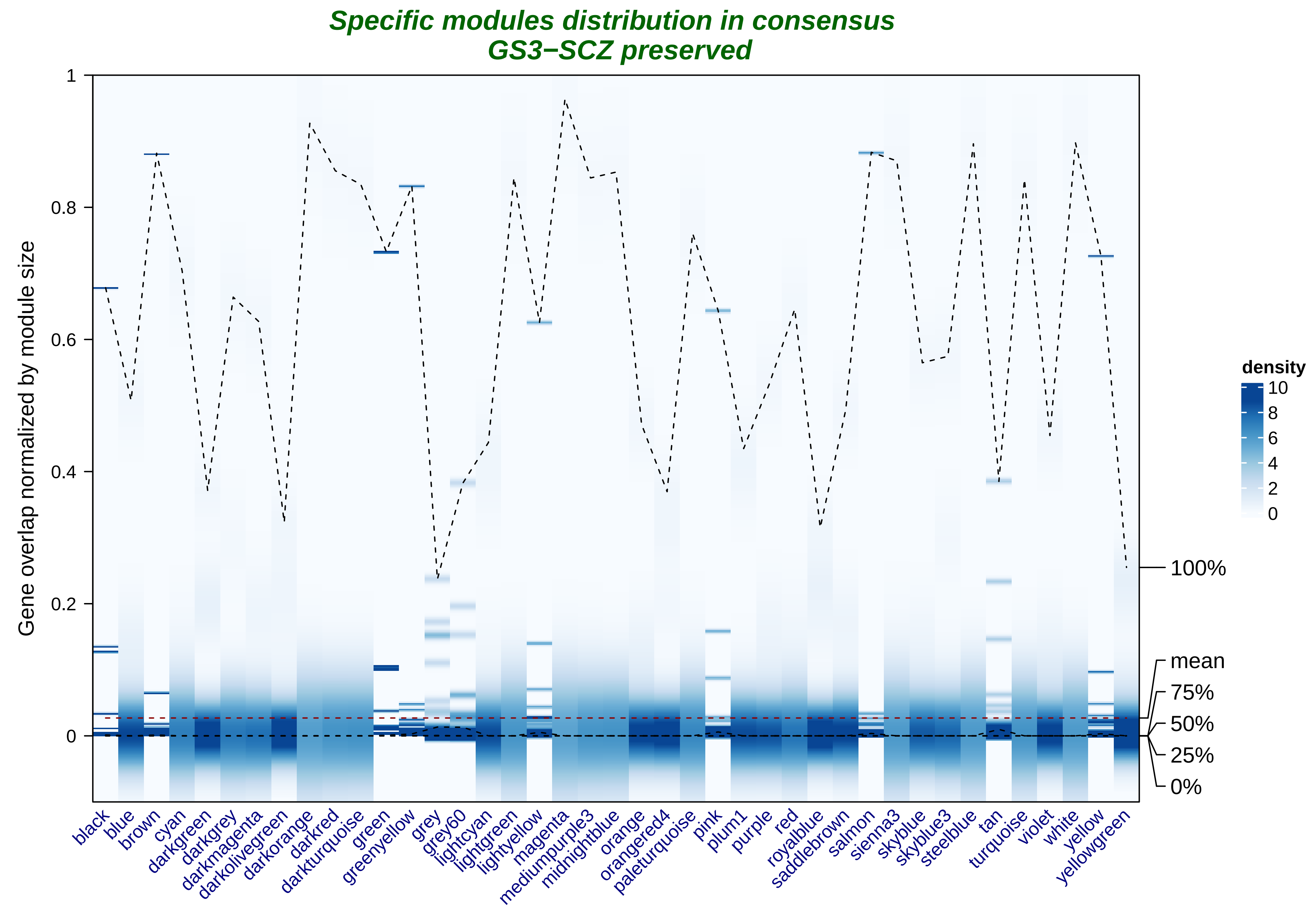

Specific modules distribution in consensus
GS3−SCZ preserved
Gene overlap normalized by module size
1
0.8
0.6
0.4
0.2
0
100%
mean
75%
50%
25%
0%
density
10
8
6
4
2
0
black
blue
brown
cyan
darkgreen
darkgrey
darkmagenta
darkolivegreen
darkorange
darkred
darkturquoise
green
greenyellow
grey
grey60
lightcyan
lightgreen
lightyellow
magenta
mediumpurple3
midnightblue
orange
orangered4
paleturquoise
pink
plum1
purple
red
royalblue
saddlebrown
salmon
sienna3
skyblue
skyblue3
steelblue
tan
turquoise
violet
white
yellow
yellowgreen

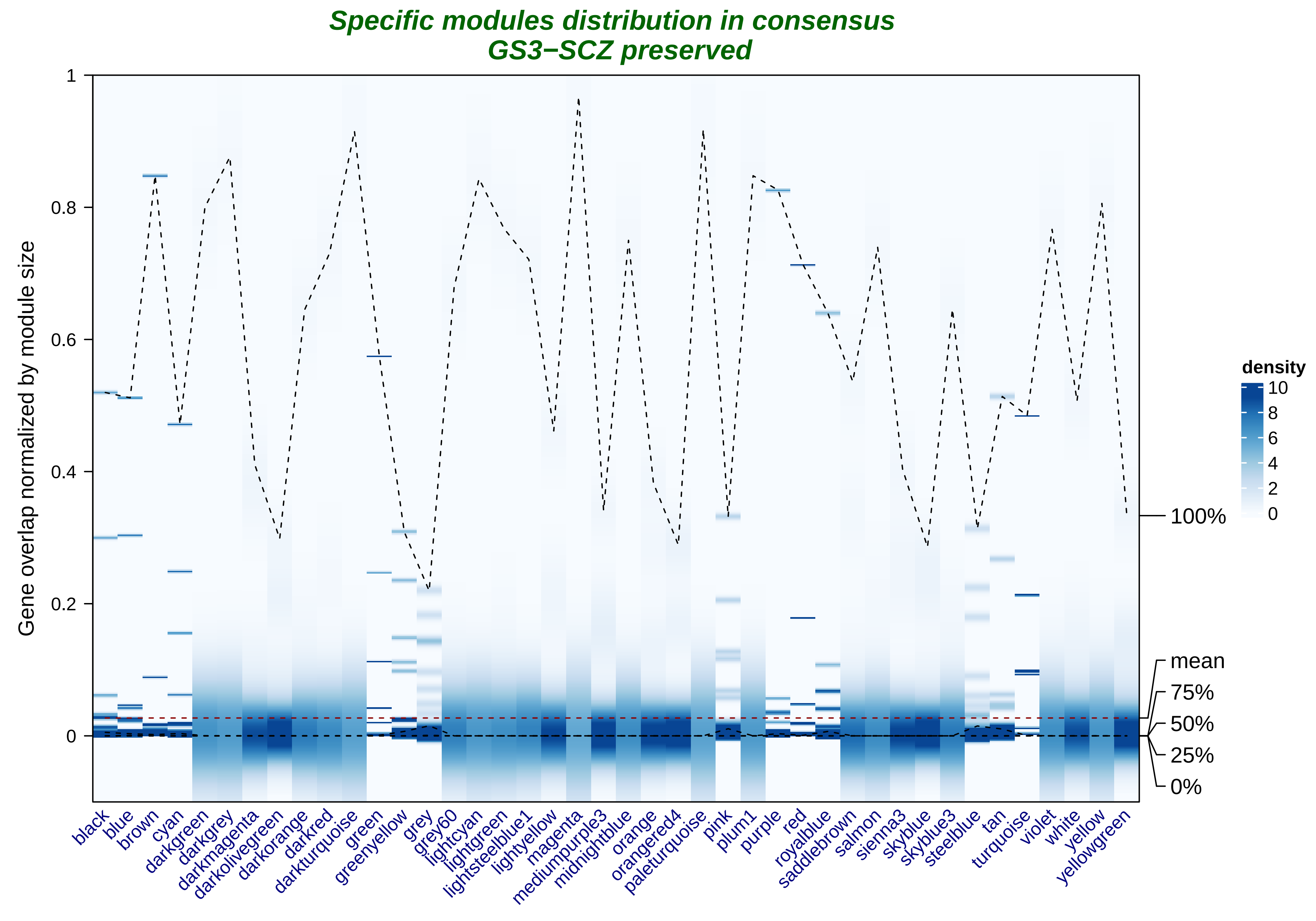

Specific modules distribution in consensus
GS3−SCZ preserved
Gene overlap normalized by module size
1
0.8
0.6
0.4
0.2
0
density
10
8
6
4
2
0
100%
mean
75%
50%
25%
0%
black
blue
brown
cyan
darkgreen
darkgrey
darkmagenta
darkolivegreen
darkorange
darkred
darkturquoise
green
greenyellow
grey
grey60
lightcyan
lightgreen
lightsteelblue1
lightyellow
magenta
mediumpurple3
midnightblue
orange
orangered4
paleturquoise
pink
plum1
purple
red
royalblue
saddlebrown
salmon
sienna3
skyblue
skyblue3
steelblue
tan
turquoise
violet
white
yellow
yellowgreen

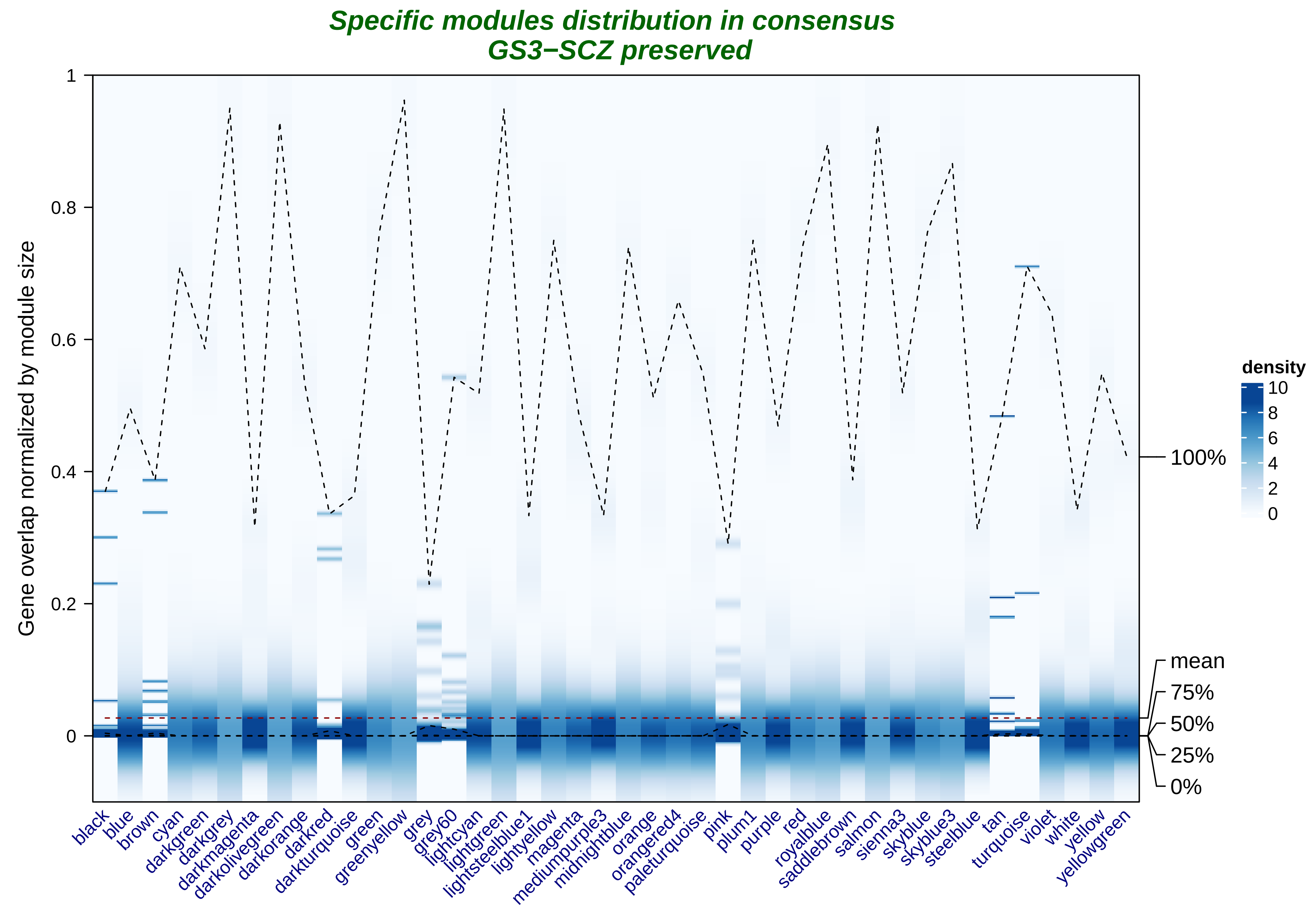
Specific modules distribution in consensus
GS3−SCZ preserved
Gene overlap normalized by module size
1
0.8
0.6
0.4
0.2
0
density
10
8
6
4
2
0
100%
mean
75%
50%
25%
0%
black
blue
brown
cyan
darkgreen
darkgrey
darkmagenta
darkolivegreen
darkorange
darkred
darkturquoise
green
greenyellow
grey
grey60
lightcyan
lightgreen
lightsteelblue1
lightyellow
magenta
mediumpurple3
midnightblue
orange
orangered4
paleturquoise
pink
plum1
purple
red
royalblue
saddlebrown
salmon
sienna3
skyblue
skyblue3
steelblue
tan
turquoise
violet
white
yellow
yellowgreen

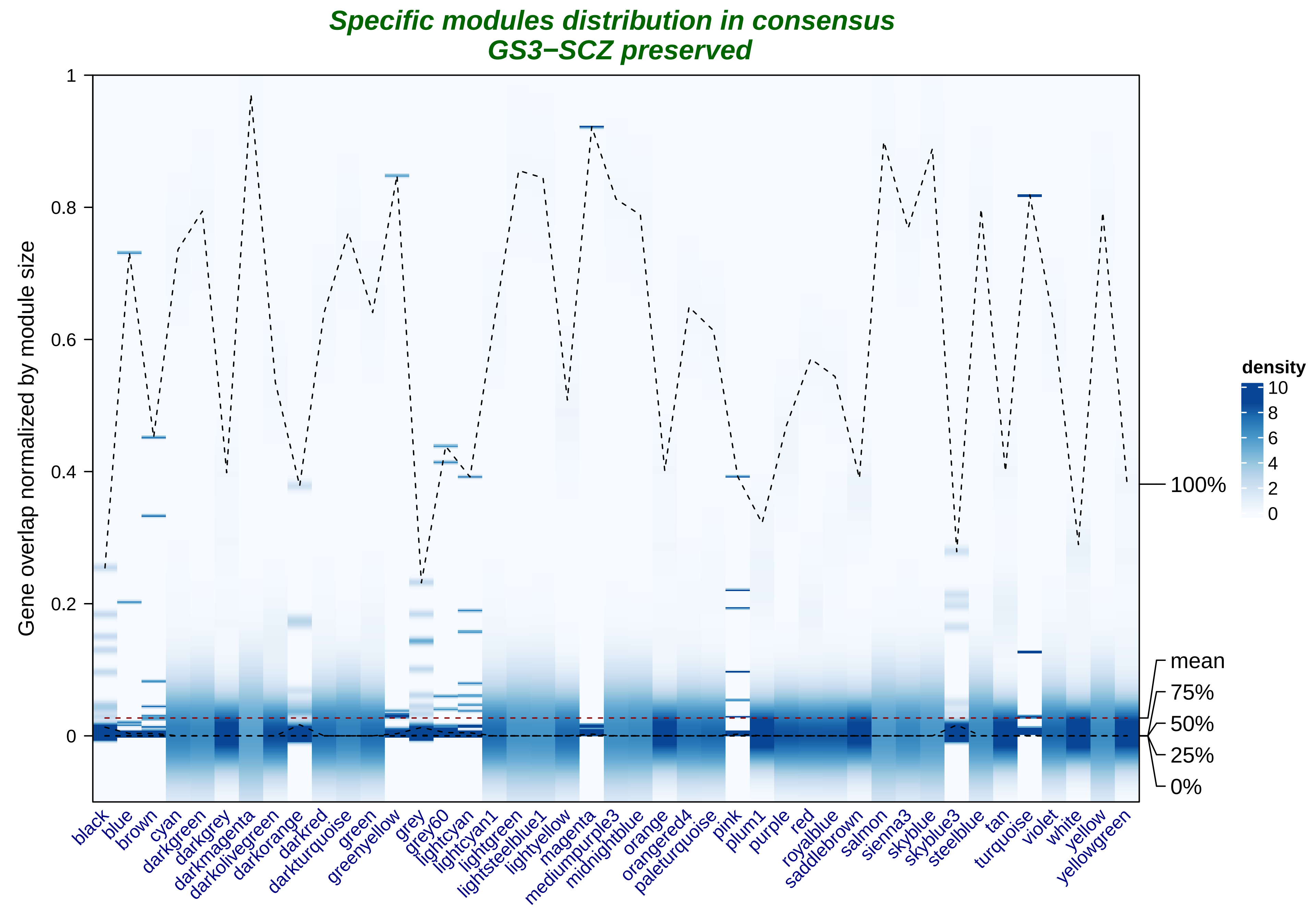
Specific modules distribution in consensus
GS3−SCZ preserved
Gene overlap normalized by module size
1
0.8
0.6
0.4
0.2
0
density
10
8
6
4
2
0
100%
mean
75%
50%
25%
0%
black
blue
brown
cyan
darkgreen
darkgrey
darkmagenta
darkolivegreen
darkorange
darkred
darkturquoise
green
greenyellow
grey
grey60
lightcyan
lightcyan1
lightgreen
lightsteelblue1
lightyellow
magenta
mediumpurple3
midnightblue
orange
orangered4
paleturquoise
pink
plum1
purple
red
royalblue
saddlebrown
salmon
sienna3
skyblue
skyblue3
steelblue
tan
turquoise
violet
white
yellow
yellowgreen

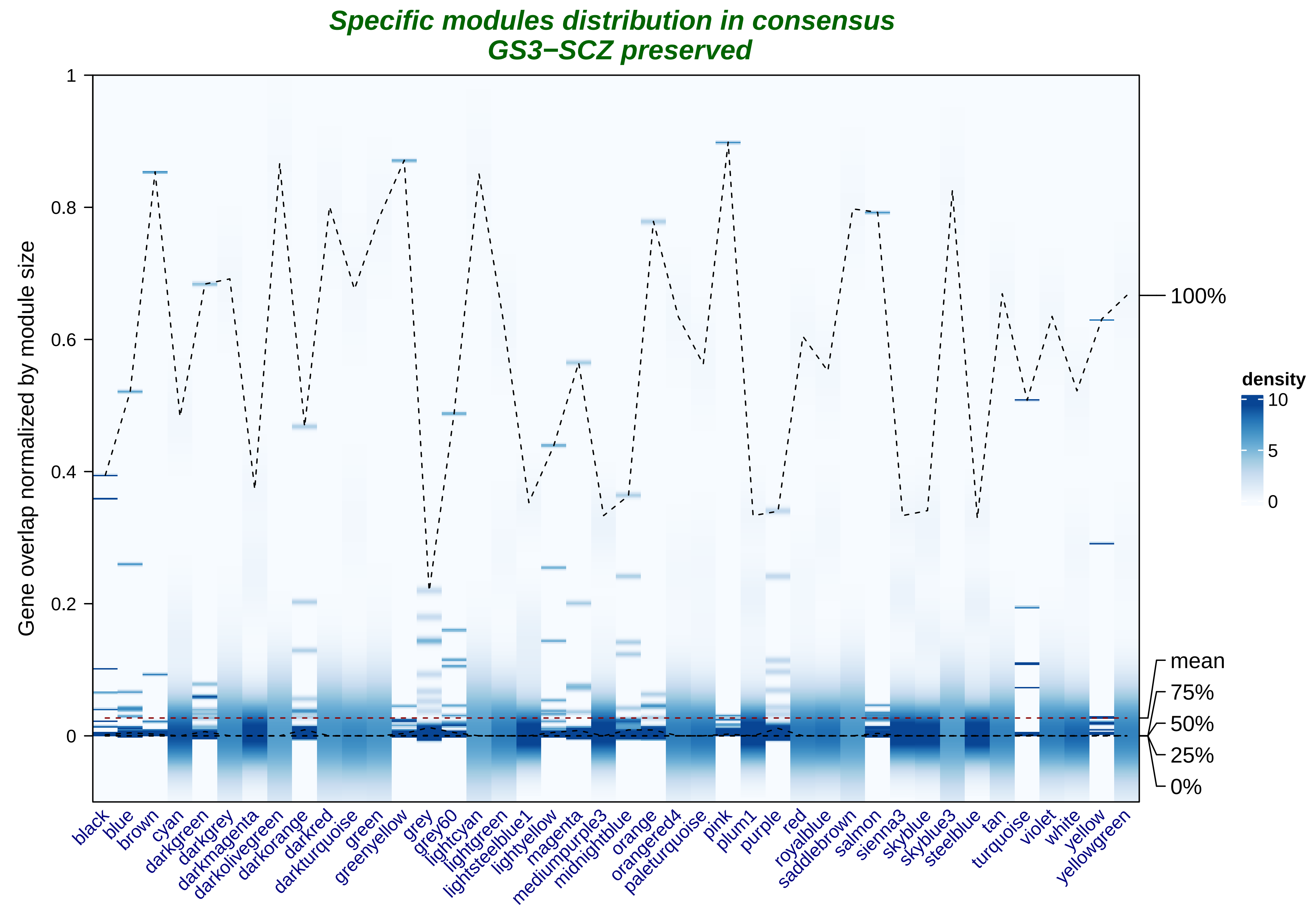

Specific modules distribution in consensus
GS3−SCZ preserved
Gene overlap normalized by module size
1
0.8
0.6
0.4
0.2
0
density
10
5
0
100%
mean
75%
50%
25%
0%
black
blue
brown
cyan
darkgreen
darkgrey
darkmagenta
darkolivegreen
darkorange
darkred
darkturquoise
green
greenyellow
grey
grey60
lightcyan
lightgreen
lightsteelblue1
lightyellow
magenta
mediumpurple3
midnightblue
orange
orangered4
paleturquoise
pink
plum1
purple
red
royalblue
saddlebrown
salmon
sienna3
skyblue
skyblue3
steelblue
tan
turquoise
violet
white
yellow
yellowgreen

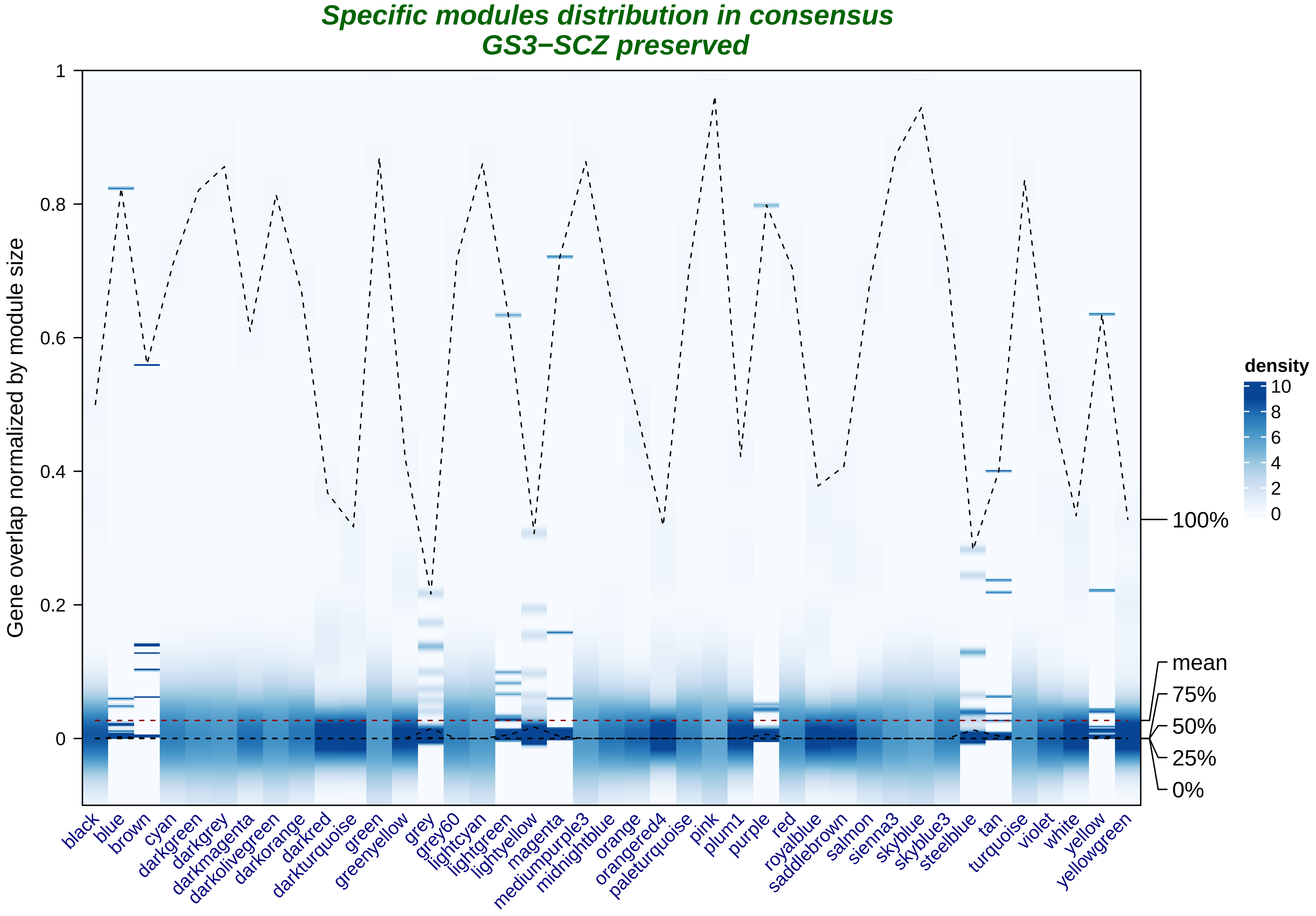

Specific modules distribution in consensus
GS3−SCZ preserved
Gene overlap normalized by module size
1
0.8
0.6
0.4
0.2
0
density
10
8
6
4
2
0
100%
mean
75%
50%
25%
0%
black
blue
brown
cyan
darkgreen
darkgrey
darkmagenta
darkolivegreen
darkorange
darkred
darkturquoise
green
greenyellow
grey
grey60
lightcyan
lightgreen
lightyellow
magenta
mediumpurple3
midnightblue
orange
orangered4
paleturquoise
pink
plum1
purple
red
royalblue
saddlebrown
salmon
sienna3
skyblue
skyblue3
steelblue
tan
turquoise
violet
white
yellow
yellowgreen

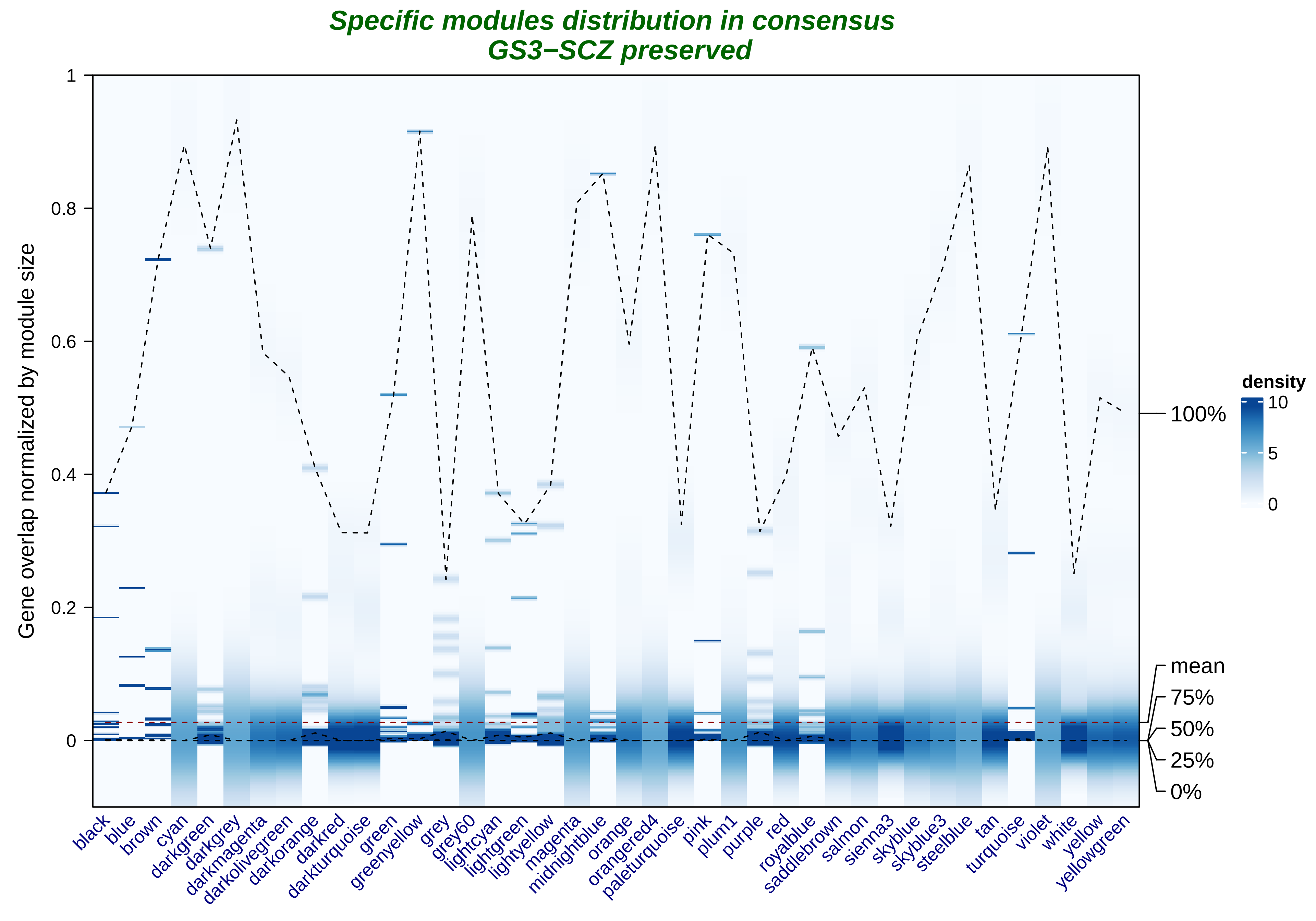

Specific modules distribution in consensus
GS3−SCZ preserved
Gene overlap normalized by module size
1
0.8
0.6
0.4
0.2
0
density
10
5
0
100%
mean
75%
50%
25%
0%
black
blue
brown
cyan
darkgreen
darkgrey
darkmagenta
darkolivegreen
darkorange
darkred
darkturquoise
green
greenyellow
grey
grey60
lightcyan
lightgreen
lightyellow
magenta
midnightblue
orange
orangered4
paleturquoise
pink
plum1
purple
red
royalblue
saddlebrown
salmon
sienna3
skyblue
skyblue3
steelblue
tan
turquoise
violet
white
yellow
yellowgreen
